# Comparison of demographic characteristics and psychiatric comorbidity among methamphetamine-, heroin- and methamphetamine-heroin co- dependent males in Hunan, China

**DOI:** 10.1186/s12888-017-1346-7

**Published:** 2017-05-12

**Authors:** Huixi Dong, Mei Yang, Liang Liu, Chenxi Zhang, Mengqi Liu, Yidong Shen, Huanzhong Liu, Wei Hao

**Affiliations:** 1 Department of Psychiatry & Mental Health Institute of the Second Xiangya Hospital, Central South University, National Clinical Research Center on Mental Disorders & National Technology Institute on Mental Disorders, Hunan Key Laboratory of Psychiatry and Mental Health, Changsha, Hunan 410011 China; 2 Shenzhen Kangning Hospital, Shenzhen, Guangzhou 518020 China; 3 Wuxi Mental Health Center, Nanjing Medical University, Wuxi, Jiangsu 214063 China; 4 Chaohu Hospital of Anhui Medical University, Hefei, Anhui 238000 China

**Keywords:** Comorbidity, DSM-iv, Dependence, Heroin, Methamphetamine, Co-dependence

## Abstract

**Background:**

There is little research of psychiatric comorbidity differences among people with different types of drug dependence in Chinese population. We explored demographic and comorbid psychiatric differences among methamphetamine- dependent males (MDs), heroin-dependent males (HDs) and methamphetamine and heroin co-dependent males (M/HDs) in Hunan province, China.

**Methods:**

A cross-sectional, structured and clinical interview method was used to examine differences in DSM-IV-TR Axis I Disorders among 346 MDs, 698 HDs and 247 M/HDs from three compulsory rehabilitation centers and two voluntary rehabilitation centers in Hunan.

**Results:**

MDs and M/HDs were younger, more likely to choose inhalation administration, less likely to have a family history of substance use, less likely to have undergone detoxification treatment, had higher incomes and shorter duration of drug use than HDs. Overall, methamphetamine-dependence related to higher rates of current and lifetime psychotic disorders, lifetime hallucinogen use disorders. Heroin-dependence related to higher rates of current and lifetime substance-induced mood disorders, sedative/hypnotic/anxiolytic and other drug use disorders and current alcohol use disorder. For M/HDs, they were more likely to have any other lifetime substance use disorders than MDs and HDs.

**Conclusions:**

There were substantial differences in epidemiological characteristics and comorbidity among MD, HD and M/HD groups, which highlights the urgent need to develop treatment services and policies for drug-specific users in China.

**Electronic supplementary material:**

The online version of this article (doi:10.1186/s12888-017-1346-7) contains supplementary material, which is available to authorized users.

## Highlights

We explored demographic and comorbidity differences in the major types of drug use populations in China.

This was a large population-based and psychiatrist-conducted study, which assessed the full range of mental disorders.

These findings can be used to help improve diagnostic rates and to help develop targeted interventions.

## Background

Since the reforms and opening of China began in the late 1980s, the production and use of illicit drugs has dramatically increased [45]. Heroin was, and remains, the most commonly illicit drug. Despite a crackdown on the use of opiates in the last decade, heroin use has not been curtailed. Concurrently, there have been a substantial increase in the use of synthetic drugs, particularly methamphetamine (MA), so-called “magu” pills—an MA derivative; 3,4-methylenedioxymethamphetamine (MDMA; Ecstasy) and ketamine [14]. Since national data on synthetic drugs use was first published in 2004, the percentage of registered users using heroin decreased from 81.1% to 41.8% at the end of 2015. Over the same period, the percentage of registered users using synthetic drugs increased from 9.5% to 57.1% [3, 4]. Between 2008 and 2012, heroin was the most commonly used illicit drug in China, followed by MA, either in crystalline form or taken as pills [47]. China currently faces dual drug use epidemics. Moreover, during the drug conversion, there was also a co-use of MA and heroin problem, and comorbidity in M/Ds is still poorly understood. Given the seriousness of MA and heroin use in China, it is urgent to conduct a detailed and up to date clinical investigation of these drug users.

MA and heroin differ greatly in many respects, including pharmacological features, patterns of use, clinical effects, and withdrawal symptoms [23, 26] and drug users with comorbidities have greater psychosocial and medical problems, poorer prognoses, and higher rates of relapse than those with only substance use disorder (SUD) [2, 9, 12, 25, 35, 46]. Thus, we suspect that different comorbidities might exist among different drug addicts. Research on comorbidity with diverse types of drug use populations is not rare in other countries, but is still blank in China. Because of the ethnic and racial differences associated with mental disorders, the limitation of survey-based studies in China indicate a need for examination of comorbidities in Chinese drug users.

For convenience, we selected study settings in Hunan province, which has borders with six other provinces and is heavily involved in the trafficking of drugs between the two major drug-importing provinces (Yunnan province and Guangdong province) and from other parts of China. In addition, we focused on male population, because the number of registered drug users was 6 times greater for males than for females in China [4] and there are gender-related differences in prevalence of comorbid psychiatric disorders [13, 18]. So we explored these problems by comparing demographic and drug use characteristics, and current and lifetime prevalence of comorbid DSM-IV-TR Axis I mental disorder diagnoses among MDs, HDs and M/HDs. We believed that the findings yielded in this study may help to improve diagnostic rates; treatment services, and the development of targeted interventions aimed at MA-, heroin-and MA and heroin co-dependent users throughout China.

## Method

### Study settings and participants

Participants were recruited from two sources. Some came via the public security system because they had been referred to compulsory rehabilitation centers (CRCs), which are the major treatment modality for drug users and responsible for detoxifying arrested drug users. Others came via the healthcare system because they were attendees of voluntary rehabilitation centers (VRCs), which are designated centers for treating substance addiction. Both the compulsory and voluntary settings are restrictive environments with structural and regulatory procedures to control or eliminate access to drugs. The relapse rate of drug users in the both CRCs and VRCs was at least 80% and many of them continue in a cycle of relapse and compulsory or voluntary rehabilitation. The participants came from urban and from rural backgrounds. We avoided recruiting the same participant at different times and from different sources by screening their name, birthday and personal identification numbers. Consecutive current MA-dependent males were recruited from Hunan Bainihu and Xinkaipu CRCs and from Hunan Kangda VRC between March 2013 and January 2014. Consecutive current heroin-dependent males were recruited from Xinkaipu and Baimalong CRCs and from the Hunan Second People’s Hospital VRC between March and October 2008.

### Procedures

The research protocol and consent procedures were approved by the Human Ethics Committee of the Second Xiangya Hospital of Central South University. Drug use is illegal in China. The psychiatrists responsible for recruiting emphatically explained to potential participants that their legal status would not be affected by participation. A signed informed consent form was obtained from each participant. The inclusion criteria were: over 18 years of age; admission in CRCs or VRCs because of using MA or heroin; mainly using MA or heroin and meeting the DSM-IV-TR criteria for MA or heroin dependence in the year preceding admission to the CRCs or VRCs; not having used MA or heroin together in the year preceding admission; capable of effective communication. Those who were current MA dependent with previous heroin dependence and current heroin dependent with previous MA dependence were classified as M/HDs. We recorded the name and personal identification number (5 or 6 digits in CRCs, 18 digits in VRCs) of every subjects before we started our interview. Anyone who had participated in this survey before was excluded.

Participants underwent a two-stage assessment. Face-to-face interviews were conducted by two groups of four psychiatrists. Demographic information and data about drug use-related conditions were obtained using a locally designed format. Current and lifetime diagnoses were obtained using the Structured Clinical Interview for DSM-IV-TR Axis I Disorders—Patient Edition (SCID-I/P), Chinese version [16, 40] (Additional file 1). The Chinese version of the SCID-I had shown excellent test–retest reliability; and the Kappa values ranged from 0.937 to 0.981 [40]. In addition, DSM-IV assumes universality of standard diagnostic criteria with very little regard to cross-cultural variability [33]. Each psychiatrist had received interview training from the primary investigator and we have good consistency.

The DSM-IV-TR psychiatric disorders diagnoses included psychotic disorders, mood disorders, anxiety disorders, somatoform disorders, and eating disorders. Each psychiatric disorder was then classified as independent psychiatric disorder or substance-induced psychiatric disorder. In the DSM-IV, the term “independent” or “primary” is used to indicate mental disorders that are not substance-induced and that are not due to general medical conditions. The DSM-IV-TR differentiates independent and substance-induced psychotic disorders using the following guidelines. A diagnosis of independent psychosis should be not due to the direct physiological effects of a substance (e.g., a drug of abuse or a medication) or of a general medical condition. For a substance-induced-psychotic diagnosis, the following criteria must be met: the symptoms [1] are etiologically related to the substance use; [2] do not precede the onset of the substance abuse or dependence; [3] persist for less than a substantial period of time (e.g., about a month) after the cessation of acute withdrawal or severe intoxication; [5] are not substantially in excess of what would be expected given the type or amount of the substance used or the duration of use; and [6] do not occur exclusively during the course of delirium. Substance use disorders (SUDs) diagnoses consisted of alcohol use disorder (abuse or dependence on alcohol) and drug-specific use disorders (abuse or dependence on an illicit drug). Drug-specific use disorders included several classes: sedative/hypnotic/anxiolytic use disorder; cannabis use disorder; hallucinogen use disorder and other drug use disorders.

Interviews with participants in CRCs were administered no earlier than 7 days after admission; the longest interval between admission and interview was 6 months. Interviews with participants in VRCs were administered within 14 days of admission. So their acute withdrawal symptoms had passed and they had relatively good physical and mental state. Lifetime prevalence is the proportion of the population who, at beginning in life up to the time of assessment, has ever had any mental disorders. Current prevalence is a period prevalence in our study. Because participants were enrolled in relatively different time, for reconciling the time of the survey and avoiding any influence of arrest, hospitalization and isolation, the current diagnoses were defined in this study as meeting the diagnostic criteria in the last month before admission in CRCs or VRCs. Generalized anxiety includes current state in the Chinese version of SCID-I; thus, the four “Life Prevalence” columns in the “Generalized Anxiety” row of Table 2 are blank. Each interview lasted from 2 to 3 h.

### Statistical analyses

Data were analyzed using SPSS version 19.0. Demographic and drug use variables, and lifetime and current prevalence of DSM-IV-TR diagnoses (Axis I) were calculated and presented in cross-tabulations. Data were presented by three groups based on MA-; heroin-; MA and heroin co-dependence. Simple descriptive statistics were expressed as “mean and standard deviation” for continuous variables; “frequency and percentage” for categorical variables. Differences in demographic and drug use variables among groups were tested using t test, ANOVA test or Pearson’s χ2 test. Furthermore, lifetime and current prevalence of psychiatric disorders and SUDs between groups were compared using binary logistic regression analysis between each two groups. Each disorder was entered as the dependent variable with the “Enter” method after the potentially confounding effects of variables that significantly differed in univariate analyses had been controlled for. To adjust for multiple comparisons the significance level was set to *p* < 0.01 (two-tailed) for all comparisons in the study.

## Results

### Demographic data and drug-use characteristics

After an initial screening, 19 MA users and 8 heroin users were excluded for not meeting DSM-IV-TR criteria for MA dependence or heroin dependence: they were diagnosed with abuse but not dependence. 86% of subjects agreed to participate in this study. Thirty-one (2.3%) of the 1322 eligible participants did not complete the interview, because they felt tired or sleepy, or for no explicit reason, and they opted to withdraw from the study. A final sample of 346 MDs, 698 HDs and 247 M/HDs participated; 315 MDs, 591HDs and 230 M/HDs were recruited from CRCs. 206 (83.4%) M/HDs were current MA dependent with previous heroin dependence and the rest were current heroin dependent with previous MA dependence. Comparing with HDs, MDs and M/HDs were younger, less likely to have a family history of substance use, less likely to have undergone detoxification treatment and had shorter durations of drug use than HDs, and there was no significant difference between MDs and M/HDs in these variables. Incomes and main administration route in the past years differed in each pairwise comparisons. MDs had the highest income state, followed by M/HDs and HDs. MDs all chose nasal inhalation as the main administration route, but most HDs chose injection. No significant differences were found in education, marital status and employment rates among three groups. (Table 1).

**Table 1:** Demographic characteristics

Characteristics﻿, n (%)	MDs (*n* = 346)	HDs (*n* = 698)	M/HDs (*n* = 247)	*F/χ2*	*P*
Age (M ± SD years)^a,c^	32.07 ± 7.19	33.33 ± 6.68	31.93 ± 7.03	5.86	**0.003**
Education				5.63	0.228
Less than junior high school	81 (23.41)	134 (19.20)	61 (24.70)		
Junior high school	192 (55.49)	390 (55.87)	134 (54.25)		
Senior high school or higher	73 (21.10)	174 (24.92)	52 (21.05)		
Marital status				8.93	0.063
Married, remarried, or cohabiting	143 (41.33)	236 (33.81)	90 (36.44)		
Widowed, separated, or divorced	67 (19.36)	132 (18.91)	56 (22.67)		
Never married	136 (39.31)	330 (47.28)	101 (40.89)		
Employment					
Unemployed/illegal work	174 (50.29)	353 (50.57)	126 (51.01)	0.03	0.985
legal work	172 (49.71)	345 (49.93)	121 (48.99)		
Income past year (¥)^a,b,c^				222.76	**<0.001**
0–9999	15 (4.34)	256 (36.68)	28 (11.34)		
10,000–29,999	33 (9.54)	129 (18.48)	41 (16.60)		
30,000–99,999	152 (43.93)	210 (30.09)	104 (42.11)		
> 100,000	146 (42.20)	103 (14.76)	74 (29.96)		
Duration of drug use (M ± SD, years)^a,c^	8.23 ± 11.51	10.00 ± 4.63	7.79 ± 5.01	12.10	**<0.001**
Main administration route (past-year)^a,b,c^				721.78	**<0.001**
Injection	0	138 (19.77)	211 (85.43)		
Nasal inhalation	346(100.00)	560 (80.23)	36 (14.57)		
Previous detoxification treatment^a,c^				243.98	**<0.001**
Yes	187 (54.04)	642 (91.98)	133 (53.85)		
No	159 (45.95)	56 (8.02)	114 (46.15)		
Family history of substance use^a,c^				90.42	**<0.001**
Yes	65 (18.79)	312 (44.70)	52 (21.05)		
No	281 (81.21)	386 (55.30)	195 (78.95)		

*MDs* Methamphetamine dependent subjects

*HDs* heroin- dependent subjects

*M/HDs* Dual methamphetamine- and heroin- dependent subjects

^a^MDs compared with HDs < 0.01

^b^MDs compared with M/HDs < 0.01

^c^HDs compared with M/HDs < 0.01

¥,Chinese yuan

### Prevalence of DSM-IV-TR psychiatric Comorbidity between MA-dependent and heroin-dependent groups

For psychiatric disorders, there were significant differences in some psychiatric comorbidities among groups. Overall, MDs and M/HDs both had a higher prevalence of current and lifetime psychotic disorders (both independent and substance-induced). No significant differences were found between these two groups and schizophrenia was the main disorder in independent psychotic disorders. HDs were more likely to have current and lifetime substance-induced mood disorders. There was no statistically significant differences in independent mood disorders, any anxiety disorders, somatoform disorders and eating disorders among the three groups respectively. (Table 2).

**Table 2:** Comparison of current and lifetime prevalence of DSM-IV-TR Axis I mental disorders between groups

Diagnoses, n (%)	Current									Lifetime								
	MDs (*n* = 346)	HDs (*n* = 698)	M/HDs (*n* = 247)	MDs vs.HDs		MDs vs. M/HDs		HDs vs. M/HDs		MDs (*n* = 346)	HDs (*n* = 698)	M/HDs (*n* = 247)	MDs vs.HDs		MDs vs. M/HDs		HDs vs. M/HDs	
				Wald	P	Wald	P	Wald	P				Wald	P	Wald	P	Wald	P
Psychotic																		
Independent Psychotic Disorder	24 (6.94)	5 (0.72)	20 (8.10)	21.61	**<0.001**	16.27	0.595	24.43	**<0.001**	31 (8.96)	6 (0.86)	28 (11.34)	29.72	**<0.001**	0.91	0.342	34.71	**<0.001**
Schizophrenia	13 (3.76)	3 (0.43)	13 (5.26)							19 (5.49)	4 (0.57)	17 (6.88)						
Schizophreniform	1 (0.29)	0	0							1 (0.29)	0	1 (0.40)						
Schizoaffective	1 (0.29)	1 (0.14)	1 (0.40)							2 (0.58)	1 (0.14)	1 (0.40)						
Other Psychotic Disorders	8 (2.31)	1 (0.14)	6 (2.43)							9 (2.60)	1 (0.14)	9 (3.64)						
Substance-induced	118 (34.10)	4 (0.57)	78 (31.58)	75.23	**<0.001**	0.42	0.519	71.98	**<0.001**	122 (35.26)	12 (1.72)	80 (32.39)	118.40	**<0.001**	0.53	0.467	106.26	**<0.001**
Mood Disorders																		
Independent Mood	17 (4.91)	66 (9.46)	18 (7.29)	6.34	0.012	1.45	0.229	0.99	0.321	49 (14.16)	117 (16.76)	42 (17.00)	0.59	0.329	0.89	0.344	0.00	0.994
Major Depressive	14 (4.05)	54 (7.74)	15 (6.07)							38 (10.98)	96 (13.75)	38 (15.38)						
Dysthymic	1(0.29)	6(0.86)	1 (0.40)							2 (0.58)	12 (1.72)	1 (0.40)						
Bipolar I	0	3(0.43)	1 (0.40)							5 (1.45)	4 (0.57)	2 (0.81)						
Bipolar II	0	0	0							2 (0.58)	1 (0.14)	0						
Other Mood Disorders	2 (0.58)	2	1 (0.40)							2 (0.58)	4 (0.57)	1 (0.40)						
Substance-induced mood	2 (0.58)	40 (5.73)	2 (0.81)	12.21	**<0.001**	0.11	0.735	7.89	**0.005**	36 (10.40)	134 (19.19)	32 (12.96)	10.80	**0.001**	0.92	0.337	3.49	0.062
Anxiety																		
Independent Anxiety	11 (0.59)	49 (7.02)	6 (2.43)	5.20	0.023	0.29	0.591	6.09	0.014	19 (5.49)	77 (11.03)	13 (5.26)	6.30	0.012	0.02	0.904	5.93	0.015
Panic with Agoraphobia	0	1 (0.14)	0							1 (0.29)	1 (0.14)	1 (0.40)						
Panic without Agoraphobia	0	8 (1.15)	0							1 (0.29)	9 (1.29)	0						
Social Phobia	2 (0.58)	7 (1.00)	0							3 (0.87)	9 (1.29)	1 (0.40)						
Specific Phobia	0	4 (0.57)	2 (0.81)							0	5 (0.71)	3 (0.43)						
Obsessive-Compulsive	7(2.02)	11 (1.58)	1 (0.40)							11 (3.18)	13 (1.86)	3 (0.43)						
Posttraumatic Stress	0	8 (1.15)	2 (0.81)							2 (0.58)	25 (3.58)	6 (2.43)						
Generalized Anxiety	3 (0.87)	13 (1.86)	3 (0.43)															
Anxiety NOS	0	3(0.43)	0							0	3 (0.43)	0						
Substance-induced Anxiety	4 (1.16)	9 (1.29)	1 (0.40)	0.16	0.690	0.89	0.346	0.99	0.319	19 (5.49)	21 (3.01)	8 (3.24)	4.44	0.035	1.65	0.200	0.00	0.956
Somatoform	2(0.58)	11 (1.58)	1 (0.40)	2.86	0.091	0.06	0.812	2.60	0.107	2 (0.58)	13 (1.86)	1 (0.40)	3.45	0.056	0.06	0.812	3.09	0.079
Eating	0	3 (0.43)	1 (0.40)							0	3(0.43)	1 (0.40)						
Substance Use Disorders (SUDs)																		
Alcohol	8 (2.31)	115 (16.48)	13 (5.3)	29.28	**<0.001**	3.48	0.062	15.31	**<0.001**	95 (27.46)	206 (29.51)	91 (36.84)	0.25	0.617	5.86	0.015	6.07	0.014
Abuse	2(0.58)	96(13.75)	9 (1.29)							51 (14.74)	150 (21.49)	41 (16.60)						
Dependence	6(1.73)	19(2.72)	4 (0.57)							44 (12.72)	56 (8.02)	50 (20.24)						
Sedative/Hypnotic/Anxiolytic	0(0)	143 (20.49)	13 (5.26)		**<0.001**		**<0.001**	23.32	**<0.001**	1 (0.29)	228 (32.66)	100 (40.5)	24.96	**<0.001**	29.217	**<0.001**	4.826	0.028
Abuse	0	44(6.30)	3 (0.43)							1 (0.29)	87 (12.46)	11 (4.45)						
Dependence	0	99(14.18)	10							0	141 (20.20)	89 (36.03)						
Cannabis	0 (0)	1 (0.14)	2 (0.81)					2.758	0.097	10 (2.89)	5 (0.72)	20 (8.10)	5.05	0.025	7.484	**0.006**	19.60	**<0.001**
Abuse	0	0	2 (0.81)							3 (0.87)	3 (0.43)	9 (3.64)						
Dependence	0	1(0.14)	0							7 (2.02)	2(0.29)	11 (4.45)						
Hallucinogen	5 (1.45)	10 (1.43)	23 (9.3)	0.10	0.751	15.10	**<0.001**	28.06	**<0.001**	81 (23.41)	63 (9.03)	95 (38.46)	33.25	**<0.001**	15.40	**<0.001**	93.67	**<0.001**
Abuse	0	0	0							0	8 (0.29)	0						
Dependence	5 (1.45)	2 (0.29)	3 (0.43)							62 (17.92)	7 (1.00)	58 (23.48)						
Others Substances	0 (0)	19 (2.72)	2 (0.81)					2.58	0.108	5 (1.45)	46 (6.59)	13 (5.26)	8.465	**0.004**	6.25	**0.012**	0.733	0.392
Abuse	0	8 (0.29)	2 (0.81)							3 (0.87)	18 (2.58)	2 (0.81)						
Dependence	0	1 (1.15)	0							2 (0.58)	28 (4.01)	11 (4.45)						

*MDs* Methamphetamine dependent subjects

*HDs* heroin- dependent subjects

*M/HDs* Dual methamphetamine- and heroin- dependent subjects

NOS, not otherwise specified

Data adjusted for potential confounding effects of age, education and marital status

### Prevalence of DSM-IV-TR SUDs between MA-dependent and heroin-dependent groups

MDs and M/HDs both had higher prevalence of current and lifetime hallucinogen use disorders. The rate of lifetime hallucinogen use disorders in M/HDs was two and four times greater than rates in M/HD and HDs. In addition, M/HDs were tended to have higher any lifetime substance use disorders. HDs had a significantly greater prevalence of current alcohol use disorders; current and lifetime sedative/hypnotic/anxiolytic use disorders and life other substance use disorders. Except for alcohol use disorders, all the three groups tended to have higher proportion of substance dependence than abuse. (Table 2).

## Discussion

To our knowledge, this is the first study that compares the prevalence of DSM-IV-TR Axis I disorders in MA-, heroin- and co-dependent males in China. MDs and M/HDs tended to be slightly younger and started using drug at an earlier age (Percentage of onset age of drug use before 25: 33.8% in MDs, 35.6% in M/HDs and 24.6% in HDs). MA might be more available to and fashionable among teenagers and young adults. Barati et al. [5] reported that for nearly half of MA users, the mean drug-abuse initiation age was <18 years old. In addition, Li et al. [30] reported being ≤25 years old was an independent factor associated with current MA use in a China-Myanmar border region. The percentage of married, remarried, or cohabiting users in our study (33.81–41.33%) was as low as, based on Grella et al. [20], who reported that 49% of their 578 participants were in one these categories, and on Salo et al. [43], who reported that 28% of their 189 participants were also.

Interestingly, we found that nasal inhalation was ubiquitous among our MDs and M/HDs, but not among HDs. Routes of drug administration vary widely by regions. The report of National Survey on Drug Use and Health between 2005 to 2007 in the United States claimed that 50.0% heroin-dependent and 13% of MA-dependent users focused on injection [36]. In our study, about 80% of M/HDs were single current MA addicts with previous heroin dependence, due to the varied epidemic trend of drug use in China. So most M/HDs chose nasal inhalation because they were MA users at the time of interview. Therefore, we can conclude that most current MA dependence users chose nasal inhalation as the main administration. Inhalation and injecting deliver a similarly rapid drug effect and high bioavailability [11], but the former was perceived to be less harmful. The benefit of inhaling rather than injecting MA is the potential to reduce the transmission of blood-borne viruses, including HIV [32]. Another reason might be some MA users took MA tablets with low purity alone or take MA tablets combined with crystalline MA in our study. Further studies are needed to find out the related causes of this phenomenon. For HDs, because of tolerance developed after long use of heroin, so injection can help them get a rush of euphoria quickly.

We also found that, our HDs were at a lower socioeconomic level than MDs and M/HDs. In addition, HDs had used drugs for longer and had spent more time in detoxification, which is consistent with heroin’s being more addictive. The dependence ranking of heroin is higher than that of amphetamine (mean score: 3.00 vs. 1.67), both psychologically and physically [39]. Moreover, longitudinal research [21] has shown that heroin-dependent users are less likely than those dependent on MA or cocaine to reduce or cease their use in the 10 years after they begin using these drugs, which suggest a more protected course of addiction. Injecting drugs is associated with a lower perceived state of general health, a higher prevalence of major depressive episodes, and higher rates of cardiorespiratory arrest [36]. Situation in M/HDs were similar as in MDs, the possible reason was M/HDs were most current MA users and might have longer duration MA use than heroin use.

Not unexpectedly, after we had controlled for demographics and duration of drug use, we found high prevalence rates of comorbid mental disorders in both groups. Generally, psychotic disorders were more common among MDs and H/MDs. The incidence of current MA induced psychotic disorders range from 20.08% to 31.4% in subjects with MA use disorders in previous cross-sectional studies [27, 31, 43, 49]. There might be a genetic predisposition to MA-induced psychosis [22]. Other distinctive demographic predictors are being male gender, being unemployed and being single, and having low level of education [15]. Indeed, this might also be the case for some other drug use behaviors, including early age at onset, a high-volume [8], long duration, high frequency of use [48], sexual abuse, a family history of drug use, other substance use, and co-occurring personality and mood disorders [15, 19, 28]. However, the relative importance of genetic predisposition and of other etiological factors remains unclear. The symptoms of psychosis induced by MA are very similar to those of schizophrenia spectrum psychosis and include: lack of concentration, delusions of persecution, increased motor activity, disorganization of thoughts, lack of insight, anxiety, suspicion and auditory hallucinations [6]. 20%–38% of the subjects with MA induced psychotic disorders in the past had a change in diagnosis to either schizophrenia or affective psychosis on follow-up (Aggarwal et al. 2012; [24]). MA use was vulnerability to independent psychotic disorders. Positive psychotic symptoms of MA induced psychotic disorders can be resolved rapidly a few days after MA cessation and/or antipsychotic treatment. Glasner-Edwards et al. [17] reported that early treatment of psychotic symptoms in MA-dependent adults was associated with a better outcome at a 3-year follow-up. This highlights the importance of being aware of comorbidities in MA-dependent patients and the need for timely and integrated treatments for such disorders, regardless of whether they are substance-induced.

MDs, HDs and M/HDs were particularly susceptible to mood disorders is noteworthy. In contrast, HDs were particularly more likely to have current substance-induced mood disorders than MDs and H/MDs. But no difference was found in lifetime rate of substance-induced mood disorders between HDs and H/MDs, which suggested that heroin use was highly relevant to mood alteration. Intense euphoria and well-being appear in heroin intoxication. Symptoms during withdrawal vary—someone feel anxious and agitated, while others experience temporary depression and anhedonia. Long-term use of opioids is associated with moderate to severe depression. Whereas MA intoxication is associated with euphoria, well-being, and perceived increased powers of thought, strength, and accomplishment [7, 42, 44]. In a follow-up study, current substance induced depression at baseline were found to be more prevalent in heroin dependent subjects than those who dependent on alcohol or cocaine [41]. The association between DSM-IV lifetime any mood disorders and opioid dependence (OR = 10.5) was found greater than the corresponding association for amphetamine dependence (OR = 6.9) in males [10]. Clinical recommendations for substance induced mood disorders may include watchful waiting and abstinence prior to the treatment of depression, whereas for independent mood disorders diagnosed among substance users, clinical recommendations may include immediate pharmacological treatment with concurrent psychotherapy [37]. Primary treatments for opioid dependence (e.g., methadone or buprenorphine maintenance or residential treatment) can substantially improve depression. Studies of antidepressant medications have produced mixed results, some positive but more negative [38].

A particular challenge is the frequency with which MDs, HDs and M/HDs have problems with abuse of and dependence on alcohol and other substances. Those who dependent both on heroin and MA particularly had more other SUDs histories suggested they had tried and altered different types of substance in their lifetime. Current prevalence of any other SUDs was not as high as lifetime prevalence showed poly-substance users were not too many in M/HDs. Based on our findings, these phenomena might also reflect their drugs of choice, which allow those who favor opioids and hallucinogens also use stimulants, when available. It is worth mentioning that current alcohol use disorders were common in HDs while lifetime alcohol use disorders were not different between HDs and MDs, but more prevalent in H/MDs. The frequent concurrent use of alcohol, sedatives, hypnotics, and anxiolytics by heroin-dependent users might be a consequence of their taking these substances to feel better, to promote sleep, to relieve depression, trait anxiety and related symptomatology and to avoid withdrawal symptoms [29, 34]. The finding that MA dependent users (MDs and H/MDs) had higher rate of hallucination use disorders, but not for HDs, confirmed the transition from traditional drugs (such as opioid or sedative) to synthetic drugs. The trend of illicit drug use was strongly influenced peoples’ choice on drugs.

Our study has some limitations. The sample was not randomly selected, which might limit the generalizability of the findings. Some of the data, such as drug history, were collected from self-reports, and the diagnoses of current DSM-IV-TR Axis I disorders were made at the time of admission to the CRC or VRC. These data might, therefore, have been affected by a social desirability bias and recall bias, particularly in cases where the onset of drug use was many years before the interview. In addition, we did not record the participants’ tobacco use. Next, the old DSM diagnose criteria was used in this study. The new Diagnostic and Statistical Manual of Mental Disorders, 5th Edition (DSM-5) has some changes to addictions compared to DSM-IV-TR. The major change with substance abuse and alcohol abuse and dependence disorders has been the removal of the distinction between “abuse” and “dependence”. There are also two changes to the DSM-5 criteria for substance use disorder. “Recurrent legal problems” criterion for substance abuse has been deleted. And a new criterion has been added: craving or a strong desire or urge to use a substance”. Moreover, severity of the DSM-5 substance use disorders is based on the number of criteria endorsed. Finally, this was a cross-sectional survey. We cannot conclude that there is a causal relationship between MA- or heroin-dependence and other covariates, nor can we conclude anything about the direction of those relationships. Longitudinal studies are needed for this.

## Conclusion

This study provides current and lifetime prevalence figures for DSM-IV-TR Axis I mental disorders, drug use patterns, and demographic correlates based on a large population-based study from China. It had a very high participation rate and included a comprehensive, psychiatrist-conducted assessment of the full range of mental disorders. We also carefully distinguished between substance-induced and non-substance-induced psychiatric disorders. The data from this study combined with data from the other studies reviewed in this article suggest that psychiatric comorbidity is a major health concern when treating addiction. It may well be that physicians who use standard drug treatment interventions need to consider concurrently treating of both substance-induced and other Axis I disorders with symptoms such as psychotic symptoms, depression, and anxiety when developing a treatment regimen.

## Additional file

Additional file 1: Structured Clinical Interview for the DSM-IV Axis I Disorders —Patient Edition 20,170,421. (PDF 915 kb)

## Abbreviations

CRCs: Compulsory rehabilitation centers
HDs: Heroin-dependent males
M/HDs: Methamphetamine and heroin co-dependent males
MA: Methamphetamine
MDMA: 3,4-methylenedioxymethamphetamine
MDs: Methamphetamine- dependent males
SCID-I/P: DSM-IV-TR Axis I Disorders—Patient Edition
SUDs: Substance use disorders
VRCs: Voluntary rehabilitation centers

## References

[1] Aggarwal M, Banerjee A, Singh SM, Mattoo SK, Basu D (2012). Substance-induced psychotic disorders: 13-year data from a de-addiction centre and their clinical implications. Asian J Psychiatr.

[2] Aharonovich E, Liu X, Nunes E, Hasin DS (2002). Suicide attempts in substance abusers: effects of major depression in relation to substance use disorders. Am J Psychiatry.

[3] Annual Report on Drug Control in China (2004). National Narcotic Control Commission (NNCC).

[4] Annual Report on Drug Control in China (2016). National Narcotic Control Commission (NNCC).

[5] Barati M, Ahmadpanah M, Soltanian AR (2014). Prevalence and factors associated with methamphetamine use among adult substance abusers. J Res Health Sci.

[6] Bramness JG, Gundersen OH, Guterstam J, Rognli EB, Konstenius M, Loberg EM, Medhus S, Tanum L, Franck J (2012). Amphetamine-induced psychosis--a separate diagnostic entity or primary psychosis triggered in the vulnerable?. BMC Psychiatry.

[7] Center for Substance Abuse Treatment (2005). Substance Abuse treatment for persons with co-occurring disorders.

[8] Chen CK, Lin SK, Sham PC, Ball D, Loh EW, Hsiao CC, Chiang YL, Ree SC, Lee CH, Murray RM (2003). Pre-morbid characteristics and co-morbidity of methamphetamine users with and without psychosis. Psychol Med.

[9] Compton WR, Cottler LB, Jacobs JL, Ben-Abdallah A, Spitznagel EL (2003). The role of psychiatric disorders in predicting drug dependence treatment outcomes. Am J Psychiatry.

[10] Conway KP, Compton W, Stinson FS, Grant BF (2006). Lifetime comorbidity of DSM-IV mood and anxiety disorders and specific drug use disorders: results from the National Epidemiologic Survey on alcohol and related conditions. J Clin Psychiatry.

[11] Cook CE, Jeffcoat AR, Hill JM, Pugh DE, Patetta PK, Sadler BM, White WR, Perez-Reyes M (1993). Pharmacokinetics of methamphetamine self-administered to human subjects by smoking S-(+)-methamphetamine hydrochloride. Drug Metab Dispos.

[12] Curran GM, Sullivan G, Williams K, Han X, Collins K, Keys J, Kotrla KJ (2003). Emergency department use of persons with comorbid psychiatric and substance abuse disorders. Ann Emerg Med.

[13] Evans EA, Grella CE, Washington DL, Upchurch DM (2017). Gender and race/ethnic differences in the persistence of alcohol, drug, and poly-substance use disorders. Drug Alcohol Depend.

[14] Fang YX, Wang YB, Shi J, Liu ZM, Lu L (2006). Recent trends in drug abuse in China. Acta Pharmacol Sin.

[15] Farnia V, Shakeri J, Tatari F, Juibari TA, Bajoghli H, Golshani S (2016). Demographic and mental history-related data predicted occurrence of psychosis in metamphetamine users. Psychiatry Res.

[16] First M, Spitzer R, Gibbon M, Williams J (2002). Structured clinical interview for DSM-IV-TR Axis I disorders.

[17] Glasner-Edwards S, Mooney LJ, Marinelli-Casey P, Hillhouse M, Ang A, Rawson R (2008). Clinical course and outcomes of methamphetamine-dependent adults with psychosis. J Subst Abus Treat.

[18] Goldstein RB, Dawson DA, Chou SP, Grant BF (2012). Sex differences in prevalence and comorbidity of alcohol and drug use disorders: results from wave 2 of the National Epidemiologic Survey on alcohol and related conditions. J Stud Alcohol Drugs.

[19] Grant KM, LeVan TD, Wells SM, Li M, Stoltenberg SF, Gendelman HE, Carlo G, Bevins RA (2012). Methamphetamine-associated psychosis. J NeuroImmune Pharmacol.

[20] Grella CE, Karno MP, Warda US, Niv N, Moore AA (2009). Gender and comorbidity among individuals with opioid use disorders in the NESARC study. Addict Behav.

[21] Hser YI, Evans E, Huang D, Brecht ML, Li L (2008). Comparing the dynamic course of heroin, cocaine, and methamphetamine use over 10 years. Addict Behav.

[22] Ikeda M, Okahisa Y, Aleksic B, Won M, Kondo N, Naruse N, Aoyama-Uehara K, Sora I, Iyo M, Hashimoto R, Kawamura Y, Nishida N, Miyagawa T, Takeda M, Sasaki T, Tokunaga K, Ozaki N, Ujike H, Iwata N (2013). Evidence for shared genetic risk between methamphetamine-induced psychosis and schizophrenia. Neuropsychopharmacology.

[23] Karila L, Weinstein A, Aubin HJ, Benyamina A, Reynaud M, Batki SL (2010). Pharmacological approaches to methamphetamine dependence: a focused review. Br J Clin Pharmacol.

[24] Kittirattanapaiboon P, Mahatnirunkul S, Booncharoen H, Thummawomg P, Dumrongchai U, Chutha W (2010). Long-term outcomes in methamphetamine psychosis patients after first hospitalisation. Drug Alcohol Rev..

[25] Landheim AS, Bakken K, Vaglum P (2006). Impact of comorbid psychiatric disorders on the outcome of substance abusers: a six year prospective follow-up in two Norwegian counties. BMC Psychiatry.

[26] Larney S, Gowing L, Mattick RP, Farrell M, Hall W, Degenhardt L (2014). A systematic review and meta-analysis of naltrexone implants for the treatment of opioid dependence. Drug Alcohol Rev..

[27] Leamon MH, Flower K, Salo RE, Nordahl TE, Kranzler HR, Galloway GP (2010). Methamphetamine and paranoia: the methamphetamine experience questionnaire. Am J Addict.

[28] Lecomte T, Mueser KT, MacEwan W, Thornton AE, Buchanan T, Bouchard V (2013). Predictors of persistent psychotic symptoms in persons with methamphetamine abuse receiving psychiatric treatment. J Nerv Ment Dis.

[29] Leri F, Bruneau J, Stewart J (2003). Understanding polydrug use: review of heroin and cocaine co-use. Addiction.

[30] Li L, Assanangkornchai S, Duo L, McNeil E, Li J (2014). Cross-border activities and association with current methamphetamine use among Chinese injection drug users (IDUs) in a China-Myanmar border region. Drug Alcohol Depend.

[31] McKetin R, McLaren J, Lubman DI, Hides L (2006). The prevalence of psychotic symptoms among methamphetamine users. Addiction.

[32] McKetin R, Ross J, Kelly E, Baker A, Lee N, Lubman DI, Mattick R (2008). Characteristics and harms associated with injecting versus smoking methamphetamine among methamphetamine treatment entrants. Drug Alcohol Rev.

[33] Mezzich JE, Kirmayer LJ, Kleinman A (1999). The place of culture in DSM-IV. J Nerv Ment Dis.

[34] Mitcheson M, Hawks D, Malone S, Davidson J, Hitchens L (1970). Sedative abuse by heroin addicts. Lancet.

[35] Najt P, Fusar-Poli P, Brambilla P (2011). Co-occurring mental and substance abuse disorders: a review on the potential predictors and clinical outcomes. Psychiatry Res.

[36] Novak SP, Kral AH (2011). Comparing injection and non-injection routes of administration for heroin, methamphetamine, and cocaine users in the United States. J Addict Dis.

[37] Nunes EV, Levin FR (2004). Treatment of depression in patients with alcohol or other drug dependence. JAMA.

[38] Nunes EV, Sullivan MA, Levin FR (2004). Treatment of depression in patients with opiate dependence. Biol Psychiatry.

[39] Nutt D, King LA, Saulsbury W, Blakemore C (2007). Development of a rational scale to assess the harm of drugs of potential misuse. Lancet.

[40] Phillips MR, Zhang J, Shi Q, Song Z, Ding Z, Pang S, Li X, Zhang Y, Wang Z (2009). Prevalence, treatment, and associated disability of mental disorders in four provinces in China during 2001-05: an epidemiological survey. Lancet.

[41] Samet S, Fenton MC, Nunes E, Greenstein E, Aharonovich E, Hasin D (2013). Effects of independent and substance-induced major depressive disorder on remission and relapse of alcohol, cocaine and heroin dependence. Addiction.

[42] Schuckit MA (2006). Drug and alcohol Abuse: a clinical guide to diagnosis and treatment.

[43] Salo R, Flower K, Kielstein A, Leamon MH, Nordahl TE, Galloway GP (2011). Psychiatric comorbidity in methamphetamine dependence. Psychiatry Res.

[44] Schuckit MA (2006). Comorbidity between substance use disorders and psychiatric conditions. Addiction.

[45] Tang YL, Hao W (2007). Improving drug addiction treatment in China. Addiction.

[46] Tate SR, Brown SA, Unrod M, Ramo DE (2004). Context of relapse for substance-dependent adults with and without comorbid psychiatric disorders. Addic Behav.

[47] Tettey J, Wong YL, Levissianos S, Eichinger N, Soe TN, Kelley S, Pahnichaputt P, Umapornsakula A. Patterns and Trends of Amphetamine-Type Stimulants (ATS) and Other Drugs - Challenges for Asia and the Pacific. United Nations Office on Drugs and Crime. 2013. https://www.unodc.org/documents/southeastasiaandpacific//Publications/2013/ats-2013/2013_Regional_ATS_Report_web.pdf59-61. Accessed Nov 2013.

[48] Ujike H, Sato M (2004). Clinical features of sensitization to methamphetamine observed in patients with methamphetamine dependence and psychosis. Ann N Y Acad Sci.

[49] Yen CF, Chong MY (2006). Comorbid psychiatric disorders, sex, and methamphetamine use in adolescents: a case-control study. Compr Psychiatry.

