# Supplementary material for: Comparison of demographic characteristics and psychiatric comorbidity among methamphetamine-, heroin- and methamphetamine-heroin co- dependent males in Hunan, China
Source: BMC Psychiatry. 2017 May 12;17:183. doi: 10.1186/s12888-017-1346-7 (PMC5427618; doi:10.1186/s12888-017-1346-7)
Supplement: Additional file 1: — Structured Clinical Interview for the DSM-IV Axis I Disorders —Patient Edition 20,170,421. (PDF 915 kb) [file 12888_2017_1346_MOESM1_ESM.pdf]

Structured Clinical Interview for DSM-IV Axis I Disorders

Patient Edition (February 1996 FINAL)
SCID-I/P (Version 2.0)

Overview

INTERVIEW INFORMATION

Status: O In progress O Completed O Consensus reviewed

Type: O Computer O Paper

Subject ID:

Subject Initials:

Rater:

Site:

Date of Interview:

Sources of information (check all that apply):
O Subject
O Family
O Health professional/chart/referral note

Relationship to Proband:

Edited and checked by:

Date:

Recruitment Source:

DEMOGRAPHIC DATA

I'm going to be asking you about problems or difficulties you may have had, and I'll be making some notes as we go along. Do you have any questions before we begin?

Information

Gender: Date of Birth: Age:

What do you consider to be your ethnic origin?

Marital Status

What is your current marital status?

Dates of Marriage

Start Date	End Date	Comments

Children

Do you have any children? O Yes O No

Children

Gender	Age	Comments

Living Situation

With whom do you live?

Religion

What was your childhood religious affiliation, if any?

What is your current religion, if any?

FAMILY HISTORY

Were you adopted? O Yes O No

Mother

Living: O Yes O No

Brief Description (age, current location and living situation, general disposition, etc):

Occupation:

Highest Level of Education:

Religion:

# of Siblings:

Father

Living: O Yes O No

Brief Description (age, current location and living situation, general disposition, etc):

Occupation:

Highest Level of Education:

Religion:

# of Siblings:

Do you have any siblings? O Yes O No

(If yes, note genders and ages. Also indicate half of step siblings.)
Are you close to any of your siblings?

What was it like growing up in your family?
(Briefly describe home environment and relationships, including any trauma or abuse.)

Family History Form

Interviewer: "Tell me about your biological parents, children, siblings and grandparents." Ask if they have had any problems with their mood or anxiety or problems with drugs or alcohol. If adopted, ask about biological family; if not known, indicate "Adoptive Family" and answer accordingly. If deceased, note both date of death and "+" symbol in current age column.

Relation	Name	Current Age	Psychiatric Symptoms	Professional Diagnosis (list)	Psychiatric Treatment	Comments

DEVELOPMENTAL HISTORY

Where were you born and raised?
(Significant moves, health, school, friends, activities, etc.)

EDUCATION

How far did you get in school?

EVER FAILED TO COMPLETE A PROGRAM IN WHICH S/HE WAS ENROLLED: Why didn't you finish?

MILITARY HISTORY

Military Service: O Yes O No

Branch:

End of Service:

Start of Service:

Theater:

Veteran: O Yes O No

Combat: O Yes O No

Type of Discharge:

Rank at Discharge:

MOS:

Service Connected Disability O Yes O No

Percent

Reason

WORK HISTORY

Are you working now? What is your job? How long have you been there?

[IF LESS THAN 6 MONTHS: Why did you leave your last job?]

Have you always done this kind of work? [IF NOT: What kind of work have you done?] What is the highest level job you have ever held? [Chronology of work history: (include longest job held and longest time unemployed)] How are you supporting yourself now? (If disability, list type, date and reason.)

Has there ever been a period of time when you were unable to work or go to school? (When? Why was that?)

OVERVIEW OF PRESENT ILLNESS

Have you been in any kind of treatment in the past month?

[IF CURRENTLY IN TREATMENT:
Date of admission to inpatient or outpatient facility.]

CHIEF COMPLAINT

(Description of presenting problem): [RECORD DIRECT QUOTE]

What led to your coming here? What is the major problem you have been having?

HISTORY OF PRESENT ILLNESS

Do you currently have any psychiatric symptoms or emotional problems? O Yes O No

IF YES: When did your current symptoms begin? When were you last feeling your normal self? Is this something new or a return of something you have had before? What was going on in your life when this began? (Environmental context for precipitants of present illness or exacerbation) Did anything happen or change? Since this began, when have you felt the worst? (IF MORE THAN A YEAR AGO: In the last year, when have you felt the worst?)

Have you had any other problems in the last month? What has your mood been like? How have you been spending your free time? Who do you spend time with?

How much have you been drinking (alcohol) (in the past month)? Have you been taking any drugs (in the past month)? (What about marijuana, cocaine, other street drugs?)

PAST PSYCHIATRIC HISTORY

When in your life did you first experience your symptoms? When was the first time you saw someone for emotional or psychiatric problems? (What was that for? What treatment(s) did you receive? What medications?) Were there other times when you had counseling or treatment of any kind? (What type? When?)

Age of first treatment for Depression

Age of first treatment for Mania

Age of first treatment for Hypomania

Age of first treatment for Mixed State

Age of first treatment for Psychosis/SZ

HOSPITALIZATIONS:

Have you ever been a patient in a psychiatric hospital? O Yes O No

(IF YES: When? Where? Why?)

Number of previous hospitalizations for Depression
(Do not include transfers)

Number of previous hospitalizations for Mania

Number of previous hospitalizations for Mixed State

Number of previous hospitalizations for Non-mood

Estimated lifetime total time of psychiatric hospitalization in weeks:

SUBSTANCE/ALCOHOL TREATMENT:

Have you ever had treatment for drugs or alcohol? O Yes O No

Treatment Information:

ATTENTION DEFICIT-HYPERACTIVITY DISORDER:

Have you ever been diagnosed with Attention Deficit-Hyperactivity Disorder? O Yes O No

(Include symptoms, presentation, age at diagnosis, age of first symptoms and treatment)

Medication Assessment Form

Category: **Class:** **Drug Name:** **Start Date:** **End Date:** O Unknown

Multiple Trials: **Duration Used:** **Reason Stopped:** **Response Type:** **Treatment Induced:**

Comments
[Record side effect information whenever possible.]

MEDICAL HISTORY

Have you had any medical problems now or in the past? (What were they? How were they treated?) Were you ever in the hospital for treatment of a medical problem? (What was that for?) Have you ever had any surgeries (including outpatient)? (When? What were they for?)

O Yes O No

ALLERGIES:
Do you have any allergies? To Medications? Other?
O Yes O No

GENETIC DISORDERS:
Do you have any other genetic disorders? (What and when diagnosed?) Do you know of any genetic disorders that run in your family? (What? Who?)
O Yes O No

THYROID DISORDER:
Have you ever been treated for a thyroid disorder? (Include diagnosis, age of diagnosis, and treatment) Was this only while on Lithium?

O Yes O No

HEAD INJURY:
Have you ever had a head injury? (Did you lose consciousness? How long? How many times have you lost consciousness due to a head injury?)
O Yes O No

FEMALES ONLY:
Have you gone through menopause? (Have you ever had any serious emotional problems associated with menopause?)
O Yes O No

OTHER CURRENT PROBLEMS

MOST LIKELY CURRENT DIAGNOSIS

DIAGNOSES THAT NEED TO BE RULED OUT

GLOBAL ASSESSMENT OF FUNCTIONING

Current GAF

DSM-IV Axis V: Global Assessment of Functioning Scale

Consider psychological, social, and occupational functioning on a hypothetical continuum of mental health-illness. Do not include impairment in functioning due to physical (or environmental) limitations. Indicate appropriate code for the LOWEST level of functioning during the week of POOREST functioning. (Use intermediate level when appropriate, e.g., 45, 58, 72.)

100<br>91	Superior functioning in a wide range of activities, life's problems never seem to get out of hand, is sought out by others because of his or her many positive qualities. No symptoms.
90	Absent or minimal symptoms (e.g., mild anxiety before an exam), good functioning in all areas, interested and involved in a wide range of activities, socially effective, generally satisfied with life, no more than everyday problems or concerns (e.g., an occasional

81	argument with family members).
80<br>71	If symptoms are present, they are transient and expectable reactions to psychosocial stressors (e.g., difficulty concentrating after family argument), no more than slight impairment in social, occupational, or school functioning (e.g., temporarily falling behind In school work).
70<br>61	Some mild symptoms (e.g., depressed mood and mild Insomnia) OR some difficulty in social, occupational, or school functioning (e.g., occasional truancy, or theft within the household), but generally functioning pretty well, has some meaningful interpersonal relationships.
60<br>51	Moderate symptoms (e.g., flat affect and circumstantial speech, occasional panic attacks) OR moderate difficulty in social, occupational, or school functioning (e.g., few friends, conflicts with co-workers).
50<br>41	Serious symptoms (e.g., suicidal ideation, severe obsessional rituals, frequent shoplifting) OR any serious impairment in social, occupational, or school functioning (e.g., no friends, unable to keep a job).
40<br>31	Some impairment in reality testing or communication (e.g., speech is at times illogical, obscure, or irrelevant) OR major impairment in several areas, such as work or school, family relations, judgment, thinking, or mood (e.g., depressed man avoids friends, neglects family, and is unable to work; child frequently beats up younger children, Is defiant at home, and is failing at school).
30<br>21	Behavior is considerably influenced by delusions or hallucinations OR serious impairment in communication or judgment (e.g., sometimes Incoherent, acts grossly inappropriately, suicidal preoccupation) OR inability to function in almost all areas (e.g., stays in bed all day; no job, home, or friends)
20<br>11	Some danger of hurting self or others (e.g., suicide attempts without clear expectation of death, frequently violent, manic excitement) OR occasionally fails to maintain minimal personal hygiene (e.g., smears feces) OR gross impairment in communication (e.g., largely incoherent or mute)
10<br>1	Persistent danger of severely hurting self or others (e.g., recurrent violence) OR persistent inability to maintain minimal personal hygiene OR serious suicide act with clear expectation of death

Module A: Depression

MAJOR AND MINOR DEPRESSIVE EPISODES

Episodes Summary

	Date of Onset	Age	Date of Offset	Duration (days)	
A - CURRENT (LAST MONTH)					O Go There
B - WORST EPISODE					O Go There
C - FIRST EPISODE					O Go There
D - ANOTHER EPISODE					O Go There
E - ANOTHER EPISODE					O Go There

Episode A: Current Depression

Date of Onset	Age	Date of Offset	Duration (days)

Depression Criteria

Question	Criteria	Rating
Now I would like to ask you some more specific questions about (TIME PERIOD FOR SUSPECTED DEPRESSIVE EPISODE).	A. Five or more of the following symptoms have been present during the same two-week period and represent a change from previous functioning; at least one of the symptoms was either (1) depressed mood or (2) loss of interest or pleasure.	
During this time, (TIME PERIOD FOR SUSPECTED DEPRESSIVE EPISODE) were you depressed or down, most of the day nearly every day? (What was that like?)<br><br>IF YES: When was that? How long did it last? As long as two weeks?	(1) depressed mood most of the day, nearly every day, as indicated by either subjective report (e.g., feels sad or empty) or observations made by others (e.g., appears tearful). Note: in children and adolescents, can be irritable mood.	O ? O 1 O 2 O 3
Did you lose interest or pleasure in things you usually enjoyed? (What was that like?)<br><br>IF YES: When was that? Was that nearly every day How long did it last? As long as two weeks?	(2) markedly diminished interest or pleasure in all, or almost all, activities most of the day, nearly every day (as indicated either by subjective account or observation made by others)	O ? O 1 O 2 O 3

FOR ALL SUBJECTS, CONTINUE ASKING ABOUT ALL SYMPTOMS EVEN IF A(1) AND/OR (2) ARE NOT ENDORSED.

NOTE: WHEN RATING THE FOLLOWING ITEMS, CODE "1" IF CLEARLY DUE TO A GENERAL MEDICAL CONDITION, SUBSTANCE, OR TO MOOD-INCONGRUENT DELUSIONS OR HALLUCINATIONS. TO COUNT TOWARD A MAJOR DEPRESSIVE EPISODE, A SYMPTOM MUST EITHER BE NEWLY PRESENT OR MUST HAVE CLEARLY WORSENED COMPARED WITH THE PERSON'S PRE-EPISODE STATUS

I would like you to focus on the worst two week period when answering the following questions. During (TIME PERIOD OF EPISODE)

FOCUS ON WORST TWO WEEK PERIOD OF EPISODE TO DETERMINE IF FULL MAJOR DEPRESSIVE EPISODE CRITERIA ARE MET

Question	Criteria	Rating
...did you lose or gain any weight? (How much? Were you trying to lose weight?)<br>IF NO: How was your appetite? What about compared to your usual appetite? Did you have to force yourself to eat? Eat (less/more) than usual? Was that nearly every day?	(3) significant weight loss when not dieting, or weight gain (e.g., a change of more than 5% of body weight in a month), or decrease or increase in appetite nearly every day. Note: in children, consider failure to make expected weight gains.<br><br>Check if:<br>weight loss or decreased appetite O<br>weight gain or increased appetite O	O ? O 1 O 2 O 3
...how were you sleeping? (Trouble falling asleep, waking frequently, trouble staying asleep, waking too early, OR sleeping too much? How many hours a night compared to usual? Was that nearly every night?)	(4) insomnia or hypersomnia nearly every day<br><br>Check if:	O ? O 1 O 2 O 3

insomnia O

hypersomnia O

...were you so fidgety or restless that you were unable to sit still? (Was it so bad that other people noticed it? What did they notice? Was that nearly every day?)<br>IF NO: What about the opposite-talking more slowly than is normal for you? Was it so bad that other people noticed it? What did they notice? Was it nearly every day?	(5) psychomotor agitation or retardation nearly every day (observable by others, not merely subjective feelings of restlessness or being slowed down)	O O O O<br>? 1 2 3

Check if:

psychomotor agitation O

psychomotor retardation O

...what was your energy like? (tired all the time? Nearly every day?)	(6) fatigue or loss of energy nearly every day	O O O O<br>? 1 2 3

...how did you feel about yourself? (Worthless? Nearly every day?)<br>IF NO: What about feeling guilty about things you had done or not done? Nearly every day?	(7) feelings of worthlessness or excessive or inappropriate guilt (which may be delusional) nearly every day (not merely self-reproach or guilt about being sick)	O O O O<br>? 1 2 3

Check if:

feelings of worthlessness O

excessive or inappropriate guilt O

...did you have trouble thinking or concentrating? (What kinds of things did it interfere with? Nearly every day?)<br>IF NO: Was it hard to make decisions about everyday things? Nearly every day?	(8) diminished ability to think or concentrate, or indecisiveness, nearly every day (either by subjective account or as observed by others)	O O O O<br>? 1 2 3

Check if:

diminished ability to think O

indecisiveness O

Were things so bad you were thinking a lot about death or that you would be better off dead? What about thinking of hurting yourself?<br>IF YES: Did you do anything to hurt yourself?	(9) recurrent thoughts of death (not just fear of dying), recurrent suicidal ideation without a specific plan, or a suicide attempt or a specific plan for committing suicide	O O O O<br>? 1 2 3

Check if:

thoughts of own death O

suicidal ideation O

specific plan O

actual attempt O

	NUMBER OF SYMPTOMS A(1) - A(9) CODED "3"	0
	**Major Depressive Episode**	
	AT LEAST FIVE OF A(1) - A(9) ARE CODED "3" AND EITHER A(1) OR A(2) ARE CODED "3"	false
	**Minor Depressive Episode**	
	EITHER TWO,THREE, OR FOUR OF A(1) - A(9) ARE CODED "3" AND EITHER A(1) OR A(2) ARE CODED "3"	false
	**SUICIDALITY IN DEPRESSION**	
	FOLLOWING EPISODE A, ASK THE THREE QUESTIONS BELOW REGARDING SUICIDALITY, THEN CONTINUE ON PAGE A5 WITH REMAINDER OF EPISODE A. FOR EPISODES B-E, SKIP THIS SECTION AND GO TO NEXT PAGE (A5).	
IF UNKNOWN: Have you ever attempted suicide during a depressive episode?	Has made a suicide attempt	O O<br>1 3
IF YES: How many times?	Lifetime total number of suicide attempts during depression	
Do you think about suicide during most of your depressive episodes?	Determine whether suicidal ideation is present during most depressive episodes	O O O O<br>? 1 2 3

IF UNCLEAR: Did (DEPRESSIVE EPISODE/OWN EQUIVALENT) make it hard for you to do your work, take care of things at home, or get along with other people?<br><br>IF YES, SPECIFY:	B. The symptoms cause clinically significant distress or impairment in social, occupational, or other important areas of functioning.<br>NOTE: FOR SOME INDIVIDUALS WITH MILDER EPISODES, FUNCTIONING MAY APPEAR TO BE NORMAL BUT REQUIRES MARKEDLY INCREASED EFFORT.	O O O O<br>? 1 2 3

Just before this began, were you physically ill?<br><br>Just before this began, were you drinking or taking any street drugs?<br><br>IF YES: Any change in the amount you were taking?<br><br>Just before this began, were you taking any medications?<br><br>IF YES: Any change in the amount you were taking?	C. Not due to the direct physiological effects of a substance (e.g., a drug of abuse, medication) or to a general medical condition (e.g., hypothyroidism)<br><br>IF GENERAL MEDICAL CONDITION OR SUBSTANCE MAY BE ETIOLOGICALLY ASSOCIATED WITH DEPRESSION, GO TO *GMC/SUBSTANCE* A.51, AND RETURN HERE TO MAKE RATING OF "1" OR "3."	O O O<br>? 1 3

IF THE EPISODE WAS PRECIPITATED BY MEDICATION TREATMENT, RECORD DETAILED INFORMATION ON THE MEDICATION ASSESSMENT FORM.

Did this begin soon after someone close to you died?	D. Not better accounted for by Bereavement, i.e., after the loss of a loved one, the symptoms persist for longer than 2 months or are characterized by marked functional impairment, morbid preoccupation with worthlessness, suicidal ideation, psychotic symptoms or psychomotor retardation.	O 1 O 3

Major Depressive Episode

MAJOR DEPRESSIVE EPISODE CRITERIA A, B, C, AND D ARE CODED "3" — false

Minor Depressive Episode

MINOR DEPRESSIVE EPISODE CRITERIA A, B, C, AND D ARE CODED "3" — false

FIRST AND WORST DEPRESSION

IF UNCLEAR: Is this your worst episode of depression?	**Worst**<br>DETERMINE WHETHER OR NOT EACH EPISODE IS THE FIRST OR THE WORST DEPRESSION. CODE "3" FOR ONLY ONE FIRST AND ONE WORST EPISODE. THEY MAY BE THE SAME EPISODE AND MAY NOT ALWAYS BE CODED IN B OR C (I.E., D OR E MAY ACTUALLY BE EARLIER OR MORE SEVERE ONCE RECALLED).	O 1 O 3
Is this the first episode?	**First**	O 1 O 3

MIXED STATE

ASK THE FOLLOWING QUESTIONS TO DETERMINE WHETHER A MIXED STATE WAS PRESENT FOR EACH EPISODE OF DEPRESSION.

During this episode of depression did you have a week or more during which your mood changed between sadness and irritability or even elation?	O ? O 1 O 2 O 3

IF YES, CHECK IF:

Irritability O

Elation O

During this episode of depression did you also experience any of the following symptoms?

Over activity, such as running around, having many projects, or feeling physically agitated?	O ?	O 1	O 2	O 3
More talkative than usual or feeling that your speech was pressured?	O ?	O 1	O 2	O 3
Thoughts racing or jumping from topic to topic?	O ?	O 1	O 2	O 3
Feeling grandiose, more important, special, or powerful?	O ?	O 1	O 2	O 3
Needing less sleep or feeling energetic after little or no sleep?	O ?	O 1	O 2	O 3
Attention distracted by unimportant things?	O ?	O 1	O 2	O 3
Doing risky things for pleasure like excessive spending, reckless driving, sexual indiscretions, etc?	O ?	O 1	O 2	O 3

	NUMBER OF "3" RESPONSES FROM MIXED STATE SECTION.	0
How long were these symptoms present?	ENTER NUMBER OF DAYS	
	CRITERIA WERE MET SIMULTANEOUSLY FOR BOTH MAJOR DEPRESSION AND MANIA. IRRITABLE MOOD PLUS FOUR SYMPTOMS, OR ELATED MOOD PLUS THREE SYMPTOMS	false
	IRRITABLE MOOD PLUS 2-3 SYMPTOMS OR ELATED MOOD PLUS 2 SYMPTOMS	false

Were your mood symptoms predominantly irritable, sad (dysphoria) or elated (euphoria)?

Predominance of:

Irritability O

Dysphoria O

Euphoria O

[PROBE IN THE SAME WAY FOR EACH CODED EPISODE]

During this episode of depression, did you have any beliefs or ideas that you later found out were not true? (Like believing that you had powers and abilities others did not have? Or that you had a special mission, perhaps from God? Or that someone was trying to harm you? How certain were you?)

Did you see or hear things other people could not see or hear?

Probe for Psychotic Symptoms per Episode:

IF DELUSIONS OR HALLUCINATIONS ARE SUSPECTED, PROBE FURTHER TO DETERMINE THE CONTENT AND WHETHER THE BELIEFS WERE HELD WITH CERTAINTY.

O ? O 1 O 3

IF YES, PLEASE CHECK:

O Delusions

O Hallucinations

IF YES, DESCRIBE:

End of Episode-Specific Questions. Will Another Episode Be Coded? O Yes O No

Episode B: Worst Depression

Date of Onset	Age	Date of Offset	Duration (days)

Depression Criteria

Now I would like to ask you some more specific questions about (TIME PERIOD FOR SUSPECTED DEPRESSIVE EPISODE).

A. Five or more of the following symptoms have been present during the same two-week period and represent a change from previous functioning; at least one of the symptoms was either (1) depressed mood or (2) loss of interest or pleasure.

During this time, (TIME PERIOD FOR SUSPECTED DEPRESSIVE EPISODE) were you depressed or down, most of the day nearly every day? (What was that like?)

IF YES: When was that? How long did it last? As long as two weeks?

(1) depressed mood most of the day, nearly every day, as indicated by either subjective report (e.g., feels sad or empty) or observations made by others (e.g., appears tearful). Note: in children and adolescents, can be irritable mood.

O ? O 1 O 2 O 3

Did you lose interest or pleasure in things you usually enjoyed? (What was that like?)

IF YES: When was that? Was that nearly every day How long did it last? As long as two weeks?

(2) markedly diminished interest or pleasure in all, or almost all, activities most of the day, nearly every day (as indicated either by subjective account or observation made by others)

O ? O 1 O 2 O 3

FOR ALL SUBJECTS, CONTINUE ASKING ABOUT ALL SYMPTOMS EVEN IF A(1) AND/OR (2) ARE NOT ENDORSED.

NOTE: WHEN RATING THE FOLLOWING ITEMS, CODE "1" IF CLEARLY DUE TO A GENERAL MEDICAL CONDITION, SUBSTANCE, OR TO MOOD-INCONGRUENT DELUSIONS OR HALLUCINATIONS. TO COUNT TOWARD A MAJOR DEPRESSIVE EPISODE, A SYMPTOM MUST EITHER BE NEWLY PRESENT OR MUST HAVE CLEARLY WORSENED COMPARED WITH THE PERSON'S PRE-EPISODE STATUS

I would like you to focus on the worst two week period when answering the following questions. During (TIME PERIOD OF EPISODE)

FOCUS ON WORST TWO WEEK PERIOD OF EPISODE TO DETERMINE IF FULL MAJOR DEPRESSIVE EPISODE CRITERIA ARE MET

...did you lose or gain any weight? (How much? Were you trying to lose weight?)<br>IF NO: How was your appetite? What about compared to your usual appetite? Did you have to force yourself to eat? Eat (less/more) than usual? Was that nearly every day?	(3) significant weight loss when not dieting, or weight gain (e.g., a change of more than 5% of body weight in a month), or decrease or increase in appetite nearly every day. Note: in children, consider failure to make expected weight gains.<br><br>Check if:<br>weight loss or decreased appetite O<br>weight gain or increased appetite O	O O O O<br>? 1 2 3
...how were you sleeping? (Trouble falling asleep, waking frequently, trouble staying asleep, waking too early, OR sleeping too much? How many hours a night compared to usual? Was that nearly every night?)	(4) insomnia or hypersomnia nearly every day<br><br>Check if:<br>insomnia O<br>hypersomnia O	O O O O<br>? 1 2 3
...were you so fidgety or restless that you were unable to sit still? (Was it so bad that other people noticed it? What did they notice? Was that nearly every day?)<br>IF NO: What about the opposite-talking more slowly than is normal for you? Was it so bad that other people noticed it? What did they notice? Was it nearly every day?	(5) psychomotor agitation or retardation nearly every day (observable by others, not merely subjective feelings of restlessness or being slowed down)<br><br>Check if:<br>psychomotor agitation O<br>psychomotor retardation O	O O O O<br>? 1 2 3
...what was your energy like? (tired all the time? Nearly every day?)	(6) fatigue or loss of energy nearly every day	O O O O<br>? 1 2 3
...how did you feel about yourself? (Worthless? Nearly every day?)<br>IF NO: What about feeling guilty about things you had done or not done? Nearly every day?	(7) feelings of worthlessness or excessive or inappropriate guilt (which may be delusional) nearly every day (not merely self-reproach or guilt about being sick)<br><br>Check if:<br>feelings of worthlessness O	O O O O<br>? 1 2 3

excessive or inappropriate guilt O

...did you have trouble thinking or concentrating? (What kinds of things did it interfere with? Nearly every day?)<br>IF NO: Was it hard to make decisions about everyday things? Nearly every day?	(8) diminished ability to think or concentrate, or indecisiveness, nearly every day (either by subjective account or as observed by others)	O O O O<br>? 1 2 3

Check if:

diminished ability to think O

indecisiveness O

Were things so bad you were thinking a lot about death or that you would be better off dead? What about thinking of hurting yourself?<br>IF YES: Did you do anything to hurt yourself?	(9) recurrent thoughts of death (not just fear of dying), recurrent suicidal ideation without a specific plan, or a suicide attempt or a specific plan for committing suicide	O O O O<br>? 1 2 3

Check if:

thoughts of own death O

suicidal ideation O

specific plan O

actual attempt O

	NUMBER OF SYMPTOMS A(1) - A(9) CODED "3"	0
	**Major Depressive Episode**<br>AT LEAST FIVE OF A(1) - A(9) ARE CODED "3" AND EITHER A(1) OR A(2) ARE CODED "3"	false
	**Minor Depressive Episode**<br>EITHER TWO,THREE, OR FOUR OF A(1) - A(9) ARE CODED "3" AND EITHER A(1) OR A(2) ARE CODED "3"	false
IF UNCLEAR: Did (DEPRESSIVE EPISODE/OWN EQUIVALENT) make it hard for you to do your work, take care of things at home, or get along with other people?<br><br>IF YES, SPECIFY:	B. The symptoms cause clinically significant distress or impairment in social, occupational, or other important areas of functioning.<br>NOTE: FOR SOME INDIVIDUALS WITH MILDER EPISODES, FUNCTIONING MAY APPEAR TO BE NORMAL BUT REQUIRES MARKEDLY INCREASED EFFORT.	O O O O<br>? 1 2 3

Just before this began, were you physically ill?<br><br>Just before this began, were you drinking or taking any street drugs?<br><br>IF YES: Any change in the amount you were taking?	C. Not due to the direct physiological effects of a substance (e.g., a drug of abuse, medication) or to a general medical condition (e.g., hypothyroidism)<br><br>IF GENERAL MEDICAL CONDITION OR SUBSTANCE MAY BE ETIOLOGICALLY ASSOCIATED WITH DEPRESSION, GO TO	O O O<br>? 1 3

Just before this began, were you taking any medications?<br><br>IF YES: Any change in the amount you were taking?	*GMC/SUBSTANCE* A.51, AND RETURN HERE TO MAKE RATING OF "1" OR "3."<br><br>IF THE EPISODE WAS PRECIPITATED BY MEDICATION TREATMENT, RECORD DETAILED INFORMATION ON THE MEDICATION ASSESSMENT FORM.	
Did this begin soon after someone close to you died?	D. Not better accounted for by Bereavement, i.e., after the loss of a loved one, the symptoms persist for longer than 2 months or are characterized by marked functional impairment, morbid preoccupation with worthlessness, suicidal ideation, psychotic symptoms or psychomotor retardation.	O O<br>1 3
	**Major Depressive Episode**<br>MAJOR DEPRESSIVE EPISODE CRITERIA A, B, C, AND D ARE CODED "3"	false
	**Minor Depressive Episode**<br>MINOR DEPRESSIVE EPISODE CRITERIA A, B, C, AND D ARE CODED "3"	false
	**FIRST AND WORST DEPRESSION**	
IF UNCLEAR: Is this your worst episode of depression?	**Worst**<br>DETERMINE WHETHER OR NOT EACH EPISODE IS THE FIRST OR THE WORST DEPRESSION. CODE "3" FOR ONLY ONE FIRST AND ONE WORST EPISODE. THEY MAY BE THE SAME EPISODE AND MAY NOT ALWAYS BE CODED IN B OR C (I.E., D OR E MAY ACTUALLY BE EARLIER OR MORE SEVERE ONCE RECALLED).	O O<br>1 3
Is this the first episode?	**First**	O O<br>1 3
	**MIXED STATE**<br>ASK THE FOLLOWING QUESTIONS TO DETERMINE WHETHER A MIXED STATE WAS PRESENT FOR EACH EPISODE OF DEPRESSION.	
During this episode of depression did you have a week or more during which your mood changed		O O O O<br>? 1 2 3

between sadness and irritability or even elation?

IF YES, CHECK IF:
Irritability O

Elation O

During this episode of depression did you also experience any of the following symptoms?

	O	O	O	O
Over activity, such as running around, having many projects, or feeling physically agitated?	?	1	2	3
More talkative than usual or feeling that your speech was pressured?	?	1	2	3
Thoughts racing or jumping from topic to topic?	?	1	2	3
Feeling grandiose, more important, special, or powerful?	?	1	2	3
Needing less sleep or feeling energetic after little or no sleep?	?	1	2	3
Attention distracted by unimportant things?	?	1	2	3
Doing risky things for pleasure like excessive spending, reckless driving, sexual indiscretions, etc?	?	1	2	3

NUMBER OF "3" RESPONSES FROM MIXED STATE SECTION. 0

How long were these symptoms present? | ENTER NUMBER OF DAYS

CRITERIA WERE MET SIMULTANEOUSLY FOR BOTH MAJOR DEPRESSION AND MANIA. IRRITABLE MOOD PLUS FOUR SYMPTOMS, OR ELATED MOOD PLUS THREE SYMPTOMS — false

IRRITABLE MOOD PLUS 2-3 SYMPTOMS OR ELATED MOOD PLUS 2 SYMPTOMS — false

Were your mood symptoms predominantly irritable, sad (dysphoria) or elated (euphoria)?

Predominance of:

Irritability O

Dysphoria O

Euphoria O

[PROBE IN THE SAME WAY FOR EACH CODED EPISODE]

During this episode of depression, did you have any beliefs or ideas that you later found out were not true? (Like believing that you had powers and abilities others did not have? Or that you had a special mission, perhaps from God? Or that someone was trying to harm you? How certain were you?)

Did you see or hear things other people could not see or hear?

Probe for Psychotic Symptoms per Episode:

IF DELUSIONS OR HALLUCINATIONS ARE SUSPECTED, PROBE FURTHER TO DETERMINE THE CONTENT AND WHETHER THE BELIEFS WERE HELD WITH CERTAINTY.

O ? O 1 O 3

IF YES, PLEASE CHECK:

O Delusions

O Hallucinations

IF YES, DESCRIBE:

End of Episode-Specific Questions. Will Another Episode Be Coded? O Yes O No

Episode C: First Depression

Date of Onset	Age	Date of Offset	Duration (days)

Depression Criteria

Now I would like to ask you some more specific questions about (TIME PERIOD FOR SUSPECTED DEPRESSIVE EPISODE).

A. Five or more of the following symptoms have been present during the same two-week period and represent a change from previous functioning; at least one of the symptoms was either (1) depressed mood or (2) loss of interest or pleasure.

During this time, (TIME PERIOD FOR SUSPECTED DEPRESSIVE EPISODE) were you depressed or down, most of the day nearly every day? (What was that like?)

IF YES: When was that? How long did it last? As long as two weeks?

(1) depressed mood most of the day, nearly every day, as indicated by either subjective report (e.g., feels sad or empty) or observations made by others (e.g., appears tearful). Note: in children and adolescents, can be irritable mood.

O ? O 1 O 2 O 3

Did you lose interest or pleasure in things you usually enjoyed? (What was that like?)<br><br>IF YES: When was that? Was that nearly every day How long did it last? As long as two weeks?	(2) markedly diminished interest or pleasure in all, or almost all, activities most of the day, nearly every day (as indicated either by subjective account or observation made by others)	O O O O<br>? 1 2 3

FOR ALL SUBJECTS, CONTINUE ASKING ABOUT ALL SYMPTOMS EVEN IF A(1) AND/OR (2) ARE NOT ENDORSED.

NOTE: WHEN RATING THE FOLLOWING ITEMS, CODE "1" IF CLEARLY DUE TO A GENERAL MEDICAL CONDITION, SUBSTANCE, OR TO MOOD-INCONGRUENT DELUSIONS OR HALLUCINATIONS. TO COUNT TOWARD A MAJOR DEPRESSIVE EPISODE, A SYMPTOM MUST EITHER BE NEWLY PRESENT OR MUST HAVE CLEARLY WORSENED COMPARED WITH THE PERSON'S PRE-EPISODE STATUS

I would like you to focus on the worst two week period when answering the following questions. During (TIME PERIOD OF EPISODE)

FOCUS ON WORST TWO WEEK PERIOD OF EPISODE TO DETERMINE IF FULL MAJOR DEPRESSIVE EPISODE CRITERIA ARE MET

...did you lose or gain any weight? (How much? Were you trying to lose weight?)<br>IF NO: How was your appetite? What about compared to your usual appetite? Did you have to force yourself to eat? Eat (less/more) than usual? Was that nearly every day?	(3) significant weight loss when not dieting, or weight gain (e.g., a change of more than 5% of body weight in a month), or decrease or increase in appetite nearly every day. Note: in children, consider failure to make expected weight gains.	O O O O<br>? 1 2 3

Check if:

weight loss or decreased appetite O

weight gain or increased appetite O

...how were you sleeping? (Trouble falling asleep, waking frequently, trouble staying asleep, waking too early, OR sleeping too much? How many hours a night compared to usual? Was that nearly every night?)	(4) insomnia or hypersomnia nearly every day	O O O O<br>? 1 2 3

Check if:

insomnia O

hypersomnia O

...were you so fidgety or restless that you were unable to sit still? (Was it so bad that other people noticed it? What did they notice? Was that nearly every day?)<br>IF NO: What about the opposite-talking more slowly than is normal for you? Was it so bad that other people noticed it? What did they notice? Was it nearly every day?	(5) psychomotor agitation or retardation nearly every day (observable by others, not merely subjective feelings of restlessness or being slowed down)	O O O O<br>? 1 2 3

Check if:

psychomotor agitation O

psychomotor retardation O

...what was your energy like? (tired all the time? Nearly every day?)	(6) fatigue or loss of energy nearly every day	O O O O<br>? 1 2 3
...how did you feel about yourself? (Worthless? Nearly every day?)<br>IF NO: What about feeling guilty about things you had done or not done? Nearly every day?	(7) feelings of worthlessness or excessive or inappropriate guilt (which may be delusional) nearly every day (not merely self-reproach or guilt about being sick)<br><br>Check if:<br>feelings of worthlessness O<br>excessive or inappropriate guilt O	O O O O<br>? 1 2 3
...did you have trouble thinking or concentrating? (What kinds of things did it interfere with? Nearly every day?)<br>IF NO: Was it hard to make decisions about everyday things? Nearly every day?	(8) diminished ability to think or concentrate, or indecisiveness, nearly every day (either by subjective account or as observed by others)<br><br>Check if:<br>diminished ability to think O<br>indecisiveness O	O O O O<br>? 1 2 3
Were things so bad you were thinking a lot about death or that you would be better off dead? What about thinking of hurting yourself?<br>IF YES: Did you do anything to hurt yourself?	(9) recurrent thoughts of death (not just fear of dying), recurrent suicidal ideation without a specific plan, or a suicide attempt or a specific plan for committing suicide<br><br>Check if:<br>thoughts of own death O<br>suicidal ideation O<br>specific plan O<br>actual attempt O	O O O O<br>? 1 2 3
	NUMBER OF SYMPTOMS A(1) - A(9) CODED "3"	0
	**Major Depressive Episode**<br>AT LEAST FIVE OF A(1) - A(9) ARE CODED "3" AND EITHER A(1) OR A(2) ARE CODED "3"	false
	**Minor Depressive Episode**<br>EITHER TWO,THREE, OR FOUR OF A(1) - A(9) ARE CODED "3" AND EITHER A(1) OR A(2) ARE CODED "3"	false
IF UNCLEAR: Did (DEPRESSIVE EPISODE/OWN EQUIVALENT) make it hard for you to do your work, take care of things at home, or get along with other people?	B. The symptoms cause clinically significant distress or impairment in social, occupational, or other important areas of functioning.<br>NOTE: FOR SOME INDIVIDUALS WITH MILDER EPISODES, FUNCTIONING MAY APPEAR TO	O O O O<br>? 1 2 3

IF YES, SPECIFY:	BE NORMAL BUT REQUIRES MARKEDLY INCREASED EFFORT.	

Just before this began, were you physically ill?<br><br>Just before this began, were you drinking or taking any street drugs?<br><br>IF YES: Any change in the amount you were taking?<br><br>Just before this began, were you taking any medications?<br><br>IF YES: Any change in the amount you were taking?	C. Not due to the direct physiological effects of a substance (e.g., a drug of abuse, medication) or to a general medical condition (e.g., hypothyroidism)<br><br>IF GENERAL MEDICAL CONDITION OR SUBSTANCE MAY BE ETIOLOGICALLY ASSOCIATED WITH DEPRESSION, GO TO *GMC/SUBSTANCE* A.51, AND RETURN HERE TO MAKE RATING OF "1" OR "3."<br><br>IF THE EPISODE WAS PRECIPITATED BY MEDICATION TREATMENT, RECORD DETAILED INFORMATION ON THE MEDICATION ASSESSMENT FORM.	O O O<br>? 1 3

Did this begin soon after someone close to you died?	D. Not better accounted for by Bereavement, i.e., after the loss of a loved one, the symptoms persist for longer than 2 months or are characterized by marked functional impairment, morbid preoccupation with worthlessness, suicidal ideation, psychotic symptoms or psychomotor retardation.	O O<br>1 3

	**Major Depressive Episode**	
	MAJOR DEPRESSIVE EPISODE CRITERIA A, B, C, AND D ARE CODED "3"	false
	**Minor Depressive Episode**	
	MINOR DEPRESSIVE EPISODE CRITERIA A, B, C, AND D ARE CODED "3"	false
	**FIRST AND WORST DEPRESSION**	
IF UNCLEAR: Is this your worst episode of depression?	**Worst**<br><br>DETERMINE WHETHER OR NOT EACH EPISODE IS THE FIRST OR THE WORST DEPRESSION. CODE "3" FOR ONLY ONE FIRST AND ONE WORST EPISODE. THEY MAY BE THE SAME EPISODE AND MAY NOT ALWAYS BE CODED IN B OR C (I.E., D OR E MAY ACTUALLY BE EARLIER OR MORE SEVERE ONCE RECALLED).	O O<br>1 3

Is this the first episode? **First**

O O
1 3

MIXED STATE

ASK THE FOLLOWING QUESTIONS TO DETERMINE WHETHER A MIXED STATE WAS PRESENT FOR EACH EPISODE OF DEPRESSION.

During this episode of depression did you have a week or more during which your mood changed between sadness and irritability or even elation?

O O O O
? 1 2 3

IF YES, CHECK IF:
Irritability O

Elation O

During this episode of depression did you also experience any of the following symptoms?

Over activity, such as running around, having many projects, or feeling physically agitated?

O O O O
? 1 2 3

More talkative than usual or feeling that your speech was pressured?

O O O O
? 1 2 3

Thoughts racing or jumping from topic to topic?

O O O O
? 1 2 3

Feeling grandiose, more important, special, or powerful?

O O O O
? 1 2 3

Needing less sleep or feeling energetic after little or no sleep?

O O O O
? 1 2 3

Attention distracted by unimportant things?

O O O O
? 1 2 3

Doing risky things for pleasure like excessive spending, reckless driving, sexual indiscretions, etc?

O O O O
? 1 2 3

NUMBER OF "3" RESPONSES FROM MIXED STATE SECTION. 0

How long were these symptoms present? ENTER NUMBER OF DAYS

CRITERIA WERE MET SIMULTANEOUSLY FOR BOTH MAJOR DEPRESSION AND MANIA. IRRITABLE MOOD PLUS FOUR SYMPTOMS, OR ELATED MOOD PLUS THREE SYMPTOMS false

IRRITABLE MOOD PLUS 2-3 SYMPTOMS OR ELATED MOOD PLUS 2 SYMPTOMS false

Were your mood symptoms predominantly irritable, sad (dysphoria) or elated (euphoria)?

Predominance of:

Irritability O

Dysphoria O

Euphoria O

[PROBE IN THE SAME WAY FOR EACH CODED EPISODE]

During this episode of depression, did you have any beliefs or ideas that you later found out were not true? (Like believing that you had powers and abilities others did not have? Or that you had a special mission, perhaps from God? Or that someone was trying to harm you? How certain were you?)

Did you see or hear things other people could not see or hear?

Probe for Psychotic Symptoms per Episode:

IF DELUSIONS OR HALLUCINATIONS ARE SUSPECTED, PROBE FURTHER TO DETERMINE THE CONTENT AND WHETHER THE BELIEFS WERE HELD WITH CERTAINTY.

O	O	O
?	1	3

IF YES, PLEASE CHECK:

O Delusions

O Hallucinations

IF YES, DESCRIBE:

End of Episode-Specific Questions. Will Another Episode Be Coded? O Yes O No

Episode D: Another Depression

Date of Onset	Age	Date of Offset	Duration (days)

Now I would like to ask you some more specific questions about (TIME PERIOD FOR SUSPECTED DEPRESSIVE EPISODE).

Depression Criteria

A. Five or more of the following symptoms have been present during the same two-week period and represent a change from previous functioning; at least one of the symptoms was either (1) depressed mood or (2) loss of interest or pleasure.

During this time, (TIME PERIOD FOR SUSPECTED DEPRESSIVE EPISODE) were you depressed or down, most of the day nearly every day? (What was

(1) depressed mood most of the day, nearly every day, as indicated by either subjective report (e.g., feels sad or empty) or observations made by

O	O	O	O
?	1	2	3

that like?)<br><br>IF YES: When was that? How long did it last? As long as two weeks?	others (e.g., appears tearful). Note: in children and adolescents, can be irritable mood.	

Did you lose interest or pleasure in things you usually enjoyed? (What was that like?)<br><br>IF YES: When was that? Was that nearly every day How long did it last? As long as two weeks?	(2) markedly diminished interest or pleasure in all, or almost all, activities most of the day, nearly every day (as indicated either by subjective account or observation made by others)	O O O O<br>? 1 2 3

FOR ALL SUBJECTS, CONTINUE ASKING ABOUT ALL SYMPTOMS EVEN IF A(1) AND/OR (2) ARE NOT ENDORSED.

NOTE: WHEN RATING THE FOLLOWING ITEMS, CODE "1" IF CLEARLY DUE TO A GENERAL MEDICAL CONDITION, SUBSTANCE, OR TO MOOD-INCONGRUENT DELUSIONS OR HALLUCINATIONS. TO COUNT TOWARD A MAJOR DEPRESSIVE EPISODE, A SYMPTOM MUST EITHER BE NEWLY PRESENT OR MUST HAVE CLEARLY WORSENED COMPARED WITH THE PERSON'S PRE-EPISODE STATUS

I would like you to focus on the worst two week period when answering the following questions. During (TIME PERIOD OF EPISODE)

FOCUS ON WORST TWO WEEK PERIOD OF EPISODE TO DETERMINE IF FULL MAJOR DEPRESSIVE EPISODE CRITERIA ARE MET

...did you lose or gain any weight? (How much? Were you trying to lose weight?)<br>IF NO: How was your appetite? What about compared to your usual appetite? Did you have to force yourself to eat? Eat (less/more) than usual? Was that nearly every day?	(3) significant weight loss when not dieting, or weight gain (e.g., a change of more than 5% of body weight in a month), or decrease or increase in appetite nearly every day. Note: in children, consider failure to make expected weight gains.	O O O O<br>? 1 2 3

Check if:

weight loss or decreased appetite O

weight gain or increased appetite O

...how were you sleeping? (Trouble falling asleep, waking frequently, trouble staying asleep, waking too early, OR sleeping too much? How many hours a night compared to usual? Was that nearly every night?)	(4) insomnia or hypersomnia nearly every day	O O O O<br>? 1 2 3

Check if:

insomnia O

hypersomnia O

...were you so fidgety or restless that you were unable to sit still? (Was it so bad that other people noticed it? What did they notice? Was that nearly every day?)<br>IF NO: What about the opposite-talking more	(5) psychomotor agitation or retardation nearly every day (observable by others, not merely subjective feelings of restlessness or being slowed down)	O O O O<br>? 1 2 3

slowly than is normal for you? Was it so bad that other people noticed it? What did they notice? Was it nearly every day?

Check if:

psychomotor agitation O

psychomotor retardation O

...what was your energy like? (tired all the time? Nearly every day?)	(6) fatigue or loss of energy nearly every day	O ? O 1 O 2 O 3

...how did you feel about yourself? (Worthless? Nearly every day?) IF NO: What about feeling guilty about things you had done or not done? Nearly every day?	(7) feelings of worthlessness or excessive or inappropriate guilt (which may be delusional) nearly every day (not merely self-reproach or guilt about being sick)	O ? O 1 O 2 O 3

Check if:

feelings of worthlessness O

excessive or inappropriate guilt O

...did you have trouble thinking or concentrating? (What kinds of things did it interfere with? Nearly every day?) IF NO: Was it hard to make decisions about everyday things? Nearly every day?	(8) diminished ability to think or concentrate, or indecisiveness, nearly every day (either by subjective account or as observed by others)	O ? O 1 O 2 O 3

Check if:

diminished ability to think O

indecisiveness O

Were things so bad you were thinking a lot about death or that you would be better off dead? What about thinking of hurting yourself? IF YES: Did you do anything to hurt yourself?	(9) recurrent thoughts of death (not just fear of dying), recurrent suicidal ideation without a specific plan, or a suicide attempt or a specific plan for committing suicide	O ? O 1 O 2 O 3

Check if:

thoughts of own death O

suicidal ideation O

specific plan O

actual attempt O

	NUMBER OF SYMPTOMS A(1) - A(9) CODED "3"	0
	**Major Depressive Episode**	
	AT LEAST FIVE OF A(1) - A(9) ARE CODED "3" AND EITHER A(1) OR A(2) ARE CODED "3"	false
	**Minor Depressive Episode**	
	EITHER TWO,THREE, OR FOUR OF A(1) - A(9) ARE CODED "3" AND EITHER A(1) OR A(2) ARE CODED "3"	false
IF UNCLEAR: Did (DEPRESSIVE EPISODE/OWN EQUIVALENT) make it hard for you to do your work, take care of things at home, or get along with other people?<br><br>IF YES, SPECIFY:	B. The symptoms cause clinically significant distress or impairment in social, occupational, or other important areas of functioning.<br>NOTE: FOR SOME INDIVIDUALS WITH MILDER EPISODES, FUNCTIONING MAY APPEAR TO BE NORMAL BUT REQUIRES MARKEDLY INCREASED EFFORT.	O ? O 1 O 2 O 3
Just before this began, were you physically ill?<br><br>Just before this began, were you drinking or taking any street drugs?<br><br>IF YES: Any change in the amount you were taking?<br><br>Just before this began, were you taking any medications?<br><br>IF YES: Any change in the amount you were taking?	C. Not due to the direct physiological effects of a substance (e.g., a drug of abuse, medication) or to a general medical condition (e.g., hypothyroidism)<br><br>IF GENERAL MEDICAL CONDITION OR SUBSTANCE MAY BE ETIOLOGICALLY ASSOCIATED WITH DEPRESSION, GO TO *GMC/SUBSTANCE* A.51, AND RETURN HERE TO MAKE RATING OF "1" OR "3."<br><br>IF THE EPISODE WAS PRECIPITATED BY MEDICATION TREATMENT, RECORD DETAILED INFORMATION ON THE MEDICATION ASSESSMENT FORM.	O ? O 1 O 3
Did this begin soon after someone close to you died?	D. Not better accounted for by Bereavement, i.e., after the loss of a loved one, the symptoms persist for longer than 2 months or are characterized by marked functional impairment, morbid preoccupation with worthlessness, suicidal ideation, psychotic symptoms or psychomotor retardation.	O 1 O 3
	**Major Depressive Episode**	
	MAJOR DEPRESSIVE EPISODE CRITERIA A, B, C, AND D ARE CODED "3"	false
	**Minor Depressive Episode**	
	MINOR DEPRESSIVE EPISODE CRITERIA A, B, C, AND D ARE CODED "3"	false

FIRST AND WORST DEPRESSION

IF UNCLEAR: Is this your worst episode of depression?

Worst

DETERMINE WHETHER OR NOT EACH EPISODE IS THE FIRST OR THE WORST DEPRESSION. CODE "3" FOR ONLY ONE FIRST AND ONE WORST EPISODE. THEY MAY BE THE SAME EPISODE AND MAY NOT ALWAYS BE CODED IN B OR C (I.E., D OR E MAY ACTUALLY BE EARLIER OR MORE SEVERE ONCE RECALLED).

O 1 O 3

Is this the first episode?

First

O 1 O 3

MIXED STATE

ASK THE FOLLOWING QUESTIONS TO DETERMINE WHETHER A MIXED STATE WAS PRESENT FOR EACH EPISODE OF DEPRESSION.

During this episode of depression did you have a week or more during which your mood changed between sadness and irritability or even elation?

O ? O 1 O 2 O 3

IF YES, CHECK IF:

Irritability O

Elation O

During this episode of depression did you also experience any of the following symptoms?

Over activity, such as running around, having many projects, or feeling physically agitated?

O ? O 1 O 2 O 3

More talkative than usual or feeling that your speech was pressured?

O ? O 1 O 2 O 3

Thoughts racing or jumping from topic to topic?

O ? O 1 O 2 O 3

Feeling grandiose, more important, special, or powerful?

O ? O 1 O 2 O 3

Needing less sleep or feeling energetic after little or no sleep?

O ? O 1 O 2 O 3

Attention distracted by unimportant things?

O ? O 1 O 2 O 3

Doing risky things for pleasure like excessive spending, reckless driving, sexual indiscretions, etc?

O ? O 1 O 2 O 3

NUMBER OF "3" RESPONSES FROM MIXED STATE SECTION. 0

How long were these symptoms present?

ENTER NUMBER OF DAYS

CRITERIA WERE MET SIMULTANEOUSLY FOR BOTH MAJOR DEPRESSION AND MANIA. IRRITABLE MOOD PLUS FOUR SYMPTOMS, OR ELATED MOOD PLUS THREE SYMPTOMS false

IRRITABLE MOOD PLUS 2-3 SYMPTOMS OR ELATED MOOD PLUS 2 SYMPTOMS false

Were your mood symptoms predominantly irritable, sad (dysphoria) or elated (euphoria)?

Predominance of:

Irritability O

Dysphoria O

Euphoria O

[PROBE IN THE SAME WAY FOR EACH CODED EPISODE]

During this episode of depression, did you have any beliefs or ideas that you later found out were not true? (Like believing that you had powers and abilities others did not have? Or that you had a special mission, perhaps from God? Or that someone was trying to harm you? How certain were you?)

Did you see or hear things other people could not see or hear?

Probe for Psychotic Symptoms per Episode:

IF DELUSIONS OR HALLUCINATIONS ARE SUSPECTED, PROBE FURTHER TO DETERMINE THE CONTENT AND WHETHER THE BELIEFS WERE HELD WITH CERTAINTY.

O ? O 1 O 3

IF YES, PLEASE CHECK:

O Delusions

O Hallucinations

IF YES, DESCRIBE:

End of Episode-Specific Questions. Will Another Episode Be Coded? O Yes O No

Episode E: Another Depression

Date of Onset | Age | Date of Offset | Duration (days)

Depression Criteria

Now I would like to ask you some more specific questions about (TIME PERIOD FOR SUSPECTED DEPRESSIVE EPISODE).

A. Five or more of the following symptoms have been present during the same two-week period and represent a change from previous functioning; at least one of the symptoms was either (1) depressed mood or (2) loss of interest or pleasure.

During this time, (TIME PERIOD FOR SUSPECTED DEPRESSIVE EPISODE) were you depressed or down, most of the day nearly every day? (What was that like?)

IF YES: When was that? How long did it last? As long as two weeks?

(1) depressed mood most of the day, nearly every day, as indicated by either subjective report (e.g., feels sad or empty) or observations made by others (e.g., appears tearful). Note: in children and adolescents, can be irritable mood.

O ? O 1 O 2 O 3

Did you lose interest or pleasure in things you usually enjoyed? (What was that like?)

IF YES: When was that? Was that nearly every day How long did it last? As long as two weeks?

(2) markedly diminished interest or pleasure in all, or almost all, activities most of the day, nearly every day (as indicated either by subjective account or observation made by others)

O ? O 1 O 2 O 3

FOR ALL SUBJECTS, CONTINUE ASKING ABOUT ALL SYMPTOMS EVEN IF A(1) AND/OR (2) ARE NOT ENDORSED.

NOTE: WHEN RATING THE FOLLOWING ITEMS, CODE "1" IF CLEARLY DUE TO A GENERAL MEDICAL CONDITION, SUBSTANCE, OR TO MOOD-INCONGRUENT DELUSIONS OR HALLUCINATIONS. TO COUNT TOWARD A MAJOR DEPRESSIVE EPISODE, A SYMPTOM MUST EITHER BE NEWLY PRESENT OR MUST HAVE CLEARLY WORSENED COMPARED WITH THE PERSON'S PRE-EPISODE STATUS

I would like you to focus on the worst two week period when answering the following questions. During (TIME PERIOD OF EPISODE)

FOCUS ON WORST TWO WEEK PERIOD OF EPISODE TO DETERMINE IF FULL MAJOR DEPRESSIVE EPISODE CRITERIA ARE MET

...did you lose or gain any weight? (How much? Were you trying to lose weight?)
IF NO: How was your appetite? What about compared to your usual appetite? Did you have to force yourself to eat? Eat (less/more) than usual? Was that nearly every day?

(3) significant weight loss when not dieting, or weight gain (e.g., a change of more than 5% of body weight in a month), or decrease or increase in appetite nearly every day. Note: in children, consider failure to make expected weight gains.

O ? O 1 O 2 O 3

Check if:

weight loss or decreased appetite O

weight gain or increased appetite O

...how were you sleeping? (Trouble falling asleep, waking frequently, trouble staying asleep, waking too early, OR sleeping too much? How many hours a night compared to usual? Was that nearly every night?)

(4) insomnia or hypersomnia nearly every day

O ? O 1 O 2 O 3

Check if:

insomnia O

hypersomnia O

...were you so fidgety or restless that you were unable to sit still? (Was it so bad that other people noticed it? What did they notice? Was that nearly every day?)<br>IF NO: What about the opposite-talking more slowly than is normal for you? Was it so bad that other people noticed it? What did they notice? Was it nearly every day?	(5) psychomotor agitation or retardation nearly every day (observable by others, not merely subjective feelings of restlessness or being slowed down)	O O O O<br>? 1 2 3

Check if:

psychomotor agitation O

psychomotor retardation O

...what was your energy like? (tired all the time? Nearly every day?)	(6) fatigue or loss of energy nearly every day	O O O O<br>? 1 2 3

...how did you feel about yourself? (Worthless? Nearly every day?)<br>IF NO: What about feeling guilty about things you had done or not done? Nearly every day?	(7) feelings of worthlessness or excessive or inappropriate guilt (which may be delusional) nearly every day (not merely self-reproach or guilt about being sick)	O O O O<br>? 1 2 3

Check if:

feelings of worthlessness O

excessive or inappropriate guilt O

...did you have trouble thinking or concentrating? (What kinds of things did it interfere with? Nearly every day?)<br>IF NO: Was it hard to make decisions about everyday things? Nearly every day?	(8) diminished ability to think or concentrate, or indecisiveness, nearly every day (either by subjective account or as observed by others)	O O O O<br>? 1 2 3

Check if:

diminished ability to think O

indecisiveness O

Were things so bad you were thinking a lot about death or that you would be better off dead? What about thinking of hurting yourself?<br>IF YES: Did you do anything to hurt yourself?	(9) recurrent thoughts of death (not just fear of dying), recurrent suicidal ideation without a specific plan, or a suicide attempt or a specific plan for committing suicide	O O O O<br>? 1 2 3

Check if:

thoughts of own death O

suicidal ideation O

specific plan O

actual attempt O

	NUMBER OF SYMPTOMS A(1) - A(9) CODED "3"	0
	**Major Depressive Episode**	
	AT LEAST FIVE OF A(1) - A(9) ARE CODED "3" AND EITHER A(1) OR A(2) ARE CODED "3"	false
	**Minor Depressive Episode**	
	EITHER TWO,THREE, OR FOUR OF A(1) - A(9) ARE CODED "3" AND EITHER A(1) OR A(2) ARE CODED "3"	false
IF UNCLEAR: Did (DEPRESSIVE EPISODE/OWN EQUIVALENT) make it hard for you to do your work, take care of things at home, or get along with other people?<br><br>IF YES, SPECIFY:	B. The symptoms cause clinically significant distress or impairment in social, occupational, or other important areas of functioning.<br>NOTE: FOR SOME INDIVIDUALS WITH MILDER EPISODES, FUNCTIONING MAY APPEAR TO BE NORMAL BUT REQUIRES MARKEDLY INCREASED EFFORT.	O ? O 1 O 2 O 3

Just before this began, were you physically ill?<br><br>Just before this began, were you drinking or taking any street drugs?<br><br>IF YES: Any change in the amount you were taking?<br><br>Just before this began, were you taking any medications?<br><br>IF YES: Any change in the amount you were taking?	C. Not due to the direct physiological effects of a substance (e.g., a drug of abuse, medication) or to a general medical condition (e.g., hypothyroidism)<br><br>IF GENERAL MEDICAL CONDITION OR SUBSTANCE MAY BE ETIOLOGICALLY ASSOCIATED WITH DEPRESSION, GO TO *GMC/SUBSTANCE* A.51, AND RETURN HERE TO MAKE RATING OF "1" OR "3."<br><br>IF THE EPISODE WAS PRECIPITATED BY MEDICATION TREATMENT, RECORD DETAILED INFORMATION ON THE MEDICATION ASSESSMENT FORM.	O ? O 1 O 3

Did this begin soon after someone close to you died?	D. Not better accounted for by Bereavement, i.e., after the loss of a loved one, the symptoms persist for longer than 2 months or are characterized by marked functional impairment, morbid preoccupation with worthlessness, suicidal ideation, psychotic symptoms or psychomotor retardation.	O 1 O 3

Major Depressive Episode

MAJOR DEPRESSIVE EPISODE CRITERIA A, B, C, AND D ARE CODED "3"

false

Minor Depressive Episode

MINOR DEPRESSIVE EPISODE CRITERIA A, B, C, AND D ARE CODED "3"

false

FIRST AND WORST DEPRESSION

IF UNCLEAR: Is this your worst episode of depression?

Worst

DETERMINE WHETHER OR NOT EACH EPISODE IS THE FIRST OR THE WORST DEPRESSION. CODE "3" FOR ONLY ONE FIRST AND ONE WORST EPISODE. THEY MAY BE THE SAME EPISODE AND MAY NOT ALWAYS BE CODED IN B OR C (I.E., D OR E MAY ACTUALLY BE EARLIER OR MORE SEVERE ONCE RECALLED).

O O
1 3

Is this the first episode?

First

O O
1 3

MIXED STATE

ASK THE FOLLOWING QUESTIONS TO DETERMINE WHETHER A MIXED STATE WAS PRESENT FOR EACH EPISODE OF DEPRESSION.

During this episode of depression did you have a week or more during which your mood changed between sadness and irritability or even elation?

O O O O
? 1 2 3

IF YES, CHECK IF:

Irritability O

Elation O

During this episode of depression did you also experience any of the following symptoms?

Over activity, such as running around, having many projects, or feeling physically agitated?

O O O O
? 1 2 3

More talkative than usual or feeling that your speech was pressured?

O O O O
? 1 2 3

Thoughts racing or jumping from topic to topic?

O O O O
? 1 2 3

Feeling grandiose, more important, special, or powerful?		O O O O<br>? 1 2 3
Needing less sleep or feeling energetic after little or no sleep?		O O O O<br>? 1 2 3
Attention distracted by unimportant things?		O O O O<br>? 1 2 3
Doing risky things for pleasure like excessive spending, reckless driving, sexual indiscretions, etc?		O O O O<br>? 1 2 3
	NUMBER OF "3" RESPONSES FROM MIXED STATE SECTION.	0
How long were these symptoms present?	ENTER NUMBER OF DAYS	
	CRITERIA WERE MET SIMULTANEOUSLY FOR BOTH MAJOR DEPRESSION AND MANIA. IRRITABLE MOOD PLUS FOUR SYMPTOMS, OR ELATED MOOD PLUS THREE SYMPTOMS	false
	IRRITABLE MOOD PLUS 2-3 SYMPTOMS OR ELATED MOOD PLUS 2 SYMPTOMS	false
Were your mood symptoms predominantly irritable, sad (dysphoria) or elated (euphoria)?	**Predominance of:**<br>Irritability O<br>Dysphoria O<br>Euphoria O	
[PROBE IN THE SAME WAY FOR EACH CODED EPISODE]<br><br>During this episode of depression, did you have any beliefs or ideas that you later found out were not true? (Like believing that you had powers and abilities others did not have? Or that you had a special mission, perhaps from God? Or that someone was trying to harm you? How certain were you?)<br><br>Did you see or hear things other people could not see or hear?	**Probe for Psychotic Symptoms per Episode:**<br>IF DELUSIONS OR HALLUCINATIONS ARE SUSPECTED, PROBE FURTHER TO DETERMINE THE CONTENT AND WHETHER THE BELIEFS WERE HELD WITH CERTAINTY.	O O O<br>? 1 3
	IF YES, PLEASE CHECK:	O Delusions<br>O Hallucinations

IF YES, DESCRIBE:

End of Episode-Specific Questions. Continue with Next Coded Episode.

Lifetime Probe for Psychotic Symptoms in Depression

Have you ever had either of these experiences (DELUSIONS/ HALLUCINATIONS) during any other periods of depression?	IF DELUSIONS OR HALLUCINATIONS ARE SUSPECTED, PROBE FURTHER TO DETERMINE THE CONTENT AND WHETHER THE BELIEFS WERE HELD WITH CERTAINTY.	O ? O 1 O 3

IF YES, DESCRIBE:

IF YES, PLEASE CHECK:

Delusions O

Hallucinations O

When you had experiences like this, were these topics and themes typical?	DETERMINE WHETHER THE DELUSIONS OR HALLUCINATIONS WERE CHARACTERISTICALLY MOOD CONGRUENT OR INCONGRUENT OVER THE LIFE SPAN	O Mood Congruent<br>O Mood Incongruent<br>O N/A<br>O Unknown<br>O No Information
	DETERMINE WHETHER OR NOT DELUSIONS HAVE EVER BEEN BIZARRE. IF UNSURE, SEE B.3 AND RETURN HERE TO CODE.	O Bizarre Delusions<br>O Non-Bizarre Delusions<br>O N/A<br>O Unknown
Do you usually have experiences like this (DELUSIONS/HALLUCINATIONS) when you have periods of depression?	DETERMINE WHETHER OR NOT PSYCHOTIC SYMPTOMS ARE TYPICAL OF MOST EPISODES OF DEPRESSION.	O Psychosis Typical<br>O Psychosis Not Typical<br>O N/A<br>O Unknown

General Depression: Clinical Data

PLEASE ANSWER ONLY APPLICABLE QUESTIONS (I.E., SKIP MAJOR DEPRESSION QUESTIONS IF MAJOR DEPRESSION NEVER ENDORSED). INFORMATION REGARDING MIXED EPISODES WILL BE SUMMARIZED AT THE END OF THE MANIA SECTION

Now I would like to ask you some general questions about depressive episodes and symptoms.	**Major Depression**	
How many separate times have you been (DEPRESSED/ OWN EQUIVALENT) nearly every day for at least two weeks and had several (five or more) of the symptoms that you described, like (SYMPTOMS OF WORST EPISODE)?	Total number of Major Depressive Episodes (MET CRITERIA)	
How old were you when you first had a lot of these symptoms for at least two weeks?	Age at onset of first unequivocal Major Depressive Episode (MET CRITERIA)	
What is the longest that a depression like this has lasted?	Duration of longest Major Depressive Episode (days) (MET CRITERIA)	

IF THERE ARE NUMEROUS EPISODES: How long do your depressions with many symptoms usually last?	Typical duration of Major Depressive Episodes (days)	
	**Minor Depression**	
How many separate times have you been (DEPRESSED/OWN EQUIVALENT) nearly every day for at least two weeks and had three or four of the symptoms that you described like (SYMPTOMS OF WORST EPISODE)?	Total number of Minor Depressive Episodes (MET CRITERIA)	
IF SUBJECT ENDORSED MAJOR DEPRESSIVE EPISODES: Did you ever have a period of time prior to age (AGE IDENTIFIED IN QUESTION ABOVE (B2)) when you had only a few of these symptoms for at least two weeks?	PROBE FOR POSSIBLE PRECEDING MINOR DEPRESSIVE EPISODES.	O ? O 1 O 3
IF YES, OR IF SUBJECT DID NOT ENDORSE MAJOR DEPRESSIVE EPISODES: How old were you when you first had a few of these symptoms for at least two weeks?	Age at onset of first unequivocal Minor Depressive Episode (MET CRITERIA)	
What is the longest that a depression with fewer symptoms like this has lasted?	Duration of longest Minor Depressive Episode (days) (MET CRITERIA)	
IF THERE ARE NUMEROUS EPISODES: How long do your depressed periods with fewer (2-4) symptoms usually last?	Typical duration of Minor Depressive Episodes (days)	
	**Depression Major/Minor**	
How old were you when you first had any symptoms of depression?	Age of first symptoms of depression	
	Maximum number of symptoms in a single episode (whether the episode meets criteria or not). Include Mixed if applicable.	
	GAF Ratings: (Click the Help button to display the scale to rate the following)	
	Typical episode of any depression	
	Worst week of most severe episode of any depression	
What portion of your life have you spent with any depressive symptoms (not including mixed)?	For Total Duration with any depressive symptoms, select one:	O Not at all (0%)<br>O Rarely (1-19%)<br>O Significant minority (20-39%)<br>O About half the time (40-69%)<br>O Significant majority (70-89%)<br>O Unknown<br>O N/A<br>O No Information

DEPRESSIVE EPISODE SPECIFIERS

**WITH POSTPARTUM ONSET**<br>**FEMALE SUBJECTS ONLY**	**POSTPARTUM ONSET CRITERIA**	
Have you ever had an episode of (DEPRESSION/OWN EQUIVALENT) which started	Has ever had a major depressive episode with onset within 4 weeks postpartum	O ? O 1 O 3

within a month of childbirth?

WITH CATATONIC FEATURES BY OBSERVATION OR HISTORY

CATATONIC FEATURES CRITERIA

Has ever had an episode in which the clinical picture was dominated by at least two of the following:

If Catatonic Features are not applicable check here:

O Check Here

(1) Motoric immobility as evidenced by catalepsy (including waxy flexibility) or stupor

O ? O 1 O 2 O 3

DESCRIBE SPECIFIC BEHAVIOR:

(2) Excessive motor activity (that is apparently purposeless and not influenced by external stimuli)

O ? O 1 O 2 O 3

DESCRIBE SPECIFIC BEHAVIOR:

(3) Extreme negativism (an apparently motiveless resistance to all instructions or maintenance or a rigid posture against attempts to be moved) or mutism

O ? O 1 O 2 O 3

DESCRIBE SPECIFIC BEHAVIOR:

(4) Peculiarities of voluntary movement as evidenced by posturing (voluntary assumption of inappropriate or bizarre postures), stereotyped movements, prominent mannerisms, or prominent grimacing

O ? O 1 O 2 O 3

DESCRIBE SPECIFIC BEHAVIOR:

(5) Echolalia (the pathological parrot-like, and apparently senseless repetition of a word or phrase just spoken by another person) or echopraxia (the repetitive imitation of the movements of another person).

O ? O 1 O 2 O 3

DESCRIBE SPECIFIC BEHAVIOR:

AT LEAST TWO CATATONIA ITEMS ARE "3"

false

WITH MELANCHOLIC FEATURES

IF UNKNOWN: Which was your worst (DEPRESSIVE EPISODE /OWN EQUIVALENT)? During that time when you were feeling the worst...

MELANCHOLIC FEATURES CRITERIA

A. Either of the following, occurring during the most severe period of the worst episode:

CODE BASED ON PAGE A2 (ITEM A2) IF WORST EPISODE PREVIOUSLY QUERIED	(1) Loss of pleasure in all, or almost all, activities.	O ? O 1 O 2 O 3
If something good happened to you or someone tried to cheer you up, did you feel better at least for a while?	(2) Lack of reactivity to usually pleasurable stimuli (does not feel much better, even temporarily, when something good happens)	O ? O 1 O 2 O 3
During that time when you were feeling the worst...	B. Three (or more) of the following:	
Was your feeling of (DEPRESSED MOOD/OWN EQUIVALENT) different from the kind of feeling you would get if someone close to you died? (Or something else bad happened to you?) IF YES: How is it different?	(1) Distinct quality of depressed mood (i.e., the depressed mood is perceived as distinctly different from the kind of feeling experience after the death of loved one)	O ? O 1 O 2 O 3
Did you usually feel worse in the morning?	(2) The depression is regularly worse in the morning	O ? O 1 O 2 O 3
CODE BASED ON PAGE A3 (ITEM A4) IF WORST EPISODE PREVIOUSLY QUERIED IF UNCLEAR: What time did you wake up in the morning? How much earlier is it than your usual time (before you were depressed)?	(3) Early morning awakening (at least two hours before usual time of awakening)	O ? O 1 O 2 O 3
CODE BASED ON PAGE A3 (ITEM A5) IF WORST EPISODE PREVIOUSLY QUERIED	(4) Marked psychomotor retardation or agitation	O ? O 1 O 2 O 3
CODE BASE ON PAGE A2 (ITEM A3) IF WORST EPISODE PREVIOUSLY QUERIED	(5) Significant anorexia or weight loss	O ? O 1 O 2 O 3
CODE BASED ON PAGE A3 (A7) IF WORST EPISODE PREVIOUSLY QUERIED IF UNCLEAR: Were you feeling guilty about things you had done or not done? IF YES: Tell me about that.	(6) Excessive or inappropriate guilt	O ? O 1 O 2 O 3
	AT LEAST THREE B ITEMS ARE CODED "3"	false
Were these symptoms typical for most of your episodes of (DEPRESSION / OWN EQUIVALENT)?	MELANCHOLIC FEATURES CRITERIA A AND B ARE CODED "3" AND ARE TYPICAL OF THE	O 1 O 3

MAJORITY OF DEPRESSIVE EPISODES.

WITH ATYPICAL FEATURES

IF LIFETIME COURSE HAS MELANCHOLIC FEATURES, CHECK HERE AND GO TO *MANIC EPISODE*

O Check Here

[NOTE: THE FOLLOWING QUESTION WAS ALREADY ASKED IN THE CONTEXT OF MELANCHOLIC FEATURES]<br>Usually when you are (DEPRESSED /OWN EQUIVALENT)...	**ATYPICAL FEATURES CRITERIA**<br>The following features characteristic of the majority of Major Depressive Episodes:	
if something good happens to you or someone tries to cheer you up, do you feel better, at least for a while?	A. Mood reactivity (i.e., mood brightens in response to actual or potential positive events.)	O ? O 1 O 2 O 3
[CODE BASED ON PREVIOUS ANSWERS OR ASK THE FOLLOWING IF UNKNOWN:]	B. Two (or more) of the following features:	
Do you gain weight or have an increased appetite?	(1) Significant weight gain or increase in appetite	O ? O 1 O 2 O 3
How many hours (in a 24-hour period) do you usually sleep (including naps)?	(2) hypersomnia NOTE: CODE "3" IF MORE THAN 10 HOURS A DAY	O ? O 1 O 2 O 3
Do your arms or legs often feel heavy (as though they were full of lead)?	(3) leaden paralysis (i.e., heavy leaden feelings in arms or legs)	O ? O 1 O 2 O 3
Are you especially sensitive to how others treat you? What happens to you when someone rejects, criticizes, or slights you? (Do you get very down or angry? For how long? How has this affected you? Is your reaction more extreme than most people's?) Have you avoided doing things or being with people because you were afraid of being criticized or rejected?	(4) longstanding pattern of interpersonal rejection sensitivity (not limited to episodes of mood disturbance) that results in significant social or occupational impairment	O ? O 1 O 2 O 3
	AT LEAST TWO "B" CRITERIA ARE CODED "3"	false
	C. Criteria are not met for "With Melancholic Features" or "With Catatonic Features" during the same episode.	O 1 O 3

Were these symptoms typical of most of your depressive episodes (OWN EQUIVALENT)?	ATYPICAL FEATURES CRITERIA A, B, AND C ARE CODED "3" AND ARE TYPICAL OF THE MAJORITY OF DEPRESSIVE EPISODES.	O 1 O 3

Module A: Mania

MANIC AND HYPOMANIC EPISODES

Episodes Summary

POSSIBLE MANIC OR HYPOMANIC EPISODES SHOULD BE REVIEWED IN THIS MODULE AS LISTED BELOW. YOU SHOULD GO THROUGH THIS SECTION AS MANY TIMES AS NECESSARY (UP TO 5), ONCE FOR EACH EPISODE AS DETERMINED FROM THE OVERVIEW IN ORDER TO DETERMINE IF A CURRENT EPISODE IS PRESENT, IF A PAST EPISODE WAS PRESENT, AN ESTIMATE OF THE NUMBER OF EPISODES, AND THE AGE AT ONSET OF THE FIRST EPISODE. THE LAST MONTH (A - CURRENT EPISODE) SHOULD ALWAYS BE REVIEWED. ALL SYMPTOMS SHOULD BE QUERIED FOR CURRENT AND SUSPECTED PAST EPISODES.

IF IT IS UNCLEAR FROM THE OVERVIEW WHETHER ANY PAST EPISODES HAVE OCCURRED, ASK QUESTIONS A1 AND A2 (p.A21 - A22) IN ORDER TO IDENTIFY POSSIBLE EPISODES OF MANIA OR HYPOMANIA FOR FURTHER CODING. ASSESS EACH UNCLEAR SUSPECTED EPISODE.
For example: "Have you ever had a period of time when you were feeling so good or hyper that other people thought you were not your normal self or you were so hyper that you got into trouble? When was it? How long did it last? ..."

A - THE LAST MONTH SHOULD BE REVIEWED FOR POSSIBLE CURRENT MANIA OR HYPOMANIA
B - THE MOST LIKELY WORST PAST EPISODE IF DIFFERENT FROM A
C - THE MOST LIKELY FIRST EPISODE IF DIFFERENT FROM A OR B
D - ANOTHER EPISODE AS NECESSARY TO DETERMINE RECURRENCE, # OF EPISODES, OR AGE AT ONSET
E - ANOTHER EPISODE AS NECESSARY TO DETERMINE RECURRENCE, # OF EPISODES, OR AGE AT ONSET

	Date of Onset	Age	Date of Offset	Duration (days)	
A - CURRENT (LAST MONTH)					O Go There
B - WORST EPISODE					O Go There
C - FIRST EPISODE					O Go There
D - ANOTHER EPISODE					O Go There
E - ANOTHER EPISODE					O Go There

Manic and Hypomanic Episode A (Current)

Date of Onset	Age	Date of Offset	Duration (days)

Now I'd like to ask you more specific questions about (TIME PERIOD FOR SUSPECTED MANIC OR HYPOMANIC EPISODE).	**MANIC EPISODE CRITERIA**	

A1. (Mania and Hypomania)

During (TIME PERIOD FOR EPISODE) were you feeling so good or hyper that other people thought you were not your normal self or you were so hyper that you got into trouble? (Did anyone say you were manic? Was that more than just feeling good?)<br><br>IF NO: What about feeling so irritable that you found yourself shouting at people or starting fights or arguments? Did you find yourself shouting at people you really didn't know?	A(1) A distinct period of abnormally and persistently ("sustained" if hypomania) elevated, expansive, or irritable mood.	O ? O 1 O 2 O 3
What was it like?	CHECK ONE:	O elevated/expansive mood<br>O irritable mood

	Select if this is a manic or hypomanic episoide	O Manic O Hypomanic

A2. (Mania)

How long did that last? (As long as one week? Did you have to go to the hospital?)	A(2) Episode lasted at least one week (any duration if hospitalization is necessary, psychosis is present, or very dangerous behaviors are present)	O 1 O 3
Did it last for at least two days?	Brief Mania (2 day duration required) PER KELSOE CONVENTION, BRIEF MANIA WILL BE INCLUDED IN THE MANIA ASSESSMENT, SPECIFIC PATTERNS.	O 1 O 3

A2. (Hypomania)

Did it last for at least four days? What was that like?	A(2) Episode lasted throughout at least 4 days, and is clearly different from the usual non-depressed mood	O 1 O 3
What was it like?	CHECK ONE:	O elevated/expansive mood<br>O irritable mood
Did it last for at least two days?	Brief Hypomania (2 day duration required)<br>PER KELSOE CONVENTION, BRIEF HYPOMANIA WILL BE INCLUDED IN THE HYPOMANIA ASSESSMENT	O 1 O 3

FOR ALL SUBJECTS, CONTINUE ASKING ABOUT ALL SYMPTOMS, EVEN IF A(1) AND (2) ARE NOT ENDORSED

NOTE: WHEN RATING THE FOLLOWING ITEMS, CODE "1" IF CLEARLY DUE TO A GENERAL MEDICAL CONDITION, SUBSTANCE, OR TO MOOD-INCONGRUENT DELUSIONS OR HALLUCINATIONS. TO COUNT TOWARD A MANIC EPISODE, A SYMPTOM MUST EITHER BE NEWLY PRESENT OR MUST HAVE CLEARLY WORSENED COMPARED WITH THE PERSON'S PRE-EPISODE STATUS.

B. (Mania and Hypomania)

I would like you to focus on the most extreme period of feeling (OWN EQUIVALENT FOR EUPHORIA OR IRRITABILITY), when answering the following questions. During (TIME PERIOD OF EPISODE)	B. During the worst period of the mood disturbance, three (or more) of the following symptoms have persisted (four if the mood is only irritable) and have been present to a significant degree:	
...how did you feel about yourself? (More self-confident than usual? Any special powers or abilities?)	(1) inflated self-esteem or grandiosity	O ? O 1 O 2 O 3

Did you need less sleep than usual?<br><br>IF YES: Did you still feel rested?	(2) decreased need for sleep (e.g., feels rested after missing at least two hours of sleep) NOTE: THIS ITEM SHOULD BE PRESENTED AT EVERY CONSENSUS TO HELP ENSURE RELIABILITY.	O O O O<br>? 1 2 3
Were you much more talkative than usual? (Did people have trouble stopping you or understanding you? Did people have trouble getting a word in edgewise?)	(3) more talkative than usual or pressure to keep talking	O O O O<br>? 1 2 3
Were your thoughts racing through your head?	(4) flight of ideas or subjective experience that thoughts are racing	O O O O<br>? 1 2 3
Were you so easily distracted by things around you that you had trouble concentrating or staying on one track?	(5) distractibility (i.e., attention too easily drawn to unimportant or irrelevant external stimuli)	O O O O<br>? 1 2 3
How did you spend your time? (Work, friends, hobbies? Were you so active that your friends or family were concerned about you?)<br><br>IF NO INCREASED ACTIVITY: Were you physically restless? How bad was it?	(6) increase in goal-directed activity (either socially, at work or school, or sexually) or psychomotor agitation<br><br>Check if:	O O O O<br>? 1 2 3<br><br>O psychomotor agitation<br>O increase in activity
Did you do anything that could have caused trouble for you or your family? (Buying things you didn't need? Anything sexual that was unusual for you? Reckless driving?)	(7) excessive involvement in pleasurable activities which have a high potential for painful consequences (e.g., engaging in unrestrained buying sprees, sexual indiscretions, or foolish business investments)	O O O O<br>? 1 2 3
	NUMBER OF MANIC/HYPOMANIC SYMPTOMS IN A AND B CODED "3" (A1 AND A2 CRITERIA COUNT AS ONE).	0
	AT LEAST THREE B SYMPTOMS ARE CODED "3" (FOUR IF MOOD ONLY IRRITABLE)	false
	Note: DSM-IV Criterion C for Mania (i.e., does not meet criteria for a Mixed Episode) has been omitted from the SCID	

C. (Mania)

IF UNKNOWN: At that time, did you have serious problems at home or at work (school) because you were (SYMPTOMS) or did you have to be admitted to a hospital?<br><br>IF YES, SPECIFY:	C. The mood disturbance is sufficiently severe to cause marked impairment in occupational functioning or in usual social activities or relationships with others, or to necessitate hospitalization to prevent harm to self or others, or there are psychotic features.	O O<br>1 3

C. (Hypomania)

IF UNKNOWN: Is this very different from the way you usually are? (How were you different? At work? With friends?) IF YES, Specify:	C. The episode is associated with an unequivocal change in functioning that is uncharacteristic of the person when not symptomatic	O O<br>1 3

D. (Hypomania)

IF UNKNOWN: Did other people notice the change in you? (What did they say?)	D. The disturbance in mood and the change in functioning are observable by others	O O<br>1 3

E. (Hypomania)

IF UNKNOWN: At that time, did you have serious problems at home or at work (school) because you were (SYMPTOMS) or did you have to be admitted to a hospital?	E. The episode is not severe enough to cause marked impairment in social or occupational functioning, or to necessitate hospitalization, and there are no psychotic features	O O<br>1 3

D. (Mania)
F. (Hypomania)

Just before this began, were you physically ill?<br><br>Just before this began, were you drinking or taking any street drugs?<br><br>IF YES: Any change in the amount you were taking?<br><br>Just before this began, were you taking any medications ,other than antidepressants?<br><br>IF YES: Any change in the amount you were taking?	D/F. Not due to the direct physiological effects of a substance (e.g., a drug of abuse, medication) or to a general medical condition<br><br>IF GENERAL MEDICAL CONDITION OR SUBSTANCE THAT CAN BE ETIOLOGICALLY ASSOCIATED WITH MANIA/HYPOMANIA, GO TO *GMC/SUBSTANCE,* A.51 AND RETURN HERE TO MAKE RATING OF "1" OR "3"<br><br>Etiological general medical conditions include: degenerative neurological illnesses (e.g., Huntington's disease, multiple sclerosis), cerebrovascular disease (e.g., stroke), metabolic conditions (e.g., Vitamin B-12 deficiency, Wilson's disease), endocrine conditions (e.g., hyperthyroidism), viral or other infections, and certain cancers (e.g., cerebral neoplasms).<br><br>Etiological substances include: alcohol, amphetamines, cocaine, hallucinogens, inhalants, opioids, phencyclidine, sedatives, hypnotics, and anxiolytics. Medications include psychotropic medications (e.g., anxiolytics), corticosteroids, anabolic steroids, isoniazid, antiparkinson medication (e.g., levodopa), and sympathomimetics/decongestants	O O<br>1 3

Were you on antidepressant treatment when this episode began?	EPISODE PRECIPITATED BY SOMATIC ANTIDEPRESSANT TREATMENT (BEGAN WITHIN TWO MONTHS OF STARTING OR CHANGING ANTIDEPRESSANT TREATMENT). CODE "3" IF APPLICABLE TO EPISODE<br><br>IF YES RECORD IN MEDICATION SECTION OF OVERVIEW<br><br>NOTE: FOR THE GENETICS STUDY AND IN DISTINCTION TO DSM IV, MANIC AND HYPOMANIC EPISODES THAT ARE CLEARLY PRECIPITATED BY SOMATIC ANTIDEPRESS-ANT TREATMENT (E.G., MEDICATION, ELECTROCONVULSIVE THERAPY, LIGHT THERAPY, SLEEP DEPRIVATION, HERBAL TREATMENTS) DO COUNT TOWARD A DIAGNOSIS OF BIPOLAR DISORDER	O O<br>1 3

IF YES, How long were you on it?	RECORD NUMBER OF WEEKS	
	MANIC EPISODE CRITERIA A, B, C AND D ARE CODED "3"	false
	HYPOMANIC EPISODE CRITERIA A, B, C, D, E, AND F ARE CODED "3"	false
	IF NO MANIC OR HYPOMANIC OR MIXED EPISODES, GO TO DYSTHYMIC DISORDER. HOWEVER, IF CODED FOR A MIXED EPISODE IN THE DEPRESSION SECTION, SKIP TO SUMMARY QUESTIONS FOR MIXED EPISODE.	
	**FIRST AND WORST MANIA/HYPOMANIA:**	
IF UNCLEAR:<br>Is this your worst episode of depression?	DETERMINE WHETHER OR NOT EACH EPISODE IS THE FIRST AND/OR THE WORST MANIA/HYPOMANIA. CODE "3" FOR ONLY ONE FIRST AND ONE WORST EPISODE. THEY MAY BE THE SAME EPISODE AND MAY NOT ALWAYS BE CODED IN B OR C (I.E., D OR E MAY ACTUALLY BE EARLIER OR MORE SEVERE ONCE RECALLED).	O O<br>1 3
Is this the first episode?		O O<br>1 3

	**SUICIDALITY IN MANIA**	
IF UNKNOWN: Have you ever attempted suicide during a manic episode?	Has made a suicide attempt	O O O O<br>? 1 2 3

IF YES: How many times have you attempted suicide during a manic episode?	Lifetime total number of suicide attempts during Manic Episodes (score Mixed Episodes in the next section)	
Do you think about suicide during most of your manic episodes?	Determine whether suicide ideation is present during most manic episodes	O ? O 1 O 2 O 3

MIXED STATE

ASK THE FOLLOWING QUESTIONS TO DETERMINE WHETHER A MIXED STATE WAS PRESENT FOR EACH EPISODE OF MANIA/HYPOMANIA.

During this episode of (MANIA/HYPOMANIA)did you have a week or more during which your mood changed between elation, irritability, or sadness?		O ? O 1 O 2 O 3

IF YES, SPECIFY:

During this episode of (MANIA/HYPOMANIA) did you also experience any of the following symptoms?

Diminished desire for food or marked overeating?		O ? O 1 O 3
Inability to sleep when sleep was desired, or excessive sleep?	[NOTE: DIFFERENTIATE BETWEEN LACK OF SLEEP DUE TO MANIA]	O ? O 1 O 3
Feeling slowed down?		O ? O 1 O 3
Having fatigue or loss of energy?		O ? O 1 O 3
Losing interest in pleasurable activities?		O ? O 1 O 3
Feeling guilty or worthless?		O ? O 1 O 3

Being unable to think or retain written information?	[NOTE: DIFFERENTIATE BETWEEN RACING THOUGHTS/DISTRACTIBILITY DUE TO MANIA]	O O O<br>? 1 3
Were things so bad you were thinking a lot about death or that you would be better off dead? What about thinking of hurting yourself?<br><br>IF YES: Did you do anything to hurt yourself?		O O O<br>? 1 3
	Check if:	O thoughts of own death<br>O suicidal ideation<br>O specific plan<br>O actual attempt
	INTERVIEWER: ENTER NUMBER OF "3" RESPONSES FOR THE NINE MIXED STATE SYMPTOMS (EXCLUDE ABOVE THREE SUCIDALITY QUESTIONS)	0
How long were these symptoms present?	ENTER NUMBER OF DAYS	

SUICIDALITY IN MIXED STATES:

FOLLOWING EPISODE A, ASK THE THREE QUESTIONS BELOW REGARDING SUICIDALITY, THEN CONTINUE WITH REMAINDER OF EPISODE A. FOR EPISODES B-E, SKIP THIS SECTION AND GO TO NUMBER OF SYMPTOMS (BELOW).

IF UNKNOWN: Have you ever attempted suicide during a mixed episode?	Has made a suicide attempt	O O O O<br>? 1 2 3
IF YES: How many times have you attempted suicide during a mixed episode?	Lifetime total number of suicide attempts during a Mixed State	
Do you think about suicide during most of your mixed episodes?	Determine whether suicidal ideation is present during most Mixed Episodes	O O O O<br>? 1 2 3
	CRITERIA WERE MET SIMULTANEOUSLY FOR BOTH MAJOR DEPRESSION AND MANIA.	O O<br>1 3
	TWO TO FOUR CRITERIA WERE MET FOR DEPRESSION	O O<br>1 3
Were your mood symptoms predominantly irritable, sad (dysphoria) or elated (euphoria)?	Predominance of:	O Irritability

O Dysphoria

O Euphoria

[PROBE FOR PSYCHOTIC SYMPOTMS IN EACH CODED EPISODE. INCLUDE MIXED STATES IN NEXT SECTION]

Probe for Psychotic Symptoms: Per Episode

During this episode of (MANIA) did you have any beliefs or ideas that you later found out were not true? (Like believing that you had powers and abilities others did not have? Or that you had a special mission, perhaps from God? Or that someone was trying to harm you? How certain were you?)
IF YES, DESCRIBE:

IF DELUSIONS OR HALLUCINATIONS ARE SUSPECTED, PROBE FURTHER TO DETERMINE THE CONTENT AND WHETHER THE BELIEFS WERE HELD WITH CERTAINTY. [NOTE: IF PSYCHOTIC SYMPTOMS ARE PRESENT DURING PREVIOUSLY CODED HYPOMANIA, IT SHOULD BE RECODED AS FULL MANIA.]

O ? O 1 O 3

Did you see or hear things other people could not see or hear?
IF YES, DESCRIBE:

IF YES, PLEASE CHECK:

O Delusions

O Hallucinations

END OF EPISODE SPECIFIC QUESTIONS. CONTINUE WITH NEXT CODED EPISODE.

AFTER REVIEWING ALL NECESSARY EPISODES, CONTINUE BELOW.

Manic and Hypomanic Episode B (Worst)

Date of Onset	Age	Date of Offset	Duration (days)

Now I'd like to ask you more specific questions about (TIME PERIOD FOR SUSPECTED MANIC OR HYPOMANIC EPISODE).

MANIC EPISODE CRITERIA

A1. (Mania and Hypomania)

During (TIME PERIOD FOR EPISODE) were you feeling so good or hyper that other people thought you were not your normal self or you were so hyper that you got into trouble? (Did anyone say you were manic? Was that more than just feeling good?)

IF NO: What about feeling so irritable that you found yourself shouting at people or starting fights or arguments? Did you find yourself shouting at people you really didn't know?

A(1) A distinct period of abnormally and persistently ("sustained" if hypomania) elevated, expansive, or irritable mood.

O ? O 1 O 2 O 3

What was it like?

CHECK ONE:

O elevated/expansive mood

O irritable mood

Select if this is a manic or hypomanic episoide O O

		Manic	Hypomanic

A2. (Mania)

How long did that last? (As long as one week? Did you have to go to the hospital?)	A(2) Episode lasted at least one week (any duration if hospitalization is necessary, psychosis is present, or very dangerous behaviors are present)	O<br>1	O<br>3
Did it last for at least two days?	Brief Mania<br>(2 day duration required)<br>PER KELSOE CONVENTION, BRIEF MANIA WILL BE INCLUDED IN THE MANIA ASSESSMENT, SPECIFIC PATTERNS.	O<br>1	O<br>3

A2. (Hypomania)

Did it last for at least four days?<br><br>What was that like?	A(2) Episode lasted throughout at least 4 days, and is clearly different from the usual non-depressed mood	O<br>1	O<br>3
What was it like?	CHECK ONE:	O elevated/expansive mood<br><br>O irritable mood	
Did it last for at least two days?	Brief Hypomania<br>(2 day duration required)<br><br>PER KELSOE CONVENTION, BRIEF HYPOMANIA WILL BE INCLUDED IN THE HYPOMANIA ASSESSMENT	O<br>1	O<br>3

FOR ALL SUBJECTS, CONTINUE ASKING ABOUT ALL SYMPTOMS, EVEN IF A(1) AND (2) ARE NOT ENDORSED

NOTE: WHEN RATING THE FOLLOWING ITEMS, CODE "1" IF CLEARLY DUE TO A GENERAL MEDICAL CONDITION, SUBSTANCE, OR TO MOOD-INCONGRUENT DELUSIONS OR HALLUCINATIONS. TO COUNT TOWARD A MANIC EPISODE, A SYMPTOM MUST EITHER BE NEWLY PRESENT OR MUST HAVE CLEARLY WORSENED COMPARED WITH THE PERSON'S PRE-EPISODE STATUS.

B. (Mania and Hypomania)

I would like you to focus on the most extreme period of feeling (OWN EQUIVALENT FOR EUPHORIA OR IRRITABILITY), when answering the following questions. During (TIME PERIOD OF EPISODE)	B. During the worst period of the mood disturbance, three (or more) of the following symptoms have persisted (four if the mood is only irritable) and have been present to a significant degree:				
...how did you feel about yourself? (More self-confident than usual? Any special powers or abilities?)	(1) inflated self-esteem or grandiosity	O<br>?	O<br>1	O<br>2	O<br>3

Did you need less sleep than usual?<br><br>IF YES: Did you still feel rested?	(2) decreased need for sleep (e.g., feels rested after missing at least two hours of sleep) NOTE: THIS ITEM SHOULD BE PRESENTED AT EVERY CONSENSUS TO HELP ENSURE RELIABILITY.	O ? O 1 O 2 O 3
Were you much more talkative than usual? (Did people have trouble stopping you or understanding you? Did people have trouble getting a word in edgewise?)	(3) more talkative than usual or pressure to keep talking	O ? O 1 O 2 O 3
Were your thoughts racing through your head?	(4) flight of ideas or subjective experience that thoughts are racing	O ? O 1 O 2 O 3
Were you so easily distracted by things around you that you had trouble concentrating or staying on one track?	(5) distractibility (i.e., attention too easily drawn to unimportant or irrelevant external stimuli)	O ? O 1 O 2 O 3
How did you spend your time? (Work, friends, hobbies? Were you so active that your friends or family were concerned about you?)<br><br>IF NO INCREASED ACTIVITY: Were you physically restless? How bad was it?	(6) increase in goal-directed activity (either socially, at work or school, or sexually) or psychomotor agitation<br><br>Check if:	O ? O 1 O 2 O 3<br><br>O psychomotor agitation<br>O increase in activity
Did you do anything that could have caused trouble for you or your family? (Buying things you didn't need? Anything sexual that was unusual for you? Reckless driving?)	(7) excessive involvement in pleasurable activities which have a high potential for painful consequences (e.g., engaging in unrestrained buying sprees, sexual indiscretions, or foolish business investments)	O ? O 1 O 2 O 3
	NUMBER OF MANIC/HYPOMANIC SYMPTOMS IN A AND B CODED "3" (A1 AND A2 CRITERIA COUNT AS ONE).	0
	AT LEAST THREE B SYMPTOMS ARE CODED "3" (FOUR IF MOOD ONLY IRRITABLE)	false
	Note: DSM-IV Criterion C for Mania (i.e., does not meet criteria for a Mixed Episode) has been omitted from the SCID	

IF UNKNOWN: At that time, did you have serious problems at home or at work (school) because you were (SYMPTOMS) or did you have to be admitted to a hospital?<br><br>IF YES, SPECIFY:	C. The mood disturbance is sufficiently severe to cause marked impairment in occupational functioning or in usual social activities or relationships with others, or to necessitate hospitalization to prevent harm to self or others, or there are psychotic features.	O O<br>1 3

C. (Hypomania)

IF UNKNOWN: Is this very different from the way you usually are? (How were you different? At work? With friends?) IF YES, Specify:	C. The episode is associated with an unequivocal change in functioning that is uncharacteristic of the person when not symptomatic	O O<br>1 3

D. (Hypomania)

IF UNKNOWN: Did other people notice the change in you? (What did they say?)	D. The disturbance in mood and the change in functioning are observable by others	O O<br>1 3

E. (Hypomania)

IF UNKNOWN: At that time, did you have serious problems at home or at work (school) because you were (SYMPTOMS) or did you have to be admitted to a hospital?	E. The episode is not severe enough to cause marked impairment in social or occupational functioning, or to necessitate hospitalization, and there are no psychotic features	O O<br>1 3

D. (Mania)
F. (Hypomania)

Just before this began, were you physically ill?<br><br>Just before this began, were you drinking or taking any street drugs?<br><br>IF YES: Any change in the amount you were taking?<br><br>Just before this began, were you taking any medications ,other than antidepressants?<br><br>IF YES: Any change in the amount you were taking?	D/F. Not due to the direct physiological effects of a substance (e.g., a drug of abuse, medication) or to a general medical condition<br><br>IF GENERAL MEDICAL CONDITION OR SUBSTANCE THAT CAN BE ETIOLOGICALLY ASSOCIATED WITH MANIA/HYPOMANIA, GO TO *GMC/SUBSTANCE,* A.51 AND RETURN HERE TO MAKE RATING OF "1" OR "3"<br><br>Etiological general medical conditions include: degenerative neurological illnesses (e.g., Huntington's disease, multiple sclerosis), cerebrovascular disease (e.g., stroke), metabolic conditions (e.g., Vitamin B-12 deficiency, Wilson's disease), endocrine conditions (e.g., hyperthyroidism), viral or other infections, and certain cancers (e.g., cerebral neoplasms).<br><br>Etiological substances include: alcohol, amphetamines, cocaine, hallucinogens, inhalants, opioids, phencyclidine, sedatives, hypnotics, and anxiolytics. Medications include psychotropic medications (e.g., anxiolytics), corticosteroids, anabolic steroids, isoniazid, antiparkinson medication (e.g., levodopa), and sympathomimetics/decongestants	O O<br>1 3

Were you on antidepressant treatment when this episode began?	EPISODE PRECIPITATED BY SOMATIC ANTIDEPRESSANT TREATMENT (BEGAN WITHIN TWO MONTHS OF STARTING OR CHANGING ANTIDEPRESSANT TREATMENT). CODE "3" IF APPLICABLE TO EPISODE<br><br>IF YES RECORD IN MEDICATION SECTION OF OVERVIEW<br><br>NOTE: FOR THE GENETICS STUDY AND IN DISTINCTION TO DSM IV, MANIC AND HYPOMANIC EPISODES THAT ARE CLEARLY PRECIPITATED BY SOMATIC ANTIDEPRESS-ANT TREATMENT (E.G., MEDICATION, ELECTROCONVULSIVE THERAPY, LIGHT THERAPY, SLEEP DEPRIVATION, HERBAL TREATMENTS) DO COUNT TOWARD A DIAGNOSIS OF BIPOLAR DISORDER	O O<br>1 3

IF YES, How long were you on it?	RECORD NUMBER OF WEEKS	
	MANIC EPISODE CRITERIA A, B, C AND D ARE CODED "3"	false
	HYPOMANIC EPISODE CRITERIA A, B, C, D, E, AND F ARE CODED "3"	false
	IF NO MANIC OR HYPOMANIC OR MIXED EPISODES, GO TO DYSTHYMIC DISORDER. HOWEVER, IF CODED FOR A MIXED EPISODE IN THE DEPRESSION SECTION, SKIP TO SUMMARY QUESTIONS FOR MIXED EPISODE.	
	**FIRST AND WORST MANIA/HYPOMANIA:**	
IF UNCLEAR:<br>Is this your worst episode of depression?	DETERMINE WHETHER OR NOT EACH EPISODE IS THE FIRST AND/OR THE WORST MANIA/HYPOMANIA. CODE "3" FOR ONLY ONE FIRST AND ONE WORST EPISODE. THEY MAY BE THE SAME EPISODE AND MAY NOT ALWAYS BE CODED IN B OR C (I.E., D OR E MAY ACTUALLY BE EARLIER OR MORE SEVERE ONCE RECALLED).	O O<br>1 3
Is this the first episode?		O O<br>1 3

MIXED STATE

ASK THE FOLLOWING QUESTIONS TO DETERMINE WHETHER A MIXED STATE WAS PRESENT FOR EACH EPISODE OF MANIA/HYPOMANIA.

During this episode of (MANIA/HYPOMANIA)did you have a week or more during which your mood changed between elation, irritability, or sadness?

O O O O
? 1 2 3

IF YES, SPECIFY:

During this episode of (MANIA/HYPOMANIA) did you also experience any of the following symptoms?

Diminished desire for food or marked overeating?

O O O
? 1 3

Inability to sleep when sleep was desired, or excessive sleep?

[NOTE: DIFFERENTIATE BETWEEN LACK OF SLEEP DUE TO MANIA]

O O O
? 1 3

Feeling slowed down?

O O O
? 1 3

Having fatigue or loss of energy?

O O O
? 1 3

Losing interest in pleasurable activities?

O O O
? 1 3

Feeling guilty or worthless?

O O O
? 1 3

Being unable to think or retain written information?

[NOTE: DIFFERENTIATE BETWEEN RACING THOUGHTS/DISTRACTIBILITY DUE TO MANIA]

O O O
? 1 3

Were things so bad you were thinking a lot about death or that you would be better off dead? What about thinking of hurting yourself?

O O O
? 1 3

IF YES: Did you do anything to hurt yourself?

	Check if:	O thoughts of own death<br>O suicidal ideation<br>O specific plan<br>O actual attempt
	INTERVIEWER: ENTER NUMBER OF "3" RESPONSES FOR THE NINE MIXED STATE SYMPTOMS (EXCLUDE ABOVE THREE SUCIDALITY QUESTIONS)	0
How long were these symptoms present?	ENTER NUMBER OF DAYS	
	CRITERIA WERE MET SIMULTANEOUSLY FOR BOTH MAJOR DEPRESSION AND MANIA.	O O<br>1 3
	TWO TO FOUR CRITERIA WERE MET FOR DEPRESSION	O O<br>1 3
[PROBE FOR PSYCHOTIC SYMPOTMS IN EACH CODED EPISODE. INCLUDE MIXED STATES IN NEXT SECTION]	**Probe for Psychotic Symptoms: Per Episode**	
During this episode of (MANIA) did you have any beliefs or ideas that you later found out were not true? (Like believing that you had powers and abilities others did not have? Or that you had a special mission, perhaps from God? Or that someone was trying to harm you? How certain were you?)<br>IF YES, DESCRIBE:<br><br>Did you see or hear things other people could not see or hear?<br>IF YES, DESCRIBE:	IF DELUSIONS OR HALLUCINATIONS ARE SUSPECTED, PROBE FURTHER TO DETERMINE THE CONTENT AND WHETHER THE BELIEFS WERE HELD WITH CERTAINTY. [NOTE: IF PSYCHOTIC SYMPTOMS ARE PRESENT DURING PREVIOUSLY CODED HYPOMANIA, IT SHOULD BE RECODED AS FULL MANIA.]	O O O<br>? 1 3
	IF YES, PLEASE CHECK:	O Delusions<br>O Hallucinations

END OF EPISODE SPECIFIC QUESTIONS. CONTINUE WITH NEXT CODED EPISODE.

AFTER REVIEWING ALL NECESSARY EPISODES, CONTINUE BELOW.

Manic and Hypomanic Episode C (First)

Date of Onset	Age	Date of Offset	Duration (days)

Now I'd like to ask you more specific questions about (TIME PERIOD FOR SUSPECTED MANIC OR HYPOMANIC EPISODE).	**MANIC EPISODE CRITERIA**	
**A1. (Mania and Hypomania)**		
During (TIME PERIOD FOR EPISODE) were you	A(1) A distinct period of abnormally and	O O O O

feeling so good or hyper that other people thought you were not your normal self or you were so hyper that you got into trouble? (Did anyone say you were manic? Was that more than just feeling good?)<br><br>IF NO: What about feeling so irritable that you found yourself shouting at people or starting fights or arguments? Did you find yourself shouting at people you really didn't know?	persistently ("sustained" if hypomania) elevated, expansive, or irritable mood.	? 1 2 3
What was it like?	CHECK ONE:	O elevated/expansive mood<br><br>O irritable mood

	Select if this is a manic or hypomanic episoide	O Manic O Hypomanic

A2. (Mania)

How long did that last? (As long as one week? Did you have to go to the hospital?)	A(2) Episode lasted at least one week (any duration if hospitalization is necessary, psychosis is present, or very dangerous behaviors are present)	O O<br>1 3

Did it last for at least two days?	Brief Mania<br>(2 day duration required)<br>PER KELSOE CONVENTION, BRIEF MANIA WILL BE INCLUDED IN THE MANIA ASSESSMENT, SPECIFIC PATTERNS.	O O<br>1 3

A2. (Hypomania)

Did it last for at least four days?<br><br>What was that like?	A(2) Episode lasted throughout at least 4 days, and is clearly different from the usual non-depressed mood	O O<br>1 3
What was it like?	CHECK ONE:	O elevated/expansive mood<br><br>O irritable mood

Did it last for at least two days?	Brief Hypomania<br>(2 day duration required)<br><br>PER KELSOE CONVENTION, BRIEF HYPOMANIA WILL BE INCLUDED IN THE HYPOMANIA ASSESSMENT	O O<br>1 3

FOR ALL SUBJECTS, CONTINUE ASKING ABOUT ALL SYMPTOMS, EVEN IF A(1) AND (2) ARE NOT ENDORSED

NOTE: WHEN RATING THE FOLLOWING ITEMS, CODE "1" IF CLEARLY DUE TO A GENERAL MEDICAL CONDITION, SUBSTANCE, OR TO MOOD-INCONGRUENT DELUSIONS OR HALLUCINATIONS. TO COUNT TOWARD A MANIC EPISODE, A SYMPTOM MUST EITHER BE NEWLY PRESENT OR MUST HAVE CLEARLY WORSENED COMPARED WITH THE PERSON'S PRE-EPISODE STATUS.

B. (Mania and Hypomania)

I would like you to focus on the most extreme period of feeling (OWN EQUIVALENT FOR EUPHORIA OR IRRITABILITY), when answering the following questions. During (TIME PERIOD OF EPISODE)	B. During the worst period of the mood disturbance, three (or more) of the following symptoms have persisted (four if the mood is only irritable) and have been present to a significant degree:	
...how did you feel about yourself? (More self-confident than usual? Any special powers or abilities?)	(1) inflated self-esteem or grandiosity	O ? O 1 O 2 O 3
Did you need less sleep than usual?<br><br>IF YES: Did you still feel rested?	(2) decreased need for sleep (e.g., feels rested after missing at least two hours of sleep) NOTE: THIS ITEM SHOULD BE PRESENTED AT EVERY CONSENSUS TO HELP ENSURE RELIABILITY.	O ? O 1 O 2 O 3
Were you much more talkative than usual? (Did people have trouble stopping you or understanding you? Did people have trouble getting a word in edgewise?)	(3) more talkative than usual or pressure to keep talking	O ? O 1 O 2 O 3
Were your thoughts racing through your head?	(4) flight of ideas or subjective experience that thoughts are racing	O ? O 1 O 2 O 3
Were you so easily distracted by things around you that you had trouble concentrating or staying on one track?	(5) distractibility (i.e., attention too easily drawn to unimportant or irrelevant external stimuli)	O ? O 1 O 2 O 3
How did you spend your time? (Work, friends, hobbies? Were you so active that your friends or family were concerned about you?)<br><br>IF NO INCREASED ACTIVITY: Were you physically restless? How bad was it?	(6) increase in goal-directed activity (either socially, at work or school, or sexually) or psychomotor agitation<br><br>Check if:	O ? O 1 O 2 O 3<br><br>O psychomotor agitation<br>O increase in activity
Did you do anything that could have caused trouble for you or your family? (Buying things you didn't	(7) excessive involvement in pleasurable activities which have a high potential for painful	O ? O 1 O 2 O 3

need? Anything sexual that was unusual for you? Reckless driving?)	consequences (e.g., engaging in unrestrained buying sprees, sexual indiscretions, or foolish business investments)	

	NUMBER OF MANIC/HYPOMANIC SYMPTOMS IN A AND B CODED "3" (A1 AND A2 CRITERIA COUNT AS ONE).	0
	AT LEAST THREE B SYMPTOMS ARE CODED "3" (FOUR IF MOOD ONLY IRRITABLE)	false
	Note: DSM-IV Criterion C for Mania (i.e., does not meet criteria for a Mixed Episode) has been omitted from the SCID	

C. (Mania)

IF UNKNOWN: At that time, did you have serious problems at home or at work (school) because you were (SYMPTOMS) or did you have to be admitted to a hospital?<br><br>IF YES, SPECIFY:	C. The mood disturbance is sufficiently severe to cause marked impairment in occupational functioning or in usual social activities or relationships with others, or to necessitate hospitalization to prevent harm to self or others, or there are psychotic features.	O O<br>1 3

C. (Hypomania)

IF UNKNOWN: Is this very different from the way you usually are? (How were you different? At work? With friends?) IF YES, Specify:	C. The episode is associated with an unequivocal change in functioning that is uncharacteristic of the person when not symptomatic	O O<br>1 3

D. (Hypomania)

IF UNKNOWN: Did other people notice the change in you? (What did they say?)	D. The disturbance in mood and the change in functioning are observable by others	O O<br>1 3

E. (Hypomania)

IF UNKNOWN: At that time, did you have serious problems at home or at work (school) because you were (SYMPTOMS) or did you have to be admitted to a hospital?	E. The episode is not severe enough to cause marked impairment in social or occupational functioning, or to necessitate hospitalization, and there are no psychotic features	O O<br>1 3

D. (Mania)
F. (Hypomania)

Just before this began, were you physically ill?<br><br>Just before this began, were you drinking or taking any street drugs?	D/F. Not due to the direct physiological effects of a substance (e.g., a drug of abuse, medication) or to a general medical condition<br><br>IF GENERAL MEDICAL CONDITION OR	O O<br>1 3

IF YES: Any change in the amount you were taking?<br><br>Just before this began, were you taking any medications ,other than antidepressants?<br><br>IF YES: Any change in the amount you were taking?	SUBSTANCE THAT CAN BE ETIOLOGICALLY ASSOCIATED WITH MANIA/HYPOMANIA, GO TO *GMC/SUBSTANCE,* A.51 AND RETURN HERE TO MAKE RATING OF "1" OR "3"<br><br>Etiological general medical conditions include: degenerative neurological illnesses (e.g., Huntington's disease, multiple sclerosis), cerebrovascular disease (e.g., stroke), metabolic conditions (e.g., Vitamin B-12 deficiency, Wilson's disease), endocrine conditions (e.g., hyperthyroidism), viral or other infections, and certain cancers (e.g., cerebral neoplasms).<br><br>Etiological substances include: alcohol, amphetamines, cocaine, hallucinogens, inhalants, opioids, phencyclidine, sedatives, hypnotics, and anxiolytics. Medications include psychotropic medications (e.g., anxiolytics), corticosteroids, anabolic steroids, isoniazid, antiparkinson medication (e.g., levodopa), and sympathomimetics/decongestants	
Were you on antidepressant treatment when this episode began?	EPISODE PRECIPITATED BY SOMATIC ANTIDEPRESSANT TREATMENT (BEGAN WITHIN TWO MONTHS OF STARTING OR CHANGING ANTIDEPRESSANT TREATMENT). CODE "3" IF APPLICABLE TO EPISODE<br><br>IF YES RECORD IN MEDICATION SECTION OF OVERVIEW<br><br>NOTE: FOR THE GENETICS STUDY AND IN DISTINCTION TO DSM IV, MANIC AND HYPOMANIC EPISODES THAT ARE CLEARLY PRECIPITATED BY SOMATIC ANTIDEPRESS-ANT TREATMENT (E.G., MEDICATION, ELECTROCONVULSIVE THERAPY, LIGHT THERAPY, SLEEP DEPRIVATION, HERBAL TREATMENTS) DO COUNT TOWARD A DIAGNOSIS OF BIPOLAR DISORDER	O O<br>1 3
IF YES, How long were you on it?	RECORD NUMBER OF WEEKS	
	MANIC EPISODE CRITERIA A, B, C AND D ARE CODED "3"	false
	HYPOMANIC EPISODE CRITERIA A, B, C, D, E, AND F ARE CODED "3"	false
	IF NO MANIC OR HYPOMANIC OR MIXED	

EPISODES, GO TO DYSTHYMIC DISORDER. HOWEVER, IF CODED FOR A MIXED EPISODE IN THE DEPRESSION SECTION, SKIP TO SUMMARY QUESTIONS FOR MIXED EPISODE.

FIRST AND WORST MANIA/HYPOMANIA:

IF UNCLEAR:<br>Is this your worst episode of depression?	DETERMINE WHETHER OR NOT EACH EPISODE IS THE FIRST AND/OR THE WORST MANIA/HYPOMANIA. CODE "3" FOR ONLY ONE FIRST AND ONE WORST EPISODE. THEY MAY BE THE SAME EPISODE AND MAY NOT ALWAYS BE CODED IN B OR C (I.E., D OR E MAY ACTUALLY BE EARLIER OR MORE SEVERE ONCE RECALLED).	O O<br>1 3
Is this the first episode?		O O<br>1 3

MIXED STATE

ASK THE FOLLOWING QUESTIONS TO DETERMINE WHETHER A MIXED STATE WAS PRESENT FOR EACH EPISODE OF MANIA/HYPOMANIA.

During this episode of (MANIA/HYPOMANIA)did you have a week or more during which your mood changed between elation, irritability, or sadness?<br><br>IF YES, SPECIFY:		O O O O<br>? 1 2 3

During this episode of (MANIA/HYPOMANIA) did you also experience any of the following symptoms?

Diminished desire for food or marked overeating?		O O O<br>? 1 3
Inability to sleep when sleep was desired, or excessive sleep?	[NOTE: DIFFERENTIATE BETWEEN LACK OF SLEEP DUE TO MANIA]	O O O<br>? 1 3
Feeling slowed down?		O O O<br>? 1 3
Having fatigue or loss of energy?		O O O<br>? 1 3

Losing interest in pleasurable activities? O O O
? 1 3

Feeling guilty or worthless? O O O
? 1 3

Being unable to think or retain written information? [NOTE: DIFFERENTIATE BETWEEN RACING THOUGHTS/DISTRACTIBILITY DUE TO MANIA] O O O
? 1 3

Were things so bad you were thinking a lot about death or that you would be better off dead? What about thinking of hurting yourself? O O O
? 1 3

IF YES: Did you do anything to hurt yourself?

Check if:

O thoughts of own death

O suicidal ideation

O specific plan

O actual attempt

INTERVIEWER: ENTER NUMBER OF "3" RESPONSES FOR THE NINE MIXED STATE SYMPTOMS (EXCLUDE ABOVE THREE SUCIDALITY QUESTIONS) 0

How long were these symptoms present? ENTER NUMBER OF DAYS

CRITERIA WERE MET SIMULTANEOUSLY FOR BOTH MAJOR DEPRESSION AND MANIA. O O
1 3

TWO TO FOUR CRITERIA WERE MET FOR DEPRESSION O O
1 3

[PROBE FOR PSYCHOTIC SYMPOTMS IN EACH CODED EPISODE. INCLUDE MIXED STATES IN NEXT SECTION]

Probe for Psychotic Symptoms: Per Episode

During this episode of (MANIA) did you have any beliefs or ideas that you later found out were not true? (Like believing that you had powers and abilities others did not have? Or that you had a special mission, perhaps from God? Or that

IF DELUSIONS OR HALLUCINATIONS ARE SUSPECTED, PROBE FURTHER TO DETERMINE THE CONTENT AND WHETHER THE BELIEFS WERE HELD WITH CERTAINTY. [NOTE: IF PSYCHOTIC SYMPTOMS ARE

O O O
? 1 3

someone was trying to harm you? How certain were you?)
IF YES, DESCRIBE:

PRESENT DURING PREVIOUSLY CODED HYPOMANIA, IT SHOULD BE RECODED AS FULL MANIA.]

Did you see or hear things other people could not see or hear?
IF YES, DESCRIBE:

IF YES, PLEASE CHECK:

O Delusions

O Hallucinations

END OF EPISODE SPECIFIC QUESTIONS. CONTINUE WITH NEXT CODED EPISODE.

AFTER REVIEWING ALL NECESSARY EPISODES, CONTINUE BELOW.

Manic and Hypomanic Episode D

Date of Onset	Age	Date of Offset	Duration (days)

Now I'd like to ask you more specific questions about (TIME PERIOD FOR SUSPECTED MANIC OR HYPOMANIC EPISODE).

MANIC EPISODE CRITERIA

A1. (Mania and Hypomania)

During (TIME PERIOD FOR EPISODE) were you feeling so good or hyper that other people thought you were not your normal self or you were so hyper that you got into trouble? (Did anyone say you were manic? Was that more than just feeling good?)

IF NO: What about feeling so irritable that you found yourself shouting at people or starting fights or arguments? Did you find yourself shouting at people you really didn't know?

A(1) A distinct period of abnormally and persistently ("sustained" if hypomania) elevated, expansive, or irritable mood.

O ? O 1 O 2 O 3

What was it like?

CHECK ONE:

O elevated/expansive mood

O irritable mood

Select if this is a manic or hypomanic episoide

O Manic O Hypomanic

A2. (Mania)

How long did that last? (As long as one week? Did you have to go to the hospital?)

A(2) Episode lasted at least one week (any duration if hospitalization is necessary, psychosis is present, or very dangerous behaviors are present)

O 1 O 3

Did it last for at least two days?	Brief Mania<br>(2 day duration required)<br>PER KELSOE CONVENTION, BRIEF MANIA WILL BE INCLUDED IN THE MANIA ASSESSMENT, SPECIFIC PATTERNS.	O O<br>1 3

A2. (Hypomania)

Did it last for at least four days?<br><br>What was that like?	A(2) Episode lasted throughout at least 4 days, and is clearly different from the usual non-depressed mood	O O<br>1 3
What was it like?	CHECK ONE:	O elevated/expansive mood<br><br>O irritable mood

Did it last for at least two days?	Brief Hypomania<br>(2 day duration required)<br><br>PER KELSOE CONVENTION, BRIEF HYPOMANIA WILL BE INCLUDED IN THE HYPOMANIA ASSESSMENT	O O<br>1 3

FOR ALL SUBJECTS, CONTINUE ASKING ABOUT ALL SYMPTOMS, EVEN IF A(1) AND (2) ARE NOT ENDORSED

NOTE: WHEN RATING THE FOLLOWING ITEMS, CODE "1" IF CLEARLY DUE TO A GENERAL MEDICAL CONDITION, SUBSTANCE, OR TO MOOD-INCONGRUENT DELUSIONS OR HALLUCINATIONS. TO COUNT TOWARD A MANIC EPISODE, A SYMPTOM MUST EITHER BE NEWLY PRESENT OR MUST HAVE CLEARLY WORSENED COMPARED WITH THE PERSON'S PRE-EPISODE STATUS.

B. (Mania and Hypomania)

I would like you to focus on the most extreme period of feeling (OWN EQUIVALENT FOR EUPHORIA OR IRRITABILITY), when answering the following questions. During (TIME PERIOD OF EPISODE)	B. During the worst period of the mood disturbance, three (or more) of the following symptoms have persisted (four if the mood is only irritable) and have been present to a significant degree:	
...how did you feel about yourself? (More self-confident than usual? Any special powers or abilities?)	(1) inflated self-esteem or grandiosity	O O O O<br>? 1 2 3

Did you need less sleep than usual?<br><br>IF YES: Did you still feel rested?	(2) decreased need for sleep (e.g., feels rested after missing at least two hours of sleep) NOTE: THIS ITEM SHOULD BE PRESENTED AT EVERY CONSENSUS TO HELP ENSURE RELIABILITY.	O O O O<br>? 1 2 3

Were you much more talkative than usual? (Did people have trouble stopping you or understanding you? Did people have trouble getting a word in edgewise?)	(3) more talkative than usual or pressure to keep talking	O O O O<br>? 1 2 3

Were your thoughts racing through your head?	(4) flight of ideas or subjective experience that thoughts are racing	O ? O 1 O 2 O 3

Were you so easily distracted by things around you that you had trouble concentrating or staying on one track?	(5) distractibility (i.e., attention too easily drawn to unimportant or irrelevant external stimuli)	O ? O 1 O 2 O 3

How did you spend your time? (Work, friends, hobbies? Were you so active that your friends or family were concerned about you?)<br><br>IF NO INCREASED ACTIVITY: Were you physically restless? How bad was it?	(6) increase in goal-directed activity (either socially, at work or school, or sexually) or psychomotor agitation	O ? O 1 O 2 O 3
	Check if:	O psychomotor agitation<br><br>O increase in activity

Did you do anything that could have caused trouble for you or your family? (Buying things you didn't need? Anything sexual that was unusual for you? Reckless driving?)	(7) excessive involvement in pleasurable activities which have a high potential for painful consequences (e.g., engaging in unrestrained buying sprees, sexual indiscretions, or foolish business investments)	O ? O 1 O 2 O 3

NUMBER OF MANIC/HYPOMANIC SYMPTOMS IN A AND B CODED "3" (A1 AND A2 CRITERIA COUNT AS ONE). 0

AT LEAST THREE B SYMPTOMS ARE CODED "3" (FOUR IF MOOD ONLY IRRITABLE) false

Note: DSM-IV Criterion C for Mania (i.e., does not meet criteria for a Mixed Episode) has been omitted from the SCID

C. (Mania)

IF UNKNOWN: At that time, did you have serious problems at home or at work (school) because you were (SYMPTOMS) or did you have to be admitted to a hospital?<br><br>IF YES, SPECIFY:	C. The mood disturbance is sufficiently severe to cause marked impairment in occupational functioning or in usual social activities or relationships with others, or to necessitate hospitalization to prevent harm to self or others, or there are psychotic features.	O 1 O 3

C. (Hypomania)

IF UNKNOWN: Is this very different from the way you usually are? (How were you different? At work?	C. The episode is associated with an unequivocal change in functioning that is uncharacteristic of the	O 1 O 3

With friends?) IF YES, Specify:	person when not symptomatic	

D. (Hypomania)

IF UNKNOWN: Did other people notice the change in you? (What did they say?)	D. The disturbance in mood and the change in functioning are observable by others	O O<br>1 3

E. (Hypomania)

IF UNKNOWN: At that time, did you have serious problems at home or at work (school) because you were (SYMPTOMS) or did you have to be admitted to a hospital?	E. The episode is not severe enough to cause marked impairment in social or occupational functioning, or to necessitate hospitalization, and there are no psychotic features	O O<br>1 3

D. (Mania)
F. (Hypomania)

Just before this began, were you physically ill?<br><br>Just before this began, were you drinking or taking any street drugs?<br><br>IF YES: Any change in the amount you were taking?<br><br>Just before this began, were you taking any medications ,other than antidepressants?<br><br>IF YES: Any change in the amount you were taking?	D/F. Not due to the direct physiological effects of a substance (e.g., a drug of abuse, medication) or to a general medical condition<br><br>IF GENERAL MEDICAL CONDITION OR SUBSTANCE THAT CAN BE ETIOLOGICALLY ASSOCIATED WITH MANIA/HYPOMANIA, GO TO *GMC/SUBSTANCE,* A.51 AND RETURN HERE TO MAKE RATING OF "1" OR "3"<br><br>Etiological general medical conditions include: degenerative neurological illnesses (e.g., Huntington's disease, multiple sclerosis), cerebrovascular disease (e.g., stroke), metabolic conditions (e.g., Vitamin B-12 deficiency, Wilson's disease), endocrine conditions (e.g., hyperthyroidism), viral or other infections, and certain cancers (e.g., cerebral neoplasms).<br><br>Etiological substances include: alcohol, amphetamines, cocaine, hallucinogens, inhalants, opioids, phencyclidine, sedatives, hypnotics, and anxiolytics. Medications include psychotropic medications (e.g., anxiolytics), corticosteroids, anabolic steroids, isoniazid, antiparkinson medication (e.g., levodopa), and sympathomimetics/decongestants	O O<br>1 3

Were you on antidepressant treatment when this episode began?	EPISODE PRECIPITATED BY SOMATIC ANTIDEPRESSANT TREATMENT (BEGAN WITHIN TWO MONTHS OF STARTING OR CHANGING ANTIDEPRESSANT TREATMENT). CODE "3" IF APPLICABLE TO EPISODE	O O<br>1 3

IF YES RECORD IN MEDICATION SECTION OF OVERVIEW

NOTE: FOR THE GENETICS STUDY AND IN DISTINCTION TO DSM IV, MANIC AND HYPOMANIC EPISODES THAT ARE CLEARLY PRECIPITATED BY SOMATIC ANTIDEPRESS-ANT TREATMENT (E.G., MEDICATION, ELECTROCONVULSIVE THERAPY, LIGHT THERAPY, SLEEP DEPRIVATION, HERBAL TREATMENTS) DO COUNT TOWARD A DIAGNOSIS OF BIPOLAR DISORDER

IF YES, How long were you on it?

RECORD NUMBER OF WEEKS

MANIC EPISODE CRITERIA A, B, C AND D ARE CODED "3"

false

HYPOMANIC EPISODE CRITERIA A, B, C, D, E, AND F ARE CODED "3"

false

IF NO MANIC OR HYPOMANIC OR MIXED EPISODES, GO TO DYSTHYMIC DISORDER. HOWEVER, IF CODED FOR A MIXED EPISODE IN THE DEPRESSION SECTION, SKIP TO SUMMARY QUESTIONS FOR MIXED EPISODE.

FIRST AND WORST MANIA/HYPOMANIA:

IF UNCLEAR:
Is this your worst episode of depression?

DETERMINE WHETHER OR NOT EACH EPISODE IS THE FIRST AND/OR THE WORST MANIA/HYPOMANIA. CODE "3" FOR ONLY ONE FIRST AND ONE WORST EPISODE. THEY MAY BE THE SAME EPISODE AND MAY NOT ALWAYS BE CODED IN B OR C (I.E., D OR E MAY ACTUALLY BE EARLIER OR MORE SEVERE ONCE RECALLED).

O O
1 3

Is this the first episode?

O O
1 3

MIXED STATE

ASK THE FOLLOWING QUESTIONS TO DETERMINE WHETHER A MIXED STATE WAS PRESENT FOR EACH EPISODE OF MANIA/HYPOMANIA.

During this episode of (MANIA/HYPOMANIA)did you have a week or more during which your mood changed between elation, irritability, or sadness?

O O O O
? 1 2 3

IF YES, SPECIFY:

During this episode of (MANIA/HYPOMANIA) did you also experience any of the following symptoms?

Diminished desire for food or marked overeating? O ? O 1 O 3

Inability to sleep when sleep was desired, or excessive sleep? [NOTE: DIFFERENTIATE BETWEEN LACK OF SLEEP DUE TO MANIA] O ? O 1 O 3

Feeling slowed down? O ? O 1 O 3

Having fatigue or loss of energy? O ? O 1 O 3

Losing interest in pleasurable activities? O ? O 1 O 3

Feeling guilty or worthless? O ? O 1 O 3

Being unable to think or retain written information? [NOTE: DIFFERENTIATE BETWEEN RACING THOUGHTS/DISTRACTIBILITY DUE TO MANIA] O ? O 1 O 3

Were things so bad you were thinking a lot about death or that you would be better off dead? What about thinking of hurting yourself? O ? O 1 O 3

IF YES: Did you do anything to hurt yourself?

	Check if:	O thoughts of own death<br>O suicidal ideation<br>O specific plan<br>O actual attempt
	INTERVIEWER: ENTER NUMBER OF "3" RESPONSES FOR THE NINE MIXED STATE SYMPTOMS (EXCLUDE ABOVE THREE SUCIDALITY QUESTIONS)	0
How long were these symptoms present?	ENTER NUMBER OF DAYS	
	CRITERIA WERE MET SIMULTANEOUSLY FOR BOTH MAJOR DEPRESSION AND MANIA.	O 1 O 3
	TWO TO FOUR CRITERIA WERE MET FOR DEPRESSION	O 1 O 3
[PROBE FOR PSYCHOTIC SYMPOTMS IN EACH CODED EPISODE. INCLUDE MIXED STATES IN NEXT SECTION]	**Probe for Psychotic Symptoms: Per Episode**	
During this episode of (MANIA) did you have any beliefs or ideas that you later found out were not true? (Like believing that you had powers and abilities others did not have? Or that you had a special mission, perhaps from God? Or that someone was trying to harm you? How certain were you?)<br>IF YES, DESCRIBE:<br><br>Did you see or hear things other people could not see or hear?<br>IF YES, DESCRIBE:	IF DELUSIONS OR HALLUCINATIONS ARE SUSPECTED, PROBE FURTHER TO DETERMINE THE CONTENT AND WHETHER THE BELIEFS WERE HELD WITH CERTAINTY. [NOTE: IF PSYCHOTIC SYMPTOMS ARE PRESENT DURING PREVIOUSLY CODED HYPOMANIA, IT SHOULD BE RECODED AS FULL MANIA.]	O ? O 1 O 3
	IF YES, PLEASE CHECK:	O Delusions<br>O Hallucinations

END OF EPISODE SPECIFIC QUESTIONS. CONTINUE WITH NEXT CODED EPISODE.

AFTER REVIEWING ALL NECESSARY EPISODES, CONTINUE BELOW.

Manic and Hypomanic Episode E

Date of Onset	Age	Date of Offset	Duration (days)

Now I'd like to ask you more specific questions about (TIME PERIOD FOR SUSPECTED MANIC OR HYPOMANIC EPISODE).	**MANIC EPISODE CRITERIA**	
**A1. (Mania and Hypomania)**		
During (TIME PERIOD FOR EPISODE) were you	A(1) A distinct period of abnormally and	O O O O

feeling so good or hyper that other people thought you were not your normal self or you were so hyper that you got into trouble? (Did anyone say you were manic? Was that more than just feeling good?)<br><br>IF NO: What about feeling so irritable that you found yourself shouting at people or starting fights or arguments? Did you find yourself shouting at people you really didn't know?	persistently ("sustained" if hypomania) elevated, expansive, or irritable mood.	? 1 2 3
What was it like?	CHECK ONE:	O elevated/expansive mood<br><br>O irritable mood

	Select if this is a manic or hypomanic episoide	O Manic O Hypomanic

A2. (Mania)

How long did that last? (As long as one week? Did you have to go to the hospital?)	A(2) Episode lasted at least one week (any duration if hospitalization is necessary, psychosis is present, or very dangerous behaviors are present)	O 1 O 3

Did it last for at least two days?	Brief Mania<br>(2 day duration required)<br>PER KELSOE CONVENTION, BRIEF MANIA WILL BE INCLUDED IN THE MANIA ASSESSMENT, SPECIFIC PATTERNS.	O 1 O 3

A2. (Hypomania)

Did it last for at least four days?<br><br>What was that like?	A(2) Episode lasted throughout at least 4 days, and is clearly different from the usual non-depressed mood	O 1 O 3
What was it like?	CHECK ONE:	O elevated/expansive mood<br><br>O irritable mood

Did it last for at least two days?	Brief Hypomania<br>(2 day duration required)<br><br>PER KELSOE CONVENTION, BRIEF HYPOMANIA WILL BE INCLUDED IN THE HYPOMANIA ASSESSMENT	O 1 O 3

FOR ALL SUBJECTS, CONTINUE ASKING ABOUT ALL SYMPTOMS, EVEN IF A(1) AND (2) ARE NOT ENDORSED

NOTE: WHEN RATING THE FOLLOWING ITEMS, CODE "1" IF CLEARLY DUE TO A GENERAL MEDICAL CONDITION, SUBSTANCE, OR TO MOOD-INCONGRUENT DELUSIONS OR HALLUCINATIONS. TO COUNT TOWARD A MANIC EPISODE, A SYMPTOM MUST EITHER BE NEWLY PRESENT OR MUST HAVE CLEARLY WORSENED COMPARED WITH THE PERSON'S PRE-EPISODE STATUS.

B. (Mania and Hypomania)

I would like you to focus on the most extreme period of feeling (OWN EQUIVALENT FOR EUPHORIA OR IRRITABILITY), when answering the following questions. During (TIME PERIOD OF EPISODE)	B. During the worst period of the mood disturbance, three (or more) of the following symptoms have persisted (four if the mood is only irritable) and have been present to a significant degree:	
...how did you feel about yourself? (More self-confident than usual? Any special powers or abilities?)	(1) inflated self-esteem or grandiosity	O O O O<br>? 1 2 3
Did you need less sleep than usual?<br><br>IF YES: Did you still feel rested?	(2) decreased need for sleep (e.g., feels rested after missing at least two hours of sleep) NOTE: THIS ITEM SHOULD BE PRESENTED AT EVERY CONSENSUS TO HELP ENSURE RELIABILITY.	O O O O<br>? 1 2 3
Were you much more talkative than usual? (Did people have trouble stopping you or understanding you? Did people have trouble getting a word in edgewise?)	(3) more talkative than usual or pressure to keep talking	O O O O<br>? 1 2 3
Were your thoughts racing through your head?	(4) flight of ideas or subjective experience that thoughts are racing	O O O O<br>? 1 2 3
Were you so easily distracted by things around you that you had trouble concentrating or staying on one track?	(5) distractibility (i.e., attention too easily drawn to unimportant or irrelevant external stimuli)	O O O O<br>? 1 2 3
How did you spend your time? (Work, friends, hobbies? Were you so active that your friends or family were concerned about you?)<br><br>IF NO INCREASED ACTIVITY: Were you physically restless? How bad was it?	(6) increase in goal-directed activity (either socially, at work or school, or sexually) or psychomotor agitation<br><br>Check if:	O O O O<br>? 1 2 3<br><br>O psychomotor agitation<br>O increase in activity
Did you do anything that could have caused trouble for you or your family? (Buying things you didn't	(7) excessive involvement in pleasurable activities which have a high potential for painful	O O O O<br>? 1 2 3

need? Anything sexual that was unusual for you? Reckless driving?)	consequences (e.g., engaging in unrestrained buying sprees, sexual indiscretions, or foolish business investments)	

	NUMBER OF MANIC/HYPOMANIC SYMPTOMS IN A AND B CODED "3" (A1 AND A2 CRITERIA COUNT AS ONE).	0
	AT LEAST THREE B SYMPTOMS ARE CODED "3" (FOUR IF MOOD ONLY IRRITABLE)	false
	Note: DSM-IV Criterion C for Mania (i.e., does not meet criteria for a Mixed Episode) has been omitted from the SCID	

C. (Mania)

IF UNKNOWN: At that time, did you have serious problems at home or at work (school) because you were (SYMPTOMS) or did you have to be admitted to a hospital?<br><br>IF YES, SPECIFY:	C. The mood disturbance is sufficiently severe to cause marked impairment in occupational functioning or in usual social activities or relationships with others, or to necessitate hospitalization to prevent harm to self or others, or there are psychotic features.	O O<br>1 3

C. (Hypomania)

IF UNKNOWN: Is this very different from the way you usually are? (How were you different? At work? With friends?) IF YES, Specify:	C. The episode is associated with an unequivocal change in functioning that is uncharacteristic of the person when not symptomatic	O O<br>1 3

D. (Hypomania)

IF UNKNOWN: Did other people notice the change in you? (What did they say?)	D. The disturbance in mood and the change in functioning are observable by others	O O<br>1 3

E. (Hypomania)

IF UNKNOWN: At that time, did you have serious problems at home or at work (school) because you were (SYMPTOMS) or did you have to be admitted to a hospital?	E. The episode is not severe enough to cause marked impairment in social or occupational functioning, or to necessitate hospitalization, and there are no psychotic features	O O<br>1 3

D. (Mania)
F. (Hypomania)

Just before this began, were you physically ill?<br><br>Just before this began, were you drinking or taking any street drugs?	D/F. Not due to the direct physiological effects of a substance (e.g., a drug of abuse, medication) or to a general medical condition<br><br>IF GENERAL MEDICAL CONDITION OR	O O<br>1 3

IF YES: Any change in the amount you were taking?<br><br>Just before this began, were you taking any medications ,other than antidepressants?<br><br>IF YES: Any change in the amount you were taking?	SUBSTANCE THAT CAN BE ETIOLOGICALLY ASSOCIATED WITH MANIA/HYPOMANIA, GO TO *GMC/SUBSTANCE,* A.51 AND RETURN HERE TO MAKE RATING OF "1" OR "3"<br><br>Etiological general medical conditions include: degenerative neurological illnesses (e.g., Huntington's disease, multiple sclerosis), cerebrovascular disease (e.g., stroke), metabolic conditions (e.g., Vitamin B-12 deficiency, Wilson's disease), endocrine conditions (e.g., hyperthyroidism), viral or other infections, and certain cancers (e.g., cerebral neoplasms).<br><br>Etiological substances include: alcohol, amphetamines, cocaine, hallucinogens, inhalants, opioids, phencyclidine, sedatives, hypnotics, and anxiolytics. Medications include psychotropic medications (e.g., anxiolytics), corticosteroids, anabolic steroids, isoniazid, antiparkinson medication (e.g., levodopa), and sympathomimetics/decongestants	
Were you on antidepressant treatment when this episode began?	EPISODE PRECIPITATED BY SOMATIC ANTIDEPRESSANT TREATMENT (BEGAN WITHIN TWO MONTHS OF STARTING OR CHANGING ANTIDEPRESSANT TREATMENT). CODE "3" IF APPLICABLE TO EPISODE<br><br>IF YES RECORD IN MEDICATION SECTION OF OVERVIEW<br><br>NOTE: FOR THE GENETICS STUDY AND IN DISTINCTION TO DSM IV, MANIC AND HYPOMANIC EPISODES THAT ARE CLEARLY PRECIPITATED BY SOMATIC ANTIDEPRESS-ANT TREATMENT (E.G., MEDICATION, ELECTROCONVULSIVE THERAPY, LIGHT THERAPY, SLEEP DEPRIVATION, HERBAL TREATMENTS) DO COUNT TOWARD A DIAGNOSIS OF BIPOLAR DISORDER	O 1 O 3
IF YES, How long were you on it?	RECORD NUMBER OF WEEKS	
	MANIC EPISODE CRITERIA A, B, C AND D ARE CODED "3"	false
	HYPOMANIC EPISODE CRITERIA A, B, C, D, E, AND F ARE CODED "3"	false
	IF NO MANIC OR HYPOMANIC OR MIXED	

EPISODES, GO TO DYSTHYMIC DISORDER. HOWEVER, IF CODED FOR A MIXED EPISODE IN THE DEPRESSION SECTION, SKIP TO SUMMARY QUESTIONS FOR MIXED EPISODE.

FIRST AND WORST MANIA/HYPOMANIA:

IF UNCLEAR:<br>Is this your worst episode of depression?	DETERMINE WHETHER OR NOT EACH EPISODE IS THE FIRST AND/OR THE WORST MANIA/HYPOMANIA. CODE "3" FOR ONLY ONE FIRST AND ONE WORST EPISODE. THEY MAY BE THE SAME EPISODE AND MAY NOT ALWAYS BE CODED IN B OR C (I.E., D OR E MAY ACTUALLY BE EARLIER OR MORE SEVERE ONCE RECALLED).	O O<br>1 3
Is this the first episode?		O O<br>1 3

MIXED STATE

ASK THE FOLLOWING QUESTIONS TO DETERMINE WHETHER A MIXED STATE WAS PRESENT FOR EACH EPISODE OF MANIA/HYPOMANIA.

During this episode of (MANIA/HYPOMANIA)did you have a week or more during which your mood changed between elation, irritability, or sadness?<br><br>IF YES, SPECIFY:		O O O O<br>? 1 2 3

During this episode of (MANIA/HYPOMANIA) did you also experience any of the following symptoms?

Diminished desire for food or marked overeating?		O O O<br>? 1 3
Inability to sleep when sleep was desired, or excessive sleep?	[NOTE: DIFFERENTIATE BETWEEN LACK OF SLEEP DUE TO MANIA]	O O O<br>? 1 3
Feeling slowed down?		O O O<br>? 1 3
Having fatigue or loss of energy?		O O O<br>? 1 3

Losing interest in pleasurable activities? O O O ? 1 3

Feeling guilty or worthless? O O O ? 1 3

Being unable to think or retain written information? [NOTE: DIFFERENTIATE BETWEEN RACING THOUGHTS/DISTRACTIBILITY DUE TO MANIA] O O O ? 1 3

Were things so bad you were thinking a lot about death or that you would be better off dead? What about thinking of hurting yourself? O O O ? 1 3

IF YES: Did you do anything to hurt yourself?

Check if:

O thoughts of own death

O suicidal ideation

O specific plan

O actual attempt

INTERVIEWER: ENTER NUMBER OF "3" RESPONSES FOR THE NINE MIXED STATE SYMPTOMS (EXCLUDE ABOVE THREE SUCIDALITY QUESTIONS) 0

How long were these symptoms present? ENTER NUMBER OF DAYS

CRITERIA WERE MET SIMULTANEOUSLY FOR BOTH MAJOR DEPRESSION AND MANIA. O O 1 3

TWO TO FOUR CRITERIA WERE MET FOR DEPRESSION O O 1 3

[PROBE FOR PSYCHOTIC SYMPOTMS IN EACH CODED EPISODE. INCLUDE MIXED STATES IN NEXT SECTION]

Probe for Psychotic Symptoms: Per Episode

During this episode of (MANIA) did you have any beliefs or ideas that you later found out were not true? (Like believing that you had powers and abilities others did not have? Or that you had a special mission, perhaps from God? Or that

IF DELUSIONS OR HALLUCINATIONS ARE SUSPECTED, PROBE FURTHER TO DETERMINE THE CONTENT AND WHETHER THE BELIEFS WERE HELD WITH CERTAINTY. [NOTE: IF PSYCHOTIC SYMPTOMS ARE

O O O ? 1 3

someone was trying to harm you? How certain were you?)<br>IF YES, DESCRIBE:<br><br>Did you see or hear things other people could not see or hear?<br>IF YES, DESCRIBE:	PRESENT DURING PREVIOUSLY CODED HYPOMANIA, IT SHOULD BE RECODED AS FULL MANIA.]	
	IF YES, PLEASE CHECK:	O Delusions<br>O Hallucinations

END OF EPISODE SPECIFIC QUESTIONS. CONTINUE WITH NEXT CODED EPISODE.

AFTER REVIEWING ALL NECESSARY EPISODES, CONTINUE BELOW.

Psychosis

	**Lifetime Probe for Psychotic Symptoms in Mania**	
Have you ever had either of these experiences (DELUSIONS/HALLUCINATIONS) during any other periods of (MANIA)?	IF DELUSIONS OR HALLUCINATIONS ARE SUSPECTED, PROBE FURTHER TO DETERMINE THE CONTENT AND WHETHER THE BELIEFS WERE HELD WITH CERTAINTY.<br>IF YES, DESCRIBE:	O ? O 1 O 3
IF YES, PLEASE CHECK:		O Delusions<br>O Hallucinations

When you had experiences like this, were these topics and themes typical of your manic states?	DETERMINE WHETHER THE DELUSIONS OR HALLUCINATIONS WERE CHARACTERISTICALLY MOOD CONGRUENT OR INCONGRUENT DURING MANIAS OVER THE LIFE SPAN	O Mood Congruent<br>O Mood Incongruent<br>O N/A<br>O Unknown<br>O No Information

	DETERMINE WHETHER OR NOT DELUSIONS WERE BIZARRE (IF UNSURE, SEE B.3 AND RETURN HERE TO CODE)	O Bizarre Delusions<br>O Non-Bizarre Delusions<br>O N/A<br>O Unknown

Do you usually have experiences like this	DETERMINE WHETHER OR NOT	O Typical

(DELUSIONS/HALLUCINATIONS) when you have periods of mania?	PSYCHOTIC SYMPTOMS ARE TYPICAL OF MOST EPISODES OF MANIA	O Not Typical<br>O N/A<br>O Unknown

Lifetime Probe for Psychotic Symptoms for Mixed States

When you had experiences like this, were these topics and themes typical of your mixed states?	DETERMINE WHETHER THE DELUSIONS OR HALLUCINATIONS WERE CHARACTERISTICALLY MOOD CONGRUENT OR INCONGRUENT OVER THE LIFE SPAN	O Mood Congruent<br>O Mood Incongruent<br>O N/A<br>O Unknown<br>O No Information

	DETERMINE WHETHER OR NOT DELUSIONS WERE BIZARRE (IF UNSURE, SEE B.3 AND RETURN HERE TO CODE	O Bizarre Delusions<br>O Non-Bizarre Delusions<br>O N/A<br>O Unknown

Do you usually have experiences like this (DELUSIONS/ HALLUCINA-TIONS) when you have periods of mixed state?	DETERMINE WHETHER OR NOT PSYCHOTIC SYMPTOMS ARE TYPICAL OF MOST EPISODES OF MIXED STATE	O Typical<br>O Not Typical<br>O N/A<br>O Unknown

GENERAL MANIA/HYPOMANIA/MIXED STATE: CLINICAL DATA

PLEASE ANSWER ONLY APPLICABLE QUESTIONS (I.E., SKIP ALL MANIA QUESTIONS IF NEVER ENDORSED).

Now I would like to ask you some general questions about (MANIC/HYPOMANIC/MIXED STATE/OWN EQUIVALENT) episodes and symptoms.

Do your periods of illness typically begin with mania or depression?	PATTERN OF ILLNESS:<br>Select one:	O Mania(hypo)precedes depression<br>O Depressionprecedes mania(hypo)<br>O Undetermined<br>O Unknown<br>O N/A<br>O No Information

Mania:

How many separate times have you been (HIGH / IRRITABLE/ OWN EQUIVALENT) nearly every day for at least a week?	Total number of Manic Episodes (MET CRITERA)	
How old were you when you first had a lot of these symptoms for at least one week?	Age at onset of first unequivocal Manic Episode (MET CRITERIA)	
What is the longest that a mania like this has lasted?	Duration of longest Manic Episode (days) (MET CRITERIA)	
IF THERE ARE NUMEROUS EPISODES: How long do your periods of mania typically last?	Typical duration of Manic Episodes (days)	

Hypomania:

How many separate times have you been (HIGH / IRRITABLE/ OWN EQUIVALENT) nearly every day for at least four days?	Total number of Hypomanic Episodes (MET CRITERIA)[ANY EPISODE >2 DAYS COUNTS]	
IF SUBJECT ENDORSED MANIC EPISODES: Did you ever have a period of time prior to age (AGE IDENTIFIED IN PREVIOUS QUESTION (D4)) when you had a few of these symptoms for at least four days?	PROBE FOR POSSIBLE PRECEDING HYPOMANIC EPISODES.	O ? O 1 O 3

IF YES, OR IF SUBJECT DID NOT ENDORSE MANIC EPISODES:How old were you when you first had a few of these symptoms for at least one week?	Age at onset of first unequivocal Hypomanic Episode (MET CRITERIA)	
What is the longest that a (HYPOMANIA/OWN EQUIVALENT) like this has lasted?	Duration of longest Hypomanic Episode (days) (MET CRITERIA)	
How long do (HIGH/IRRITABLE/ OWN EQUIVALENT) periods typically last?	Typical duration of Hypomanic Episodes (days)	

Mixed States:

How old were you when you first had any symptoms of a mixed episode?	Age of first symptoms of Mixed Episode	
How old were you when you first had a lot of these symptoms for at least one week?	Age at onset of first unequivocal Mixed Episode (MET CRITERIA)	
How many separate times have you been (MIXED/ OWN EQUIVALENT) nearly every day for at least a week?	Total number of Mixed Episodes (MET CRITERIA)	
What is the longest that a (MIXED STATE/OWN EQUIVALENT) like this has lasted?	Duration of longest Mixed Episode (days) (MET CRITERIA)	
How long do mixed periods typically last?	Typical duration of Mixed Episodes (days)	

Rapid Cycling:

How old were you the first time you experienced rapid cycling?	Age at onset of first year of Rapid Cycling [> FOUR OR MORE EPISODES PER YEAR]	
Over your lifetime, how many times have you had periods of rapid cycling? Only once? Several	Overall lifetime course of rapid cycling	O Episodic

separate episode? Or one continuous episode lasting at least 2 years?		O Chronic<br>O Single<br>O Unknown<br>O N/A<br>O No Information
How frequently does your mood change - hourly, daily, weekly, or monthly?	Overall lifetime frequency of mood changes	O Hourly<br>O Daily<br>O Weekly<br>O Monthly<br>O Unknown<br>O N/A<br>O No Information
Is rapid cycling typical of most of your episodes?	Determine whether or not rapid cycling is a typical lifetime pattern	O Typical<br>O Not Typical<br>O N/A<br>O Unknown
	**Summary for Mania/Hypomania/Mixed:**	
How old were you when you first had any symptoms of (MANIA/ HYPOMANIA/OWN EQUIVALENT)?	Age of first symptoms of Mania/ Hypomania	
	Maximum number of manic symptoms endorsed in Criteria A and B (page A24) in a single episode (whether the episode meets criteria or not). Include Mixed if applicable.	
	GAF Ratings: (Click the Help button to display the scale to rate the following)	
	Typical episode of Mania	
	Worst week of most severe episode of Mania	
	Typical episode of Mixed State	
	Worst week of most severe episode of Mixed State	
What portion of your life have you spent with any manic/hypomanic symptoms?	Total Duration of any manic symptoms:	O Not at all (0%)<br>O Rarely (1-19%)<br>O Significant minority (20-39%)<br>O About half the time (40-69%)<br>O Significant majority (70-89%)<br>O Unknown<br>O N/A<br>O No Information

What portion of your life have you spent with any mixed symptoms?

Total Duration of any mixed symptoms:

O Not at all (0%)

O Rarely (1-19%)

O Significant minority (20-39%)

O About half the time (40-69%)

O Significant majority (70-89%)

O Unknown

O N/A

O No Information

MANIC/HYPOMANIC/MIXED EPISODE SPECIFIERS

WITH POSTPARTUM ONSET
FEMALE SUBJECTS ONLY

Have you ever had an episode of (MANIA/HYPOMANIA/MIXED STATE/OWN EQUIVALENT) which started within a month of childbirth?

Has ever had a Manic, Hypomanic or Mixed Episode with onset within 4 weeks postpartum

O ? O 1 O 3

Module A: Other Mood Disorders

DYSTHYMIC DISORDER

(FOR GENETICS STUDIES, SCORE PAST AND CURRENT EPISODES)

IF THERE HAS EVER BEEN A MANIC OR HYPOMANIC EPISODE, CHECK HERE AND GO TO THE NEXT SECTION, CYCLOTHYMIC DISORDER, A44. O Check Here

(CURRENT SYMPTOMS)

IF NO MAJOR DEPRESSIVE EPISODE IN PAST TWO YEARS:
For the past couple of years, have you been bothered by depressed mood most of the day, more days than not? (More than half the time?)

IF YES: What was that like?

DYSTHYMIC DISORDER CRITERIA

A. Depressed mood for most of the day, for more days than not, as indicated either by subjective account or observation made by others, for at least two years. Note: In children and adolescents, mood can be irritable and duration must be at least one year.

O ? O 1 O 2 O 3

IF CURRENT MAJOR DEPRESSIVE EPISODE:
Let's review when you first had most of the symptoms of (CURRENT MAJOR DEPRESSIVE EPISODE). For the two years prior to (BEGINNING DATE), were you bothered by depressed mood, most of the day, more days than not? (More than half the time?)

FIRST MET CRITERIA FOR CURRENT MAJOR DEPRESSIVE EPISODE:

AGE

FOR A PAST MAJOR DEPRESSIVE EPISODE DURING THE PAST TWO YEARS:
Let's review when you first had most of the symptoms of (PAST MAJOR DEPRESSIVE EPISODE) and the point at which you no longer had most of the symptoms. Since the (DATE OF NO LONGER MEETING CRITERIA), have you still been bothered by depressed mood, so that you have been depressed for most of the day, more days than not?

FIRST MET CRITERIA FOR PAST MAJOR DEPRESSIVE EPISODE IN PAST TWO YEARS:

IF YES: For the two years prior to (DATE OF

BEGINNING OF PAST MAJOR DEPRESSIVE EPISODE), were you bothered by depressed mood, most of the day, more days than not? More than half the time?

AGE

NO LONGER MET CRITERIA FOR PAST MAJOR DEPRESSIVE EPISODE IN PAST TWO YEARS:

AGE

(PAST SYMPTOMS)
FOR ALL OTHER SITUATIONS: Have you ever had a period of two years or longer when you have been bothered by a depressed mood most of the day, more days than not? (More than half the time?)

PROBE FOR POSSIBLE PAST DYSTHYMIC EPISODE. ANSWER ABOVE.

During these periods of (OWN WORDS FOR CHRONIC DEPRESSION) do you often…	B. Presence, while depressed, of two (or more) of the following:	
...lose your appetite? What about overeating?	(1) poor appetite or overeating	O ? O 1 O 2 O 3
...have trouble sleeping or sleep too much?	(2) insomnia or hypersomnia	O ? O 1 O 2 O 3
...have little energy to do things or feel tired a lot?	(3) low energy or fatigue	O ? O 1 O 2 O 3
...feel down on yourself? Feel worthless, or like a failure?	(4) low self-esteem	O ? O 1 O 2 O 3
...have trouble concentrating or making decisions?	(5) poor concentration or difficulty making decisions	O ? O 1 O 2 O 3
...feel hopeless?	(6) feelings of hopelessness	O ? O 1 O 2 O 3

AT LEAST TWO "B" SYMPTOMS CODED "3" false

What is the longest period of time, during this period of depression, that you felt okay (NO DYSTHYMIC SYMPTOMS)?	C. During the two year period (one year for children or adolescents) of the disturbance, the person has never been without the symptoms in criteria A and B for more than two months at a	O ? O 1 O 2 O 3

time. [NOTE: CODE "1" IF NORMAL MOOD FOR AT LEAST TWO MONTHS AT A TIME]

How long have/had you been feeling this depression? How old were you the very first time you felt this way for at least two years?

IF COMPLETED CURRENT SECTION ON A37 FOR CRITERION A, USE THAT INFORMATION TO ANSWER THIS QUESTION.

IF A MAJOR DEPRESSIVE EPISODE PRECEDED DYSTHYMIC SYMPTOMS: Now I want to know whether you got completely back to your usual self after that (MAJOR DEPRESSIVE EPISODE) you had (DATE), before this long period of being mildly depressed? (Were you back to your usual self for at least two months?)

D. No Major Depressive Episode during the first two years of the disturbance (one year for children and adolescents); i.e., not better accounted for by chronic Major Depressive Disorder or Major Depressive Disorder in partial remission.

Note: There may have been a previous Major Depressive Episode provided there was a full remission (no significant signs or symptoms for two months) before development of the Dysthymic Disorder. In addition, after the initial two years (one year for children or adolescents) of Dysthymic Disorder, there may be superimposed episodes of Major Depressive Disorder, in which case both diagnoses may be given when the criteria are met for a Major Depressive Episode.

NOTE: CODE "3" IF NO PAST MAJOR DEPRESSIVE EPISODES OR IF MAJOR DEPRESSIVE EPISODES WERE NOT PRESENT DURING THE FIRST TWO YEARS OR IF THERE WAS AT LEAST A TWO MONTH PERIOD WITHOUT SYMPTOMS PRECEDING THE ONSET.

O O O O
? 1 2 3

Age at onset of Dysthymic Disorder

COMPARE ONSET OF DYSTHYMIC SYMPTOMS WITH DATES OF PAST MAJOR DEPRESSIVE EPISODES TO DETERMINE IF THERE WERE ANY MAJOR DEPRESSIVE EPISODES IN FIRST TWO YEARS OF DYSTHYMIC DISORDER.

E. There has never been a Manic, Mixed, or Hypomanic Episode, and the criteria have never been met for Cyclothymic Disorder. If necessary, go to Cyclothymic Disorder page A44 and complete section before returning here to code this item.

O O O O
? 1 2 3

IF NOT ALREADY CLEAR: RETURN TO THIS ITEM AFTER COMPLETING THE PSYCHOTIC DISORDERS SECTION.

F. The disturbance does not occur exclusively during the course of a chronic psychotic disorder, such as Schizophrenia or Delusional Disorder.

O O O O
? 1 2 3

NOTE: CODE "3" IF NO CHRONIC PSYCHOTIC DISORDER OR IF NOT SUPERIMPOSED ON A CHRONIC PSYCHOTIC DISORDER

Just before this began, were you physically ill?
IF YES: What did the doctor say?

Just before this began, were you drinking or taking any street drugs?
IF YES: Any change in the amount you were taking?

Just before this began, were you taking any medications?

IF YES: Any change in the amount you were taking?

G. Not due to the direct physiological effects of a substance (e.g., a drug of abuse, medication) or to a general medical condition

O ? O 1 O 2 O 3

IF THERE IS ANY INDICATION THAT THE DEPRESSION MAY BE SECONDARY (I.E., A DIRECT PHYSIOLOGICAL CONSEQUENCE OF A GMC OR SUBSTANCE), GO TO *GMC/SUBSTANCE,* A.51, AND RETURN HERE TO MAKE A RATING OF "1" OR "3."

Etiological general medical conditions include: degenerative neurological illnesses (e.g., Parkinson's disease, Huntington's disease), cerebrovascular disease, metabolic and endocrine conditions (e.g., B-12 deficiency, hypothyroidism), autoimmune conditions (e.g., systemic lupus erythematosis), viral or other infections (e.g., hepatitis, mononucleosis, HIV), and certain cancers (e.g., carcinoma of the pancreas)

Etiological substances include: alcohol, amphetamines, cocaine, hallucinogens, inhalants, opioids, phencyclidine, sedatives, hypnotics, anxiolytics. Medications include antihypertensives, oral contraceptives, corticosteroids, anabolic steroids, anticancer agents, analgesics, anticholinergics, and cardiac medications.

IF UNCLEAR: How much did your depressed feelings interfere with your life?

H. The symptoms cause clinically significant distress or impairment in social, occupational, or other important areas of functioning

O ? O 1 O 2 O 3

DYSTHYMIC DISORDER CRITERIA A, B, C, D, E, F, G, AND H ARE CODED "3"

false

Probe for Psychotic Symptoms In Coded Episode:

During this episode of Dysthymia, did you have any beliefs or ideas that you later found out were not true? (Like believing that you had powers and abilities others did not have? Or that you had a special mission, perhaps from God? Or that

IF DELUSIONS OR HALLUCINATIONS ARE SUSPECTED, PROBE FURTHER TO DETERMINE THE CONTENT AND WHETHER THE BELIEFS WERE HELD WITH CERTAINTY. USE THIS INFORMATION IN MODULE D MOOD

O ? O 1 O 3

someone was trying to harm you? How certain were you?) DIFFERENTIAL.

Did you see or hear things other people could not see or hear?

IF YES, DESCRIBE:

IF YES, PLEASE CHECK:

O Delusions

O Hallucinations

Lifetime Probe for Psychotic Symptoms:

Have you ever had either of these experiences (DELUSIONS/HALLUCINATIONS) during any other periods of Dysthymia?

IF YES, DESCRIBE:

IF DELUSIONS OR HALLUCINATIONS ARE SUSPECTED, PROBE FURTHER TO DETERMINE THE CONTENT AND WHETHER THE BELIEFS WERE HELD WITH CERTAINTY. USE THIS INFORMATION IN MODULE D MOOD DIFFERENTIAL.

O O O
? 1 3

IF YES, PLEASE CHECK:

O Delusions

O Hallucinations

NOTE, IF DELUSIONS ARE PRESENT FOR A SUBSTANTIAL PORTION OF TIME, SEE MODULE "C" AND CONSIDER DIAGNOSIS OF PSYCHOTIC DISORDER NOS.

WITH POSTPARTUM ONSET
FEMALE SUBJECTS ONLY

POSTPARTUM ONSET CRITERIA

Have you ever had an episode of DYSTHYMIA/OWN EQUIVALENT) which started within a month of childbirth?

Has ever had a Dysthymic period with onset within 4 weeks postpartum

O O O
? 1 3

ATYPICAL FEATURES SPECIFIER

ATYPICAL FEATURES CRITERIA

During times like this when you're feeling depressed...

If something good happens to you or someone tries to cheer you up, do you feel better, at least for a while?

A. Mood reactivity (i.e., mood brightens in response to actual or potential positive events)

O O O O
? 1 2 3

Two (or more) of the following features:

CODE BASED ON RESPONSE TO ITEM B(1) ON A.39.

(1) significant weight gain or increase in appetite

O O O O
? 1 2 3

How many hours (in a 24 hour period) do you usually sleep (including naps) on days when you're feeling depressed?

(2) hypersomnia [NOTE: CODE "3" IF MORE THAN 10 HOURS A DAY]

O O O O
? 1 2 3

Do your arms or legs often feel heavy (as though they were full of lead)?	(3) leaden paralysis (i.e., heavy, leaden feeling in arms or legs)	O ? O 1 O 2 O 3
Are you especially sensitive to how others treat you?What happens to you when someone rejects, criticizes, or slights you? (Do you get very down or angry? For how long? How has this affected you? Is your reaction more extreme than most people's?)Have you avoided doing things or being with people because you were afraid of being criticized or rejected?	(4) long-standing pattern of interpersonal rejection sensitivity (not limited to episodes of mood disturbance) that results in significant social or occupational impairment	O ? O 1 O 2 O 3

	AT LEAST TWO B CRITERIA ARE CODED "3"	false
	ATYPICAL FEATURES CRITERIA A AND B ARE CODED "3"	false

CYCLOTHYMIC DISORDER

(FOR GENETICS STUDY, SCORE PAST AND CURRENT EPISODES)

INTERVIEWER: If subject reported episodes of major depression or mania, distinguish these from the less severe, fluctuating mood changes typical of Cyclothymic Disorder by beginning the questions with: "Other than the severe episodes you've mentioned . . ."

Many subjects with Cyclothymic Disorder will have already reported numerous Hypomanias. In this case, interviewer must look for periods of depressive symptoms and establish chronicity.

CYCLOTHYMIC DISORDER CRITERIA

Have you ever had a two year period or longer when you have been a very moody person—someone who often had only a few hours or days when you felt better than normal or high and other times when you felt down or depressed?<br>IF YES: What was that like?	A. For at least two years, the presence of numerous periods with hypomanic symptoms and numerous periods with depressive symptoms that do not meet criteria for Major Depressive Episode. [Note: In children and adolescents, the duration must be at least 1 year.]	O ? O 1 O 2 O 3

How long did the longest period like this last? Was it at least two years?	ESTABLISH TWO-YEAR MINIMUM. IF 24 MONTHS OR LONGER, CODE "3" FOR CRITERION A. IF LESS THAN 24 MONTHS, CODE "1" FOR CRITERION A.	
Let's review when you first had these symptoms for at least two years. When did that period begin?	ESTABLISH ONSET OF FIRST EPISODE (MONTH/YEAR)	
	AGE	
When did this period end?	ESTABLISH OFFSET OF FIRST EPISODE (WITH 24 MONTH MINIMUM) (MONTH/YEAR)	
	AGE	
	CODE THIS IDENTIFIED FIRST EPISODE IN THE FOLLOWING SERIES OF QUESTIONS.	
During this period, did you experience any of the following symptoms . . .	PRESENCE OF AT LEAST TWO ELATED SYMPTOMS.	O ? O 1 O 2 O 3

O euphiric or irritable mood?

O more active or energetic than usual?

O needing less sleep than usual?

O more talkative than usual?

O thoughts racing?

O feeling very important?

During this period, did you have any of the following symptoms . . .	PRESENCE OF AT LEAST TWO DEPRESSED SYMPTOMS.	O O O O<br>? 1 2 3

O trouble sleeping or sleeping too much?

O loss of appetite or overeating?

O trouble concentrating?

O loss of energy?

O feeling worthless or guilty?

O being unable to enjoy things?

O thinking about death?

During that period, was your mood ever normal for more than two months in a row—that is, two months when you were not sad, blue, down, or high?	B. During the above 2-year period (1 year in children and adolescents), the person has not been without the symptoms in Criterion A for more than 2 months at a time.	O O O O<br>? 1 2 3
Did you have an episode of depression or mania during the first two years of this period?	C. No Major Depressive Episode, Manic Episode, or Mixed Episode has been present during the first 2 years of the disturbance.<br><br>[Note: After the initial 2 years of Cyclothymic Disorder, there may be superimposed Manic, Mixed or Major Depressive Episodes.]	O O O O<br>? 1 2 3
	D.The symptoms in Criterion A are not better accounted for by Schizoaffective Disorder and are not superimposed on Schizophrenia, Schizophreniform Disorder, Delusional Disorder, or Psychotic Disorder NOS.	O O O O<br>? 1 2 3
Just before and during this period was there a change in your use of street drugs, alcohol, or prescription medications, or did you have any serious physical illnesses?	E. The symptoms are not due to the direct physiological effects of a substance (e.g., a drug of abuse, a medication) or a general medical condition (e.g., hyperthyroidism).	O O O O<br>? 1 2 3

IF UNKNOWN: At that time, did you have serious problems at home or at work (school) because you were (SYMPTOMS)?	F. The symptoms cause clinically significant distress or impairment in social, occupational, or other important areas of functioning.	O ? O 1 O 2 O 3

	CYCLOTHYMIC DISORDER CRITERIA A, B, C, D, E, AND F. ARE CODED "3"	false
Over the course of your life have you primarily experienced episodes of highs or lows?	PREDOMINANT SYMPTOMS: CHECK ONE:	O Hypomanic<br>O Depressive<br>O Mixed/Irritable

How frequently do your moods typically switch?	DETERMINE USUAL DURATION OF CYCLING: CHECK ONE.	O Hourly<br>O Daily<br>O Weekly<br>O Unknown

Probe for Psychotic Symptoms In Coded Episode:

During this episode of Cyclothymia, did you have any beliefs or ideas that you later found out were not true? (Like believing that you had powers and abilities others did not have? Or that you had a special mission, perhaps from God? Or that someone was trying to harm you? How certain were you?)<br>IF YES, DESCRIBE:	IF DELUSIONS OR HALLUCINATIONS ARE SUSPECTED, PROBE FURTHER TO DETERMINE THE CONTENT AND WHETHER THE BELIEFS WERE HELD WITH CERTAINTY. USE THIS INFORMATION IN MODULE D MOOD DIFFERENTIAL.	O ? O 1 O 3
Did you see or hear things other people could not see or hear?<br>IF YES, DESCRIBE:	IF YES, PLEASE CHECK:	O Delusions<br>O Hallucinations

Lifetime Probe for Psychotic Symptoms:

Have you ever had either of these experiences (DELUSIONS/HALLUCINATIONS) during any other periods of Cyclothymia?<br>IF YES, DESCRIBE:	IF DELUSIONS OR HALLUCINATIONS ARE SUSPECTED, PROBE FURTHER TO DETERMINE THE CONTENT AND WHETHER THE BELIEFS WERE HELD WITH CERTAINTY. USE THIS INFORMATION IN MODULE D MOOD DIFFERENTIAL.	O ? O 1 O 3
	IF YES, PLEASE CHECK:	O Delusions<br>O Hallucinations
	NOTE, IF DELUSIONS ARE PRESENT FOR A SUBSTANTIAL PORTION OF TIME, SEE MODULE "C" AND CONSIDER DIAGNOSIS OF	

WITH POSTPARTUM ONSET<br>FEMALE SUBJECTS ONLY	POSTPARTUM ONSET CRITERIA	
Have you ever had an episode of (CYCLOTHYMIA/OWN EQUIVALENT) which started within a month of childbirth?	Has ever had a Cyclothymic period with onset within 4 weeks postpartum	O O O<br>? 1 3

HYPERTHYMIC TEMPERAMENT

(FOR GENETICS STUDY, SCORE PAST AND CURRENT EPISODES)

INTERVIEWER: If subject reported episodes of mania or hypomania, distinguish these from the less severe periods of hyperthymia by beginning the questions with: "Other than the severe episodes you mentioned" . .

HYPERTHYMIC TEMPERAMENT CRITERIA

A. pervasive pattern of energetic, cheerful, and/or irritable mood present in a variety of contexts, as indicated by at least three (or more) of the following:

Are you usually a very energetic, hyper, driven, or excited person? (Do other people comment on this?)	(1) High energy as either by subjective account or observation made by others	O O O O<br>? 1 2 3
Are you usually very cheerful or very optimistic? (Tell me more about this.)<br><br>What about irritable or angry or cranky? (Tell me about that.)	2) Cheerful, overly optimistic, or exuberant<br>OR<br>Irritable or angry	O O O O<br>? 1 2 3
	IF YES: CHOOSE ONLY ONE	O Elated<br>O Irritable
Are you generally a warm and outgoing person? (Tell me more about this.)<br><br>Are you a very social person? (Tell me about that.)	(3) Warm, people-seeking, or extroverted, gregarious.	O O O O<br>? 1 2 3
Have you often done things that were uninhibited or unrestrained? (What have you done?)<br><br>Do you often look for things to do that are stimulating or risky? (Can you give me some examples of that?)	(4) Uninhibited, stimulus seeking, risk taking, or promiscuous	O O O O<br>? 1 2 3

IF YES TO EITHER ABOVE: How often does it happen? What kinds of problems has it caused?

Do you often become over-involved in things at home or work? (Tell me more about that.)<br><br>Have you often found yourself getting too involved with others to the point that they were bothered by it? (Can you give me examples of that?)	(5) Over-involved or meddlesome	O ? O 1 O 2 O 3
Do you often feel overly confident, or like you had special powers or abilities? (Tell me more about this.)<br><br>Have you often bragged a lot? (Tell me about that.)	(6) Overconfident, self-assured, boastful, bombastic, or grandiose	O ? O 1 O 2 O 3
Have you often been described as being well-spoken? (Tell me more about this.)<br><br>Have you often been told that you talked too much? (Tell me about that.)<br><br>What about being described as the life of the party? (Did you enjoy being "on stage " or the center of attention?)	(7) Articulate, verbose, jocular, or attention-seeking	O ? O 1 O 2 O 3
		O ? O 1 O 2 O 3
	[RECORD AVERAGE NUMBER OF HOURS PER NIGHT]	
	NUMBER OF HYPERTHYMIC TEMPERAMENT CRITERIA MET	0
	AT LEAST THREE HYPERTHYMIC TEMPERAMENT CRITERIA ARE CODED "3"	false

WITH POSTPARTUM ONSET

POSTPARTUM ONSET CRITERIA

FEMALE SUBJECTS ONLY

Have you ever had (HYPERTHYMIA/ OWN EQUIVALENT) start or get more noticeable within a month of childbirth?	Has ever had Hyperthymia start or amplify within 4 weeks postpartum	O ? O 1 O 3

MOOD DISORDER DUE TO A GENERAL MEDICAL CONDITION

IF SYMPTOMS NOT TEMPORALLY ASSOCIATED WITH A GENERAL MEDICAL CONDITION, CHECK HERE AND GO TO *SUBSTANCE-INDUCED MOOD DISORDER

O Check Here

MOOD DISORDER DUE TO A GENERAL MEDICAL CONDITION CRITERIA

CODE BASED ON INFORMATION ALREADY OBTAINED	A. A prominent and persistent disturbance in mood predominates in the clinical picture and is characterized by either (or both) of the following:	
	(1) depressed mood or markedly diminished interest or pleasure in all, or almost all, activities	O ? O 1 O 2 O 3
	(2) elevated, expansive, or irritable mood	O ? O 1 O 2 O 3
	MET EITHER A1 AND/OR A2 CRITERION (OR BOTH)	false
Do you think your (MOOD SYMPTOMS) were in any way related to your (COMORBID GENERAL MEDICAL CONDITION)?<br>IF YES: Tell me how.<br><br>Did the (MOOD SYMPTOMS) start or get much worse only after (COMORBID GENERAL MEDICAL CONDITION) began?<br>IF YES AND GMC HAS RESOLVED: Did the (MOOD SYMPTOMS) get better once the (COMORBID GENERAL MEDICAL CONDITION) got better?	B./C. There is evidence from the history, physical examination, or laboratory findings that the disturbance is the direct physiological consequence of a general medical condition and the disturbance is not better accounted for by another mental disorder (e.g., Adjustment Disorder With Depressed Mood, in response to the stress of having a general medical condition).<br><br>THE FOLLOWING FACTORS SHOULD BE CONSIDERED AND SUPPORT THE CONCLUSION THAT THE GMC IS ETIOLOGIC TO THE MOOD SYMPTOMS:<br><br>1) THERE IS EVIDENCE FROM THE LITERATURE OF A WELL ESTABLISHED ASSOCIATION BETWEEN THE GMC AND MOOD SYMPTOMS.<br><br>2) THERE IS A CLOSE TEMPORAL RELATIONSHIP BETWEEN THE COURSE OF THE MOOD SYMPTOMS AND THE COURSE OF THE GENERAL MEDICAL CONDITION.<br><br>3) THE MOOD SYMPTOMS ARE CHARACTERIZED BY UNUSUAL PRESENTING FEATURES (E.G., LATE AGE AT ONSET).<br><br>4) THE ABSENCE OF ALTERNATIVE EXPLANATIONS (E.G., MOOD SYMPTOMS AS A PSYCHOLOGICAL REACTION TO THE GMC).	O ? O 1 O 2 O 3

IF UNKNOWN: How much did (MOOD SYMPTOMS) interfere with your life?

E. The symptoms cause clinically significant distress or impairment in social, occupational, or other important areas of functioning.

O ? O 1 O 2 O 3

D. The disturbance does not occur exclusively during the course of Delirium.

O 1 O 3

MOOD DISORDER DUE TO A GMC CRITERIA A, B, C, D, AND E ARE CODED "3"

false

If these are the only mood symptoms reported in subject's lifetime, Complete Modules B and C and D1 *SUICIDAL IDEATION SUMMARY* and then skip to D32 to first make a formal diagnosis of Mood Disorder Due to a GMC and then complete Diagnostic Specifiers.

If they are not the only mood symptoms reported, return to A1 or A20 to continue coding potential mood episodes.

SUBSTANCE-INDUCED MOOD DISORDER

EPISODE BEING EVALUATED FOR SUBSTANCE INDUCED CRITERIA: (SELECT ONE)

O Major Depression

O Minor Depression

O Mania/Hypomania

O Dysthymic

O Cyclothymic

O Hyperthymic

IF SYMPTOMS NOT TEMPORALLY ASSOCIATED WITH SUBSTANCE, CHECK HERE AND RETURN TO EPISODE BEING EVALUATED.

O Check Here

SUBSTANCE-INDUCED MOOD DISORDER CRITERIA

CODE BASED ON INFORMATION ALREADY OBTAINED

A. A prominent and persistent disturbance in mood predominates in the clinical picture and is characterized by one (or both) of the following:

(1) Depressed mood or markedly diminished interest or pleasure in all, or almost all, activities

O ? O 1 O 2 O 3

(2) Elevated, expansive, or irritable mood

O ? O 1 O 2 O 3

	MET A1 AND/OR A2 CRITERION	false
IF UNKNOWN: When did the (MOOD SYMPTOMS) begin? Were you already using (SUBSTANCE) or had you just stopped or cut down your use?	B. There is evidence from the history, physical examination, or laboratory findings that either (1) the symptoms in A developed during or within a month of substance intoxication or withdrawal, or (2) medication use is etiologically related to the disturbance	O O O O<br>? 1 2 3
Do you think your (MOOD SXS) are in any way related to your (SUBSTANCE USE)?<br>IF YES: Tell me how.<br><br>[ASK ANY OF THE FOLLOWING QUESTIONS AS NEEDED TO RULE OUT A NON-SUBSTANCE-INDUCED ETIOLOGY]<br><br>IF UNKNOWN: Which came first, the (SUBSTANCE USE) or the (MOOD SYMPTOMS)?<br><br>IF UNKNOWN: Have you had a period of time when you stopped using (SUBSTANCE)?<br><br>IF YES: After you stopped using (SUBSTANCE) did the (MOOD SYMPTOMS) get better?<br><br>IF UNKNOWN: How much (SUBSTANCE) were you using when you began to have (MOOD SYMPTOMS)?<br><br>IF UNKNOWN: Have you had any other episodes of (MOOD SYMPTOMS)?<br><br>IF YES: How many? Were you using (SUBSTANCES) at those times?	C. The disturbance is not better accounted for by a Mood Disorder that is not substance-induced. Evidence that the symptoms are better accounted for by a Mood Disorder that is not substance-induced might include:<br><br>1) the mood symptoms precede the onset of the Substance Abuse or Dependence (or medication use)<br><br>2) the mood symptoms persist for a substantial period of time (e.g., about a month) after the cessation of acute withdrawal or severe intoxication<br><br>3) the mood symptoms are substantially in excess of what would be expected given the type, duration, or amount of the substance used<br><br>4) there is evidence suggesting the existence of an independent non-substance-induced Mood Disorder (e.g., a history of recurrent Major Depressive Episodes)	O O O O<br>? 1 2 3
IF UNKNOWN: How much did (MOOD SYMPTOMS) interfere with your life?	E. The symptoms cause clinically significant distress or impairment in social, occupational, or other important areas of functioning	O O O O<br>? 1 2 3
	D. The disturbance does not occur exclusively during the course of Delirium	O O<br>1 3
	If these are the only mood symptoms reported in subject's lifetime, Complete Modules B and C and D1 *SUICIDAL IDEATION SUMMARY* and then skip to D35 to first make a formal diagnosis of Substance-Induced Mood Disorder and then complete Diagnostic Specifiers.<br><br>If they are not the only mood symptoms reported, return to A1 or A20 to continue coding potential	

mood episodes.

Module B: Psychotic Symptoms

THIS MODULE IS FOR CODING PSYCHOSIS AND ASSOCIATED SYMPTOMS THAT HAVE BEEN PRESENT AT ANY POINT IN THE SUBJECT'S LIFETIME.

FOR EACH PSYCHOTIC SYMPTOM CODED "3", DESCRIBE THE ACTUAL CONTENT, HOW FIRMLY THE BELIEF IS HELD, AND INDICATE THE PERIOD OF TIME DURING WHICH THE SYMPTOM WAS PRESENT.

DELUSIONS

IF ALREADY HAS ACKNOWLEDGED PSYCHOTIC SYMPTOMS: You've told me about (PSYCHOTIC SYMPTOMS). Now I'd like to ask you about other experiences like that.

IF NO ACKNOWLEDGEMENT OF PSYCHOTIC SYMPTOMS SO FAR: Now I'd like to ask you about unusual experiences that people sometimes have.

DELUSIONS CRITERIA

False personal beliefs based on incorrect inference about external reality and firmly sustained in spite of what almost everyone else believes and in spite of what constitutes incontrovertible and obvious proof or evidence to the contrary. The belief is not one ordinarily accepted by other members of the person's culture or subculture. Code overvalued ideas (unreasonable and sustained beliefs that are maintained with less than delusional intensity) as "2".

Has it ever seemed like people were talking about you or taking special notice of you?

IF YES: Were you convinced they were talking about you or did you think it might have been your imagination?

CONTINUE ASKING...
What about receiving special messages from the TV, radio, or newspaper, or from the way things were arranged around you?

Delusion of reference, i.e. events, objects, or other people in the individual's immediate environment have a particular or unusual significance.

O ? O 1 O 2 O 3

DESCRIBE:

What about anyone going out of their way to give you a hard time, or to try to hurt you?

Persecutory delusion, i.e., the individual (or his or her group) is being attacked, harassed, cheated, persecuted, or conspired against.

O ? O 1 O 2 O 3

DESCRIBE:

Did you ever feel that you were especially important in some way, or that you had special powers to do things that other people couldn't do?

Grandiose delusion, i.e., content involves exaggerated power, knowledge or importance, or a special relationship to a deity or famous person.

O ? O 1 O 2 O 3

DESCRIBE:

Did you ever feel that something was very wrong with you physically even though your doctor said

Somatic delusion, i.e., content involves change or disturbance in body appearance or functioning.

O ? O 1 O 2 O 3

nothing was wrong...like you had cancer or some other terrible disease?

Have you ever been convinced that something was very wrong with the way a part or parts of your body looked?

DESCRIBE:

Did you ever feel that something strange was happening to parts of your body?

Did you ever have any unusual religious experiences?

Other delusions

O ? O 1 O 2 O 3

Did you ever feel that you had committed a crime or done something terrible for which you should be punished?

Check if:

O religious delusions

O delusions of guilt

O jealous delusions

O erotomanic delusions

Have you ever been convinced that your significant other was being unfaithful to you?

Have you ever believed that another person was in love with you when there was no real reason to think so?

DESCRIBE:

IF NEVER HAD A DELUSION AND THERE IS NO SUSPICION OF ANY PSYCHOTIC FEATURES, CHECK HERE AND GO TO HALLUCINATIONS.

O Check Here

Did you ever feel that someone or something outside yourself was controlling your thoughts or actions against your will?

Delusion of being controlled, i.e., feelings, impulses, thoughts or actions are experienced as being under the control of some external force

O ? O 1 O 2 O 3

Did you ever feel that certain thoughts that were not your own were put into your head?

DESCRIBE:

What about taken out of your head?

CHECK IF:

O thought insertion

O thought withdrawl

Did you ever feel as if your thoughts were being broadcast out loud so that other people could actually hear what you were thinking?

Thought broadcasting, i.e., the delusion that one's thoughts are audible to others

O ? O 1 O 2 O 3

Did you ever believe that someone could read your mind?

DESCRIBE:

IF APPLICABLE: How do you explain (CONTENT OF BIZARRE DELUSION)?

Bizarre delusion, i.e., involving a phenomenon that the individual's subculture would regard as totally implausible (e.g., the person's brain has been

O ? O 1 O 2 O 3

removed and replaced with someone else's brain)

DESCRIBE:

HALLUCINATIONS (PSYCHOTIC)

A sensory perception that has the compelling sense of reality of a true perception but occurs without external stimulation of the relevant sensory organ. (CODE "2" FOR HALLUCINATIONS THAT ARE SO TRANSIENT AS TO BE WITHOUT DIAGNOSTIC SIGNIFICANCE)

AUDITORY HALLUCINATIONS CRITERIA

Did you ever hear things that other people couldn't hear, such as noises, or the voices of people whispering or talking? (Were you awake at the time?)<br><br>IF YES: What did you hear? How often did you hear it?	Auditory hallucinations when fully awake, heard either inside or outside of head	O ? O 1 O 2 O 3

DESCRIBE:

IF VOICES: Did they comment on what you were doing or thinking?	A voice keeping up a running commentary on the individual's behavior or thoughts as they occur	O ? O 1 O 2 O 3

DESCRIBE:

IF APPLICABLE: How many voices did you hear? Were they talking to each other?	Two or more voices conversing with each other	O ? O 1 O 2 O 3

DESCRIBE:

VISUAL HALLUCINATIONS CRITERION

Did you ever have visions or see things that other people couldn't see? (Were you awake at the time? How long were they present?)	Visual hallucinations<br><br>NOTE: DISTINGUISH FROM AN ILLUSION, I.E., A MISPERCEPTION OF A REAL EXTERNAL STIMULUS.	O ? O 1 O 2 O 3

DESCRIBE:

OTHER HALLUCINATIONS CRITERIA

What about strange sensations in your body or on your skin?	Tactile hallucinations, e.g., electricity	O ? O 1 O 2 O 3

DESCRIBE:

What about smelling or tasting things that other people couldn't smell or taste?	Other hallucinations, e.g., gustatory, olfactory	O ? O 1 O 2 O 3
	Check if:	O gustatory<br>O olfactory

DESCRIBE:

IF NO SUGGESTION THAT THERE HAVE EVER BEEN PSYCHOTIC SYMPTOMS, CHECK HERE AND SKIP TO MODULE D. O Check Here

OTHER PSYCHOTIC SYMPTOMS

(Let me stop for a minute while I make a few notes...)	**OTHER PSYCHOTIC SYMPTOMS CRITERIA**	
THE FOLLOWING ITEMS ARE RATED BASED ON OBSERVATION AND HISTORY (CONSULT OLD CHARTS, OTHER OBSERVERS, E.G., FAMILY MEMBERS, THERAPEUTIC STAFF)	Catatonic behavior:motoric immobility (i.e., catalepsy or stupor)	O ? O 1 O 2 O 3
	excessive motor activity (i.e., apparently purposeless agitation not influenced by external stimuli)	O ? O 1 O 2 O 3
	extreme negativism (i.e., apparently motiveless resistance to instructions or attempts to be moved) or mutism	O ? O 1 O 2 O 3
	posturing or stereotyped movements	O ? O 1 O 2 O 3
	echolalia or echopraxia	O ? O 1 O 2 O 3

DESCRIBE:

	Grossly disorganized behavior: May range from childlike silliness to unpredictable agitation. The person may appear markedly disheveled, may dress in an unusual manner (e.g., wearing multiple overcoats, scarves, and gloves on a hot day), display clearly inappropriate sexual behavior (e.g., public masturbation) or unpredictable and untriggered agitation (e.g., shouting or swearing).	O ? O 1 O 2 O 3

DESCRIBE:

	Grossly inappropriate affect: affect that is clearly discordant with the content of speech or ideation, e.g., smiling while discussing being persecuted.	O ? O 1 O 2 O 3

DESCRIBE:

Disorganized speech: frequent derailment (loosening of associations) or incoherence; derailment is a pattern of speech in which the ideas slip off the track onto another that is completely unrelated or only obliquely related. The person may shift the topic idiosyncratically from one frame of reference to another and things may be said in juxtaposition that lack a meaningful relationship. Incoherence is speech that is essentially incomprehensible to others because words or phrases are joined together without a logical or meaningful connection.

O ? O 1 O 2 O 3

DESCRIBE:

NEGATIVE SYMPTOMS

FOR ANY NEGATIVE SYMPTOMS CODED "3", DETERMINE WHETHER THE SYMPTOMIS DEFINITELY PRIMARY OR WHETHER IT IS POSSIBLY PRIMARY/SECONDARY [I.E., RELATED TO ANOTHER MENTAL DISORDER (E.G., DEPRESSION), A SUBSTANCE (E.G., METHAMPHETAMINE) OR A GENERAL MEDICAL CONDITION (E.G., MEDICATION-INDUCED AKINESIA), OR TO ANOTHER PSYCHOTIC SYMPTOM (E.G., COMMAND HALLUCINATIONS NOT TO MOVE)]. IF UNCLEAR CODE AS SECONDARY.

NEGATIVE SYMPTOMS CRITERIA

IF UNKNOWN: How do you spend your time?

Avolition: an inability to initiate and persist in goal-directed activities. When severe enough to be considered pathological, avolition is pervasive and prevents the person from completing many different types of activities (e.g., work, intellectual pursuits, self-care).

O ? O 1 O 2 O 3

O 1Primary O 3Secondary

DESCRIBE:

Alogia: Impoverishment in thinking that is inferred from observing speech and language behavior. There may be restriction in the amount of spontaneous speech and brief and concrete replies to questions (poverty of speech). Sometimes the speech is adequate in amount but conveys little information because it is over-concrete, over-abstract, repetitive, or stereotyped (poverty of content).

O ? O 1 O 2 O 3

O 1Primary O 3Secondary

DESCRIBE:

Affective flattening: absence or near absence of signs of affective expression.

O ? O 1 O 2 O 3

O O

1Primary 3Secondary

DESCRIBE:

CHRONOLOGY OF PSYCHOTIC SYMPTOMS

IF ANY PSYCHOTIC SYMPTOMS ENDORSED, NOTE TYPE, COURSE, ONSET AND OFFSET DATES AND WHETHER PRESENT DURING PAST MONTH (E.G., "BIZARRE DELUSIONS OF BEING CONTROLLED BY ALIENS, PRESENT INTERMITTENTLY, ONSET 1969, OFFSET JUNE 1993, NOT).

IF UNKNOWN, ASK QUESTIONS LIKE: How often have you had (SYMPTOMS OF PSYCHOSIS)?

IF NOT CURRENTLY PRESENT: When did they last occur?

TYPE OF SYMPTOM	COURSE	ONSET	OFFSET	CHECK IF PRESENT LAST MONTH
				O

IF UNCLEAR: How old were you when you first experienced the types of symptoms we have been talking about?

Age at onset of first Psychotic symptoms

IF UNKNOWN: How many times did you have episodes of (PSYCHOTIC SYMPTOMS)?

Number of episodes or exacerbations (CODE 99 IF TOO NUMEROUS OR INDISTINCT TO COUNT)

END OF MODULE B **GO TO MODULE C**

Module C: Psychotic Differential

IF ALL PSYCHOTIC SYMPTOMS IN MODULE B. ARE DUE TO A SUBSTANCE OR A GENERAL MEDICAL CONDITION, GO TO *GMC/SUBSTANCE*. O Go to GMC/SUBSTANCE

IF THERE ARE NO ITEMS CODED "3" IN MODULE B, PSYCHOTIC AND ASSOCIATED SYMPTOMS, CHECK HERE AND SKIP TO MODULE D. O Check Here

BOTH PRIMARY PSYCHOTIC SYMPTOMS AND PSYCHOTIC SYMPTOMS THAT ARE SUBSTANCE-INDUCED OR DUE TO A GENERAL MEDICAL CONDITION MAY BE PRESENT IN THE SAME INDIVIDUAL AT THE SAME TIME. THIS MAY REQUIRE MULTIPLE "PASSES" THROUGH THE ALGORITHMS IN THIS MODULE.

IF A MAJOR DEPRESSIVE OR MANIC EPISODE HAS EVER BEEN PRESENT: Has there ever been a time when you had (PSYCHOTIC SYMPTOMS) and you were not (DEPRESSED/MANIC)?

Psychotic symptoms occur at times other than during Major Depressive, Manic, or Mixed Episodes.

O ? O 1 O 3

NOTE: CODE "3" IF NO MAJOR DEPRESSIVE, MANIC, OR MIXED EPISODES OR IF SOME PSYCHOTIC SYMPTOMS OCCUR OUTSIDE OF MOOD EPISODES. CODE "1" ONLY IF PSYCHOTIC SXS OCCUR EXCLUSIVELY DURING MOOD EPISODES.

SCHIZOPHRENIA

CHECK FOR PRESENCE OF ACTIVE PHASE SYMPTOMS.

[NOTE: CRITERIA ARE IN DIFFERENT ORDER THAN IN DSM-IV]

SCHIZOPHRENIA CRITERIA

REFER TO ITEMS CODED "3" IN MODULE B, PSYCHOTIC AND ASSOCIATED SYMPTOMS.	A. Two (or more) of the following, each present for a significant portion of time during a one month period (or less if successfully treated):<br><br>1. delusions<br>2. hallucinations<br>3. disorganized speech (e.g., frequent derailment or incoherence)<br>4. grossly disorganized or catatonic behavior<br>5. negative symptoms, i.e.,affective flattening, alogia, or avolition<br><br>NOTE: ONLY ONE "A" SYMPTOM IS REQUIRED IF DELUSIONS ARE BIZARRE OR HALLUCINATIONS CONSIST OF A VOICE KEEPING UP A RUNNING COMMENTARY ON THE PERSON'S BEHAVIOR OR THOUGHTS, OR TWO OR MORE VOICES CONVERSING WITH EACH OTHER.	O O O<br>? 1 3
IF UNKNOWN: Has there ever been a time when you had (SYMPTOMS FROM ACTIVE PHASE) at the same time that you were (DEPRESSED/HIGH/IRRIT-ABLE/OWN EQUIVALENT)?	D. Schizoaffective Disorder and Mood Disorder with psychotic features have been ruled out because either:<br><br>1. No Major Depressive, Manic or Mixed Episodes have occurred concurrently with the active phase symptoms (i.e., the "A" symptoms listed above)<br><br>CODE "3" IF NEVER ANY MAJOR DEPRESSIVE OR MANIC EPISODES OR IF ALL MAJOR DEPRESSIVE AND MANIC EPISODES OCCURRED DURING THE PRODROMOL OR RESIDUAL PHASE. CODE "1" IF ANY MOOD EPISODES OVERLAP WITH PSYCHOTIC SYMPTOMS.<br><br>NOTE: BECAUSE OF THE DIFFICULTY IN DISTINGUISHING THE PRODROMAL AND RESIDUAL SYMPTOMS OF SCHIZOPHRENIA FROM A MAJOR DEPRESSIVE SYNDROME, THE RATER SHOULD RECONSIDER ANY PREVIOUSLY CODED MAJOR DEPRESSIVE EPISODE TO BE SURE IT IS UNEQUIVOCAL.	O O O<br>? 1 3
IF UNKNOWN: How much of the time that you have had (SYMPTOPMS FROM ACTIVE AND RESIDUAL PHASES) would you say you have also been (DEPRESSED/ HIGH/IRRIT-ABLE/OWN EQUIVALENT)?	2. The total duration of mood episodes (occurring during the disturbance) has been brief relative to the total duration of the active and residual phases.<br><br>NOTE: CODE "1" IF SYMPTOMS MEETING CRITERIA FOR A MAJOR DEPRESSIVE, MANIC, OR MIXED EPISODE HAVE BEEN PRESENT FOR A SUBSTANTIAL PORTION OF THE TOTAL	O O O<br>? 1 3

	DURATION OF THE ACTIVE AND RESIDUAL PHASES.	
DIFFERENTIAL DIAGNOSIS BETWEEN SCHIZOPHRENIA AND SCHIZOPHRENIFORM DISORDER<br><br>IF UNKNOWN: Between (MULTIPLE EPISODES), were you back to your normal self?(How long did each episode last?)	C. Continuous signs of the disturbance persist for at least six months. This six-month period must include at least one month of symptoms that meet criterion A (i.e., active phase symptoms), and may include periods of prodromal or residual symptoms. During these prodromal or residual periods, the signs of disturbance may be manifested by only negative sxs (i.e., affective flattening, alogia, avolition) or two or more symptoms listed in criterion A present in an attenuated form (e.g., odd beliefs, unusual perceptual experiences, ideas of reference).	O O O<br>? 1 3
IF NOT ALREADY KNOWN: When you had (A CRITERION SYMPTOMS), were you working? (having a social life, taking care of yourself)?	B. For a significant portion of the time since the onset of the disturbance, one or more major areas of functioning such as work, interpersonal relations or self-care is markedly below the level achieved prior to the onset (or when the onset is in childhood or adolescence, failure to achieve expected level of interpersonal, academic, or occupational achievement).	O O O<br>? 1 3
IF UNKNOWN: Were you physically ill at this time?<br><br>IF UNKNOWN: Were you taking any drugs or medicines during this time?	E. The disturbance is not due to the direct physiological effects of a substance (e.g., a drug of abuse, a medication) or to a general medical condition.<br><br>Etiological general medical conditions include: neurological conditions (e.g., neoplasms, cerebrovascular disease, Huntington's disease epilepsy, auditory nerve injury, deafness, migraine, central nervous system infections); endocrine conditions (e.g., hyper- and hypo-parathyroidism, hypocortisolism); metabolic conditions (e.g., hypoxia, hypercarbia, hypoglycemia); fluid or electrolyte imbalances; hepatic or renal diseases; and autoimmune disorders with central nervous system involvement (e.g., systemic lupus erythematosis).<br><br>Etiological substances include:: alcohol, amphetamine, cannabis, cocaine, hallucinogens, inhalants, opiods (meperidine), phencyclidine sedatives, hypnotics, and anxiolytics, and other or	O O O<br>? 1 3

unknown substances.

Check here to move to GMC/Substance section O Check Here

SCHIZOPHRENIA FEATURES/SUBTYPES

DETERMINE THE PREDOMINANT LIFETIME PHENOMENOLOGIC TYPE AND INDICATE ON THE DIAGNOSTIC SCORE SHEET (DSS):

**PARANOID TYPE**	Paranoid Type: Currently the following two criteria are met: A. Preoccupation with one or more delusions or frequent auditory hallucinations B. None of the following is prominent: disorganized speech, disorganized behavior, flat or inappropriate affect or catatonic behavior.	O<br>?	O<br>1	O<br>3
**CATATONIC TYPE**	Catatonic Type: Currently the clinical picture is dominated by at least two of the following:<br><br>1) motoric immobility as evidenced by catalepsy (including waxy flexibility) or stupor<br>2) excessive motor activity (that is apparently purposeless and not influenced by external stimuli)<br>3) extreme negativism (an apparently motiveless resistance to all instructions or maintenance of a rigid posture against attempts to be moved) or mutism<br>4) peculiarities of voluntary movement as evidenced by posturing (voluntary assumption of inappropriate or bizarre postures), stereotyped movements, prominent mannerisms, or prominent grimacing<br>5) echolalia or echopraxia	O<br>?	O<br>1	O<br>3
**DISORGANIZED TYPE**	Disorganized Type: currently the following criteria are met:<br>A. All of the following are prominent:<br>1) disorganized speech<br>2) disorganized behavior<br>3) flat or inappropriate affect<br><br>B. Does not meet criteria for Catatonic type.	O<br>?	O<br>1	O<br>3

**UNDIFFERENTIATED TYPE**	Undifferentiated Type: currently symptoms meeting criterion A for Schizophrenia are present, but the criteria are not met for the Paranoid, Catatonic, or Disorganized Types.	O O O<br>? 1 3
**RESIDUAL TYPE**	Residual Type: currently, the following criteria are met:<br>A. Criterion A for Schizophrenia (i.e., active phase symptoms) is no longer met, and criteria are not met for the Paranoid, Catatonic, Disorganized, and Undifferentiated types.<br><br>B. There is continuing evidence of the disturbance, as indicated by the presence of negative symptoms or two or more symptoms listed in criterion A for Schizophrenia, present in an attenuated form (e.g., odd beliefs, unusual perceptual experiences)	O O O<br>? 1 3
**NO SUBTYPE**	CHECK ONE IF APPLICABLE:	O No Subtype O No Information

SCHIZOPHRENIA DIAGNOSTIC SPECIFIERS

LIFETIME SEVERITY:

(Additional questions regarding impairment may be necessary.)

O Mild: (GAF = 61-80) Minimum symptom criteria are met or few, if any, symptoms in excess of those required to make the diagnosis and/or symptoms result in only minimal interference in normal functioning. Either mild disability is present or substantial or unusual effort is needed to function normally.

O Moderate: (GAF = 51-60) Intermediate between "mild" and "severe."

O Severe: (GAF lt;= 50) Several symptoms in excess of those required to make the diagnosis and/or symptoms result in only limited functioning in most aspects of life almost all the time.

O No Information

PSYCHOTIC FEATURES:

(This rating refers to Schizophrenia over the subject's lifetime.)

O With Prominent Negative Symptoms: Prominent negative symptoms are present during the majority of the illness.

O Without Prominent Negative Symptoms: No prominent negative symptoms are present during the majority of the illness.

O No Information

CURRENT STATE:

O Single Episode In Partial Remission: This specifier applies when there has been a single episode in which criterion A for Schizophrenia is met and some clinically significant residual symptoms remain for less than two months following the end of the episode.

O Single Episode In Full Remission: This specifier applies when there has been a single episode in which criterion A for Schizophrenia is met and no clinically significant residual symptoms remain for the past two months.

O Currently Meets Full Criteria: Full Criteria for an illness must be met at the time of the interview.

O Currently Symptomatic: Symptoms are present but full criteria are not met. This differs from Partial Remission in that it does not immediately follow a period of illness (i.e., there was a symptom-free period of at least two months before the current symptoms began).

O No Information

LIFETIME COURSE:

O Episodic With Interepisode Residual: This specifier applies when the course is characterized by episodes in which criterion A for Schizophrenia is met and there are clinically significant residual symptoms between these episodes.

O Episodic With No Interepisode Residual: This specifier applies when the course is characterized by episodes in which criterion A for Schizophrenia is met and there are no clinically significant residual symptoms between these episodes.

O Continuous: This specifier applies when characteristic symptoms of criterion A are met throughout all (or most) of the course of illness.

O No Information

SPECIFIC PATTERNS:

O Single Episode: This specifier applies when there has been a single episode in which criterion A for Schizophrenia is met.

O Multiple Episodes: This specifier is applied when there has been two or more psychotic episodes in which criterion A for Schizophrenia is met.

O Other or Unspecified Pattern: This specifier is used if another or unspecified course pattern has been present (or if pattern is unknown).

O No Information

ONSET:

O History of Substance Induced Onset: This specifier applies if substance use may be etiologically associated with the onset of illness.

O No History of Substance Induced Onset: This specifier is applied when there has been no evidence that a substance was etiologically associated with the onset of illness.

O No Information

CATATONIC FEATURES:

O History of Catatonic Features: This specifier applies if the subject has ever experienced any catatonic symptoms in his/her lifetime (see page B5).

O No History of Catatonic Features: This specifier is applied when the subject has never experienced any catatonic symptoms in his/her lifetime (see page B5).

O No Information

CHRONOLOGY

How old were you when you first began to have these experiences regularly?	DETERMINE AGE AT ONSET OF SCHIZOPHRENIA	

***WITH POSTPARTUM ONSET* FEMALES SUBJECTS ONLY**

Have you ever had an episode of (PSYCHOSIS/OWN EQUIVALENT) which started within a month of childbirth?	Has ever had a psychotic episode with onset within 4 weeks postpartum	O ? O 1 O 3

SEASONAL PATTERN

IF THERE HAVE BEEN FEWER THAN TWO PSYCHOTIC EPISODES, CHECK HERE AND GO TO NEXT MODULE (D). O Check Here

IF UNKNOWN: Do the periods of (PSYCHOSIS/OWN EQUIVALENT) mostly seem to happen at the same time of the year, like fall or winter?	A. There has been a regular temporal relationship between the onset of the psychotic symptoms and a particular time of year (e.g., regular appearance of psychotic symptoms in the fall or winter).<br><br>NOTE: DO NOT INCLUDE CASES IN WHICH THERE IS AN OBVIOUS EFFECT OF SEASONAL RELATED PSYCHOSOCIAL STRESSORS	O 1 O 3
IF UNKNOWN: Do the periods of (PSYCHOSIS/OWN EQUIVALENT) get better around the same time of year, like in the spring?	B. Full remissions also occur at a characteristic time of the year (e.g., psychotic symptoms disappear in the spring).	O 1 O 3

IF UNKNOWN: How many periods of (PSYCHOSIS/OWN EQUIVALENT) did you have during the past two years?How many of these occurred at the same time of the year?	C. In the last 2 years, two Psychotic Episodes have occurred that demonstrate the temporal relationship defined in criteria A and B, and no non-seasonal episodes have occurred during that same period.	O O<br>1 3
IF UNKNOWN: About how many years can you recall having had periods of (PSYCHOSIS/OWN EQUIVALENT)? How often did they occur in (ALTERNATES OF SEASONS STATED) rather than in (SEASONS STATED)?	D. Seasonal Psychotic Episodes (as described above) substantially outnumber the non-seasonal Psychotic Episodes that may have occurred over the individual's lifetime.	O O<br>1 3

SCHIZOAFFECTIVE DISORDER

SCHIZOPHRENIA AND SCHIZOPHRENIFORM DISORDER HAVE BEEN RULED OUT BECAUSE OF PROMINENT MOOD SYMPTOMS. CONSIDER A DIAGNOSIS OF SCHIZOAFFECTIVE DISORDER.

SCHIZOAFFECTIVE DISORDER CRITERIA

	A. An uninterrupted period of illness during which, at some time, there is either a Major Depressive Episode (which must include A (1) depressed mood), a Manic or a Mixed Episode concurrent with symptoms that meet Criterion A for Schizophrenia.	O O O<br>? 1 3
IF NOT ALREADY KNOWN:Have there been any times when you had (PSYCHOTIC SYMPTOMS) when you were not (MANIC OR DEPRESSED)?	B. During the same period of illness, there have been delusions or hallucinations for at least two weeks in the absence of prominent mood symptoms.	O O O<br>? 1 3
	C. Symptoms meeting criteria for a mood episode are present for a substantial portion of the total duration of the active and residual periods of the illness.	O O O<br>? 1 3
IF UNKNOWN: Were you taking any drugs or medicines during this time?<br><br>IF UNKNOWN: Were you physically ill at this time?	D. Not due to the direct physiological effects of a substance (e.g., a drug of abuse, medication) or to a general medical condition.<br><br>IF GENERAL MEDICAL CONDITION OR SUBSTANCE MAY BE ETIOLOGICALLY ASSOCAITED WITH PSYCHOTIC SYMPTOMS, GO TO *GMS/SUBSTANCE*, C21 AND RETURN HERE FOR RATING OF "1" OR "3"<br><br>REFER TO LIST OF GENERAL MEDICAL CONDITIONS AND SUBSTANCES ON PAGE C3.	O O O<br>? 1 3

SCHIZOAFFECTIVE DISORDER DIAGNOSTIC SPECIFIERS

LIFETIME SEVERITY:

(Additional questions regarding impairment may be necessary.)

- O Mild: (GAF = 61-80) Minimum symptom criteria are met or few, if any, symptoms in excess of those required to make the diagnosis and/or symptoms result in only minimal interference in normal functioning. Either mild disability is present or substantial or unusual effort is needed to function normally.
- O Moderate: (GAF = 51-60) Intermediate between "mild" and "severe."
- O Severe: (GAF lt;= 50) Several symptoms in excess of those required to make the diagnosis and/or symptoms result in only limited functioning in most aspects of life almost all the time.
- O No Information

PSYCHOTIC FEATURES:

(This rating refers to Bipolar I Disorder over the subject's lifetime. Severity ratings in Module A refer to the actual depression and manic symptoms as opposed to the diagnosis as a whole.)

- O Mood-congruent psychotic features: Delusions or hallucinations whose content is entirely consistent with either the typical manic themes of inflated worth, power, knowledge, identity, or special relationship to a deity or famous person, or the typical depressive themes of personal inadequacy, guilt, disease, death, nihilism, or deserved punishment.
- O Mood-incongruent psychotic features: Delusions or hallucinations whose content does not involve either typical manic themes of inflated worth, power, knowledge, identity, or special relationship to a deity or famous person or typical depressive themes of personal inadequacy, guilt, disease, death, nihilism, or deserved punishment. Included are such symptoms as persecutory delusions (not directly related to grandiose ideas/themes or to depressive themes), thought insertion, thought broadcasting, and delusions of being controlled.
- O History of Psychotic Features, Unspecified
- O No History of Psychotic Features
- O No Information

CURRENT STATE:

- O In Partial Remission: Symptoms of a Hypomanic, Mixed, or Major Depressive Episode are present but full criteria are not met, or there is a period without any significant symptoms of a Manic, Mixed, or Major Depressive Episode lasting less than two months following the end of the episode.
- O In Full Remission: During the past two months no significant signs or symptoms of the disturbance.
- O Currently Meets Full Criteria: Full Criteria for an illness must be met at the time of the interview
- O Currently Symptomatic: Symptoms are present but full criteria are not met. This differs from Partial Remission in that it does not immediately follow a period of illness (i.e., there was a symptom-free period of at least two months before the current symptoms began).
- O No Information

LIFETIME COURSE:

- O With Full Interepisode Recovery: Full remission is attained between most Mood Episodes for at least two months.
- O Without Full Interepisode Recovery: Full remission is not attained between most Mood Episodes.
- O Chronic: Continuously met criteria for a minimum of two years since onset of illness.
- O N/A: Only one episode
- O No Information

FEATURES/SUBTYPES:

- O Bipolar Type: Manic Episode or Mixed Episode (with or without Major Depressive Episodes) during the course of the disturbance.
- O Depressive Type: Major Depressive Episodes during the course of the disturbance
- O Other or Unknown Type
- O No Information

MOST RECENT EPISODE:

(Indicate type of current (or most recent) episode.)

O Hypomanic

O Manic

O Mixed

O Depressed

O Unspecified: Criteria, except for duration, are currently met for a Manic, Hypomanic, Major Depressive, or Mixed Episode.

O No Information

SPECIFIC PATTERNS:

IF UNKNOWN: Have you had several periods of being (MANIC/HIGH/ IRRITABLE/OWN WORDS) and periods of being (DEPRESSED/ OWN WORDS) during the course of one year?	At least four episodes of a mood disturbance in the past 12 months that meet criteria for a Depressive, Manic, Mixed, or Hypomanic Episode. Note: Episodes are demarcated by either partial or full remission for at least 2 months or a switch to an episode of opposite polarity (e.g., Major Depressive Episode to Manic Episode)	O 1 O 3
	IF NO, SELECT ONE:	O Single Manic Episode<br>O No Pattern<br>O No Information

WITH POSTPARTUM ONSET FEMALE SUBJECTS ONLY

Have you ever had an episode of (DEPRESSION/MANIA/HYPO-MANIA/MIXED STATE/OWN EQUIVALENT) which started within a month of childbirth?	Has ever had a Depressed, Manic, Hypomanic or Mixed Episode with onset within 4 weeks postpartum	O ? O 1 O 3

CATATONIC FEATURES:

O History of Catatonic Features: This specifier applies if the subject has ever experienced any catatonic symptoms in his/her lifetime (see page B5).

O No History of Catatonic Features: This specifier is applied when the subject has never experienced any catatonic symptoms in his/her lifetime (see page B5).

O No Information

TREATMENT INDUCED:

O Only Treatment Induced Episodes: This specifier applies if the subject has only experienced treatment induced episodes in his/her lifetime (see page A26 and Medication Assessment in Overview).

O History of Treatment Induced Episodes: This specifier applies if the subject has ever experienced any treatment induced episodes in his/her lifetime (see page A26 and Medication Assessment in Overview).

O No History of Treatment Induced Episodes

O No Information

CHRONOLOGY

How old were you when you first began to have these experiences regularly?	DETERMINE AGE AT ONSET OF SCHIZOAFFECTIVE DISORDER	

SEASONAL PATTERN

IF THERE HAVE BEEN FEWER THAN TWO MAJOR DEPRESSIVE, MANIC OR HYPOMANIC EPISODES, CHECK HERE AND GO TO NEXT MODULE (D). O Check Here

IF UNKNOWN: Do the periods of (MOOD EPISODES) mostly seem to happen at the same time of the year, like fall or winter?	A. There has been a regular temporal relationship between the onset of either the Major Depressive, Manic, Hypomanic or Mixed Episodes in Bipolar I or Bipolar II Disorder and a particular time of year	O 1 O 3

	(e.g., regular appearance of the Major Depressive Episode in the fall or winter).<br><br>Note: Do not include cases in which there is an obvious effect of seasonal-related psychosocial stressors (e.g., regularly being unemployed every winter).	
IF UNKNOWN: Do the periods of (MOOD EPISODES) get better around the same time of year, like in the spring?	B. Full remissions (or a change of state from depression to mania/hypomania or mania/hypomania to depression) also occur at a characteristic time of the year (e.g., depression disappears in the spring).	O O<br>1 3
IF UNKNOWN: How many periods of (MOOD EPISODES) did you have during the past two years? How many of these occurred at the same time of the year?	C. In the last 2 years, two Major Depressive, Manic, Hypomanic or Mixed Episodes have occurred that demonstrate the temporal relationship defined in criteria A and B, and no non-seasonal episodes have occurred during that same period.	O O<br>1 3
IF UNKNOWN: About how many years can you recall having had periods of (MOOD EPISODES)? How often did they occur in (ALTERNATES OF SEASONS STATED) rather than in (SEASONS STATED)?	D. Seasonal Major Depressive Episodes (as described above) substantially outnumber the non-seasonal Major Depressive Episodes that may have occurred over the individual's lifetime.	O O<br>1 3

SCHIZOPHRENIFORM DISORDER

SCHIZOPHRENIA HAS BEEN RULED OUT BECAUSE THE DURATION IS LESS THAN SIX MONTHS.

	**SCHIZOPHRENIFORM DISORDER CRITERIA**	
	A. Criteria A, D, and E of Schizophrenia are met.	false
	B. An episode of the disorder (including prodromal, active and residual phases) lasts at least one month but less than six months.	O O<br>1 3
IF UNKNOWN: Were you taking any drugs or medicines during this time?<br><br>IF UNKNOWN: Were you physically ill at this time?	C. Not due to the direct physiological effects of a substance (e.g., a drug of abuse, medication) or to a general medical condition.<br><br>NOTE: CRITERION C WAS ADDED PER KELSOE CONVENTION. IF GENERAL MEDICAL CONDITION OR SUBSTANCE MAY BE ETIOLOGICLALY ASSOCIATED WITH PSYCHOTIC SYMPTOMS, GO TO *GMC/SUBSTANCE*, C16 AND RETURN HERE FOR RATING OF "1" OR "3".<br><br>REFER TO LIST OF GENERAL MEDICAL CONDITIONS AND SUBSTANCES, C3.<br><br>WHEN THE DIAGNOSIS IS MADE WITHOUT WAITING FOR RECOVERY, IT SHOULD BE	O O O O<br>? 1 2 3

QUALIFIED AS "PROVISIONAL".

CODE "2" IF THE EXPECTED RECOVERY HAS NOT YET OCCURRED. CODE "3" IF THERE HAS BEEN A FULL RECOVERY.

Presence of at least two of the following features that are generally associated with good prognosis:
DETERMINE WHETHER OR NOT GOOD PROGNOSTIC FEATURES ARE PRESENT.

1) onset of prominent psychotic symptoms within four weeks of first noticeable change in usual behavior or functioning	O<br>?	O<br>1	O<br>3
2) confusion or perplexity at the height of the psychotic episode	O<br>?	O<br>1	O<br>3
3) good premorbid social and occupational functioning	O<br>?	O<br>1	O<br>3
4) absence of blunted or flat affect	O<br>?	O<br>1	O<br>3

AT LEAST TWO GOOD PROGNOSTIC FEATURES CODED "3".

false

IF CODED "3", RECORD THIS UNDER FEATURES/SUBTYES ON THE DIAGNOSTIC SCORE SHEET.

SCHIZOPHRENIFORM DISORDER DIAGNOSTIC SPECIFIERS

LIFETIME SEVERITY:

(Additional questions regarding impairment may be necessary.)

- O Mild: (GAF = 61-80) Minimum symptom criteria are met or few, if any, symptoms in excess of those required to make the diagnosis and/or symptoms result in only minimal interference in normal functioning. Either mild disability is present or substantial or unusual effort is needed to function normally.
- O Moderate: (GAF = 51-60) Intermediate between "mild" and "severe."
- O Severe: (GAF lt;= 50) Several symptoms in excess of those required to make the diagnosis and/or symptoms result in only limited functioning in most aspects of life almost all the time.
- O No Information

ONSET

O History of Substance Induced Onset: This specifier applies if substance use may be etiologically associated with the onset of illness.

O No History of Substance Induced Onset: This specifier is applied when there has been no evidence that a substance was etiologically associated with the onset of illness.

O No Information

CHRONOLOGY

How old were you when you first began to have these experiences regularly?

DETERMINE AGE AT ONSET OF SCHIZOPHRENIFORM DISORDER

End of Schizophreniform Disorder

Continue with Mood Differential, Module D

DELUSIONAL DISORDER

SCHIZOPHRENIA, SCHIZOPHRENIFORM, AND SCHIZOAFFECTIVE DISORDERS HAVE BEEN RULED OUT.

NOTE: THE ORDER OF THE CRITERIA BELOW DIFFERS FROM THAT IN DSM-IV.

	**DELUSIONAL DISORDER CRITERIA**	
IF UNKNOWN: Has there ever been a time when you have been (DELUSIONAL) at the same time that you were (DEPRESSED/HIGH/IRRITABLE/OWN EQUIVALENT)?	D (1) There are no Major Depressive, Manic or Mixed Episodes that have occurred concurrently with delusions. NOTE: CODE "3" IF THERE HAVE NEVER BEEN ANY MAJOR DEPRESSIVE, MANIC, OR MIXED EPISODES OR IF ALL MOOD EPISODES OCCURRED AT TIMES OTHER THAN DURING DELUSIONAL PERIODS. CODE "1" IF THERE HAS BEEN A PERIOD OF OVERLAP WITH THE DELUSIONS.	O ? O 1 O 3
IF UNKNOWN: How much of the time that you have had (DELUSIONS) would you say you have also been (DEPRESSED/HIGH/IRRITABLE/ OWN EQUIVALENT)?	D (2) The total duration of all mood episodes occurring concurrently with delusions has been brief relative to the duration of the delusional periods. NOTE: CODE "1" IF SYMPTOMS MEETING CRITERIA FOR A MOOD EPISODE ARE PRESENT FOR A SUBSTANTIAL PORTION OF THE TOTAL DURATION OF THE DISTURBANCE.	O ? O 1 O 3
IF UNKNOWN: Have you only had (DELUSIONS) only at times when you were (DEPRESSED/ HIGH/OWN EQUIVALENT)?	Psychotic symptoms occur exclusively during Major Depressive, Manic, and Mixed Episodes.	O ? O 1 O 3
	A. Non-bizarre delusions (i.e., involving situations that occur in real life, such as being followed, poisoned, infected, loved at a distance, having a disease, or being deceived by one's spouse or lover) of at least one month's duration. NOTE: CODE "3" IF OTHER ACTIVE PHASE SYMPTOMS OF SCHIZOPHRENIA (E.G., HALLUCINATIONS) ARE NOT SIGNIFICANT (I.E., LAST LESS THAN ONE MONTH).	O ? O 1 O 3

	B. Has never met criterion A for Schizophrenia. Note: Tactile and olfactory hallucinations may be present in Delusional Disorder if they are related to the delusional theme.	O ? O 1 O 3

	C. Apart from the impact of the delusion(s) or its ramifications, functioning is not markedly impaired and behavior is not obviously odd or bizarre.	O ? O 1 O 3

IF UNKNOWN: Were you taking any drugs or medicines during this time? IF UNKNOWN: Were you physically ill at this time?	E. Not due to the direct physiological effects of a substance (e.g., a drug of abuse, a medication) or a general medical condition. IF GENERAL MEDICAL CONDITION OR SUBSTANCE MAY BE ETIOLOGICALLY ASSOCIATED WITH PSYCHOTIC SYMPTOMS, GO TO *GMC/SUBSTANCE* C21 AND RETURN HERE FOR RATING OF "1" OR "3" REFER TO LIST OF GENERAL MEDICAL CONDITIONS AND SUBSTANCES ON PAGE C3.	O ? O 1 O 3

	Check here to move to GMC/Substance section	O Check Here

DELUSIONAL DISORDER DIAGNOSTIC SPECIFIERS

LIFETIME SEVERITY:

(Additional questions regarding impairment may be necessary.)

O Mild: (GAF = 61-80) Minimum symptom criteria are met or few, if any, symptoms in excess of those required to make the diagnosis and/or symptoms result in only minimal interference in normal functioning. Either mild disability is present or substantial or unusual effort is needed to function normally.

O Moderate: (GAF = 51-60) Intermediate between "mild" and "severe."

O Severe: (GAF lt;= 50) Several symptoms in excess of those required to make the diagnosis and/or symptoms result in only limited functioning in most aspects of life almost all the time.

O No Information

CURRENT STATE:

O In Partial Remission: Symptoms are present but full criteria are not met, or there is a period without any significant symptoms lasting less than two months following the end of the episode.

O In Full Remission: During the past two months no significant signs or symptoms of the disturbance.

O Currently Meets Full Criteria: Full Criteria for an illness must be met at the time of the interview

O Currently Symptomatic: Symptoms are present but full criteria are not met. This differs from Partial Remission in that it does not immediately follow a period of illness (i.e., there was a symptom-free period of at least two months before the current symptoms began).

O No Information

FEATURES/SUBTYPES:

(Specify type on the basis of predominant theme of the delusion(s).)

O Persecutory: delusions that one (or someone to whom one is close) is being malevolently treated in some way

O Jealous: delusions that one's sexual partner is unfaithful

O Erotomanic: delusions that one is loved by another person, usually of higher status

O Somatic: delusions that one has some physical defect or general medical condition

O Grandiose: delusions of inflated worth, power, knowledge, identity, or special relationship to a deity or famous person

O Mixed: delusions characteristic of more than one of the above types but no one theme predominates

O Unspecified Type

O No Information

CHRONOLOGY

How old were you when you first began to have these experiences regularly?

DETERMINE AGE AT ONSET OF DELUSIONAL DISORDER

BRIEF PSYCHOTIC DISORDER

NOTE: ORDER OF CRITERIA DIFFERS FROM THAT IN DSM-IV.

BRIEF PSYCHOTIC DISORDER CRITERIA

A. Presence of one (or more) of the following symptoms:
1) delusions
2) hallucinations
3) disorganized speech (e.g., frequent derailment or incoherence)
4) grossly disorganized or catatonic behavior

O ? O 1 O 3

IF UNKNOWN: Were you taking any drugs or medicines during this time? IF UNKNOWN: Were you physically ill at this time?

C. (2) Not due to the direct physiological effects of a substance (e.g., a drug of abuse, a medication) or a general medical condition. IF GENERAL MEDICAL CONDITION OR SUBSTANCE MAY BE ETIOLOGICALLY ASSOCIATED WITH PSYCHOTIC SYMPTOMS, GO TO *GMC/SUBSTANCE*,C22 AND RETURN HERE FOR RATING OF "1" OR "3" REFER TO LIST ON PAGE C3

O ? O 1 O 3

Check here to move to GMC/Substance section

O Check Here

B. Duration of an episode of the disturbance is at least one day, but less than one month, with an eventual full return to premorbid level of functioning.

O ? O 1 O 3

C. (1) Not better accounted for by a Mood Disorder With psychotic features, Schizoaffective Disorder, or Schizophrenia. NOTE: CODE "3" IF NO MOOD EPISODES.

O ? O 1 O 3

BRIEF PSYCHOTIC DISORDER DIAGNOSTIC SPECIFIERS

LIFETIME SEVERITY:

(Additional questions regarding impairment may be necessary.)

- O Mild: (GAF = 61-80) Minimum symptom criteria are met or few, if any, symptoms in excess of those required to make the diagnosis and/or symptoms result in only minimal interference in normal functioning. Either mild disability is present or substantial or unusual effort is needed to function normally.
- O Moderate: (GAF = 51-60) Intermediate between "mild" and "severe."
- O Severe: (GAF lt;= 50) Several symptoms in excess of those required to make the diagnosis and/or symptoms result in only limited functioning in most aspects of life almost all the time.
- O No Information

CURRENT STATE:

- O In Partial Remission: Symptoms are present but full criteria are not met, or there is a period without any significant symptoms lasting less than two months following the end of the episode.
- O In Full Remission: During the past two months no significant signs or symptoms of the disturbance.
- O Currently Meets Full Criteria: Full Criteria for an illness must be met at the time of the interview
- O Currently Symptomatic: Symptoms are present but full criteria are not met. This differs from Partial Remission in that it does not immediately follow a period of illness (i.e., there was a symptom-free period of at least two months before the current symptoms began).
- O No Information

FEATURES/SUBTYPES:

(Specify type on the basis of predominant theme of the delusion(s).)

- O With Marked Stressor(s): This specifier may be noted if the psychotic symptoms develop shortly after and apparently in response to one or more events that, singly or together, wouild be markedly stressful to almost anyone in similar circumstances in that person's culture. The precipitating event(s) may be any major stress, such as the loss of a loved one or the psychological trauma of combat. Determining whether a specific stressor was a precipitant or a consequence of the illness may sometimes be clinically difficult. In such instances, the decision will depend on related factors such as the temporal relationship between the stressor and the onset of the symptoms, ancillary information from a spouse or friend about the level of functioning prior to the stressor, and history of similar responses to stressful events in the past.
- O Without Marked Stressor(s): This specifier may be noted if the psychotic symptoms are not apparently in response to events that would be markedly stressful to almost anyone in similar circumstances in the person's culture.
- O Unspecified
- O No Information

CHRONOLOGY

How old were you when you first began to have these experiences regularly?	DETERMINE AGE AT ONSET OF BRIEF PSYCHOTIC DISORDER	

WITH POSTPARTUM ONSET FEMALE SUBJECTS ONLY

Have you ever had an episode of (PSYCHOTIC SYMPTOMS/ OWN EQUIVALENT) which started within a month of childbirth?	Has ever had a psychotic period with onset within 4 weeks postpartum	O ? O 1 O 3

PSYCHOTIC DISORDER DUE TO A GENERAL MEDICAL CONDITION

IF SYMPTOMS NOT TEMPORALLY ASSOCIATED WITH A GENERAL MEDICAL CONDITION, CHECK HERE AND GO TO SUBSTANCE-INDUCED PSYCHOTIC DISORDER, C23. O Check Here

PSYCHOTIC DISORDER DUE TO A GENERAL MEDICAL CONDITION CRITERIA

CODE BASED ON INFORMATION ALREADY OBTAINED	A. Prominent hallucinations or delusions.	O ? O 1 O 3

Do you think your (DELUSIONS/HALLUCINATIONS) were in any way related to your (COMORBID GENERAL MEDICAL CONDITION)?<br><br>IF YES: Tell me how.<br><br>Did the (DELUSIONS/HALLUCINATIONS) start or get much worse only after (COMORBID GENERAL MEDICAL CONDITION) began?	B. There is evidence from the history, physical examination, or laboratory findings that the disturbance is the direct physiological consequence of a general medical condition.<br><br>THE FOLLOWING FACTORS SHOULD BE CONSIDERED AND SUPPORT THE CONCLUSION THAT THE GMC IS ETIOLOGIC TO THE PSYCHOTIC SYMPTOMS:<br><br>1) There is evidence from the literature of a well-established association between the GMC and psychotic symptoms.<br>2) There is a close temporal relationship between the course of the psychotic symptoms and the course of the general medical condition.<br>3) The psychotic symptoms are characterized by unusual presenting features (e.g., late age at onset).<br>4) The absence of alternative explanations.	O O O<br>? 1 3

CODE BASED ON INFORMATION ALREADY OBTAINED	C. The disturbance is not better accounted for by another mental disorder.	O O O<br>? 1 3

	D. The disturbance does not occur exclusively during the course of Delirium.	O O<br>1 3

PSYCHOTIC DISORDER DUE TO A GMC DIAGNOSTIC SPECIFIERS

LIFETIME SEVERITY:

(Additional questions regarding impairment may be necessary.)

O Mild: (GAF = 61-80) Minimum symptom criteria are met or few, if any, symptoms in excess of those required to make the diagnosis and/or symptoms result in only minimal interference in normal functioning. Either mild disability is present or substantial or unusual effort is needed to function normally.

O Moderate: (GAF = 51-60) Intermediate between "mild" and "severe."

O Severe: (GAF lt;= 50) Several symptoms in excess of those required to make the diagnosis and/or symptoms result in only limited functioning in most aspects of life almost all the time.

O No Information

CURRENT STATE:

O In Partial Remission: Symptoms are present but full criteria are not met, or there is a period without any significant symptoms lasting less than two months following the end of the episode.

O In Full Remission: During the past two months no significant signs or symptoms of the disturbance.

O Currently Meets Full Criteria: Full Criteria for an illness must be met at the time of the interview

O Currently Symptomatic: Symptoms are present but full criteria are not met. This differs from Partial Remission in that it does not immediately follow a period of illness (i.e., there was a symptom-free period of at least two months before the current symptoms began).

O No Information

FEATURES/SUBTYPES:

Indicate which type of symptom presentation predominates.

O With Delusions: This subtype is used if delusions are the predominant symptom.

O With Hallucinations: This subtype is used if hallucinations are the predominant symptom.

O With Delusions Hallucinations: Both delusions and hallucinations are equally predominant.

O Other or Unknown Type

O No Information

CHRONOLOGY

How old were you when you first began to have these experiences regularly?	DETERMINE AGE AT ONSET OF PSYCHOTIC DISORDER DUE TO A GMC	

SUBSTANCE-INDUCED PSYCHOTIC DISORDER

EPISODE BEING EVALUATED FOR SUBSTANCE INDUCED CRITERIA: (SELECT ONE)

O Schizophrenia

O Schizoaffective Disorder

O Schizophreniform

O Delusional Disorder

O Brief Psychotic Disorder

IF SYMPTOMS NOT TEMPORALLY ASSOCIATED WITH SUBSTANCE USE, CHECK HERE AND RETURN TO DISORDER BEING EVALUATED OR MODULE D, MOOD DIFFERENTIAL D1.

O Check Here

SUBSTANCE-INDUCED PSYCHOTIC DISORDER CRITERIA

CODE BASED ON INFORMATION ALREADY OBTAINED.	A. Prominent hallucinations or delusions.<br><br>NOTE: DO NOT INCLUDE HALLUCINATIONS IF THE PERSON HAS INSIGHT THAT THEY ARE SUBSTANCE-INDUCED.	O ? O 1 O 3
IF UNKNOWN: When did the (DELUSIONS/HALLUCINATIONS) begin? Were you already using (SUBSTANCE) or had you just stopped or cut down on its use?	B. There is evidence from the history, physical examination, or laboratory findings that either:<br><br>(1) the symptoms in A developed during, or within a month of, Substance Intoxication or Withdrawal, or<br><br>(2) medication use is etiologically related to the disturbance	O ? O 1 O 3

Do you think your (DELUSIONS/ HALLUCINA-TIONS) in any way relate to your (SUBSTANCE USE)?<br><br>IF YES: Tell me how.<br><br>ASK ANY OF THE FOLLOWING QUESTIONS AS NEEDED TO RULE OUT A NON-SUBSTANCE-INDUCED ETIOLOGY.<br><br>IF UNKNOWN: Which came first, the (SUBSTANCE USE) or the (PSYCHOTIC SYMPTOMS?)<br><br>IF UNKNOWN: Have you had a period of time when you stopped using (SUBSTANCE)?<br><br>IF YES: After you stopped using (SUBSTANCE) did the (DELUSIONS/HALLUCINATIONS) get better or were you still having (DELUSIONS/HALLUCINATIONS)?<br><br>IF UNKNOWN: How much of (SUBSTANCE) were you taking when you began to have (DELUSIONS/HALLUCINATIONS)?<br><br>IF UNKNOWN: Have you had any other episodes of (DELUSIONS/HALLUCINATIONS)?	C. The disturbance is not better accounted for by a Psychotic Disorder that is not substance-induced. Evidence that the symptoms are better accounted for by a Psychotic Disorder that is not substance-induced might include:<br><br>IF YES TO ONE OF THE FOLLOWING, CODE "1" FOR CRITERION C.<br><br>1) The psychotic symptoms precede the onset of the Substance Abuse or Dependence (or medication use).<br><br>2) The psychotic symptoms persist for a substantial period of time (e.g., about a month) after the cessation of acute withdrawal or severe intoxication.<br><br>3) The psychotic symptoms are substantially in excess of what would be expected given the type or amount of the substance used or the duration of use.<br><br>4) There is evidence suggesting the existence of an independent non-substance-induced Psychotic Disorder (e.g., a history of recurrent non-substance-related psychotic episodes).	O O O<br>? 1 3
	D. The disturbance does not occur exclusively during the course of Delirium.	O O<br>1 3

SUBSTANCE-INDUCED PSYCHOTIC DISORDER DIAGNOSTIC SPECIFIERS

LIFETIME SEVERITY:

(Additional questions regarding impairment may be necessary.)

O Mild: (GAF = 61-80) Minimum symptom criteria are met or few, if any, symptoms in excess of those required to make the diagnosis and/or symptoms result in only minimal interference in normal functioning. Either mild disability is present or substantial or unusual effort is needed to function normally.

O Moderate: (GAF = 51-60) Intermediate between "mild" and "severe."

O Severe: (GAF lt;= 50) Several symptoms in excess of those required to make the diagnosis and/or symptoms result in only limited functioning in most aspects of life almost all the time.

O No Information

CURRENT STATE:

O In Remission: No significant signs or symptoms of the disturbance.

O Currently Meets Full Criteria: Full Criteria for an illness must be met at the time of the interview.

O No Information

FEATURES/SUBTYPES:

Indicate which type of symptom presentation predominates.

O With Delusions: This subtype is used if delusions are the predominant symptom.

O With Hallucinations: This subtype is used if hallucinations are the predominant symptom.

O With Delusions Hallucinations: Both delusions and hallucinations are equally predominant.

O Other or Unknown Type

O No Information

ONSET:

O With Onset During Intoxication: This specifier should be used if criteria for intoxication with the substance are met and the symptoms develop during the intoxication syndrome.

O With Onset During Withdrawal: This specifier should be used if criteria for withdrawal from the substance are met and the symptoms develop during, or shortly after, a withdrawal syndrome.

O No Information

CHRONOLOGY

How old were you when you first began to have these experiences regularly?	DETERMINE AGE AT ONSET OF SUBSTANCE INDUCED PSYCHOTIC DISORDER	

PSYCHOTIC DISORDER NOT OTHERWISE SPECIFIED

NOTE: IF PSYCHOTIC SYMPTOMS HAVE BEEN PRESENT BUT ARE NOT DIAGNOSTICALLY SIGNIFICANT, CODE "1" (I.E., DO NOT DIAGNOSE PSYCHOTIC DISORDER NOS). ONE EXAMPLE IS THE RECURRENT "HALLUCINATIONS" OF NAME BEING CALLED.	This category should be used to diagnose psychotic symptomatology (i.e., delusions, hallucinations, disorganized speech, grossly disorganized or catatonic behavior) about which there is inadequate information to make a specific diagnosis, or about which there is contradictory information, or disorders with psychotic symptoms that do not meet the criteria for any specific psychotic disorder defined previously in the module.	O 1 O 3

PSYCHOTIC DISORDER NOS DIAGNOSTIC SPECIFIERS

LIFETIME SEVERITY:

(Additional questions regarding impairment may be necessary.)

O Mild: (GAF = 61-80) Minimum symptom criteria are met or few, if any, symptoms in excess of those required to make the diagnosis and/or symptoms result in only minimal interference in normal functioning. Either mild disability is present or substantial or unusual effort is needed to function normally.

O Moderate: (GAF = 51-60) Intermediate between "mild" and "severe."

O Severe: (GAF lt;= 50) Several symptoms in excess of those required to make the diagnosis and/or symptoms result in only limited functioning in most aspects of life almost all the time.

O No Information

CURRENT STATE:

O In Partial Remission: Symptoms are present but full criteria are not met, or there is a period without any significant symptoms lasting less than two months following the end of the episode.

O In Full Remission: During the past two months no significant signs or symptoms of the disturbance.

O Currently Meets Full Criteria: Full Criteria for an illness must be met at the time of the interview

O Currently Symptomatic: Symptoms are present but full criteria are not met. This differs from Partial Remission in that it does not immediately follow a period of illness (i.e., there was a symptom-free period of at least two months before the current symptoms began).

O No Information

FEATURES/SUBTYPES:

Indicate which type of symptom presentation predominates.

- O Postpartum psychosis that does not meet criteria for Mood Disorder With Psychotic Features, Brief Psychotic Disorder, Psychotic Disorder Due to a General Medical Condition, or a Substance-Induced Psychotic Disorder.
- O Psychotic symptoms that have lasted for less than one month but have not yet remitted so that the criteria for Brief Psychotic Disorder are not met.
- O Persistent auditory hallucinations in the absence of any other features.
- O Persistent non-bizarre delusions with periods of overlapping mood episodes that have been present for a substantial portion of the delusional disturbance.
- O Ambiguous situations in which the clinician has concluded that a psychotic disorder is present but is unable to determine whether it is primary, due to a general medical condition, or substance-induced.
- O Other (describe):
- O No Information

CHRONOLOGY

How old were you when you first began to have these experiences regularly?	DETERMINE AGE AT ONSET OF PSYCHOTIC DISORDER NOS	

***WITH POSTPARTUM ONSET* FEMALE SUBJECTS ONLY**

Have you ever had an episode of (PSYCHOTIC SYMPTOMS/ OWN EQUIVALENT) which started within a month of childbirth?	Has ever had a psychotic period with onset within 4 weeks postpartum	O ? O 1 O 3

Module D: Mood Differential

SUICIDAL IDEATION SUMMARY

SUICIDAL IDEATION SUMMARY FOR ALL SUBJECTS

IF UNCLEAR: Have you ever thought about suicide?	SUBJECT HAS EVER HAD SUICIDAL IDEATION	O ? O 1 O 3
IF UNKNOWN: Have you ever attempted suicide? IF YES: How many times?	Total number of suicide attempts	

IF THERE HAVE NEVER BEEN ANY CLINICALLY SIGNIFICANT MOOD SYMPTOMS, CHECK HERE AND GO TO THE NEXT MODULE (E).

O Check Here

BIPOLAR I DISORDER

BIPOLAR I DISORDER DIFFERENTIAL CRITERIA

History of one or more Manic or Mixed Episodes — O 1 O 3

NOTE: A MIXED EPISODE IS DEFINED AS AN EPISODE IN WHICH THE CRITERIA ARE MET FOR A MANIC EPISODE AND A MAJOR DEPRESSIVE EPISODE (EXCEPT FOR DURATION) NEARLY EVERY DAY DURING AT LEAST A ONE-WEEK PERIOD

At least one Manic or Mixed Episode is not due to the direct physiological effects of a general medical condition or substance use — O 1 O 3

NOTE: MANIC-LIKE EPISODES THAT ARE CLEARLY CAUSED BY SOMATIC ANTIDEPRESSANT TREATMENT (E.G., MEDICATION, ECT, LIGHT THERAPY) SHOULD COUNT TOWARD A DIAGNOSIS OF BIPOLAR I DISORDER (KELSOE CONVENTION)

At least one Depressed, Manic or Mixed Episode is not better accounted for by Schizoaffective Disorder and is not superimposed on Schizophrenia, Schizophreniform Disorder, Delusional Disorder, or Psychotic Disorder NOS	O<br>1	O<br>3

BIPOLAR I DISORDER DIAGNOSTIC SPECIFIERS

LIFETIME SEVERITY:

(Additional questions regarding impairment may be necessary.)

O Mild: (GAF = 61-80) Minimum symptom criteria are met for a Manic Episode or few, if any, symptoms in excess of those required to make the diagnosis and symptoms result in only minor impairment in occupational functioning or in usual social activities or relationships with others. Either mild disability is present or substantial or unusual effort is needed to function normally.

O Moderate: (GAF = 51-60) Extreme increase in activity or impairment in judgment; intermediate between "mild" and "severe."

O Severe: (GAF = 50) Several symptoms in excess of those required to make the diagnosis, and symptoms markedly interfere with occupational functioning or with usual social activities or relationships with others. Almost continual supervision is required during many episodes in order to prevent physical harm to self or others.

O No Information

PSYCHOTIC FEATURES:

(This rating refers to Bipolar I Disorder over the subject's lifetime. Psychotic symptom ratings in Module A refer to the actual depression and manic episodes as opposed to the diagnosis as a whole.)

O Mood-Congruent Psychotic Features: Delusions or hallucinations whose content is entirely consistent with either the typical manic themes of inflated worth, power, knowledge, identity, or special relationship to a deity or famous person, or the typical depressive themes of personal inadequacy, guilt, disease, death, nihilism, or deserved punishment.

O Mood-Incongruent Psychotic Features: Delusions or hallucinations whose content does not involve either typical manic themes of inflated worth, power, knowledge, identity, or special relationship to a deity or famous person or typical depressive themes of personal inadequacy, guilt, disease, death, nihilism, or deserved punishment. Included are such symptoms as persecutory delusions (not directly related to grandiose ideas/themes or to depressivethemes), thought insertion, thought broadcasting, and delusions of being controlled.

O History of Psychotic Features, Unspecified

O No History of Psychotic Features

O No Information

CURRENT STATE:

O In Partial Remission: Symptoms of a Manic, Mixed, or Major Depressive Episode are present but full criteria are not met, or there is a period without any significant symptoms of a Manic, Mixed, or Major Depressive Episode lasting less than two months following the end of the episode.

O In Full Remission: During the past two months no significant signs or symptoms of the disturbance.

O Currently Meets Full Criteria: Full Criteria for an illness must be met at the time of the interview.

O Currently Symptomatic: Symptoms are present but full criteria are not met. This differs from Partial Remission in that it does not immediately follow a period of illness (i.e., there was a symptom-free period of at least two months before the current symptoms began).

O No Information

LIFETIME COURSE:

O With Full Interepisode Recovery: This specifier is used when full remission is attained between most Mood Episodes for at least two months.

O Without Full Interepisode Recovery: This specifier is used when full remission is not attained between most Mood Episodes.

O Chronic: Continuously met criteria for a minimum of two years since onset of illness

O N/A: Only one episode

O No Information

MOST RECENT EPISODE:

(Indicate type of current (or most recent) episode)

O Hypomanic

O Manic

O Mixed

O Major Depressive

O Unspecified (i.e., criteria, except for duration, are currently met for a Manic, Hypomanic, Major Depressive, or Mixed Episode)

O No Information

TREATMENT INDUCED:

(Use the Medication Assessment Form (from Overview) and information obtained in Module A to chose one of the following specifiers.)

O Only Treatment Induced Mania

O History of Treatment Induced Mania/Hypomania

O No History of Treatment Induced Mania/Hypomania

O No Information

SPECIFIC PATTERNS: RAPID CYCLING

IF UNKNOWN: How many times in the past have you had periods of being (MANIC/HIGH /IRRITABLE/ OWN WORDS) and periods of being (DEPRESSED/ OWN WORDS)?	At least four episodes of a mood disturbance in a 12 month period that met criteria for a Depressive, Manic, Mixed, or Hypomanic Episode.	O 1 O 3
	Note: Episodes are demarcated by either partial or full remission for at least 2 months or a switch to an episode of opposite polarity (e.g., Major Depressive Episode to Manic Episode)	O Brief Mania/s Only (2-4 days)<br>O Single Manic Episode<br>O No/Unspecified Pattern<br>O No Information

SEASONAL PATTERN

IF THERE HAVE BEEN FEWER THAN TWO MAJOR DEPRESSIVE, MANIC, OR HYPOMANIC EPISODES, CHECK HERE AND GO TO *CYCLOTHYMIC DISORDER*.

O Check Here

IF UNKNOWN: Do the periods of (DEPRESSED MOOD/ MANIA) mostly seem to happen at the same time of the year, like fall or winter?	A. There has been a regular temporal relationship between the onset of either the Major Depressive, Manic, Hypomanic or Mixed Episodes in Bipolar I or Bipolar II Disorder and a particular time of year (e.g., regular appearance of the Major Depressive Episode in the fall or winter).<br><br>NOTE: DO NOT INCLUDE CASES IN WHICH THERE IS AN OBVIOUS EFFECT OF SEASONAL-RELATED PSYCHOSOCIAL STRESSORS (E.G., REGULARLY BEING UNEMPLOYED EVERY WINTER)	O 1 O 3
IF UNKNOWN: Do the periods of (DEPRESSED MOOD/ MANIA) get better around the same time of year, like in the spring?	B. Full remissions (or a change of state from depression to mania/hypomania or mania/hypomania to depression) also occur at a characteristic time of the year (e.g., depression disappears in the spring).	O 1 O 3
IF UNKNOWN: How many periods of (DEPRESSION/ MANIA] did you have during the past two years? How many of these occurred at the same time of the year?	C. In the last 2 years, two Major Depressive, Manic, Hypomanic or Mixed Episodes have occurred that demonstrate the temporal relationship defined in criteria A and B, and no non-seasonal episodes have occurred during that same period.	O 1 O 3
IF UNKNOWN: About how many years can you recall having had periods of (DEPRESSION)? How often did they occur in (ALTERNATES OF SEASONS STATED) rather than in (SEASONS STATED)?	D. Seasonal Major Depressive Episodes (as described above) substantially outnumber the non-seasonal Major Depressive Episodes that may have occurred over the individual's lifetime	O 1 O 3
	SEASONAL PATTERN CRITERIA A, B, C AND D ARE ALL CODED "3"	false

BIPOLAR II DISORDER

BIPOLAR II DISORDER DIFFERENTIAL CRITERIA

	History of one or more Hypomanic Episodes which is not due to the direct physiological effects of a general medical condition or substance use<br><br>NOTE: HYPOMANIC-LIKE EPISODES THAT ARE CLEARLY CAUSED BY SOMATIC ANTIDEPRESSANT TREATMENT (E.G., MEDICATION, ECT, LIGHT THERAPY) SHOULD COUNT TOWARD A DIAGNOSIS OF BIPOLAR II DISORDER.	O 1 O 3
	At least one Major Depressive Episode not due to the direct physiological effects of a general medical condition or substance use	O 1 O 3
	There have never been any Manic or Mixed Episodes<br><br>NOTE: CODE "3" IF NEVER MANIC OR MIXED.	O 1 O 3

IF CODING "1", CONSIDER RETURNING TO BIPOLAR I DISORDER. IF FULL CRITERIA ARE NOT MET FOR MANIC OR MIXED EPISODE, GO TO BIPOLAR DISORDER NOS, D9.

The mood symptoms are not better accounted for by Schizoaffective Disorder and are not superimposed on Schizophrenia, Schizophreniform Disorder, Delusional Disorder, or Psychotic Disorder Not Otherwise Specified	O<br>1	O<br>3

BIPOLAR II DISORDER DIAGNOSTIC SPECIFIERS

LIFETIME SEVERITY:

(Additional questions regarding impairment may be necessary.)

O Mild: (GAF = 61-80) Minimum symptom criteria are met for a Hypomanic Episode or few, if any, symptoms in excess of those required to make the diagnosis and symptoms result in only minor impairment in occupational functioning or in usual social activities or relationships with others. Either mild disability is present or substantial or unusual effort is needed to function normally.

O Moderate: (GAF = 51-60) Extreme increase in activity or impairment in judgment; intermediate between "mild" and "severe."

O Severe: (GAF = 50) Several symptoms in excess of those required to make the diagnosis, and symptoms markedly interfere with occupational functioning or with usual social activities or relationships with others. Almost continual supervision is required during many episodes in order to prevent physical harm to self or others.

O No Information

PSYCHOTIC FEATURES:

(This rating refers to Bipolar II Disorder over the subject's lifetime. Psychotic symptom ratings in Module A refer to the actual depression and manic episodes as opposed to the diagnosis as a whole.)

O Mood-Congruent Psychotic Features: Delusions or hallucinations whose content is entirely consistent with either the typical manic themes of inflated worth, power, knowledge, identity, or special relationship to a deity or famous person, or the typical depressive themes of personal inadequacy, guilt, disease, death, nihilism, or deserved punishment.

O Mood-Incongruent Psychotic Features: Delusions or hallucinations whose content does not involve either typical manic themes of inflated worth, power, knowledge, identity, or special relationship to a deity or famous person or typical depressive themes of personal inadequacy, guilt, disease, death, nihilism, or deserved punishment. Included are such symptoms as persecutory delusions (not directly related to grandiose ideas/themes or to depressivethemes), thought insertion, thought broadcasting, and delusions of being controlled.

O History of Psychotic Features, Unspecified

O No History of Psychotic Features

O No Information

CURRENT STATE:

O In Partial Remission: Symptoms of a Hypomanic or Major Depressive Episode are present but full criteria are not met, or there is a period without any significant symptoms of a Hypomanic or Major Depressive Episode lasting less than two months following the end of the episode.

O In Full Remission: During the past two months no significant signs or symptoms of the disturbance.

O Currently Meets Full Criteria: Full Criteria for an illness must be met at the time of the interview.

O Currently Symptomatic: Symptoms are present but full criteria are not met. This differs from Partial Remission in that it does not

immediately follow a period of illness (i.e., there was a symptom-free period of at least two months before the current symptoms began).

O No Information

LIFETIME COURSE:

O With Full Interepisode Recovery: This specifier is used when full remission is attained between most Mood Episodes for at least two months.

O Without Full Interepisode Recovery: This specifier is used when full remission is not attained between most Mood Episodes.

O Chronic: Continuously met criteria for a minimum of two years since onset of illness

O N/A: Only one episode

O No Information

MOST RECENT EPISODE:

(Indicate type of current (or most recent) episode)

O Hypomanic

O Major Depressive

O Unspecified: (i.e., criteria, except for duration, are currently met for a Manic, Hypomanic, Major Depressive, or Mixed Episode)

O No Information

TREATMENT INDUCED:

(Use the Medication Assessment Form (from Overview) and information obtained in Module A to chose one of the following specifiers.)

O Only Treatment Induced Hypomania

O History of Treatment Induced Hypomania

O No History of Treatment Induced Hypomania

O No Information

SPECIFIC PATTERNS: RAPID CYCLING

IF UNKNOWN: How many times in the past have you had periods of being (MANIC/HIGH/IRRITABLE/ OWN WORDS) and periods of being (DEPRESSED/ OWN WORDS)?	At least four episodes of a mood disturbance in a 12 month period that met criteria for a Depressive, Manic, Mixed, or Hypomanic Episode.	O 1 O 3

Note: Episodes are demarcated by either partial or full remission for at least 2 months or a switch to an episode of opposite polarity (e.g., Major Depressive Episode to Manic Episode)

O Brief Hypomania Only (2-4 days)

O Single Hypomanic Episode

O No Pattern

O No Information

SEASONAL PATTERN

IF THERE HAVE BEEN FEWER THAN TWO MAJOR DEPRESSIVE OR HYPOMANIC EPISODES, CHECK HERE AND GO TO *CYCLOTHYMIC DISORDER*

O Check Here

IF UNKNOWN: Do the periods of (DEPRESSED MOOD/ HYPOMANIA) mostly seem to happen at the same time of the year, like fall or winter?	A. There has been a regular temporal relationship between the onset of either the Major Depressive or Hypomanic Episodes in Bipolar II Disorder and a particular time of year (e.g., regular appearance of the Major Depressive Episode in the fall or	O 1 O 3

	winter).<br><br>NOTE: DO NOT INCLUDE CASES IN WHICH THERE IS AN OBVIOUS EFFECT OF SEASONAL-RELATED PSYCHOSOCIAL STRESSORS (E.G., REGULARLY BEING UNEMPLOYED EVERY WINTER)	
IF UNKNOWN: Do the periods of (DEPRESSED MOOD/ HYPOMANIA) get better around the same time of year, like in the spring?	B. Full remissions (or a change of state from depression to hypomania or hypomania to depression) also occur at a characteristic time of the year (e.g., depression disappears in the spring).	O O<br>1 3
IF UNKNOWN: How many periods of (DEPRESSION/ HYPOMANIA) did you have during the past two years? How many of these occurred at the same time of the year?	C. In the last 2 years, two Major Depressive or Hypomanic Episodes have occurred that demonstrate the temporal relationship defined in criteria A and B, and no non-seasonal episodes have occurred during that same period.	O O<br>1 3
IF UNKNOWN: About how many years can you recall having had periods of (DEPRESSION)? How often did they occur in (ALTERNATES OF SEASONS STATED) rather than in (SEASONS STATED)?	D. Seasonal Major Depressive Episodes (as described above) substantially outnumber the non-seasonal Major Depressive Episodes that may have occurred over the individual's lifetime	O O<br>1 3
	SEASONAL PATTERN CRITERIA A, B, C AND D ARE ALL CODED "3"	false

BIPOLAR DISORDER NOT OTHERWISE SPECIFIED (NOS)

BIPOLAR DISORDER NOT OTHERWISE SPECIFIED (NOS) DIFFERENTAIL CRITERIA

	Clinically significant Manic or Hypomanic symptoms that are not accounted for by a psychotic disorder (e.g., Schizophrenia) and do not meet criteria for any specific Bipolar Disorder	O O<br>1 3
IF UNKNOWN: Just before this began, were you physically ill?<br><br>IF YES: What did the doctor say?<br><br>IF UNKNOWN: Just before this began, were you taking any medications?<br><br>IF YES: Any change in the amount you were taking?<br><br>IF UNKNOWN: Just before this began, were you drinking or using any street drugs?	Not due to the direct physiological effects of a substance (i.e., a drug of abuse, medication) or to a general medical condition IF GENERAL MEDICAL CONDITION OR SUBSTANCE MAY BE ETIOLOGIC-ALLY ASSOCIATED WITH MANIC OR HYPOMANIC SYMPTOMS, GO TO *SUBSTANCE/GMC*, A51. AND RETURN HERE TO MAKE A RATING OF "1" OR "3."<br><br>Etiological general medical conditions include: degenerative neurological illness (e.g., Parkinson's disease, Huntington's disease), cerebrovascular disease, metabolic conditions (e.g., B-12 deficiency), endocrine conditions (e.g. hyperthyroidism), autoimmune conditions (e.g., systemic lupus erythematosus), viral or other infections (e.g., hepatitis, mononucleosis, HIV), and certain cancers (e.g., carcinoma of the pancreas)<br><br>Etiological substances include: alcohol,	O O O<br>? 1 3

amphetamines, cocaine, hallucinogens, inhalants, opioids, phencyclidine, sedatives, hypnotics, and anxiolytics. Medications include psychotropic medications (e.g., antidepressants), corticosteroids, anabolic steroids, isoniazid, antiparkinson medication (e.g., levodopa), and sympathomimetics/ decongestants.

BIPOLAR DISORDER NOS DIAGNOSTIC SPECIFIERS

LIFETIME SEVERITY:

(Additional questions regarding impairment may be necessary.)

O Mild: (GAF = 61-80) Minimum symptom criteria are met or few, if any, symptoms in excess of those required to make the diagnosis and/or symptoms result in only minimal interference in normal functioning. Either mild disability is present or substantial or unusual effort is needed to function normally.

O Moderate: (GAF = 51-60) Intermediate between "mild" and "severe."

O Severe: (GAF = 50) Several symptoms in excess of those required to make the diagnosis and/or symptoms result in only limited functioning in most aspects of life almost all the time.

O No Information

PSYCHOTIC FEATURES:

(This rating refers to Bipolar Disorder NOS over the subject's lifetime. Psychotic symptom ratings in Module A refer to the actual depression and manic episodes as opposed to the diagnosis as a whole.)

O Mood-Congruent Psychotic Features: Delusions or hallucinations whose content is entirely consistent with either the typical manic themes of inflated worth, power, knowledge, identity, or special relationship to a deity or famous person, or the typical depressive themes of personal inadequacy, guilt, disease, death, nihilism, or deserved punishment.

O Mood-Incongruent Psychotic Features: Delusions or hallucinations whose content does not involve either typical manic themes of inflated worth, power, knowledge, identity, or special relationship to a deity or famous person or typical depressive themes of personal inadequacy, guilt, disease, death, nihilism, or deserved punishment. Included are such symptoms as persecutory delusions (not directly related to grandiose ideas/themes or to depressivethemes), thought insertion, thought broadcasting, and delusions of being controlled.

O History of Psychotic Features, Unspecified

O No History of Psychotic Features

O No Information

CURRENT STATE:

- O In Partial Remission: Symptoms are present but full criteria are not met, or there is a period without any significant symptoms lasting less than two months following the end of the episode.
- O In Full Remission: During the past two months no significant signs or symptoms of the disturbance.
- O Currently Meets Full Criteria: Full Criteria for an illness must be met at the time of the interview.
- O Currently Symptomatic: Symptoms are present but full criteria are not met. This differs from Partial Remission in that it does not immediately follow a period of illness (i.e., there was a symptom-free period of at least two months before the current symptoms began).
- O No Information

Features/Subtypes

Indicate which type of symptom presentation predominates.

- O Very rapid alternation (over days) between manic symptoms and depressive symptoms that do not meet minimum duration criteria for a Manic Episode or a Major Depressive Episode
- O Recurrent Hypomanic Episodes without intercurrent depressive symptoms
- O A Manic or Mixed Episode Superimposed on Delusional Disorder, residual Schizphrenia, or Psychotic Disorder Not Otherwise Specified
- O Hypomanic Episodes, along with chronic depressive symptoms, that are too infrequent to qualify for a diagnosis of Cyclothymic Disorder
- O Ambiguous situations in which the clinician has concluded that a psychotic disorder is present but is unable to determine whether it is primary, due to a general medical condition, or substance-induced.
- O Other (describe):
- O No Information

CHRONOLOGY

How old were you when you first began to have (SYMPTOMS OF BIPOLAR DISORDER NOS) regularly?	Age at onset of Bipolar Disorder NOS (CODE 99 IF UNKNOWN)	
IF UNCLEAR: During the past month how many periods of (SYMPTOMS OF HYPERTHYMIC TEMPERAMENT) have you had?	Has met symptomatic criteria for Hyperthymic Temperament during past month	O ? O 1 O 3
When did you last have any (SYMPTOMS OF HYPERTHYMIC TEMPERAMENT)?	Number of months prior to interview when last had a symptom of Hyperthymic Temperament	
End of Bipolar NOS	Continue below with Cyclothymic Disorder	

CYCLOTHYMIC DISORDER

CYCLOTHYMIC DISORDER DIFFERENTIAL CRITERIA

No Major Depressive Episode, Manic Episode, or Mixed Episode has been present during the first two years of the disturbance — O 1 O 3

NOTE: AFTER THE INITIAL TWO YEARS (1 YEAR IN CHILDREN OR ADOLESCENTS) OF CYCLOTHYMIC DISORDER, THERE MAY BE SUPERIMPOSED MANIC OR MIXED EPISODES (IN WHICH CASE BOTH BIPOLAR I DISORDER AND CYCLOTHYMIC DISORDER MAY BE DIAGNOSED) OR MAJOR DEPRESSIVE EPISODES (IN WHICH CASE BOTH BIPOLAR II DISORDER AND CYCLOTHYMIC DISORDER MAY BE DIAGNOSED).

The symptoms are not due to the direct physiological effects of a substance (e.g., drug of abuse, a medication) or a general medical condition (e.g., hypothyroidism).	O O<br>1 3

The symptoms are not better accounted for by Schizoaffective Disorder and are not superimposed on Schizophrenia, Schizophreniform Disorder, Delusional Disorder, or Psychotic Disorder Not Otherwise Specified.	O O<br>1 3

CYCLOTHYMIC DISORDER DIAGNOSTIC SPECIFIERS

LIFETIME SEVERITY:

(Additional questions regarding impairment may be necessary.)

O Mild: (GAF = 61-80) Minimum symptom criteria are met or few, if any, symptoms in excess of those required to make the diagnosis and/or symptoms result in only minimal interference in normal functioning. Either mild disability is present or substantial or unusual effort is needed to function normally.

O Moderate: (GAF = 51-60) Intermediate between "mild" and "severe."

O Severe: (GAF = 50) Several symptoms in excess of those required to make the diagnosis and/or symptoms result in only limited functioning in most aspects of life almost all the time.

O No Information

PSYCHOTIC FEATURES:

(This rating refers to Cyclothymic Disorder over the subject's lifetime. Psychotic symptom ratings in Module A refer to the actual mood symptoms as opposed to the diagnosis as a whole.)

O Mood-Congruent Psychotic Features: Delusions or hallucinations whose content is entirely consistent with either the typical manic themes of inflated worth, power, knowledge, identity, or special relationship to a deity or famous person, or the typical depressive themes of personal inadequacy, guilt, disease, death, nihilism, or deserved punishment.

O Mood-Incongruent Psychotic Features: Delusions or hallucinations whose content does not involve either typical manic themes of inflated worth, power, knowledge, identity, or special relationship to a deity or famous person or typical depressive themes of personal inadequacy, guilt, disease, death, nihilism, or deserved punishment. Included are such symptoms as persecutory delusions (not directly related to grandiose ideas/themes or to depressivethemes), thought insertion, thought broadcasting, and delusions of being controlled.

O History of Psychotic Features, Unspecified

O No History of Psychotic Features

O No Information

CURRENT STATE:

O In Partial Remission: Symptoms are present but full criteria are not met, or there is a period without any significant symptoms lasting less than two months following the end of the episode.

O In Full Remission: During the past two months no significant signs or symptoms of the disturbance.

O Currently Meets Full Criteria: Full Criteria for an illness must be met at the time of the interview.

O Currently Symptomatic: Symptoms are present but full criteria are not met. This differs from Partial Remission in that it does not immediately follow a period of illness (i.e., there was a symptom-free period of at least two months before the current symptoms began).

O No Information

LIFETIME COURSE:

O With Full Interepisode Recovery: This specifier is used when full remission is attained between most Mood Episodes for at least two months.

O Without Full Interepisode Recovery: This specifier is used when full remission is not attained between most Mood Episodes.

O Chronic: Continuously met criteria for a minimum of two years since onset of illness

O N/A: Only one episode

O No Information

TREATMENT INDUCED:

Use the Medication Assessment Form (from Overview) and information obtained in Module A to chose one of the following specifiers.)

O Only Treatment Induced Cycling

O History of Treatment Induced Cycling

O No History of Treatment Induced Cycling

O No Information

CHRONOLOGY

How old were you when you first began to have (SYMPTOMS OF CYCLOTHYMIC DISORDER) regularly?	Age at onset of Cyclothymic Disorder (CODE 99 IF UNKNOWN)	
IF UNCLEAR: During the past month how many periods of (SYMPTOMS OF CYCLOTHYMIC DISORDER) have you had?	Has met symptomatic criteria for Cyclothymic Disorder during past month	O ? O 1 O 3
When did you last have any (SYMPTOMS OF CYCLOTHYMIC DISORDER)?	Number of months prior to interview when last had a symptom of Cyclothymic Disorder	
End of Cyclothymic Disorder	Continue below with Major Depressive Disorder	

MAJOR DEPRESSIVE DISORDER

MAJOR DEPRESSIVE DISORDER DIFFERENTIAL CRITERIA

At least one Major Depressive Episode that is not due to the direct physiological effects of a general medical condition or substance use — O 1 O 3

At least one Major Depressive Episode is not better accounted for by Schizoaffective Disorder and is not superimposed on Schizophrenia, Schizophreniform Disorder, Delusional Disorder, or Psychotic Disorder Not Otherwise Specified — O 1 O 3

Has never had any Manic, Mixed, or unequivocal Hypomanic Episodes — O 1 O 3

NOTE: THIS EXCLUSION DOES APPLY IF ALL OF THE MANIC-LIKE, MIXED-LIKE, OR HYPOMANIC-LIKE EPISODES ARE TREATMENT INDUCED. IF SO, CODE "1" AND RECONSIDER BPI, BPII, BP NOS. IF FULL CRITERIA ARE NOT MET, GO TO MOOD DISORDER NOS, D27. THIS EXCLUSION DOES NOT APPLY IF ALL OF THE MANIC-LIKE, MIXED-LIKE, OR HYPOMANIC-LIKE EPISODES ARE SUBSTANCE INDUCED OR ARE DUE TO THE DIRECT PHYSIOLOGICAL EFFECTS OF A GENERAL MEDICAL CONDITION (CODE "3").

MAJOR DEPRESSIVE DISORDER DIAGNOSTIC SPECIFIERS

LIFETIME SEVERITY:

(Additional questions regarding impairment may be necessary.)

- O Mild: (GAF = 61-80) Minimum symptom criteria are met for a Depressive Episode or few, if any, symptoms in excess of those required to make the diagnosis and symptoms result in only minor impairment in occupational functioning or in usual social activities or relationships with others. Either mild disability is present or substantial or unusual effort is needed to function normally.
- O Moderate: (GAF = 51-60) Intermediate between "mild" and "severe."
- O Severe: (GAF = 50) Several symptoms in excess of those required to make the diagnosis, and symptoms markedly interfere with occupational functioning or with usual social activities or relationships with others.
- O No Information

PSYCHOTIC FEATURES:

(This rating refers to Major Depressive Disorder over the subject's lifetime. Psychotic symptom ratings in Module A refer to the actual depressive episodes as opposed to the diagnosis as a whole.)

- O Mood-Congruent Psychotic Features: Delusions or hallucinations whose content is entirely consistent with either the typical manic themes of inflated worth, power, knowledge, identity, or special relationship to a deity or famous person, or the typical depressive themes of personal inadequacy, guilt, disease, death, nihilism, or deserved punishment.
- O Mood-Incongruent Psychotic Features: Delusions or hallucinations whose content does not involve either typical manic themes of inflated worth, power, knowledge, identity, or special relationship to a deity or famous person or typical depressive themes of personal inadequacy, guilt, disease, death, nihilism, or deserved punishment. Included are such symptoms as persecutory delusions (not directly related to grandiose ideas/themes or to depressivethemes), thought insertion, thought broadcasting, and delusions of being controlled.
- O History of Psychotic Features, Unspecified
- O No History of Psychotic Features
- O No Information

CURRENT STATE:

- O In Partial Remission: Symptoms of a Major Depressive Episode are present but full criteria are not met, or there is a period without any significant symptoms of a Major Depressive Episode lasting less than two months following the end of the episode.
- O In Full Remission: During the past two months no significant signs or symptoms of the disturbance.
- O Currently Meets Full Criteria: Full Criteria for an illness must be met at the time of the interview
- O Currently Symptomatic: Symptoms are present but full criteria are not met. This differs from Partial Remission in that it does not immediately follow a period of illness (i.e., there was a symptom-free period of at least two months before the current symptoms began).
- O No Information

LIFETIME COURSE:

- O With Full Interepisode Recovery: This specifier is used when full remission is attained between most Mood Episodes for at least two months.
- O Without Full Interepisode Recovery: This specifier is used when full remission is not attained between most Mood Episodes.
- O Chronic: Continuously met criteria for a minimum of two years since onset of illness
- O N/A: Only one episode
- O No Information

SPECIFIC PATTERNS

- O Single Episode: This specifier should be used if there has been only one episode of Major Depression since onset of illness.
- O Recurrent: This specifier should be used if there has been two or more episodes of Major Depression since onset if illness.
- O No Pattern:

O No Information

SEASONAL PATTERN

IF THERE HAVE BEEN FEWER THAN TWO MAJOR DEPRESSIVE EPISODES, CHECK HERE AND GO TO *DYSTHYMIC DISORDER*.

O Check Here

IF UNKNOWN: Do the periods of (DEPRESSED MOOD) mostly seem to happen at the same time of the year, like fall or winter?	A. There has been a regular temporal relationship between the onset of Major Depressive Episodes and a particular time of year (e.g., regular appearance of the Major Depressive Episode in the fall or winter).<br><br>NOTE: DO NOT INCLUDE CASES IN WHICH THERE IS AN OBVIOUS EFFECT OF SEASONAL-RELATED PSYCHOSOCIAL STRESSORS (E.G., REGULARLY BEING UNEMPLOYED EVERY WINTER)	O 1 O 3
IF UNKNOWN: Do the periods of (DEPRESSED MOOD) get better around the same time of year, like in the spring?	B. Full remissions also occur at a characteristic time of the year (e.g., depression disappears in the spring).	O 1 O 3
IF UNKNOWN: How many periods of (DEPRESSION) did you have during the past two years? How many of these occurred at the same time of the year?	C. In the last 2 years, two Major Depressive Episodes have occurred that demonstrate the temporal relationship defined in criteria A and B, and no non-seasonal episodes have occurred during that same period.	O 1 O 3
IF UNKNOWN: About how many years can you recall having had periods of (DEPRESSION)? How often did they occur in (ALTERNATES OF SEASONS STATED) rather than in (SEASONS STATED)?	D. Seasonal Major Depressive Episodes (as described above) substantially outnumber the non-seasonal Major Depressive Episodes that may have occurred over the individual's lifetime	O 1 O 3
	SEASONAL PATTERN CRITERIA A, B, C AND D ARE ALL CODED "3"	false

MINOR DEPRESSIVE DISORDER

MINOR DEPRESSIVE DISORDER DIFFERENTIAL CRITERIA

	At least one Minor Depressive Episode that is not due to the direct physiological effects of a general medical condition or substance use	O 1 O 3
	At least one Minor Depressive Episode is not better accounted for by Schizoaffective Disorder and is not superimposed on Schizophrenia, Schizophreniform Disorder, Delusional Disorder, or Psychotic Disorder Not Otherwise Specified	O 1 O 3
	Has never had any Major Depressive, Manic, Mixed, or unequivocal Hypomanic Episodes	O 1 O 3

NOTE: THIS EXCLUSION DOES APPLY IF ALL OF THE MANIC-LIKE, MIXED-LIKE, OR HYPOMANIC-LIKE EPISODES ARE TREATMENT INDUCED. IF SO, CODE "1" AND RECONSIDER BPI, BPII, OR BP NOS. IF FULL CRITERIA ARE NOT MET, GO TO MOOD DISORDER NOS, D27. THIS EXCLUSION DOES NOT APPLY IF ALL OF THE MANIC-LIKE, MIXED-LIKE, OR HYPOMANIC-LIKE EPISODES ARE SUBSTANCE INDUCED OR ARE DUE TO THE DIRECT PHYSIOLOGICAL EFFECTS OF A GENERAL MEDICAL CONDITION (CODE "3").

MINOR DEPRESSIVE DISORDER DIAGNOSTIC SPECIFIERS

LIFETIME SEVERITY:

(Additional questions regarding impairment may be necessary.)

- O Mild: (GAF = 61-80) Minimum symptom criteria are met for a Depressive Episode or few, if any, symptoms in excess of those required to make the diagnosis and symptoms result in only minor impairment in occupational functioning or in usual social activities or relationships with others. Either mild disability is present or substantial or unusual effort is needed to function normally.
- O Moderate: (GAF = 51-60) Intermediate between "mild" and "severe."
- O Severe: (GAF = 50) Several symptoms in excess of those required to make the diagnosis, and symptoms markedly interfere with occupational functioning or with usual social activities or relationships with others.
- O No Information

PSYCHOTIC FEATURES:

(This rating refers to Minor Depressive Disorder over the subject's lifetime. Psychotic symptom ratings in Module A refer to the actual depressive episodes as opposed to the diagnosis as a whole.)

- O Mood-Congruent Psychotic Features: Delusions or hallucinations whose content is entirely consistent with either the typical manic themes of inflated worth, power, knowledge, identity, or special relationship to a deity or famous person, or the typical depressive themes of personal inadequacy, guilt, disease, death, nihilism, or deserved punishment.
- O Mood-Incongruent Psychotic Features: Delusions or hallucinations whose content does not involve either typical manic themes of inflated worth, power, knowledge, identity, or special relationship to a deity or famous person or typical depressive themes of personal inadequacy, guilt, disease, death, nihilism, or deserved punishment. Included are such symptoms as persecutory delusions (not directly related to grandiose ideas/themes or to depressivethemes), thought insertion, thought broadcasting, and delusions of being controlled.
- O History of Psychotic Features, Unspecified
- O No History of Psychotic Features
- O No Information

CURRENT STATE:

- O In Partial Remission: Symptoms of a Minor Depressive Episode are present but full criteria are not met, or there is a period without any significant symptoms of a Minor Depressive Episode lasting less than two months following the end of the episode.
- O In Full Remission: During the past two months no significant signs or symptoms of the disturbance.
- O Currently Meets Full Criteria: Full Criteria for an illness must be met at the time of the interview
- O Currently Symptomatic: Symptoms are present but full criteria are not met. This differs from Partial Remission in that it does not immediately follow a period of illness (i.e., there was a symptom-free period of at least two months before the current symptoms began).
- O No Information

LIFETIME COURSE:

O With Full Interepisode Recovery: This specifier is used when full remission is attained between most Mood Episodes for at least two months.

O Without Full Interepisode Recovery: This specifier is used when full remission is not attained between most Mood Episodes.

O Chronic: Continuously met criteria for a minimum of two years since onset of illness

O N/A: Only one episode

O No Information

SPECIFIC PATTERNS

O Single Episode: This specifier should be used if there has been only one episode of Minor Depression since onset of illness.

O Recurrent: This specifier should be used if there has been two or more episodes of Minor Depression since onset if illness.

O No Pattern:

O No Information

SEASONAL PATTERN

IF THERE HAVE BEEN FEWER THAN TWO MINOR DEPRESSIVE EPISODES, CHECK HERE AND GO TO *DYSTHYMIC DISORDER*.

O Check Here

IF UNKNOWN: Do the periods of (DEPRESSED MOOD) mostly seem to happen at the same time of the year, like fall or winter?	A. There has been a regular temporal relationship between the onset of the Minor Depressive Episodes in Minor Depressive Disorder and a particular time of year (e.g., regular appearance of the Minor Depressive Episode in the fall or winter).<br><br>NOTE: DO NOT INCLUDE CASES IN WHICH THERE IS AN OBVIOUS EFFECT OF SEASONAL-RELATED PSYCHOSOCIAL STRESSORS (E.G., REGULARLY BEING UNEMPLOYED EVERY WINTER)	O 1 O 3
IF UNKNOWN: Do the periods of [DEPRESSED MOOD] get better around the same time of year, like in the spring?	B. Full remissions also occur at a characteristic time of the year (e.g., depression disappears in the spring).	O 1 O 3
IF UNKNOWN: How many periods of [DEPRESSION] did you have during the past two years? How many of these occurred at the same time of the year?	C. In the last 2 years, two Minor Depressive Episodes have occurred that demonstrate the temporal relationship defined in criteria A and B, and no non-seasonal Major/Minor Depressive Episodes have occurred during that same period.	O 1 O 3
IF UNKNOWN: About how many years can you recall having had periods of [DEPRESSION]? How often did they occur in [ALTERNATES OF SEASONS STATED] rather than in [SEASONS STATED]?	D. Seasonal Minor Depressive Episodes (as described above) substantially outnumber the non-seasonal Minor Depressive Episodes that may have occurred over the individual's lifetime	O 1 O 3
	SEASONAL PATTERN CRITERIA A, B, C AND D ARE ALL CODED "3"	false

DYSTHYMIC DISORDER

DYSTHYMIC DISORDER DIFFERENTIAL CRITERIA

	No Major Depressive Episode has been present during the first two years of the disturbance (1 year for children and adolescents); i.e., the disturbance is not better accounted for by chronic Major Depressive Disorder, or Major Depressive Disorder, In Partial Remission.	O 1 O 3

NOTE: THERE MAY HAVE BEEN A PREVIOUS MAJOR DEPRESSIVE EPISODE PROVIDED THERE WAS A FULL REMISSION (NO SIGNIFICANT SIGNS OR SYMPTOMS FOR TWO MONTHS) BEFORE DEVELOPMENT OF THE DYSTHYMIC DISORDER. IN ADDITION, AFTER THE INITIAL TWO YEARS (1 YEAR IN CHILDREN OR ADOLESCENTS) OF DYSTHYMIC DISORDER, THERE MAY BE SUPERIMPOSED EPISODES OF MAJOR DEPRESSIVE DISORDER, IN WHICH CASE BOTH DIAGNOSES MAY BE GIVEN WHEN THE CRITERIA ARE MET FOR A MAJOR DEPRESSIVE EPISODE.

There has never been a Manic Episode, a Mixed Episode, or a Hypomanic Episode, and criteria have never been met for Cyclothymic Disorder.	O 1	O 3
The disturbance does not occur exclusively during the course of a chronic Psychotic Disorder, such as Schizophrenia or Delusional Disorder.	O 1	O 3
The symptoms are not due to the direct physiological effects of a substance (e.g., drug of abuse, a medication) or a general medical condition (e.g., hypothyroidism).	O 1	O 3

DYSTHYMIC DISORDER DIAGNOSTIC SPECIFIERS

LIFETIME SEVERITY:

(Additional questions regarding impairment may be necessary.)

O Mild: (GAF = 61-80) Minimum symptom criteria are met or few, if any, symptoms in excess of those required to make the diagnosis and/or symptoms result in only minimal interference in normal functioning. Either mild disability is present or substantial or unusual effort is needed to function normally.

O Moderate: (GAF = 51-60) Intermediate between "mild" and "severe."

O Severe: (GAF = 50) Several symptoms in excess of those required to make the diagnosis and/or symptoms result in only limited functioning in most aspects of life almost all the time.

O No Information

PSYCHOTIC FEATURES:

(This rating refers to Dysthymic Disorder over the subject's lifetime. Psychotic symptom ratings in Module A refer to the actual mood symptoms as opposed to the diagnosis as a whole.)

O Mood-Congruent Psychotic Features: Delusions or hallucinations whose content is entirely consistent with either the typical manic themes of inflated worth, power, knowledge, identity, or special relationship to a deity or famous person, or the typical depressive themes of personal inadequacy, guilt, disease, death, nihilism, or deserved punishment.

O Mood-Incongruent Psychotic Features: Delusions or hallucinations whose content does not involve either typical manic themes of inflated worth, power, knowledge, identity, or special relationship to a deity or famous person or typical depressive themes of personal inadequacy, guilt, disease, death, nihilism, or deserved punishment. Included are such symptoms as persecutory delusions (not directly related to grandiose ideas/themes or to depressivethemes), thought insertion, thought broadcasting, and delusions of being controlled.

O History of Psychotic Features, Unspecified

O No History of Psychotic Features

O No Information

CURRENT STATE:

- O In Partial Remission: Symptoms are present but full criteria are not met, or there is a period without any significant symptoms lasting less than two months following the end of the episode.
- O In Full Remission: During the past two months no significant signs or symptoms of the disturbance.
- O Currently Meets Full Criteria: Full Criteria for an illness must be met at the time of the interview.
- O Currently Symptomatic: Symptoms are present but full criteria are not met. This differs from Partial Remission in that it does not immediately follow a period of illness (i.e., there was a symptom-free period of at least two months before the current symptoms began).
- O No Information

LIFETIME COURSE:

- O With Full Interepisode Recovery: This specifier is used when full remission is attained between most Mood Episodes for at least two months.
- O Without Full Interepisode Recovery: This specifier is used when full remission is not attained between most Mood Episodes.
- O Chronic: Continuously met criteria for a minimum of two years since onset of illness
- O N/A: Only one episode
- O No Information

SPECIFIC PATTERNS:

- O Single Episode: Only one episode of Dysthymic Disorder has ever been present.
- O Recurrent: More than one episode of Dysthymic Disorder has been present.
- O No Pattern:
- O No Information

ONSET:

- O Early Onset: Onset before age 21.
- O Late Onset: Onset at or after age 21.
- O No Information

CHRONOLOGY

How old were you when you first began to have (SYMPTOMS OF DYSTHYMIC DISORDER) regularly?	Age at onset of Dysthymic Disorder (CODE 99 IF UNKNOWN)	
IF UNCLEAR: During the past month how many periods of (SYMPTOMS OF DYSTHYMIC DISORDER) have you had?	Has met symptomatic criteria for Dysthymic Disorder during past month	O ? O 1 O 3
When did you last have any (SYMPTOMS OF DYSTHYMIC DISORDER)?	Number of months prior to interview when last had a symptom of Dysthymic Disorder	
End of Dysthymic Disorder	Check here to continue with Mood Disorder Due to	O Check Here

a GMC

DEPRESSIVE DISORDER NOT OTHERWISE SPECIFIED

DEPRESSIVE DISORDER NOT OTHERWISE SPECIFIED DIFFERENTIAL CRITERIA

Clinically significant depressive symptoms that do not meet criteria for Major Depressive Disorder, Dysthymic Disorder, or Adjustment Disorder, and are not better accounted for by Bereavement

O 1 O 3

IF UNKNOWN: Just before this began, were you physically ill?

IF YES: What did the doctor say?

IF UNKNOWN: Just before this began, were you taking any medications?

IF YES: Any change in the amount you were taking?

IF UNKNOWN: Just before this began, were you drinking or using any street drugs?

Not due to the direct physiological effects of a substance (e.g., a drug of abuse, medication) or to a general medical condition

IF THERE IS ANY INDICATION THAT THE DEPRESSIVE SYMPTOMS MAY BE SECONDARY (I.E., A DIRECT PHYSIOLOGICAL CONSEQUENCE OF A GMC OR SUBSTANCE, GO TO *GMC/SUBSTANCE* A51, AND RETURN HERE TO MAKE A RATING OF "1" OR "3."

O ? O 1 O 3

Etiological general medical conditions include: degenerative neurological illness (e.g., Parkinson's disease), cerebrovascular disease (e.g., stroke), metabolic conditions (e.g., hyper- and hypothyroidism, hyper- and hypoadrenocorticism); viral or other infections (e.g., hepatitis, mononucleosis, HIV), and certain cancers (e.g., carcinoma of the pancreas).

Etiological substances include: alcohol, amphetamines, cocaine, hallucinogens, inhalants, opiods, phencyclidine, sedatives, hypnotics, anxiolytics. Medications include antihypertensives, oral contraceptives, corticosteroids, anabolic steroids, anticancer agents, analgesics, anticholinergics, cardiac medications.

O Select to go to MOOD DISORDER SECONDARY TO A GMC

O Select to go to SUBSTANCE-INDUCED MOOD DISORDER

DEPRESSIVE DISORDER NOS DIAGNOSTIC SPECIFIERS

LIFETIME SEVERITY:

(Additional questions regarding impairment may be necessary.)

O Mild: (GAF = 61-80) Minimum symptom criteria are met or few, if any, symptoms in excess of those required to make the diagnosis and/or symptoms result in only minimal interference in normal functioning. Either mild disability is present or substantial or unusual effort is needed to function normally.

O Moderate: (GAF = 51-60) Intermediate between "mild" and "severe."

O Severe: (GAF = 50) Several symptoms in excess of those required to make the diagnosis and/or symptoms result in only limited functioning in most aspects of life almost all the time.

O No Information

PSYCHOTIC FEATURES:

(This rating refers to Minor Depressive Disorder over the subject's lifetime. Psychotic symptom ratings in Module A refer to the actual depressive episodes as opposed to the diagnosis as a whole.)

O Mood-Congruent Psychotic Features: Delusions or hallucinations whose content is entirely consistent with either the typical manic themes of inflated worth, power, knowledge, identity, or special relationship to a deity or famous person, or the typical depressive themes of personal inadequacy, guilt, disease, death, nihilism, or deserved punishment.

O Mood-Incongruent Psychotic Features: Delusions or hallucinations whose content does not involve either typical manic themes of inflated worth, power, knowledge, identity, or special relationship to a deity or famous person or typical depressive themes of personal inadequacy, guilt, disease, death, nihilism, or deserved punishment. Included are such symptoms as persecutory delusions (not directly related to grandiose ideas/themes or to depressivethemes), thought insertion, thought broadcasting, and delusions of being controlled.

O History of Psychotic Features, Unspecified

O No History of Psychotic Features

O No Information

FEATURES/SUBTYPES:

O Premenstrual Dysphoric Disorder: In most menstrual cycles in the past year, symptoms (e.g., markedly depressed mood, marked anxiety, marked affective lability, decreased interest in activities) regularly occurred during the last week of the luteal phase (and remitted within a few days of the onset of menses). These symptoms must be severe enough to markedly interfere with work, school, or usual activities and be entirely absent for at least one week postmenses (see DSM-IV p.715 for suggested research criteria).

O Recurrent Brief Depressive Disorder: Depressive episodes from two days up to two weeks, occurring at least once a month for 12 months (not associated with the menstrual cycle) (see DSM-IV p.721 for suggested research criteria).

O Postpsychotic Depressive Disorder: A Major Depressive Episode that occurs during the residual phase of Schizophrenia (see p.711 for suggested research criteria).

O Major Depressive Episode Superimposed on Psychosis: This specifier applies when a Major Depressive Episode is superimposed on Delusional Disorder, Psychotic Disorder Not Otherwise Specifed, or the active phase of Schizophrenia.

O Depressive Disorder with GMC/Substance: This specifier should be used in situations in which the clinician has concluded that a depressive disorder is present but is unable to determine whether it is primary, due to a general medical condition, or substance induced.

O Other (describe):

O No Information

CHRONOLOGY

How old were you when you first began to have (SYMPTOMS OF DEPRESSIVE DISORDER NOS) regularly?	Age at onset of Depressive Disorder NOS (CODE 99 IF UNKNOWN)	
IF UNCLEAR: During the past month how many periods of (SYMPTOMS OF DEPRESSIVE DISORDER NOS) have you had?	Has met symptomatic criteria for Depressive Disorder NOS during past month	O ? O 1 O 3
When did you last have any (SYMPTOMS OF DEPRESSIVE DISORDER NOS)?	Number of months prior to interview when last had a symptom of Depressive Disorder NOS	
End of Depressive Disorder NOS	Check here to continue with Hyperthymic Treatment	O Check Here

MOOD DISORDER NOT OTHERWISE SPECIFIED (NOS)

MOOD DISORDER NOT OTHERWISE SPECIFIED (NOS) DIFFERENTIAL CRITERIA

This category includes disorders with mood symptoms that do not meet criteria for any specific Mood Disorder and in which it is difficult to choose between Depressive Disorder Not Otherwise Specified and Bipolar Disorder Not Otherwise Specified (e.g., acute agitation).

O 1 O 3

IF UNKNOWN: Just before this began, were you physically ill?

IF YES: What did the doctor say?

IF UNKNOWN: Just before this began, were you taking any medications?

IF YES: Any change in the amount you were taking?

IF UNKNOWN: Just before this began, were you drinking or using any street drugs?

Not due to the direct physiological effects of a substance (e.g., a drug of abuse, medication) or to a general medical condition

IF THERE IS ANY INDICATION THAT THE DEPRESSIVE SYMPTOMS MAY BE SECONDARY (I.E., A DIRECT PHYSIOLOGICAL CONSEQUENCE OF A GMC OR SUBSTANCE, GO TO *GMC/SUBSTANCE* A51, AND RETURN HERE TO MAKE A RATING OF "1" OR "3."

O ? O 1 O 3

Etiological general medical conditions include: degenerative neurological illness (e.g., Parkinson's disease), cerebrovascular disease (e.g., stroke), metabolic conditions (e.g., hyper- and hypothyroidism, hyper- and hypoadrenocorticism); viral or other infections (e.g., hepatitis, mononucleosis, HIV), and certain cancers (e.g., carcinoma of the pancreas).

Etiological substances include: alcohol, amphetamines, cocaine, hallucinogens, inhalants, opiods, phencyclidine, sedatives, hypnotics, anxiolytics. Medications include antihypertensives, oral contraceptives, corticosteroids, anabolic steroids, anticancer agents, analgesics, anticholinergics, cardiac medications.

O Select to go to MOOD DISORDER SECONDARY TO A GMC

O Select to go to SUBSTANCE-INDUCED MOOD DISORDER

MOOD DISORDER NOS DIAGNOSTIC SPECIFIERS

LIFETIME SEVERITY:

(Additional questions regarding impairment may be necessary.)

O Mild: (GAF = 61-80) Minimum symptom criteria are met or few, if any, symptoms in excess of those required to make the diagnosis and/or symptoms result in only minimal interference in normal functioning. Either mild disability is present or substantial or unusual effort is needed to function normally.

O Moderate: (GAF = 51-60) Intermediate between "mild" and "severe."

O Severe: (GAF = 50) Several symptoms in excess of those required to make the diagnosis and/or symptoms result in only limited functioning in most aspects of life almost all the time.

O No Information

PSYCHOTIC FEATURES:

(This rating refers to Mood Disorder NOS over the subject's lifetime. Psychotic symptom ratings in Module A refer to the actual depression and manic episodes as opposed to the diagnosis as a whole.)

O Mood-Congruent Psychotic Features: Delusions or hallucinations whose content is entirely consistent with either the typical manic themes of inflated worth, power, knowledge, identity, or special relationship to a deity or famous person, or the typical depressive themes of personal inadequacy, guilt, disease, death, nihilism, or deserved punishment.

O Mood-Incongruent Psychotic Features: Delusions or hallucinations whose content does not involve either typical manic themes of inflated worth, power, knowledge, identity, or special relationship to a deity or famous person or typical depressive themes of personal inadequacy, guilt, disease, death, nihilism, or deserved punishment. Included are such symptoms as persecutory delusions (not directly related to grandiose ideas/themes or to depressivethemes), thought insertion, thought broadcasting, and delusions of being controlled.

O History of Psychotic Features, Unspecified

O No History of Psychotic Features

O No Information

CHRONOLOGY

How old were you when you first began to have

Age at onset of Mood Disorder NOS (CODE 99 IF

(SYMPTOMS OF MOOD DISORDER NOS) regularly?	UNKNOWN)	
IF UNCLEAR: During the past month how many periods of (SYMPTOMS OF MOOD DISORDER NOS) have you had?	Has met symptomatic criteria for Mood Disorder NOS during past month	O ? O 1 O 3
When did you last have any (SYMPTOMS OF MOOD DISORDER NOS)?	Number of months prior to interview when last had a symptom of Mood Disorder NOS	
End of Mood Disorder NOS	Check here to continue with Hyperthymic Treatment	O Check Here

MOOD DISORDER SECONDARY TO A GMC

MOOD DISORDER SECONDARY TO A GMC DIFFERENTIAL CRITERIA

There is evidence from the history, physical examination, or laboratory findings that the disturbance is the direct physiological consequence of a general medical condition. — O 1 O 3

The disturbance is not better accounted for by another mental disorder (e.g., Adjustment Disorder With Depressed Mood in response to the stress of having a general medical condition). — O 1 O 3

The disturbance does not occur exclusively during the course of a delirium. — O 1 O 3

MOOD DISORDER SECONDARY TO A GMC DIAGNOSTIC SPECIFIERS

LIFETIME SEVERITY:

(Additional questions regarding impairment may be necessary.)

O Mild: (GAF = 61-80) Minimum symptom criteria are met or few, if any, symptoms in excess of those required to make the diagnosis and/or symptoms result in only minimal interference in normal functioning. Either mild disability is present or substantial or unusual effort is needed to function normally.

O Moderate: (GAF = 51-60) Intermediate between "mild" and "severe."

O Severe: (GAF = 50) Several symptoms in excess of those required to make the diagnosis and/or symptoms result in only limited functioning in most aspects of life almost all the time.

O No Information

PSYCHOTIC FEATURES:

(This rating refers to the Mood Disorder Due to a GMC diagnosis. Psychotic symptom ratings in Module A refer to the actual depression and manic symptoms as opposed to the diagnosis as a whole.)

O Mood-Congruent Psychotic Features: Delusions or hallucinations whose content is entirely consistent with either the typical manic themes of inflated worth, power, knowledge, identity, or special relationship to a deity or famous person, or the typical depressive themes of personal inadequacy, guilt, disease, death, nihilism, or deserved punishment.

O Mood-Incongruent Psychotic Features: Delusions or hallucinations whose content does not involve either typical manic themes of inflated worth, power, knowledge, identity, or special relationship to a deity or famous person or typical depressive themes of personal inadequacy, guilt, disease, death, nihilism, or deserved punishment. Included are such symptoms as persecutory delusions (not directly related to grandiose ideas/themes or to depressivethemes), thought insertion, thought broadcasting, and delusions of being controlled.

O History of Psychotic Features, Unspecified

O No History of Psychotic Features

O No Information

CURRENT STATE:

O In Partial Remission: Symptoms are present but full criteria are not met, or there is a period without any significant symptoms lasting less than two months following the end of the episode.

O In Full Remission: During the past two months no significant signs or symptoms of the disturbance.

O Currently Meets Full Criteria: Full Criteria for an illness must be met at the time of the interview.

O Currently Symptomatic: Symptoms are present but full criteria are not met. This differs from Partial Remission in that it does not immediately follow a period of illness (i.e., there was a symptom-free period of at least two months before the current symptoms began).

O No Information

FEATURES/SUBTYPES:

O With Depressive Features: This subtype is used if the predominant mood is depressed, but the full criteria for a Major Depressive Episode are not met.

O Met Criteria for Major Depression: This subtype is used if the full criteria (except Criterion D) for a Major Depressive Episode are met.

O With Manic Features: This subtype is used if the predominant mood is elevated, euphoric, or irritable

O Met Criteria for Mania: This subtype is used if the full criteria (except Criterion E) for a Manic Episode are met.

O Met Criteria for Hypomania: This subtype is used if the full criteria (except Criterion F) for a Hypomanic Episode are met.

O With Mixed Features: This subtype is used if the symptoms of both mania and depression are present but neither predominates.

O Met Criteria for Mixed State: This subtype is used if the full criteria (except Criterion C) for a Mixed State Episode are met.

O No Information

CHRONOLOGY

How old were you when you first began to have (SYMPTOMS OF MOOD DISORDER SECONDARY TO A GMC) regularly?	Age at onset of Mood Disorder Secondary to a GMC (CODE 99 IF UNKNOWN)	
IF UNCLEAR: During the past month how many periods of (SYMPTOMS OF MOOD DISORDER SECONDARY TO A GMC) have you had?	Has met symptomatic criteria for Mood Disorder Secondary to a GMC during past month	O ? O 1 O 3
When did you last have any (SYMPTOMS OF MOOD DISORDER SECONDARY TO A GMC)?	Number of months prior to interview when last had a symptom of Mood Disorder Secondary to a GMC	
End of Mood Disorder Secondary to GMC	Continue below with Substance-Induced Mood Disorder	

SUBSTANCE-INDUCED MOOD DISORDER

SUBSTANCE-INDUCED MOOD DISORDER DIFFERENTIAL CRITERIA

There is evidence from the history, physical examination, or laboratory findings of either (1) or (2):<br><br>(1) The mood symptoms developed during, or within a month of, Substance Intoxication or Withdrawal.<br>(2) Medication use is etiologically related to the disturbance.	O 1 O 3

The disturbance is not better accounted for by a Mood Disorder that is not substance induced. Evidence that the symptoms are better accounted for be a Mood Disorder that is not substance induced might include the following: the symptoms precede the onset of the substance use (or medication use); the symptoms persist for a substantial period of time (e.g., about one month) after the cessation of acute withdrawal or severe intoxication or are substantially in excess of what would be expected given the type or amount of the substance used or the duration of use; or there is other evidence that suggests the existence of an independent non-substance-induced Mood Disorder (e.g., a history of recurrent Major Depressive Episodes)	O 1 O 3

The disturbance does not occur exclusively during the course of a delirium.	O 1 O 3

SUBSTANCE INDUCED MOOD DISORDER DIAGNOSTIC SPECIFIERS

LIFETIME SEVERITY:

(Additional questions regarding impairment may be necessary.)

O Mild: (GAF = 61-80) Minimum symptom criteria are met or few, if any, symptoms in excess of those required to make the diagnosis and/or symptoms result in only minimal interference in normal functioning. Either mild disability is present or substantial or unusual effort is needed to function normally.

O Moderate: (GAF = 51-60) Intermediate between "mild" and "severe."

O Severe: (GAF = 50) Several symptoms in excess of those required to make the diagnosis and/or symptoms result in only limited functioning in most aspects of life almost all the time.

O No Information

PSYCHOTIC FEATURES:

(This rating refers to the Mood Disorder Due to a GMC diagnosis. Psychotic symptom ratings in Module A refer to the actual depression and manic symptoms as opposed to the diagnosis as a whole.)

O Mood-Congruent Psychotic Features: Delusions or hallucinations whose content is entirely consistent with either the typical manic themes of inflated worth, power, knowledge, identity, or special relationship to a deity or famous person, or the typical depressive themes of personal inadequacy, guilt, disease, death, nihilism, or deserved punishment.

O Mood-Incongruent Psychotic Features: Delusions or hallucinations whose content does not involve either typical manic themes of inflated worth, power, knowledge, identity, or special relationship to a deity or famous person or typical depressive themes of personal inadequacy, guilt, disease, death, nihilism, or deserved punishment. Included are such symptoms as persecutory delusions (not directly related to grandiose ideas/themes or to depressivethemes), thought insertion, thought broadcasting, and delusions of

being controlled.

O History of Psychotic Features, Unspecified

O No History of Psychotic Features

O No Information

CURRENT STATE:

O In Partial Remission: Symptoms are present but full criteria are not met, or there is a period without any significant symptoms lasting less than two months following the end of the episode.

O In Full Remission: During the past two months no significant signs or symptoms of the disturbance.

O Currently Meets Full Criteria: Full Criteria for an illness must be met at the time of the interview.

O Currently Symptomatic: Symptoms are present but full criteria are not met. This differs from Partial Remission in that it does not immediately follow a period of illness (i.e., there was a symptom-free period of at least two months before the current symptoms began).

O No Information

FEATURES/SUBTYPES:

O With Depressive Features: This subtype is used if the predominant mood is depressed, but the full criteria for a Major Depressive Episode are not met.

O Met Criteria for Major Depression: This subtype is used if the full criteria (except Criterion D) for a Major Depressive Episode are met.

O With Manic Features: This subtype is used if the predominant mood is elevated, euphoric, or irritable

O Met Criteria for Mania: This subtype is used if the full criteria (except Criterion E) for a Manic Episode are met.

O Met Criteria for Hypomania: This subtype is used if the full criteria (except Criterion F) for a Hypomanic Episode are met.

O With Mixed Features: This subtype is used if the symptoms of both mania and depression are present but neither predominates.

O Met Criteria for Mixed State: This subtype is used if the full criteria (except Criterion C) for a Mixed State Episode are met.

O No Information

ONSET:

O With Onset During Intoxication: This specifier should be used if criteria for intoxication with the substance are met and the symptoms develop during the intoxication syndrome.

O With Onset During Withdrawal: This specifier should be used if criteria for withdrawal from the substance are met and the symptoms develop during, or shortly after, a withdrawal syndrome.

O No Information

CHRONOLOGY

How old were you when you first began to have (SYMPTOMS OF SUBSTANCE INDUCED MOOD DISORDER) regularly?	Age at onset of Substance Induced Mood Disorder (CODE 99 IF UNKNOWN)	
IF UNCLEAR: During the past month how many periods of (SYMPTOMS OF SUBSTANCE INDUCED MOOD DISORDER) have you had?	Has met symptomatic criteria for Substance Induced Mood Disorder during past month	O ? O 1 O 3
When did you last have any (SYMPTOMS OF SUBSTANCE INDUCED MOOD DISORDER)?	Number of months prior to interview when last had a symptom of Substance Induced Mood Disorder	

End of Substance-Induced Mood Disorder

Continue below with Hyperthymic Temperament

HYPERTHYMIC TEMPERAMENT

HYPERTHYMIC TEMPERAMENT DIFFERENTIAL CRITERIA

Pervasive pattern of energetic, cheerful, and/or irritable mood present in a variety of contexts, as indicated by at least three (or more) symptoms

O 1 O 3

The symptoms are not better accounted for by Schizoaffective Disorder and are not superimposed on Schizophrenia, Schizophreniform Disorder, Delusional Disorder, or Psychotic Disorder Not Otherwise Specified.

O 1 O 3

The symptoms are not due to the direct physiological effects of a substance (e.g., drug of abuse, a medication) or a general medical condition (e.g., hypothyroidism).

O 1 O 3

HYPERTHYMIC TEMPERAMENT DIAGNOSTIC SPECIFIERS

LIFETIME SEVERITY:

(Additional questions regarding impairment may be necessary.)

O Mild: (GAF = 61-80) Minimum symptom criteria are met or few, if any, symptoms in excess of those required to make the diagnosis and/or symptoms result in only minimal interference in normal functioning. Either mild disability is present or substantial or unusual effort is needed to function normally.

O Moderate: (GAF = 51-60) Intermediate between "mild" and "severe."

O Severe: (GAF = 50) Several symptoms in excess of those required to make the diagnosis and/or symptoms result in only limited functioning in most aspects of life almost all the time.

O No Information

PSYCHOTIC FEATURES:

(This rating refers to Hyperthymic Temperament over the subject's lifetime. Psychotic symptom ratings in Module A refer to the actual mood symptoms as opposed to the diagnosis as a whole.)

O Mood-Congruent Psychotic Features: Delusions or hallucinations whose content is entirely consistent with either the typical manic themes of inflated worth, power, knowledge, identity, or special relationship to a deity or famous person, or the typical depressive themes of personal inadequacy, guilt, disease, death, nihilism, or deserved punishment.

O Mood-Incongruent Psychotic Features: Delusions or hallucinations whose content does not involve either typical manic themes of inflated worth, power, knowledge, identity, or special relationship to a deity or famous person or typical depressive themes of personal inadequacy, guilt, disease, death, nihilism, or deserved punishment. Included are such symptoms as persecutory delusions (not directly related to grandiose ideas/themes or to depressivethemes), thought insertion, thought broadcasting, and delusions of being controlled.

O History of Psychotic Features, Unspecified

O No History of Psychotic Features

O No Information

CURRENT STATE:

O In Partial Remission: Symptoms are present but full criteria are not met, or there is a period without any significant symptoms lasting less than two months following the end of the episode.

O In Full Remission: During the past two months no significant signs or symptoms of the disturbance.

O Currently Meets Full Criteria: Full Criteria for an illness must be met at the time of the interview.

O Currently Symptomatic: Symptoms are present but full criteria are not met. This differs from Partial Remission in that it does not immediately follow a period of illness (i.e., there was a symptom-free period of at least two months before the current symptoms began).

O No Information

LIFETIME COURSE:

O With Full Interepisode Recovery: This specifier is used when full remission is attained between most Mood Episodes for at least two months.

O Without Full Interepisode Recovery: This specifier is used when full remission is not attained between most Mood Episodes.

O Chronic: Continuously met criteria for a minimum of two years since onset of illness

O N/A: Only one episode

O No Information

SPECIFIC PATTERNS:

O Single Episode: Only one episode of Hyperthymic Temperament has ever been present.

O Recurrent: More than one episode of Hyperthymic Temperament has been present.

O No Pattern:

O No Information

TREATMENT INDUCED:

(Use the Medication Assessment Form (from Overview) and information obtained in Module A to chose one of the following specifiers.)

O Only Treatment Induced Episodes

O History of Treatment Induced Episodes

O No History of Treatment Induced Episodes

O No Information

CHRONOLOGY

How old were you when you first began to have (SYMPTOMS OF HYPERTHYMIC TEMPERAMENT) regularly?	Age at onset of Hyperthymic Temperament (CODE 99 IF UNKNOWN)	
IF UNCLEAR: During the past month how many periods of (SYMPTOMS OF HYPERTHYMIC TEMPERAMENT) have you had?	Has met symptomatic criteria for Hyperthymic Temperament during past month	O ? O 1 O 3
When did you last have any (SYMPTOMS OF HYPERTHYMIC TEMPERAMENT)?	Number of months prior to interview when last had a symptom of Hyperthymic Temperament	
End of Module	Continue with Substance Use Disorders, Module E	

Module E: Subtance Use Disorders

ALCOHOL USE SCREENING (LIFETIME)

What are your drinking habits like? (How much do you drink? Has there ever been a time in your life when you had five or more drinks on one occasion?)

CURRENT ALCOHOL USE:

When in your life were you drinking the most? (How

long did that period last?)

RECORD DATE OF HEAVIEST USE AND DESCRIBE PATTERN:

During that time, how often were you drinking?

What were you drinking? How much?

During that time, did your drinking cause problems for you?

Did anyone object to your drinking?

Alcohol Use Screening

O Alcohol dependence seems likely. (Skip to Alcohol Dependence)

O Incidents of excessive drinking or evidence of alcohol-related problems. (Continue with Alcohol Abuse)

O Never had incidents of excessive drinking and there is no evidence of alcohol-related problems. (Skip to Nicotine Use)

ALCOHOL ABUSE

ALCOHOL ABUSE CRITERIA

Let me ask you a few more questions about your drinking habits.

A. A maladaptive pattern of substance use leading to clinically significant impairment or distress, as manifested by one (or more) of the following occurring within a twelve month period:

Have you ever missed work or school because you were intoxicated, high, or very hung over? (How often? What about doing a bad job at work or failing courses at school because of your drinking?)

IF NO: What about not keeping your house clean or not taking proper care of your children because of your drinking? How often?

IF YES TO EITHER OF ABOVE: How often? Over what period of time?

1) Recurrent alcohol use resulting in a failure to fulfill major role obligations at work, school, or home (e.g., repeated absences or poor work performance related to alcohol use; alcohol-related absences, suspensions, or expulsions from school; neglect of children or household)

O ? O 1 O 2 O 3

Did you ever drink in a situation in which it might have been dangerous to drink at all? (Did you ever drive while you were really too drunk to drive?)

IF YES AND UNKNOWN: How many times? When?

2) Recurrent alcohol use in situations in which it is physically hazardous (e.g., driving an automobile or operating a machine when impaired by alcohol use)

O ? O 1 O 2 O 3

Has your drinking gotten you into trouble with the law?<br><br>IF YES AND UNKNOWN: How often? Over what period of time?	3) Recurrent alcohol-related legal problems (e.g., arrests for alcohol-related disorderly conduct)	O O O O<br>? 1 2 3

IF UNKNOWN: Has your drinking caused problems with other people, such as with family members, friends, or people at work? (Have you ever gotten into physical fights when you were drinking? What about having bad arguments?)<br><br>IF YES: Did you keep drinking anyway? Over what period of time?	4) Continued alcohol use despite having persistent or recurrent social or interpersonal problems caused or exacerbated by the effects of alcohol (e.g., arguments with spouse about consequences of intoxication, physical fights)	O O O O<br>? 1 2 3

	AT LEAST ONE CRITERION A ITEM CODED "3"	false
CRITERION A - NOT SATISFIED:<br>IF NO POSSIBILITY OF PHYSIOLOGICAL DEPENDENCE OR COMPULSIVE USE, GO TO *NICOTINE USE*; OTHERWISE, CONTINUE ASKING ABOUT *DEPENDENCE*.	CRITERION A - SATISFIED:<br>IF ALCOHOL DEPENDENCE QUESTIONS HAVE ALREADY BEEN ASKED (I.E., DEPENDENCE SEEMED LIKELY AFTER ALCOHOL SCREENING) BUT FULL CRITERIA WERE NOT MET, GO TO *ALCOHOL ABUSE DIAGNOSTIC SPECIFIERS* BELOW.<br><br>IF ALCOHOL DEPENDENCE QUESTIONS HAVE NOT YET BEEN EVALUATED, FIRST COMPLETE *ALCOHOL ABUSE DIAGNOSTIC SPECIFIERS* BELOW, THEN CONTINUE WITH *ALCOHOL DEPENDENCE*.	

ALCOHOL ABUSE DIAGNOSTIC SPECIFIERS

LIFETIME SEVERITY:

(Additional questions regarding impairment may be necessary.)

O Mild: (GAF = 61-80) Minimum symptom criteria are met or few, if any, symptoms in excess of those required to make the diagnosis and/or symptoms result in only minimal interference in normal functioning. Either mild disability is present or substantial or unusual effort is needed to function normally.

O Moderate: (GAF = 51-60) Intermediate between "mild" and "severe."

O Severe: (GAF = 50) Several symptoms in excess of those required to make the diagnosis and/or symptoms result in only limited functioning in most aspects of life almost all the time.

O No Information

CURRENT STATE:

O In Remission: This specifier is used if, for at least one month, but less than 12 months, one or more criteria for Dependence or Abuse have been met (but the full criteria for Dependence have not been met).

O Currently Meets Full Criteria:

O Currently Symptomatic

O No Information

CHRONOLOGY

How old were you when you first began to have (SYMPTOMS OF ALCOHOL ABUSE) regularly?	Age at onset of Alcohol Abuse (CODE 99 IF UNKNOWN)	
IF UNCLEAR: During the past month, have you had anything at all to drink?<br><br>IF YES: Tell me more about it. Has your drinking caused you any problems?)	Criteria for Alcohol Abuse met at any time in past month	O ? O 1 O 3

	IF ALCOHOL DEPENDENCE QUESTIONS HAVE ALREADY BEEN ASKED, CHECK HERE TO GO TO *NICOTINE USE*.<br><br>IF ALCOHOL DEPENDENCE QUESTIONS HAVE NOT YET BEEN EVALUATED, CONTINUE WITH *ALCOHOL DEPENDENCE*.	O Check Here

ALCOHOL DEPENDENCE

ALCOHOL DEPENDENCE CRITERIA

NOTE: CRITERIA FOR ALCOHOL DEPENDENCE ARE NOT IN DSM-IV ORDER

I'd now like to ask you some more questions about your drinking habits.	A maladaptive pattern of alcohol use, leading to clinically significant impairment or distress, as manifested by three (or more) of the following occurring at any time in the same twelve month period:	
Have you often found that when you started drinking you ended up drinking much more than you were planning to?<br><br>IF NO: What about drinking for a much longer period of time than you were planning to?	1) alcohol is often taken in larger amounts OR over a longer period than intended	O ? O 1 O 2 O 3

Have you tried to cut down or stop drinking alcohol?<br><br>IF YES: Did you ever stop drinking altogether? How many times did you try to cut down or stop altogether?<br><br>IF NO: Did you want to stop or cut down? Is this	2) there is a persistent desire OR unsuccessful efforts to cut down or control alcohol use	O ? O 1 O 2 O 3

something you kept worrying about?

Have you spent a lot of time drinking, being high, or hung over?	3) a great deal of time is spent in activities necessary to obtain alcohol, use alcohol, or recover from its effects	O O O O<br>? 1 2 3
Have you had times when you would drink instead of working or spending time with your family or friends or engaging in other important activities, such as sports, gardening, or playing music?	4) important social, occupational, or recreational activities given up or reduced because of alcohol use	O O O O<br>? 1 2 3
IF UNKNOWN: Has your drinking ever caused any psychological problems like making you depressed or anxious, making it difficult to sleep, or causing "blackouts?"<br><br>IF UNKNOWN: Has your drinking ever caused significant physical problems or made a physical problem worse?<br><br>IF YES TO EITHER OF ABOVE: Did you keep on drinking anyway?	5) alcohol use is continued despite knowledge of having a persistent or recurrent physical or psychological problem that is likely to have been caused or exacerbated by alcohol (e.g., continued drinking despite recognition that an ulcer was made worse by alcohol consumption)	O O O O<br>? 1 2 3
Have you found that you needed to drink a lot more than you did when you first started drinking in order to get the feeling you wanted?<br><br>IF YES: How much more?<br><br>IF NO: What about finding that when you drank the same amount, it had much less effect than before?	6) tolerance, as defined by either of the following:<br>a) a need for markedly increased amounts of alcohol to achieve intoxication or desired effect<br>b) markedly diminished effect with continued use of the same amount of alcohol	O O O O<br>? 1 2 3
Have you ever had any withdrawal symptoms when you cut down or stopped drinking like...<br>...sweating or racing heart?<br>...shaking hands?	7) withdrawal, as manifested by either (a) or (b):<br><br>a) at least two of the following:<br>- autonomic hyperactivity	O O O O<br>? 1 2 3

...trouble sleeping?
...feeling nauseated or vomiting?
...feeling agitated?
...or feeling anxious?

How about having a seizure or seeing, feeling, or hearing things that weren't really there?

IF NO: Have you ever started the day with a drink, or did you often drink or take some other drug or medication to keep yourself from getting the shakes or becoming sick?

(e.g., sweating or pulse rate greater than 100)
- increased hand tremor
- insomnia
- nausea or vomiting
- psychomotor agitation
- anxiety
- grand mal seizures
- transient visual, tactile, or auditory hallucinations or illusions.

b) alcohol (or a substance from the sedative/hypnotic/anxiolytic class) taken to relieve or avoid withdrawal symptoms

IF UNKNOWN: When did (SYMPTOMS CODED "3" ABOVE) occur? (Did they all happen around the same time?)

AT LEAST THREE ALCOHOL DEPENDENCE ITEMS CODED "3" AND ITEMS OCCURRED WITHIN THE SAME TWELVE MONTH PERIOD

false

ALCOHOL DEPENDENCE DIAGNOSTIC SPECIFIERS

LIFETIME SEVERITY:

(Additional questions regarding impairment may be necessary.)

O Mild: (GAF = 61-80) Minimum symptom criteria are met or few, if any, symptoms in excess of those required to make the diagnosis and/or symptoms result in only minimal interference in normal functioning. Either mild disability is present or substantial or unusual effort is needed to function normally.

O Moderate: (GAF = 51-60) Intermediate between "mild" and "severe."

O Severe: (GAF = 50) Several symptoms in excess of those required to make the diagnosis and/or symptoms result in only limited functioning in most aspects of life almost all the time.

O No Information

CURRENT STATE:

(The following remission specifiers can be applied only after no criteria for dependence or abuse have been met for at least one month in the past. Note: These specifiers do not apply if the individual is On Agonist Therapy or In a Controlled Environment (next page).

O Early Partial Remission: This specifier is used if, for at least one month, but less than 12 months, one or more criteria for Dependence or Abuse have been met (but the full criteria for Dependence have not been met).

O Early Full Remission: This specifier is used if, for at least one month, but less than twelve months, no criteria for Dependence or Abuse have been met.

O Sustained Partial Remission: This specifier is used if full criteria for Dependence have not been met for a period of twelve months or longer; however, one or more criteria for Dependence or Abuse have been met.

O Sustained Full Remission: This specifier is used if none of the criteria for Dependence or Abuse have been met at any time during a period of twelve months or longer

O Currently Meets Full Criteria:

O Currently Symptomatic

O No Information

LIFETIME COURSE:

O With Full Interepisode Recovery: Full remission is attained for at least two months.

O Without Full Interepisode Recovery: Full remission is not attained.

O Chronic: Continuously met criteria for a minimum of two years since onset of illness.

O N/A: Only one episode.

O No Information

FEATURES/SUBTYPES:

O With Physiological Dependence: This specifier should be used when Substance Dependence isaccompanied by current or prior evidence of tolerance or withdrawal.

O Without Physiological Dependence: This specifier should be used when there is no current or prior evidence of tolerance or withdrawal. In these individuals, Substance Dependence is characterized by a pattern of compulsive use.

O No Features

O No Information

CURRENT TREATMENT:

O On Agonist Therapy: The individual is on a prescribed agonist medication (e.g., valium) and no criteria for Dependence or Abuse have been met for that class of medication for at least the past month (except tolerance to, or withdrawal from, the agonist). This category also applies to those being treated for Dependence using a partial agonist or a mixed agonist/antagonist.

O In a Controlled Environment: The individual is in an environment where access to alcohol and controlled substances is restricted and no criteria for Dependence or Abuse have been met for at least the past month. Examples are closely-supervised and substance-free jails, therapeutic communities, and locked hospital units.

O On Agonist Therapy In a Controlled Environment: See items #1 and #2 above.

O No Current Treatment

O No Information

CHRONOLOGY

How old were you when you first had (LIST OF ALCOHOL DEPENDENCE SYMPTOMS CODED "3")?	Age at onset of Alcohol Dependence (CODE 99 IF UNKNOWN)	
IF UNCLEAR: During the past month, have you had anything at all to drink?<br><br>IF YES: Tell me more about it. Has your drinking caused you any problems?	Full criteria for Alcohol Dependence met at any time in past month (or never had a month without symptoms of Dependence or Abuse since onset of Dependence)	O ? O 1 O 3
	NUMBER OF MONTHS PRIOR TO INTERVIEW LAST HAD PROBLEMS WITH ALCOHOL:	

NICOTINE USE

1.) Have you ever used cigarettes or any other forms of tobacco on a daily basis? O ? O 1 O 3

2.) Are you currently smoking or using tobacco? O 1 O 3

IF NO: When did you quit smoking or using tobacco? AGE

3.) How old were you the first time you used any form of tobacco? AGE

4.) How old were you when you first started using any form of tobacco on a daily basis? AGE

5.) What form of tobacco have you used on a daily basis?

O Cigarettes

O Cigars

O Pipes

O Chewing tobacco/snuff

O Other

5a. Cigarettes
How many packs of cigarettes per day do/did you smoke?
[ESTIMATE THE NUMBER OF PACK YEARS.]

\# packs/day X # years = 0 Pack years

5b. Cigars
What was/is the average number of whole cigars used per day?

\# cigars/day # years

5c. Pipes
What was/is the average number of full pipe bowls used per day?

\# bowls/day # years

5d. Chewing Tobacco
What was/is the average number of full pinches of chewing tobacco or full pinches of snuff per day?

\# pinches/day # years

NON-ALCOHOL SUBSTANCE USE DISORDERS

NON-ALCOHOL SUBSTANCE USE SCREENING (LIFETIME DEPENDENCE AND ABUSE)
DETERMINE LEVEL OF DRUG USE USING GUIDELINES BELOW. CODE EACH DRUG GROUP.
Now I am going to ask you about your use of drugs or medicines.

SHOW DRUG LIST TO SUBJECT.

Have you ever taken any of these to get high, sleep better, lose weight, or change your mood?

GUIDELINES FOR RATING LEVEL OF DRUG USE

ASK FOR EACH DRUG GROUP EVER USED:

IF STREET DRUG: When were you using (DRUG) the most? Has there ever been a time when you used it at least ten times in a one-month period of time?

IF PRESCRIBED: Did you ever get hooked (BECOME DEPENDENT) on (PRESCRIBED DRUG) or take much more of it than was prescribed?

Either (1) or (2)

(1) has ever taken street drug more than ten times in a one-month period

(2) reports becoming dependent on a prescribed drug OR using much more of it than was prescribed

IF DRUG GROUP NEVER USED OR USED ONLY ONCE, OR IF PRESCRIBED DRUG USED AS DIRECTED, CIRCLE "1" FOR DRUG GROUP.

IF DRUG GROUP USED AT LEAST TWICE, BUT LESS THAN LEVEL INDICATED ON (1), CODE "2" FOR DRUG GROUP.

IF DRUG GROUP USED AT LEVEL INDICATED IN ITEM (1) OR IF POSSIBLY DEPENDENT ON PRESCRIBED DRUG (ITEM (2) IS TRUE), CODE "3" FOR DRUG GROUP.

SELECT THE NAME OF EACH DRUG EVER USED (OR WRITE IN NAME IF "OTHER")	RECORD PERIOD OF HEAVIEST USE (AGE OR DATE, AND DURATION) AND DESCRIBE PATTERN OF USE	INDICATE LEVEL OF USE (USE GUIDELINES ABOVE)
**Sedatives-hypnotics-anxiolytics:**<br>O Quaalude O Seconal<br>O Valium O Xanax<br>O Librium O barbiturates<br>O Miltown O Ativan<br>O Dalmane O Halcion<br>O Restoril O Unknown<br>O Other		O ? O 1 O 2 O 3
**Cannabis:**<br>O Marijuana O Hashish O THC<br>O Unknown O Other		O ? O 1 O 2 O 3
**Stimulants:**<br>O Amphetamine O Speed<br>O Crystal meth O Dexadrine<br>O Ritalin O Ice<br>O Unknown O Other		O ? O 1 O 2 O 3
**Opioids:**<br>O Heroin O Morphine<br>O Opium O Methadone<br>O Darvon O Codeine<br>O Percodan O Demerol		O ? O 1 O 2 O 3

O Dilaudid O Unknown

O Other

Cocaine:

O Intranasal O IV O Freebase

O Crack O Speedball O Unknown

O Other

O ? O 1 O 2 O 3

Hallucinogens/PCP:

O LSD

O Mescaline

O Peyote

O Psilocybin

O STP

O Mushrooms

O PCP ("angel dust")

O Ecstasy

O MDMA

O Unknown

O Other

O ? O 1 O 2 O 3

Other:

O Steroids

O Glue

O paint

O Inhalants

O Nitrous oxide ("laughing gas")

O Amyl or butyl nitrate ("poppers")

O GHB (liquid Ecstasy)

O nonprescription sleep or diet pills

O Unknown

O Other

O ? O 1 O 2 O 3

ANY DRUG GROUPS CODED "2" OR "3" false

IF NO DRUG CLASSES WERE CODED "3" ON PREVIOUS PAGE (I.E., "2"S ONLY), GO TO *SUBSTANCE ABUSE*.

ASK EACH OF THE FOLLOWING SUBSTANCE DEPENDENCE QUESTIONS FOR EACH DRUG CODED "3":

IF AT LEAST THREE DRUG GROUPS USED AND PERIOD OF INDISCRIMINANT USE SEEMS LIKELY, ASK THE FOLLOWING:

You've told me that you've used (DRUG). Was there a period when you were using a lot of different drugs at the same time and it did not matter what you were taking as long as you could get high?

Behavior during the same 12-month period in which the person was repeatedly using at least three groups of substance (not including caffeine and nicotine), but no single substance predominated. Further, during this period, the Dependence criteria were (likely) met for substances as a group but not for any specific substance.

O 1 O 3

NOTE: IN CASES THAT INCLUDE PERIODS OF INDISCRIMINATE USE AND OTHER PERIODS OF USE OF SPECIFIC DRUGS, POLY DRUG SHOULD BE CODED IN ADDITION TO SPECIFIC DRUG COLUMNS.

SUBSTANCE DEPENDENCE

NOTE: CRITERIA FOR DEPENDENCE ARE IN A DIFFERENT ORDER THAN IN DSM-IV.

Now I'm going to ask you some specific questions about your use of (DRUGS CODED "3").

SUBSTANCE DEPENDENCE CRITERIA

A maladaptive pattern of substance use, leading to clinically significant impairment or distress, as manifested by three (or more) of the following, occurring at any time in the same 12-month period:

Have you often found that when you started using (DRUG) you ended up using much more of it than you were planning to?

IF NO: What about using it over a much longer period of time than you were planning to?

	SED/ HYPN/ ANX	CANN ABIS	STIMU LANTS	OPIOID	COCAINE	HALL/ PCP	POLY	OTHER
(3) The substance is often taken in larger amounts OR over a longer period than was intended.	O 3	O 3	O 3	O 3	O 3	O 3	O 3	O 3
	O 2	O 2	O 2	O 2	O 2	O 2	O 2	O 2
	O 1	O 1	O 1	O 1	O 1	O 1	O 1	O 1
	O ?	O ?	O ?	O ?	O ?	O ?	O ?	O ?

Have you tried to cut down or stop using (DRUG)?

IF YES: Have you ever actually stopped using (DRUG) altogether? How many times did you try to cut down or stop altogether?

IF UNKNOWN: Did you want to stop or cut down? Is this something you kept worrying about?

	SED/ HYPN/ ANX	CANN ABIS	STIMU LANTS	OPIOID	COCAINE	HALL/ PCP	POLY	OTHER
(4) There is a persistent desire OR unsuccessful efforts to cut down or control substance use.	O 3	O 3	O 3	O 3	O 3	O 3	O 3	O 3
	O 2	O 2	O 2	O 2	O 2	O 2	O 2	O 2
	O 1	O 1	O 1	O 1	O 1	O 1	O 1	O 1

O ?	O ?	O ?	O ?	O ?	O ?	O ?	O ?

Have you spent a lot of time using (DRUG) or doing whatever you had to do to get it? Did it take you a long time to get back to normal? (How much time? As long as several hours?)

	SED/ HYPN/ ANX	CANN ABIS	STIMU LANTS	OPIOID	COCAINE	HALL/ PCP	POLY	OTHER
(5) A great deal of time is spent in activities necessary to obtain the substance, use the substance, or recover from its effects.	O 3	O 3	O 3	O 3	O 3	O 3	O 3	O 3
	O 2	O 2	O 2	O 2	O 2	O 2	O 2	O 2
	O 1	O 1	O 1	O 1	O 1	O 1	O 1	O 1
	O ?	O ?	O ?	O ?	O ?	O ?	O ?	O ?

Have you had times when you used (DRUG) instead of working or spending time on hobbies or with your family or friends?

	SED/ HYPN/ ANX	CANN ABIS	STIMU LANTS	OPIOID	COCAINE	HALL/ PCP	POLY	OTHER
(6) Important social, occupational, or recreational activities given up or reduced because of substance use.	O 3	O 3	O 3	O 3	O 3	O 3	O 3	O 3
	O 2	O 2	O 2	O 2	O 2	O 2	O 2	O 2
	O 1	O 1	O 1	O 1	O 1	O 1	O 1	O 1
	O ?	O ?	O ?	O ?	O ?	O ?	O ?	O ?

IF UNKNOWN: Has (DRUG) caused psychological problems, like making you depressed?

IF UNKNOWN: Has (DRUG) ever caused physical problems or made a physical problem worse?

IF YES TO EITHER OF ABOVE: Did you keep on using (DRUG) anyway?

	SED/ HYPN/ ANX	CANN ABIS	STIMU LANTS	OPIOID	COCAINE	HALL/ PCP	POLY	OTHER
(7) The substance use is continued despite knowledge of having had a persistent or recurrent physical or psychological problem that is likely to have been caused or exacerbated by the substance (e.g., recurrent cocaine use despite recognition of cocaine-related depression).	O 3	O 3	O 3	O 3	O 3	O 3	O 3	O 3
	O 2	O 2	O 2	O 2	O 2	O 2	O 2	O 2
	O 1	O 1	O 1	O 1	O 1	O 1	O 1	O 1
	O ?	O ?	O ?	O ?	O ?	O ?	O ?	O ?

Have you found that you needed to use a lot more (DRUG) in order to get high than you did when you first started using it?

IF YES: How much more?

IF NO: What about finding that when you used the same amount, it had much less effect than before?

	SED/ HYPN/ ANX	CANN ABIS	STIMU LANTS	OPIOID	COCAINE	HALL/ PCP	POLY	OTHER
(1) Tolerance, as defined by either of the following:	O 3	O 3	O 3	O 3	O 3	O 3	O 3	O 3

(a) a need for markedly increased amounts of the substance to achieve intoxication or desired effect.<br>(b) markedly diminished effect with continued use of the same amount of this substance.	O 2<br>O 1<br>O ?	O 2<br>O 1<br>O ?	O 2<br>O 1<br>O ?	O 2<br>O 1<br>O ?	O 2<br>O 1<br>O ?	O 2<br>O 1<br>O ?	O 2<br>O 1<br>O ?	O 2<br>O 1<br>O ?

THE FOLLOWING ITEM MAY NOT APPLY TO CANNABIS AND HALLUCINOGENS/PCP.

Have you ever had withdrawal symptoms; that is, felt sick when you cut down or stopped using (DRUG)?

IF YES: What symptoms did you have? (REFER TO LIST OF WITHDRAWAL SYMPTOMS ON E15-E16)

IF NO: After not using (DRUG) for a few hours or more, have you often used it to keep yourself from getting sick with (WITHDRAWAL SYMPTOMS)?

IF NO: What about using (DRUG IN SAME GROUP) when you were feeling sick with (WITHDRAWAL SYMPTOMS) so that you would feel better?

	SED/ HYPN/ ANX	CANN ABIS	STIMU LANTS	OPIOID	COCAINE	HALL/ PCP	POLY	OTHER
(2) Withdrawal, as manifested by either of the following:<br>(a) the characteristic withdrawal syndrome for the substance.<br>(b) the same (or a closely related) substance is taken to relieve or avoid withdrawal symptoms.	O 3<br>O 2<br>O 1<br>O ?	O 3<br>O 2<br>O 1<br>O ?	O 3<br>O 2<br>O 1<br>O ?	O 3<br>O 2<br>O 1<br>O ?	O 3<br>O 2<br>O 1<br>O ?	O 3<br>O 2<br>O 1<br>O ?	O 3<br>O 2<br>O 1<br>O ?	O 3<br>O 2<br>O 1<br>O ?

LIST OF WITHDRAWAL SYMPTOMS (FROM DSM-IV CRITERIA)

Listed below are the characteristic withdrawal symptoms for those classes of psychoactive substances for which a withdrawal syndrome has been identified. (NOTE: A specific withdrawal syndrome has not been identified for CANNABIS AND HALLUCINOGENS/PCP). Withdrawal symptoms may occur following the cessation of prolonged moderate or heavy use of a psychoactive substance or a reduction in the amount used. For polysubstance use, ask about each applicable category.

SEDATIVES, HYPNOTICS, AND ANXIOLYTICS:

Two (or more) of the following, developing within several hours to a few days after cessation (or reduction) of sedative, hypnotic, or anxiolytic use, which has been heavy and prolonged:

O autonomic hyperactivity (e.g., sweating or pulse rate greater than 100)

O increased hand tremor

O insomnia

O nausea or vomiting

O transient visual, tactile, or auditory hallucinations or illusions

O psychomotor agitation

O anxiety

O grand mal seizures

STIMULANTS/COCAINE:

Dysphoric mood AND two (or more) of the following physiological changes, developing within a few hours to several days after cessation (or reduction of substance use which has been heavy and prolonged):

O fatigue

O vivid, unpleasant dreams

O insomnia or hypersomnia

O increased appetite

O psychomotor retardation or agitation

OPIOIDS:

Three (or more) of the following, developing within minutes to several days after cessation (or reduction) of opioid use which has been heavy and prolonged (several weeks or longer) or after administration of an opioid antagonist (after a period of opioid use):

O dysphoric mood

O nausea or vomiting

O muscle aches

O lacrimation or rhinorrhea

O pupillary dilation, piloerection, or sweating

O diarrhea

O yawning

O fever

O insomnia

FOR EACH DRUG CLASS CODED "3" BELOW, GO TO *SUBSTANCE DEPENDENCE DIAGNOSTIC SPECIFIERS*

FOR EACH DRUG CLASS CODED "1" BELOW, GO TO *SUBSTANCE ABUSE* AND ASK THE FOUR ABUSE ITEMS.

IF UNKNOWN: When did (SYMPTOMS CODED "3" ABOVE) occur? (Did they all happen around the same time?)

	SED/ HYPN/ ANX	CANN ABIS	STIMU LANTS	OPIOID	COCAINE	HALL/ PCP	POLY	OTHER
Total Number of Symptoms	0	0	0	0	0	0	0	0
SUBSTANCE DEPENDENCE: AT LEAST 3 ITEMS ARE CODED "3" AND ITEMS OCCURRED WITHIN THE SAME TWELVE-MONTH PERIOD	O 3<br>O 1	O 3<br>O 1	O 3<br>O 1	O 3<br>O 1	O 3<br>O 1	O 3<br>O 1	O 3<br>O 1	O 3<br>O 1
Criteria Met:	false	false	false	false	false	false	false	false

SUBSTANCE DEPENDENCE DIAGNOSTIC SPECIFIERS

LIFETIME SEVERITY:
(Additional questions regarding impairment may be necessary.)

O Mild: (GAF = 61-80) Minimum symptom criteria are met or few, if any, symptoms in excess of those required to make the diagnosis and/or symptoms result in only minimal interference in normal functioning. Either mild disability is present or substantial or unusual effort is needed to function normally.

O Moderate: (GAF = 51-60) Intermediate between "mild" and "severe."

O Severe: (GAF = 50) Several symptoms in excess of those required to make the diagnosis and/or symptoms result in only limited functioning in most aspects of life almost all the time.

O No Information

Sedatives	
Cannabis	
Stimulants	
Opioids	
Cocaine	
Hallucinogens	
Poly Drug	
Other	

CURRENT STATE:
(The following remission specifiers can be applied only after no criteria for dependence or abuse have been met for at least one month in the past. Note: These specifiers do not apply if the individual is On Agonist Therapy or In a Controlled Environment (next page).

O Early Partial Remission: This specifier is used if, for at least one month, but less than 12 months, one or more criteria for Dependence or Abuse have been met (but the full criteria for Dependence have not been met).

O Early Full Remission: This specifier is used if, for at least one month, but less than twelve months, no criteria for Dependence or Abuse have been met.

O Sustained Partial Remission: This specifier is used if full criteria for Dependence have not been met for a period of twelve months or longer; however, one or more criteria for Dependence or Abuse have been met.

O Sustained Full Remission: This specifier is used if none of the criteria for Dependence or Abuse have been met at any time during a period of twelve months or longer

O Currently Meets Full Criteria:

O Currently Symptomatic

O No Information

Sedatives	
Cannabis	
Stimulants	
Opioids	
Cocaine	
Hallucinogens	
Poly Drug	
Other	

LIFETIME COURSE

O With Full Interepisode Recovery: Full remission is attained for at least two months.

O Without Full Interepisode Recovery: Full remission is not attained.

O Chronic: Continuously met criteria for a minimum of two years since onset of illness.

O N/A: Only one episode.

O No Information

Sedatives	
Cannabis	
Stimulants	
Opioids	
Cocaine	
Hallucinogens	
Poly Drug	
Other	

FEATURES/SUBTYPES:

O With Physiological Dependence: This specifier should be used when Substance Dependence isaccompanied by current or prior evidence of tolerance or withdrawal.

O Without Physiological Dependence: This specifier should be used when there is no current or prior evidence of tolerance or withdrawal. In these individuals, Substance Dependence is characterized by a pattern of compulsive use.

O No Features

O No Information

Sedatives	
Cannabis	
Stimulants	
Opioids	
Cocaine	
Hallucinogens	
Poly Drug	
Other	

CURRENT TREATMENT:

O On Agonist Therapy: The individual is on a prescribed agonist medication (e.g., valium) and no criteria for Dependence or Abuse have been met for that class of medication for at least the past month (except tolerance to, or withdrawal from, the agonist). This category also applies to those being treated for Dependence using a partial agonist or a mixed agonist/antagonist.

O In a Controlled Environment: The individual is in an environment where access to alcohol and controlled substances is restricted and no criteria for Dependence or Abuse have been met for at least the past month. Examples are closely-supervised and substance-free jails, therapeutic communities, and locked hospital units.

O On Agonist Therapy In a Controlled Environment: See items #1 and #2 above.

O No Current Treatment

O No Information

Sedatives	
Cannabis	
Stimulants	
Opioids	
Cocaine	
Hallucinogens	
Poly Drug	
Other	

CHRONOLOGY

How old were you when you first had (LIST OF SUBSTANCE DEPENDENCE SYMPTOMS CODED "3")?

Age at onset of Substance Dependence (CODE 99 IF UNKNOWN)

Sedatives	
Cannabis	
Stimulants	
Opioids	
Cocaine	
Hallucinogens	
Poly Drug	
Other	

IF UNCLEAR: During the past month, have you used (DRUG) at all?

IF YES: How about being high when you were at school or work, or taking care of children? How about missing something important because of being high or hung over? How about using (DRUG) while you were driving? How about getting into trouble with the law because of your use of (DRUG)?

FULL CRITERIA FOR SUBSTANCE DEPENDENCE MET AT ANY TIME IN PAST MONTH (OR NEVER HAD A MONTH WITHOUT SYMPTOMS OF DEPENDENCE OR ABUSE SINCE ONSET OF DEPENDENCE)

O ? O 1 O 3

Sedatives	O
Cannabis	O
Stimulants	O
Opioids	O
Cocaine	O
Hallucinogens	O
Poly Drug	O
Other	O

NUMBER OF MONTHS PRIOR TO INTERVIEW LAST HAD PROBLEMS WITH DRUG?

Sedatives	
Cannabis	
Stimulants	
Opioids	
Cocaine	
Hallucinogens	
Poly Drug	
Other	

SUBSTANCE ABUSE

SUBSTANCE ABUSE CRITERIA

A maladaptive pattern of substance use, leading to clinically significant impairment or distress, as manifested by one (or more) of the following, occurring at any time in the same 12-month period:

Have you ever missed work or school because you were intoxicated, high, or very hung over? (How often? What about doing a bad job at work or failing courses at school because of your [DRUG] use?)

IF NO: What about not keeping your house clean or not taking proper care of your children because of your (DRUG) use?

IF YES TO EITHER OF ABOVE: How often? Over what period of time?

	SED/ HYPN/ ANX	CANN ABIS	STIMU LANTS	OPIOID	COCAINE	HALL/ PCP	POLY	OTHER
(1) Recurrent substance use resulting in a failure to fulfill major role obligations at work, school, or home (e.g., repeated absences or poor work performance related to substance use; substance-related absences, suspensions, or expulsions from school; neglect of children or household)	O 3<br>O 2<br>O 1<br>O ?	O 3<br>O 2<br>O 1<br>O ?	O 3<br>O 2<br>O 1<br>O ?	O 3<br>O 2<br>O 1<br>O ?	O 3<br>O 2<br>O 1<br>O ?	O 3<br>O 2<br>O 1<br>O ?	O 3<br>O 2<br>O 1<br>O ?	O 3<br>O 2<br>O 1<br>O ?

Have you ever used (DRUG) in a situation in which it might have been dangerous to be using (DRUG)? Have you ever driven while you were really too high to drive?

IF YES AND UNKNOWN: How often? Over what period of time?

	SED/ HYPN/ ANX	CANN ABIS	STIMU LANTS	OPIOID	COCAINE	HALL/ PCP	POLY	OTHER
(2) Recurrent substance use in situations in which it is physically hazardous (e.g., driving	O 3	O 3	O 3	O 3	O 3	O 3	O 3	O 3

an automobile or operating a machine when impaired by substance use)	O 2	O 2	O 2	O 2	O 2	O 2	O 2	O 2
	O 1	O 1	O 1	O 1	O 1	O 1	O 1	O 1
	O ?	O ?	O ?	O ?	O ?	O ?	O ?	O ?

Has your use of (DRUG) ever gotten you into trouble with the law?

IF YES AND UNKNOWN: How often? Over what period of time?

	SED/ HYPN/ ANX	CANN ABIS	STIMU LANTS	OPIOID	COCAINE	HALL/ PCP	POLY	OTHER
(3) Recurrent substance-related legal problems (e.g., arrests for substance-related disorderly conduct)	O 3	O 3	O 3	O 3	O 3	O 3	O 3	O 3
	O 2	O 2	O 2	O 2	O 2	O 2	O 2	O 2
	O 1	O 1	O 1	O 1	O 1	O 1	O 1	O 1
	O ?	O ?	O ?	O ?	O ?	O ?	O ?	O ?

IF NOT ALREADY KNOWN: Has your use of (DRUG) caused problems with other people, such as with family members, friends, or people at work? (Did you ever get into physical fights or bad arguments about your drug use?)

IF YES: Did you keep on using (DRUG) anyway? Over what period of time?

	SED/ HYPN/ ANX	CANN ABIS	STIMU LANTS	OPIOID	COCAINE	HALL/ PCP	POLY	OTHER
(4) Continued substance use despite having persistent or recurrent social or interpersonal problems caused or exacerbated by the effects of the substance (e.g., arguments with spouse about consequences of intoxication, physical fights)	O 3	O 3	O 3	O 3	O 3	O 3	O 3	O 3
	O 2	O 2	O 2	O 2	O 2	O 2	O 2	O 2
	O 1	O 1	O 1	O 1	O 1	O 1	O 1	O 1
	O ?	O ?	O ?	O ?	O ?	O ?	O ?	O ?

FOR EACH DRUG CLASS CODED "3" BELOW, GO TO *SUBSTANCE ABUSE DIAGNOSTIC SPECIFIERS*.

IF ALL DRUG CLASSES CODED "1" BELOW, GO TO ANXIETY DISORDERS MODULE F.

IF UNKNOWN: When did (SYMPTOMS CODED "3" ABOVE) occur? (Did they all happen around the same time?)

	SED/ HYPN/ ANX	CANN ABIS	STIMU LANTS	OPIOID	COCAINE	HALL/ PCP	POLY	OTHER
Total Number of Symptoms	0	0	0	0	0	0	0	0
SUBSTANCE ABUSE AT LEAST ONE ITEM IS CODED "3" AND ITEM OCCURRED WITHIN THE SAME TWELVE-MONTH PERIOD	O 3	O 3	O 3	O 3	O 3	O 3	O 3	O 3
	O 1	O 1	O 1	O 1	O 1	O 1	O 1	O 1
Criteria Met:	false	false	false	false	false	false	false	false

SUBSTANCE ABUSE DIAGNOSTIC SPECIFIERS

LIFETIME SEVERITY:
(Additional questions regarding impairment may be necessary.)

O Mild: (GAF = 61-80) Minimum symptom criteria are met or few, if any, symptoms in excess of those required to make the diagnosis and/or symptoms result in only minimal interference in normal functioning. Either mild disability is present or substantial or unusual effort is needed to function normally.

O Moderate: (GAF = 51-60) Intermediate between "mild" and "severe."

O Severe: (GAF = 50) Several symptoms in excess of those required to make the diagnosis and/or symptoms result in only limited functioning in most aspects of life almost all the time.

O No Information

Sedatives	
Cannabis	
Stimulants	
Opioids	
Cocaine	
Hallucinogens	
Poly Drug	
Other	

CURRENT STATE:

O In Remission: This specifier is used if, for at least one month, but less than 12 months, one or more criteria for Dependence or Abuse have been met (but the full criteria for Dependence have not been met).

O Currently Meets Full Criteria:

O Currently Symptomatic

O No Information

Sedatives	
Cannabis	
Stimulants	
Opioids	
Cocaine	
Hallucinogens	
Poly Drug	
Other	

CHRONOLOGY

How old were you when you first had (LIST OF SUBSTANCE ABUSE SYMPTOMS CODED "3")?

Age at onset of Substance Dependence (CODE 99 IF UNKNOWN)

Sedatives	
Cannabis	
Stimulants	
Opioids	
Cocaine	
Hallucinogens	
Poly Drug	
Other	

IF UNCLEAR: During the past month, have you used (DRUG) at all?

IF YES: How about being high when you were at school or work, or taking care of children? How about missing something important because of being high or hung over? How about using (DRUG) while you were driving? How about getting into trouble with the law because of your use of (DRUG)?

FULL CRITERIA FOR SUBSTANCE ABUSE MET AT ANY TIME IN PAST MONTH (OR NEVER HAD A MONTH WITHOUT SYMPTOMS OF DEPENDENCE OR ABUSE SINCE ONSET OF DEPENDENCE)

O ? O 1 O 3

Sedatives	O
Cannabis	O
Stimulants	O
Opioids	O
Cocaine	O
Hallucinogens	O
Poly Drug	O
Other	O

NUMBER OF MONTHS PRIOR TO INTERVIEW LAST HAD PROBLEMS WITH DRUG?

Sedatives	
Cannabis	
Stimulants	
Opioids	

Cocaine	
Hallucinogens	
Poly Drug	
Other	

Module F: Anxiety Disorders

PANIC DISORDER

PANIC DISORDER CRITERIA

A. Both (1) and (2)

Have you ever had a panic attack, when you suddenly felt frightened or anxious or developed a lot of physical symptoms?<br><br>IF YES: Have these attacks ever come on completely out of the blue, in situations where you didn't expect to be nervous or uncomfortable?<br><br>IF UNCLEAR: How many of these kinds of attacks have you had? (At least two?)	(1) Recurrent, unexpected panic attacks	O ? O 1 O 2 O 3
After any of these attacks...<br><br>Did you worry that there might be something terribly wrong with you, like you were having a heart attack or were going crazy? (How long did you worry? At least a month?)<br><br>IF NO: Did you worry a lot about having another attack? How long did you worry? At least a month?<br><br>IF NO: Did you do anything differently because of the attacks(like avoiding certain places, going out alone, or certain activities like exercise. Were you ever concerned about things like making sure you're near a bathroom or exit?)	(2) At least one of the attacks has been followed by a month (or more) of one (or more) of the following:<br><br>(b) worry about the implications of the attack or its consequences(e.g., losing control, having a heart attack, "going crazy")<br><br>(a) persistent concern about having additional attacks<br><br>(c) a significant change in behavior related to the attacks	O ? O 1 O 2 O 3

DETERMINE IF CRITERIA ARE MET FOR A PANIC ATTACK.

When was the last bad attack? What was the first thing you noticed? Then what?<br><br>IF UNKNOWN: Did the symptoms come on all of a sudden?<br><br>IF YES: How long did it take from when it began to when it got really bad?(Less than ten minutes?)	The panic attack symptoms developed abruptly and reached a peak within ten minutes	O ? O 1 O 2 O 3

During that attack...

...did your heart race, pound or skip?	(1) palpitations, pounding heart, or accelerated heart rate	O ? O 1 O 2 O 3
...did you sweat?	(2) sweating	O ? O 1 O 2 O 3
...did you tremble or shake?	(3) trembling or shaking	O ? O 1 O 2 O 3
...were you short of breath? (Have trouble catching your breath?	(4) sensations of shortness of breath or smothering	O ? O 1 O 2 O 3
...did you feel as if you were choking?	(5) feeling of choking	O ? O 1 O 2 O 3
...did you have chest pain or pressure?	(6) chest pain or discomfort	O ? O 1 O 2 O 3
...did you have nausea, an upset stomach, or the feeling that you were going to have diarrhea?	(7) nausea or abdominal distress	O ? O 1 O 2 O 3
...did you feel dizzy, unsteady, or like you might faint?	(8) feeling dizzy, unsteady, lightheaded or faint	O ? O 1 O 2 O 3
...did things around you seem unreal or did you feel detached from things around you or from part of your body?	(9) derealization (feelings of unreality) or depersonalization(being detached from oneself)	O ? O 1 O 2 O 3
...were you afraid you were going crazy or might	(10) fear of losing control or going crazy	O O O O

lose control?		? 1 2 3
...were you afraid that you might die?	(11) fear of dying	O O O O<br>? 1 2 3
...did you have tingling or numbness in parts of your body?	(12) paresthesias (numbness or tingling sensations)	O O O O<br>? 1 2 3
...did you have flushes (hot flashes) or chills?	(13) chills or hot flushes	O O O O<br>? 1 2 3
	AT LEAST FOUR PANIC ATTACK SYMPTOMS CODED "3"	false
Just before you began having panic attacks, were you taking any drugs, caffeine, diet pills, or other medications?<br><br>How much coffee, tea, or caffeinated soda were you drinking a day?<br><br>Just before the attacks, were you physically ill?<br><br>IF YES: What did the doctor say?	C. Not due to the direct physiological effects of a substance (e.g., a drug of abuse, medication) or to a general medical condition<br><br>IF THERE IS ANY INDICATION THAT PANIC ATTACKS MAY BE SECONDARY (I.E., A DIRECT PHYSIOLOGICAL CONSEQUENCE OF A GMC OR SUBSTANCE, GO TO *GMC/SUBSTANCE,* F36, AND RETURN HERE TO MAKE A RATING OF "1" OR "3."<br><br>Etiological general medical conditions include: hyperthyroidism, hyperparathyroidism, pheochromo-cytoma, vestibular dysfunctions, seizure disorders, and cardiac conditions (e.g., arrhythmias, supraventricular tachycardia).<br><br>Etiological substances include: intoxication with central nervous stimulants (e.g., cocaine, amphetamines, caffeine) or cannabis or withdrawal from central nervous system depressants (e.g., alcohol, barbiturates) or from cocaine.	O O O<br>? 1 3
	D. The Panic Attacks are not better accounted for by another mental disorder, such as Social Phobia (e.g., occurring on exposure to feared social situations), Specific Phobia, Obsessive-Compulsive Disorder (e.g., on exposure to dirt in someone with an obsession about contamination), Posttraumatic Stress	O O O O<br>? 1 2 3

Disorder, or Separation Anxiety Disorder.

PANIC DISORDER CRITERIA A, B, C, AND D ARE CODED "3."

false

PANIC DISORDER WITH AGORAPHOBIA

B. The presence of Agoraphobia:

IF NOT OBVIOUS FROM OVERVIEW: Are there situations that make you nervous because you are afraid that you might have a panic attack? (Tell me about that.)

(1) Anxiety about being in places or situations from which escape might be difficult (or embarrassing) or in which help may not be available in the event of having an unexpected or situationally predisposed Panic Attack or panic-like symptoms.

O O O O
? 1 2 3

IF CANNOT GIVE SPECIFICS: What about being uncomfortable if you are...
...more than a certain distance from home?
...in a crowded place like a busy store, movie theatre, or restaurant?
...standing in a line?
...on a bridge?
...using public transportation like a bus, train, or subway, or driving a car?

Agoraphobic fears typically involve characteristic clusters of situations that include being outside the home alone; being in a crowd or standing in a line; being on a bridge; and traveling in a bus, train, or automobile.

Do you avoid these situations?

IF NO: When you are in one of these situations, do you feel very uncomfortable or like you might have a panic attack? Can you go into one of these situations only if you are with someone you know?)

(2) Agoraphobic situations are avoided (e.g., travel is restricted), endured with marked distress and anxiety about having a panic attack or panic-like symptoms, or are confronted only in the presence of a companion

O O O O
? 1 2 3

(3) The anxiety or phobic avoidance is not better accounted for by another mental disorder, such as Social Phobia (e.g., avoidance limited to social situations because of fear of embarrassment), Specific Phobia (e.g., avoidance limited to a single situation like elevators), Obsessive-Compulsive Disorder (e.g., avoidance of dirt in someone with an obsession about contamination), Posttraumatic Stress Disorder (e.g., avoidance of stimuli associated with a severe stressor), or Separation Anxiety Disorder (e.g., avoidance of leaving home or relatives).

O O O O
? 1 2 3

NOTE: CONSIDER SPECIFIC PHOBIA IF FEAR

IS LIMITED TO ONE OR ONLY A FEW SPECIFIC SITUATIONS OR SOCIAL PHOBIA IF FEAR IS LIMITED TO SOCIAL SITUATIONS

B(1), B(2), B(3) ALL CODED "3"

false

PANIC DISORDER DIAGNOSTIC SPECIFIERS

LIFETIME SEVERITY:

(Additional questions regarding impairment may be necessary.)

O Mild: (GAF = 61-80) Minimum symptom criteria are met or few, if any, symptoms in excess of those required to make the diagnosis and/or symptoms result in only minimal interference in normal functioning. Either mild disability is present or substantial or unusual effort is needed to function normally.

O Moderate: (GAF = 51-60) Intermediate between "mild" and "severe."

O Severe: (GAF = 50) Several symptoms in excess of those required to make the diagnosis and/or symptoms result in only limited functioning in most aspects of life almost all the time.

O No Information

CURRENT STATE:

O In Partial Remission: Symptoms are present but full criteria are not met, or there is a period withoutany significant symptoms lasting less than two months following the end of the episode.

O In Full Remission: During the past two months no significant signs or symptoms of the disturbance.

O Prior History: There is a history of the criteria having been met for the disorder but the individualis considered to have recovered from it.

O Currently Meets Full Criteria: Full Criteria for an illness must be met at the time of the interview

O Currently Symptomatic: Symptoms are present but full criteria are not met. This differs fromPartial Remission in that it does not immediately follow a period of illness (i.e., there was a symptom-free period of at least two months before the current symptoms began).

O No Information

LIFETIME COURSE:

O With Full Interepisode Recovery: This specifier is used when full remission is attained betweenmost Episodes for at least two months.

O Without Full Interepisode Recovery: This specifier is used when full remission is not attainedbetween most Episodes.

O Chronic: Continuously met criteria for a minimum of four years since onset of illness.

O N/A: Only one episode

O No Information

ONSET

O History of Substance Induced Onset: Onset of panic symptoms are related to a substance (e.g.,marijuana).

O No History of Substance Induced Onset: Onset of panic symptoms are not related to substanceuse.

O No Information

CHRONOLOGY

How old were you when you first began to have (SYMPTOMS OF PANIC DISORDER) regularly?	Age at onset of Panic Disorder (CODE 99 IF UNKNOWN)	
IF UNCLEAR: During the past month how many panic attacks have you had?	Has met symptomatic criteria for Panic Disorder during past month, i.e., recurrent unexpected panic attacks or agoraphobic avoidance	O ? O 1 O 3
When did you last have (ANY SYMPTOMS OF PANIC DISORDER)?	Number of months prior to interview when last had a symptom of Panic Disorder	

IF EVER MET CRITERIA FOR PANIC DISORDER, CHECK HERE O AND SKIP TO *SOCIAL PHOBIA*

AGORAPHOBIA WITHOUT HISTORY OF PANIC DISORDER (AWOPD)

AGORAPHOBIA WITHOUT HISTORY OF PANIC DISORDER (AWOPD) CRITERIA

A. The presence of Agoraphobia related to fear of developing panic-like symptoms (e.g., dizziness or diarrhea). Agoraphobia includes (1), (2), and (3) below:

Were you ever afraid of going out of the house alone, being alone, being in a crowd, standing in a line, or traveling on buses or trains? (What were you afraid could happen?)	(1) Anxiety about being in places or situations from which escape might be difficult (or embarrassing) or in which help may not be available in the event of having panic-like symptoms (e.g., dizziness or diarrhea). Agoraphobic fears typically involve characteristic clusters of situations that include being outside the home alone; being in a crowd or standing in a line; being on a bridge; and traveling in a bus, train, or car	O ? O 1 O 2 O 3

INDICATE FEARED SYMPTOM:

O having a limited symptom attack (a panic-like attack with less than four symptoms)

O becoming dizzy or falling

O depersonalization or derealization

O loss of bladder or bowel control

O vomiting

O fear of cardiac distress

O other (Specify)

NOTE: CONSIDER THE DIAGNOSIS OF SPECIFIC PHOBIA IF THE AVOIDANCE IS LIMITED TO ONE OR ONLY A FEW SPECIFIC SITUATIONS, OR SOCIAL PHOBIA IF THE AVOIDANCE IS LIMITED TO SOCIAL SITUATIONS

Do you avoid these situations?<br><br>IF NO: When you are in one of these situations, do you feel very uncomfortable or like you might have a panic attack? Can you go into one of these situations only if you are with someone you know?)	(2) Agoraphobic situations are avoided (e.g., travel is restricted), endured with marked distress and anxiety about having panic-like symptoms, or are only confronted in the presence of a companion.	O ? O 1 O 2 O 3
	(3) The anxiety or phobic avoidance is not better accounted for by another mental disorder, such as Social Phobia (e.g., avoidance limited to social situations because of fear of embarrassment), Specific Phobia (e.g., avoidance limited to single situations like elevators), Obsessive-Compulsive Disorder (e.g., avoidance of dirt in someone with an obsession about contamination), Posttraumatic Stress Disorder (e.g., avoidance of stimuli associated with a severe stressor), Separation Anxiety Disorder (e.g., avoidance of leaving home or relatives).	O ? O 1 O 2 O 3
	AGORAPHOBIA CRITERIA A(1), A(2), A(3) ALL CODED "3"	false
	B. Criteria have never been met for Panic Disorder	O 1 O 3
Just before you began having these fears, were you taking any drugs, caffeine, diet pills, or other medicines?<br><br>(How much coffee, tea, or caffeinated soda do you	C. The disturbance is not due to the direct physiological effects of a substance(e.g., a drug of abuse, medication(or to a general medical condition	O ? O 1 O 3

drink a day?)

Just before the fears began, were you physically ill?

IF YES: What did the doctor say?

IF THERE IS ANY INDICATION THAT THE ANXIETY MAY BE SECONDARY (I.E., A DIRECT PHYSIOLOGICAL CONSEQUENCE OF A GMC OR SUBSTANCE, GO TO *GMC/SUBSTANCE,* F36, AND RETURN HERE TO MAKE A RATING OF "1" OR "3."

Etiological general medical conditions include hyper- and hypo- thyroidism, hypoglycemia, hyperparathyroidism, pheochromocytoma, congestive heart failure, arrhythmias, pulmonary embolism, chronic obstructive pulmonary disease, pneumonia, hyperventilation, B-12 deficiency, porphyria, CNS neoplasms, vestibular dysfunction, encephalitis.

Etiological substances include intoxication with central nervous stimulants (e.g., cocaine, amphetamines, caffeine) or cannabis, hallucinogens, PCP, or alcohol, or withdrawal from central nervous system depressants (e.g., alcohol, sedatives, hypnotics) or from cocaine.

D. If an associated general medical condition is present, the fear described in criterion A is clearly in excess of that usually associated with the condition.

O ? O 1 O 2 O 3

AGORAPHOBIA WITHOUT HISTORY OF PANIC DISORDER CRITERIA A, B, C AND D ARE CODED "3"

false

AGORAPHOBIA WITHOUT HISTORY OF PANIC DISORDER DIAGNOSTIC SPECIFIERS

LIFETIME SEVERITY:

(Additional questions regarding impairment may be necessary.)

O Mild: (GAF = 61-80) Minimum symptom criteria are met or few, if any, symptoms in excess of those required to make the diagnosis and/or symptoms result in only minimal interference in normal functioning. Either mild disability is present or substantial or unusual effort is needed to function normally.

O Moderate: (GAF = 51-60) Intermediate between "mild" and "severe."

O Severe: (GAF = 50) Several symptoms in excess of those required to make the diagnosis and/or symptoms result in only limited functioning in most aspects of life almost all the time.

O No Information

CURRENT STATE:

O In Partial Remission: Symptoms are present but full criteria are not met, or there is a period withoutany significant symptoms lasting less than two months following the end of the episode.

O In Full Remission: During the past two months no significant signs or symptoms of the disturbance.

O Currently Meets Full Criteria: Full Criteria for an illness must be met at the time of the interview

O Currently Symptomatic: Symptoms are present but full criteria are not met. This differs fromPartial Remission in that it does not immediately follow a period of illness (i.e., there was a symptom-free period of at least two months before the current symptoms began).

O No Information

LIFETIME COURSE:

O With Full Interepisode Recovery: This specifier is used when full remission is attained betweenmost Episodes for at least two months.

O Without Full Interepisode Recovery: This specifier is used when full remission is not attainedbetween most Episodes.

O Chronic: Continuously met criteria for a minimum of four years since onset of illness.

O N/A: Only one episode

O No Information

CHRONOLOGY

When did you last have any (SYMPTOMS OF AGORAPHOBIA)?	Number of months prior to interview when last had a symptom of Agoraphobia	
IF UNKNOWN: How old were you when you first started having (SYMPTOMS OF AGORAPHOBIA)?	Age at onset of Agoraphobia (CODE 99 IF UNKNOWN)	
IF UNCLEAR: During the past month, have you been bothered by (AGORAPHOBIA)?	Has met criteria for Agoraphobia during past month	O ? O 1 O 3

IF EVER MET CRITERIA FOR AGORAPHOBIA,
CHECK HERE O AND SKIP TO *SOCIAL PHOBIA*

SOCIAL PHOBIA

	SOCIAL PHOBIA CRITERIA	
Was there anything that you have been afraid to do or felt uncomfortable doing in front of other people, like speaking, eating, or writing? (Tell me about it. What were you afraid would happen when you (CONFRONTED PHOBIC STIMULUS)?	A. A marked and persistent fear of one or more social or performance situations in which the person is exposed to unfamiliar people or to possible scrutiny by others. The individual fears that he or she will act in a way (or show anxiety symptoms) that will be humiliating or embarrassing.	O ? O 1 O 2 O 3

PHOBIC SITUATION(S) Check all that apply:

O public speaking

O eating in front of others

O writing in front of others

O generalized (most social situations)

O other (Specify)

NOTE: IN CHILDREN, THERE MUST BE EVIDENCE OF CAPACITY FOR

	AGE-APPROPRIATE RELATIONSHIPS WITH FAMILIAR PEOPLE AND THE ANXIETY MUST OCCUR IN PEER SETTINGS, NOT JUST IN INTERACTIONS WITH ADULTS.	
Have you always felt anxious when you (CONFRONTED PHOBIC STIMULUS)?	B. Exposure to the feared social situation almost invariably provokes anxiety, which may take the form of a situationally bound or situationally predisposed panic attack.<br><br>NOTE: IN CHILDREN, THE ANXIETY MAY BE EXPRESSED BY CRYING, TANTRUMS, FREEZING, OR SHRINKING FROM SOCIAL SITUATIONS WITH UNFAMILIAR PEOPLE.	O ? O 1 O 2 O 3
Did you think that you were more afraid of (PHOBIC ACTIVITY) than you should have been (or than made sense)?	C. The person recognizes that the fear is excessive or unreasonable.<br><br>NOTE: IN CHILDREN, THIS FEATURE MAY BE ABSENT.	O ? O 1 O 2 O 3
IF UNKNOWN: Did you go out of your way to avoid (PHOBIC SITUATIONS)?<br><br>IF NO: How hard is it for you to (CONFRONT PHOBIC SITUATIONS)?	D. The feared social or performance situations are avoided, or else endured with intense anxiety or distress.	O ? O 1 O 2 O 3
IF UNKNOWN: How much did (PHOBIC SITUATION) interfere with your life?<br><br>IF DOES NOT INTERFERE WITH LIFE: How much has the fact that you have this fear bothered you?	E. The avoidance, anxious anticipation, or distress in the feared social or performance situation(s) interferes significantly with the person's normal routine, occupational (academic) functioning, or social activities or relationships, or there is marked distress about having the phobia.	O ? O 1 O 2 O 3
IF UNDER AGE 18: How long have you had these fears?	F. In individuals under age 18 years, the duration is at least 6 months.	O ? O 1 O 2 O 3
Just before you began having these fears, were you taking any drugs, caffeine, diet pills, or other medicines? (How much coffee, tea, or caffeinated soda do you drink a day?)<br><br>Just before the fears began, were you physically ill?<br><br>IF YES: What did the doctor	G1. The fear or avoidance is not due to the direct physiological effects of a substance (e.g., a drug of abuse, a medication) or a general medical condition.<br><br>IF THERE IS ANY INDICATION THAT THE ANXIETY MAY BE SECONDARY (I.E., A DIRECT PHYSIOLOGICAL CONSEQUENCE OF THE	O ? O 1 O 3

say?

GMC OR SUBSTANCE, GO TO *GMC/SUBSTANCE,* F36, AND RETURN HERE TO MAKE A RATING OF "1" OR "3."

Etiological general medical conditions include: hyper- and hypo- thyroidism, hypoglycemia, hyperparathyroidism, pheochromocytoma, congestive heart failure, arrhythmias, pulmonary embolism, chronic obstructive pulmonary disease, pneumonia, hyperventilation, B-12 deficiency, porphyria, CNS neoplasms, vestibular dysfunction, encephalitis.

Etiological substances include: intoxication with central nervous stimulants (e.g., cocaine, amphetamines, caffeine) or cannabis, hallucinogens, PCP, or alcohol, or withdrawal from central nervous system depressants (e.g., alcohol, sedatives, hypnotics) or from cocaine.

G2. The fear or avoidance is not better accounted for by another mental disorder (e.g., Panic Disorder Without Agoraphobia, Separation Anxiety, Disorder, Body Dysmorphic Disorder, a Pervasive Developmental Disorder, or Schizoid Personality Disorder).

O	O	O	O
?	1	2	3

IF NOT ALREADY CLEAR: RETURN TO THIS ITEM AFTER COMPLETING INTERVIEW.

H. If a general medical condition or other mental disorder is present, the fear in A is unrelated to it, e.g., the fear is not of stuttering, trembling (in Parkinson's disease) or exhibiting abnormal eating behavior (in Anorexia Nervosa or Bulimia Nervosa).

NOTE: Social anxiety related to a general medical condition or other mental disorder may be indicated as Anxiety Disorder NOS (page F43)

O	O	O	O
?	1	2	3

SOCIAL PHOBIA CRITERIA A, B, C, D, E, F, G, AND H ARE CODED "3"

false

SOCIAL PHOBIA DIAGNOSTIC SPECIFIERS

LIFETIME SEVERITY:

(Additional questions regarding impairment may be necessary.)

O Mild: (GAF = 61-80) Minimum symptom criteria are met or few, if any, symptoms in excess of those required to make the diagnosis and/or symptoms result in only minimal interference in normal functioning. Either mild disability is present or substantial or unusual effort is needed to function normally.

O Moderate: (GAF = 51-60) Intermediate between "mild" and "severe."

O Severe: (GAF = 50) Several symptoms in excess of those required to make the diagnosis and/or symptoms result in only limited functioning in most aspects of life almost all the time.

O No Information

CURRENT STATE:

O In Partial Remission: Symptoms are present but full criteria are not met, or there is a period withoutany significant symptoms lasting less than two months following the end of the episode.

O In Full Remission: During the past two months no significant signs or symptoms of the disturbance.

O Currently Meets Full Criteria: Full Criteria for an illness must be met at the time of the interview

O Currently Symptomatic: Symptoms are present but full criteria are not met. This differs fromPartial Remission in that it does not immediately follow a period of illness (i.e., there was a symptom-free period of at least two months before the current symptoms began).

O No Information

LIFETIME COURSE:

O With Full Interepisode Recovery: This specifier is used when full remission is attained betweenmost Episodes for at least two months.

O Without Full Interepisode Recovery: This specifier is used when full remission is not attainedbetween most Episodes.

O Chronic: Continuously met criteria for a minimum of four years since onset of illness.

O N/A: Only one episode

O No Information

FEATURES/SUBTYPES:

O Generalized Type: This specifier is used if the fears include most social situations.

O Specific Type: This specifier is used if the fears are specific to a particular situation.

O No Information

CHRONOLOGY

When did you last have any (SYMPTOMS OF SOCIAL PHOBIA)?	Number of months prior to interview when last had a symptom of Social Phobia	
IF UNKNOWN: How old were you when you first started having (SYMPTOMS OF SOCIAL PHOBIA)?	Age at onset of Social Phobia (CODE 99 IF UNKNOWN)	
IF UNCLEAR: During the past month, have you been bothered by (SOCIAL PHOBIA)?	Has met criteria for Social Phobia during past month	O ? O 1 O 3

END OF SOCIAL PHOBIA

CONTINUE WITH SPECIFIC PHOBIA

SPECIFIC PHOBIA

SPECIFIC PHOBIA CRITERIA

Are there any other things that you have been especially afraid of, like flying, seeing blood, getting a shot, heights, closed places, or certain kinds of animals or insects? Tell me about that. What were you afraid would happen when (CONFRONTED	A. Marked and persistent fear that is excessive or unreasonable, cued by the presence or anticipation of a specific object or situation (e.g., flying, heights, animals, receiving an injection, seeing blood).	O ? O 1 O 2 O 3

WITH PHOBIC STIMULUS)?

Did you always feel frightened when you (CONFRONTED PHOBIC STIMULUS)?	B. Exposure to the phobic stimulus almost invariably provokes an immediate anxiety response, which may take the form of a situationally bound or situationally predisposed panic attack.<br><br>NOTE: IN CHILDREN, THE ANXIETY MAY BE EXPRESSED BY CRYING, TANTRUMS, FREEZING, OR CLINGING.	O O O O<br>? 1 2 3
Did you think that you were more afraid of (PHOBIC STIMULUS) than you should have been (or than made sense?)	C. The person recognizes that the fear is excessive or unreasonable. Note: in children, this feature may be absent.	O O O O<br>? 1 2 3
Did you go out of your way to avoid (PHOBIC STIMULUS)?<br><br>(Are there things you didn't do because of this fear that you would otherwise have done?)<br><br>IF NO: How hard (is/was) it for you to (CONFRONT PHOBIC STIMULUS)?	D. The phobic situation(s) is avoided, or else endured with intense anxiety or distress.	O O O O<br>? 1 2 3
IF UNKNOWN: How much did (PHOBIA) interfere with your life?<br><br>Is there anything you've avoided because of being afraid of (PHOBIC STIMULUS)?<br><br>IF DOES NOT INTERFERE WITH LIFE: How much has the fact that you were afraid of (PHOBIC STIMULUS) bothered you?	E. The avoidance, anxious anticipation, or distress in the feared situation(s) interferes significantly with the person's normal routine, occupational (or academic) functioning, or social activities or relationships, or there is marked distress about having the phobia.	O O O O<br>? 1 2 3
IF YOUNGER THAN AGE 18: How long have you had these fears?	F. In individuals under age 18 years, the duration is at least 6 months.	O O O O<br>? 1 2 3

IF NOT ALREADY CLEAR: RETURN TO THIS ITEM AFTER COMPLETING REMAINDER OF ANXIETY MODULE	G. The anxiety, Panic Attacks, or phobic avoidance associated with the specific object or situation are not better accounted for by another mental disorder, such as Obsessive-Compulsive Disorder (e.g., fear of dirt in someone with an obsession about contamination), Posttraumatic Stress Disorder (e.g., avoidance of stimuli associated with a severe stressor), Separation Anxiety Disorder (e.g., avoidance of school, Social Phobia (e.g., avoidance of social situations because of fear of embarrassment), Panic Disorder With Agoraphobia, or Agoraphobia Without History of Panic Disorder.	O ? O 1 O 2 O 3
	SPECIFIC PHOBIA CRITERIA A, B, C, D, E, F AND G ARE CODED "3"	false

SPECIFIC PHOBIA DIAGNOSTIC SPECIFIERS

LIFETIME SEVERITY:

(Additional questions regarding impairment may be necessary.)

O Mild: (GAF = 61-80) Minimum symptom criteria are met or few, if any, symptoms in excess of those required to make the diagnosis and/or symptoms result in only minimal interference in normal functioning. Either mild disability is present or substantial or unusual effort is needed to function normally.

O Moderate: (GAF = 51-60) Intermediate between "mild" and "severe."

O Severe: (GAF = 50) Several symptoms in excess of those required to make the diagnosis and/or symptoms result in only limited functioning in most aspects of life almost all the time.

O No Information

CURRENT STATE:

O In Partial Remission: Symptoms are present but full criteria are not met, or there is a period withoutany significant symptoms lasting less than two months following the end of the episode.

O In Full Remission: During the past two months no significant signs or symptoms of the disturbance.

O Currently Meets Full Criteria: Full Criteria for an illness must be met at the time of the interview

O Currently Symptomatic: Symptoms are present but full criteria are not met. This differs fromPartial Remission in that it does not immediately follow a period of illness (i.e., there was a symptom-free period of at least two months before the current symptoms began).

O No Information

LIFETIME COURSE:

O With Full Interepisode Recovery: This specifier is used when full remission is attained betweenmost Episodes for at least two months.

O Without Full Interepisode Recovery: This specifier is used when full remission is not attainedbetween most Episodes.

O Chronic: Continuously met criteria for a minimum of four years since onset of illness.

O N/A: Only one episode

O No Information

FEATURES/SUBTYPES:

O Animal Type: This specifier is used if the fears include animals (including insects).

O Natural Environment Type: This specifier is used if the fears are specific to a particular naturalsituation (e.g., storms, heights, water).

O Blood-Injection-Injury Type: This specifier is used if the fears are specific to seeing blood or injuryor receiving an injection or other invasive procedure.

O Situational Type: This specifier is used if the fears are specific to public transportation, tunnels,bridges, elevators, flying, driving, or enclosed places.

O Other Type: This specifier is used if the fears are less specific (i.e., fear of situations that might leadto choking, vomiting, or contracting an illness; in children, avoidance of loud sounds or costumedcharacters)

O No Information

CHRONOLOGY

IF UNCLEAR: During the past month, have you been bothered by (SPECIFIC PHOBIA)?	Has met criteria for Specific Phobia during past month	O ? O 1 O 3
When did you last have (ANY SYMPTOMS OF SPECIFIC PHOBIA)?	Number of months prior to interview when last had a symptom of Specific Phobia	
IF UNKNOWN: How old were you when you first started having (SYMPTOMS OF SPECIFIC PHOBIA)?	Age at onset of Specific Phobia(CODE 99 IF UNKNOWN)	

END OF SPECIFIC PHOBIA

CONTINUE WITH OBSESSIVE COMPULSIVE DISORDER

OBSESSIVE COMPULSIVE DISORDER

OBSESSIONS

Obsessions as defined by (1), (2), (3), and (4):

Now I would like to ask you if you have ever been bothered by thoughts that didn't make any sense and kept coming back to you even when you tried not to have them? (What were they?)

IF SUBJECT NOT SURE WHAT IS MEANT: ...thoughts like hurting someone even though you really didn't want to or being contaminated by germs or dirt?

(1) Recurrent and persistent thoughts, impulses, or images that are experienced, at some time during the disturbance, as intrusive and inappropriate, and that cause marked anxiety or distress

O ? O 1 O 2 O 3

(2) The thoughts, impulses, or images are not simply excessive worries about real-life problems.

O ? O 1 O 2 O 3

When you had these thoughts, did you try hard to get them out of your head? (What would you try to do?)

(3) The person attempts to ignore or suppress such thoughts, impulses, or images, or to neutralize them with some other thought or action

O ? O 1 O 2 O 3

IF UNCLEAR: Where did you think these thoughts were coming from?

(4) The person recognizes that the obsessional thoughts, impulses, or images are a product of his

O ? O 1 O 2 O 3

	or her own mind (not imposed from without as in thought insertion)	
	ALL OBSESSIONS CRITERIA 1-4 ARE CODED "3"	false
COMPULSIONS<br>Was there ever anything that you had to do over and over again and couldn't resist doing, like washing your hands again and again, counting up to a certain number, or checking something several times to make sure that you'd done it right? What did you have to do?	Compulsions as defined by (1) and (2):<br>(1) Repetitive behaviors (e.g., handwashing, ordering, checking) or mental acts (e.g., praying, counting, repeating words silently) that the person feels driven to perform in response to an obsession, or according to rules that must be applied rigidly	O ? O 1 O 2 O 3
IF UNKNOWN: Why did you have to do (COMPULSIVE ACT?) What would happen if you didn't do it?<br><br>IF UNCLEAR: How many times would you do (COMPULSIVE ACT)? How much time a day would you spend doing it?	(2) The behaviors or mental acts are aimed at preventing or reducing distress or preventing some dreaded event or situation; however, these behaviors or mental acts either are not connected in a realistic way with what they are designed to neutralize or prevent, or are clearly excessive	O ? O 1 O 2 O 3
	BOTH COMPULSIONS CRITERIA 1-2 ARE CODED "3"	false
	EITHER OBSESSIONS OR COMPULSIONS	false
	B. At some point during the course of the disorder, the person has recognized that the obsessions or compulsions are excessive or unreasonable. Note: this does not apply to children.	O ? O 1 O 2 O 3
Have you thought about (OBSESSIVE THOUGHTS) or done (COMPULSIVE ACTS) more than you should have (or than made sense)?<br><br>IF NO: How about when you first started having this problem?<br><br>What effect did this (OBSESSION OR COMPULSION) have on your life? (Did [OBSESSION OR COMPULSION] bother you a lot?<br><br>How much time do you spend on [OBSESSION OR COMPULSION]?	C. The obsessions or compulsions cause marked distress, are time-consuming (take more than an hour a day) or significantly interfere with the person's normal routing, occupational functioning, or usual social activities or relationships.	O ? O 1 O 2 O 3

IF NOT ALREADY CLEAR: RETURN TO THIS ITEM AFTER COMPLETING INTERVIEW	D. If another Axis I disorder is present, the content of the obsessions or compulsions is not restricted to it (e.g., preoccupation with food in the presence of an Eating Disorder; hair pulling in the presence of Trichotillo-mania; concern with appearance in the presence of Body Dysmorphic Disorder; preoccupation with drugs in the presence of a Substance Use Disorder; preoccupation with having a serious illness in the presence of Hypo-chondriasis; preoccupation with sexual urges or fantasies in the presence of a Paraphilia, or guilty ruminations in the presence of Major Depressive Disorder).	O O O O<br>? 1 2 3

Just before you began having (OBSESSIONS OR COMPULSIONS) were you taking any drugs or medications<br><br>Just before the (OBSESSIONS OR COMPULSIONS) started, were you physically ill? (What did the doctor say?)	E. The disturbance is not due to the direct physiological effects of a substance (e.g., a drug of abuse, medication) or to a general medical condition<br><br>IF THERE IS ANY INDICATION THAT THE ANXIETY MAY BE SECONDARY (I.E., A DIRECT PHYSIOLOGICAL CONSEQUENCE OF THE GMC OR SUBSTANCE, GO TO *GMC/ SUBSTANCE,* F36, AND RETURN HERE TO MAKE A RATING OF "1" OR "3."<br><br>Etiological general medical conditions include: certain CNS neoplasms.<br><br>Etiological substances include: intoxication with central nervous stimulants (e.g., cocaine, amphetamines)	O O O<br>? 1 3

OBSESSIVE COMPULSIVE DISORDER CRITERIA A, B, C, D, AND E ARE CODED "3" — false

OBSESSIVE COMPULSIVE DISORDER
DIAGNOSTIC SPECIFIERS

LIFETIME SEVERITY:

(Additional questions regarding impairment may be necessary.)

O Mild: (GAF = 61-80) Minimum symptom criteria are met or few, if any, symptoms in excess of those required to make the diagnosis and/or symptoms result in only minimal interference in normal functioning. Either mild disability is present or substantial or unusual effort is needed to function normally.

O Moderate: (GAF = 51-60) Intermediate between "mild" and "severe."

O Severe: (GAF = 50) Several symptoms in excess of those required to make the diagnosis and/or symptoms result in only limited functioning in most aspects of life almost all the time.

O No Information

CURRENT STATE:

O In Partial Remission: Symptoms are present but full criteria are not met, or there is a period withoutany significant symptoms

lasting less than two months following the end of the episode.

O In Full Remission: During the past two months no significant signs or symptoms of the disturbance.

O Currently Meets Full Criteria: Full Criteria for an illness must be met at the time of the interview

O Currently Symptomatic: Symptoms are present but full criteria are not met. This differs fromPartial Remission in that it does not immediately follow a period of illness (i.e., there was a symptom-free period of at least two months before the current symptoms began).

O No Information

LIFETIME COURSE:

O With Full Interepisode Recovery: This specifier is used when full remission is attained betweenmost Episodes for at least two months.

O Without Full Interepisode Recovery: This specifier is used when full remission is not attainedbetween most Episodes.

O Chronic: Continuously met criteria for a minimum of four years since onset of illness.

O N/A: Only one episode

O No Information

FEATURES/SUBTYPES:

O With Poor Insight: This specifier is used if, for most of the time during the current episode,the person does not recognize that the obsessions and compulsions are excessive or unreasonable.

O With Insight: This specifier is used if there is recognition that the obsessions and compulsionsare excessive or unreasonable.

O No Information

CHRONOLOGY

IF UNCLEAR: During the past month, have you been bothered by (OBSESSIVE/COMPULSIVE SYMPTOMS)?	Has met criteria for Obsessive Compulsive Disorder during past month	O ? O 1 O 3
When did you last have any (SYMPTOMS OF OBSESSIVE COMPULSIVE DISORDER)?	Number of months prior to interview when last had a symptom of Obsessive Compulsive Disorder	
IF UNKNOWN: How old were you when you first started having (SYMPTOMS OF OBSESSIVE COMPULSIVE DISORDER)?	Age at onset of Obsessive Compulsive Disorder(CODE 99 IF UNKNOWN)	

POSTTRAUMATIC STRESS DISORDER (Screening)

Sometimes things that are very upsetting happen to people. This includes being in a life-threatening situation, like a major disaster, serious accident, or fire; being physically assaulted or raped; seeing another person killed, dead, or badly hurt; or hearing about something horrible that has happened to someone to whom you are close. At any time during your life, have any of these kinds of things happened to you?

IF NO SUCH EVENTS,
CHECK HERE O AND GO TO *GENERALIZED ANXIETY DISORDER*

TRAUMATIC EVENTS LIST

**Brief Description**	**Date (Month/Yr)**	**Age**

IF ANY EVENTS LISTED: Sometimes these things keep coming back in nightmares, flashbacks, or thoughts that you can't get rid of. Has that ever happened to you?

IF NO: What about being very upset when you were in a situation that reminded you of one of these terrible things?

IF NO TO BOTH ABOVE (I.E., SUBJECT HAS HAD A TRAUMATIC EXPERIENCE BUT NO ADVERSE CONSEQUENCES), CHECK HERE O AND SKIP TO *GENERALIZED ANXIETY DISORDER*

POSTTRAUMATIC STRESS DISORDER CRITERIA

FOR THE FOLLOWING QUESTIONS, FOCUS ON THE TRAUMATIC EVENT(S) MENTIONED IN SCREENING QUESTION ABOVE.	A. The person has been exposed to a traumatic event in which both of the following were present:	
IF MORE THAN ONE TRAUMA WAS REPORTED: Which of these do you think affected you the most?	(1) The person experienced, witnessed, or was confronted with an event or events that involved actual or threatened death or serious injury, or a threat to the physical integrity of self or others	O O O O<br>? 1 2 3
IF UNCLEAR: How did you react when (TRAUMA) happened? (Were you very afraid or did you feel terrified or helpless?)	(2) The person's response involved intense fear, helplessness, or horror.	O O O O<br>? 1 2 3
	BOTH A CRITERIA ARE CODED "3"	false
Now I'd like to ask a few questions about specific ways that it may have affected you.<br>For example...	B. The traumatic event is persistently re-experienced in one (or more) of the following ways:	
...did you think about (TRAUMA) when you didn't want to or did thoughts about (TRAUMA) come to you suddenly when you didn't want them to?	(1) Recurrent and intrusive distressing recollections of the event, including images, thoughts or perceptions.	O O O O<br>? 1 2 3
What about having dreams about (TRAUMA)?	(2) Recurrent distressing dreams of the event	O O O O<br>? 1 2 3
What about finding yourself acting or feeling as if you were back in the situation?	(3) Acting or feeling as if the traumatic event were recurring (includes a sense of reliving the experience, illusions, hallucinations, and dissociative flashback episodes, including those that occur on awakening or when intoxicated)	O O O O<br>? 1 2 3
What about getting very upset when something reminded you of (TRAUMA)?	(4) Intense psychological distress at exposure to internal or external cues that symbolize or	O O O O<br>? 1 2 3

	resemble an aspect of the traumatic event	
What about having physical symptoms – like breaking out in a sweat, breathing heavily or irregularly, or feeling your heart pounding or racing?	(5) physiological reactivity on exposure to internal or external cues that symbolize or resemble an aspect of the traumatic event	O ? O 1 O 2 O 3
	AT LEAST ONE B CRITERIA IS CODED "3"	false
Since (THE TRAUMA)...	C. Persistent avoidance of stimuli associated with the trauma and numbing of general responsiveness (not present before the trauma), as indicated by three (or more) of the following:	
...have you made a special effort to avoid thinking or talking about what happened?	(1) efforts to avoid thoughts, feelings, or conversations associated with the trauma	O ? O 1 O 2 O 3
Have you stayed away from things or people that reminded you of (TRAUMA)?	(2) efforts to avoid activities, places, or people that arouse recollections of the trauma	O ? O 1 O 2 O 3
Have you been unable to remember some important part of what happened?	(3) inability to recall an important aspect of the trauma	O ? O 1 O 2 O 3
Have you been much less interested in doing things that used to be important to you, like seeing friends, reading books, or watching TV?	(4) markedly diminished interest or participation in significant activities	O ? O 1 O 2 O 3
Have you felt distant or cut off from others?	(5) feeling of detachment or estrangement from others	O ? O 1 O 2 O 3
Have you felt "numb" or like you no longer had strong feelings about anything or loving feelings for anyone?	(6) restricted range of affect, (e.g., unable to have loving feelings)	O ? O 1 O 2 O 3
Did you notice a change in the way you think about	(7) sense of a foreshortened future (e.g., does not	O O O O

or plan for the future?	expect to have a career, marriage, children, or a normal life span)	? 1 2 3
	AT LEAST THREE C CRITERIA ARE CODED "3"	false
Since (THE TRAUMA)...	D. Persistent symptoms of increased arousal (not present before the trauma) as indicated by two (or more) of the following:	
...have you had trouble sleeping? (What kind of trouble?)	(1) difficulty falling or staying asleep	O O O O<br>? 1 2 3
Have you been unusually irritable? What about outbursts of anger?	(2) irritability or outbursts of anger	O O O O<br>? 1 2 3
Have you had trouble concentrating?	(3) difficulty concentrating	O O O O<br>? 1 2 3
Have you been watchful or on guard even when there was no reason to be?	(4) hypervigilance	O O O O<br>? 1 2 3
Have you been jumpy or easily startled, like by sudden noises?	(5) exaggerated startle response	O O O O<br>? 1 2 3
	AT LEAST TWO D CRITERIA ARE CODED "3"	false
About how long did these problems (CITE POSITIVE PTSD SYMPTOMS) last?	E. Duration of the disturbance (symptoms in criteria B, C, and D) is more than one month	O O O O<br>? 1 2 3
	F. The disturbance causes clinically significant distress or impairment in social, occupational, or other important areas of functioning.	O O O O<br>? 1 2 3
	POSTTRAUMATIC STRESS DISORDER CRITERIA A, B, C, D, E, AND F ARE CODED "3"	false

POSTTRAUMATIC STRESS DISORDER
DIAGNOSTIC SPECIFIERS

LIFETIME SEVERITY:

(Additional questions regarding impairment may be necessary.)

O Mild: (GAF = 61-80) Minimum symptom criteria are met or few, if any, symptoms in excess of those required to make the diagnosis and/or symptoms result in only minimal interference in normal functioning. Either mild disability is present or substantial or unusual effort is needed to function normally.

O Moderate: (GAF = 51-60) Intermediate between "mild" and "severe."

O Severe: (GAF = 50) Several symptoms in excess of those required to make the diagnosis and/or symptoms result in only limited functioning in most aspects of life almost all the time.

O No Information

CURRENT STATE:

O In Partial Remission: Symptoms are present but full criteria are not met, or there is a period withoutany significant symptoms lasting less than two months following the end of the episode.

O In Full Remission: During the past two months no significant signs or symptoms of the disturbance.

O Currently Meets Full Criteria: Full Criteria for an illness must be met at the time of the interview

O Currently Symptomatic: Symptoms are present but full criteria are not met. This differs fromPartial Remission in that it does not immediately follow a period of illness (i.e., there was a symptom-free period of at least two months before the current symptoms began).

O No Information

LIFETIME COURSE:

O Acute: This specifier should be used when the duration of symptoms is less than 3 months.

O Chronic: This specifier should be used when the symptoms last 3 months or longer.

O No Information

FEATURES/SUBTYPES:

O With Delayed Onset: This specifier indicates that at least 6 months have passed betweenthe traumatic event and the onset of the symptoms.

O Without Delayed Onset: This specifier is used if onset of symptoms is within 6 months of thetraumatic event.

O No Information

CHRONOLOGY

IF UNCLEAR: During the past month, have you been bothered by (PTSD SYMPTOMS)?	Has met criteria for Posttraumatic Stress Disorder during past month	O O O<br>? 1 3
When did you last have any (SYMPTOMS OF PTSD)?	Number of months prior to interview when last had a symptom of Posttraumatic Stress Disorder	
IF UNKNOWN: How old were you when you first started having (SYMPTOMS OF PTSD)?	Age at onset of Posttraumatic Stress Disorder(CODE 99 IF UNKNOWN)	

GENERALIZED ANXIETY DISORDER

IF IN RESIDUAL PHASE OF SCHIZOPHRENIA
CHECK HERE O AND GO TO *ANXIETY DISORDER NOS*

GENERALIZED ANXIETY DISORDER CRITERIA

NOTE: THE DIAGNOSTIC CRITERIA ARE IN A DIFFERENT ORDER THAN APPEAR IN DSM-IV.

Have you ever had a time when you were particularly nervous or anxious? (Did you also worry a lot about bad things that might happen?)	A. Excessive anxiety and worry (apprehensive expectation), occurring more days than not for at least six months, about a number of events or	O O O O<br>? 1 2 3

IF YES: What did you worry about? How much did you worry about (EVENTS OR ACTIVITIES)?<br><br>IF YES: Would you say that you worried more days than not for at least six months?	activities (such as work or school performance)	
When you're worrying this way, do you find that it's hard to stop yourself?	B. The person finds it difficult to control the worry.	O O O O<br>? 1 2 3
When did this anxiety start?<br><br>[COMPARE ANSWER WITH ONSET OF MOOD OR PSYCHOTIC DISORDER.]	F(2). Does not occur exclusively during the course of a Mood Disorder, Psychotic Disorder, or a Pervasive Developmental Disorder	O O O O<br>? 1 2 3
Now I am going to ask you some questions about symptoms that often go along with being nervous.<br><br>Thinking about those periods when you're feeling nervous or anxious...	C. The anxiety and worry are associated with three (or more) of the following six symptoms (with at least some symptoms present for more days than not for the past six months):<br><br>NOTE: ONLY ONE ITEM IS REQUIRED IN CHILDREN	
...do you often feel physically restless – can't sit still?<br><br>Do you often feel keyed up or on edge?	(1) restlessness or feeling keyed up or on edge	O O O O<br>? 1 2 3
Do you often tire easily?	(2) being easily fatigued	O O O O<br>? 1 2 3
Do you have trouble concentrating or does your mind go blank?	(3) difficulty concentrating or mind going blank	O O O O<br>? 1 2 3
Are you often irritable?	(4) irritability	O O O O<br>? 1 2 3

Are your muscles often tense?	(5) muscle tension	O O O O<br>? 1 2 3
Do you often have trouble falling or staying asleep?	(6) sleep disturbance (difficulty falling or staying asleep, or restless unsatisfying sleep)	O O O O<br>? 1 2 3
	AT LEAST THREE C CRITERIA ARE CODED "3"	false
CODE BASED ON PREVIOUS INFORMATION.	D. The focus of the anxiety and worry is not confined to the features of another Axis I Disorder, e.g., the anxiety or worry is not about having a panic attack (as in Panic Disorder), being embarrassed in public (as in Social Phobia), being contaminated (as in Obsessive Compulsive Disorder), being away from home or close relatives (as in Separation Anxiety Disorder), gaining weight (as in Anorexia Nervosa), having multiple physical complaints (as in Somatization Disorder), or having a serious illness (as in Hypochon-driasis), and the anxiety or worry do not occur exclusively during Posttraumatic Stress Disorder.	O O O O<br>? 1 2 3
IF UNCLEAR: What effect has the anxiety, worry, or (PHYSICAL SYMPTOMS) had on your life? (Has it made it hard for you to do your work or be with your friends?)	E. The anxiety, worry, or physical symptoms cause clinically significant distress or impairment in social, occupational, or other important areas of functioning	O O O O<br>? 1 2 3
Just before you began having this anxiety, were you taking any drugs, caffeine, diet pills, or other medicines?<br><br>(How much coffee, tea, or caffeinated soda do you drink a day?)<br><br>Just before these problems began, were you physically ill?<br><br>IF YES: What did the doctor say?	F. Not due to the direct physiological effects of a substance (e.g., a drug of abuse, medication) or to a general medical condition<br><br>IF THRE IS ANY INDICATION THAT THE ANXIETY MAY BE SECONDARY (I.E., A DIRECT PHYSIOLOGICAL CONSEQUENCE OF A GMC OR SUBSTANCE, GO TO *GMC/SUBSTANCE,* AND RETURN HERE TO MAKE A RATING OF "1" OR "3."<br><br>Etiological general medical conditions include: hyper- and hypothyroidism, hypoglycemia, hyperparathyroidism, pheochromocytoma, congestive heart failure, arrhythmias, pulmonary embolism, chronic obstructive pulmonary disease, pneumonia, hyperventilation, B-12 deficiency, porphyria, CNS neoplasms, vestibular dysfunction, encephalitis.<br><br>Etiological substances include: intoxication with central nervous stimulants (e.g., cocaine, amphetamines, caffeine) or cannabis, hallucinogens, PCP, or alcohol, or withdrawal from	O O O<br>? 1 3

central nervous system depressants (e.g., alcohol, sedatives, hypnotics) or from cocaine.

GENERALIZED ANXIETY DISORDER CRITERIA A, B, C, D, E, AND F ARE CODED "3"

false

GENERALIZED ANXIETY DISORDER
DIAGNOSTIC SPECIFIERS

LIFETIME SEVERITY:

(Additional questions regarding impairment may be necessary.)

O Mild: (GAF = 61-80) Minimum symptom criteria are met or few, if any, symptoms in excess of those required to make the diagnosis and/or symptoms result in only minimal interference in normal functioning. Either mild disability is present or substantial or unusual effort is needed to function normally.

O Moderate: (GAF = 51-60) Intermediate between "mild" and "severe."

O Severe: (GAF = 50) Several symptoms in excess of those required to make the diagnosis and/or symptoms result in only limited functioning in most aspects of life almost all the time.

O No Information

CURRENT STATE:

O In Partial Remission: Symptoms are present but full criteria are not met, or there is a period withoutany significant symptoms lasting less than two months following the end of the episode.

O In Full Remission: During the past two months no significant signs or symptoms of the disturbance.

O Currently Meets Full Criteria: Full Criteria for an illness must be met at the time of the interview

O Currently Symptomatic: Symptoms are present but full criteria are not met. This differs fromPartial Remission in that it does not immediately follow a period of illness (i.e., there was a symptom-free period of at least two months before the current symptoms began).

O No Information

LIFETIME COURSE:

O With Full Interepisode Recovery: This specifier is used when full remission is attained betweenmost Episodes for at least two

months.

O Without Full Interepisode Recovery: This specifier is used when full remission is not attainedbetween most Episodes.

O Chronic: Continuously met criteria for a minimum of four years since onset of illness.

O N/A: Only one episode

O No Information

CHRONOLOGY

IF UNCLEAR: During the past month, have you been bothered by (GENERALIZED ANXIETY DISORDER SYMPTOMS)?	Has met criteria for Generalized Anxiety Disorder during past month	O ? O 1 O 3
When did you last have any (SYMPTOMS OF GENERALIZED ANXIETY DISORDER)?	Number of months prior to interview when last had a symptom of Generalized Anxiety Disorder	
IF UNKNOWN: How old were you when you first started having (SYMPTOMS OF GENERALIZED ANXIETY DISORDER)?	Age at onset of Generalized Anxiety Disorder(CODE 99 IF UNKNOWN)	

END OF GENERALIZED ANXIETY DISORDERS

CONTINUE WITH *GMC/SUBSTANCE INDUCED ANXIETY SYMPTOMS*

ANXIETY DISORDER DUE TO A GENERAL MEDICAL CONDITION

IF SYMPTOMS NOT TEMPORALLY ASSOCIATED WITH A GENERAL MEDICAL CONDITION, CHECK HERE O AND GO TO *SUBSTANCE-INDUCED ANXIETY DISORDER*

NOTE: THE DIAGNMOSTIC CRITERIA ARE IN A DIFFERENT ORDER THAN APPEAR IN DSM-IV.

ANXIETY DISORDER DUE TO A GMC CONDITION

CODE BASED ON INFORMATION ALREADY OBTAINED	A. Prominent anxiety, panic attacks, obsessions or compulsions predominate in the clinical picture.	O ? O 1 O 2 O 3
Did the (ANXIETY SYMPTOMS) start or get much worse only after (GMC) began?<br><br>IF GMC HAS RESOLVED: Did the (ANXIETY SYMPTOMS) get better once the (GMC) got better?	B/C. There is evidence from the history, physical examination, or laboratory findings that the disturbance is the direct physiological consequence of a general medical condition and the disturbance is not better accounted for by another mental disorder (e.g., Adjustment Disorder with Anxiety), in which the stressor is a serious general medical condition).<br><br>THE FOLLOWING FACTORS SHOULD BE CONSIDERED AND SUPPORT THE CONCLUSION THAT THE GMC IS ETIOLOGIC TO THE ANXETY SYMPTOMS.<br><br>1) THERE IS EVIDENCE FROM THE LITERATURE OF A WELL-ESTABLISHED ASSOCIATION BETWEEN THE GMC AND ANXIETY SYMPTOMS.<br><br>2) THERE IS A CLOSE TEMPORAL RELATIONSHIP BETWEEN THE COURSE OF THE ANXIETY SYMPTOMS AND THE COURSE OF THE GENERAL MEDICAL CONDITION.	O ? O 1 O 2 O 3

3) THE ANXIETY SYMPTOMS ARE CHARACTERIZED BY UNUSUAL PRESENTING FEATURES (E.G., LATE AGE AT ONSET)

4) THE ABSENCE OF ALTERNATIVE EXPLANATIONS (E.G., ANXIETY SYMPTOMS AS A PSYCHOLOGICAL REACTION TO THE GMC).

IF UNCLEAR: How much did (ANXIETY SYMPTOMS) interfere with your life?

(Has it made it hard for you to do your work or be with your friends?)

E. The disturbance causes clinically significant distress or impairment in social, occupational or other important areas of functioning.

O ? O 1 O 2 O 3

D. The disturbance does not occur exclusively during the course of Delirium.

O 1 O 3

ANXIETY DISORDER DUE TO A GMC CRITERIA A, B, C, D, AND E ARE ALL CODED "3"

false

ANXIETY DISORDER DUE TO A GMC DIAGNOSTIC SPECIFIERS

LIFETIME SEVERITY:

(Additional questions regarding impairment may be necessary.)

O Mild: (GAF = 61-80) Minimum symptom criteria are met or few, if any, symptoms in excess of those required to make the diagnosis and/or symptoms result in only minimal interference in normal functioning. Either mild disability is present or substantial or unusual effort is needed to function normally.

O Moderate: (GAF = 51-60) Intermediate between "mild" and "severe."

O Severe: (GAF = 50) Several symptoms in excess of those required to make the diagnosis and/or symptoms result in only limited functioning in most aspects of life almost all the time.

O No Information

CURRENT STATE:

O In Partial Remission: Symptoms are present but full criteria are not met, or there is a period withoutany significant symptoms lasting less than two months following the end of the episode.

O In Full Remission: During the past two months no significant signs or symptoms of the disturbance.

O Currently Meets Full Criteria: Full Criteria for an illness must be met at the time of the interview

O Currently Symptomatic: Symptoms are present but full criteria are not met. This differs fromPartial Remission in that it does not immediately follow a period of illness (i.e., there was a symptom-free period of at least two months before the current symptoms began).

O No Information

FEATURES/SUBTYPES:

O With Generalized Anxiety: This specifier may be used if excessive anxiety or worry about anumber of events or activities predominates in the clinical presentation.

O With Panic Attacks: This specifier may be used if Panic Attacks predominate in the clinicalpresentaion.

O With Obsessive-Compulsive Symptoms: This specifier may be used if obsessions orcompulsions predominate in the clinical presentation.

O With Phobic Symptoms: This specifier may be used if phobic symptoms predominate in theclinical presentation.

O Other Type: This specifier may be used if symptoms do not fall into any of the above categories.

O No Information

CHRONOLOGY

IF UNCLEAR: During the past month, have you been bothered by (ANXIETY DISORDER DUE TO A GMC SYMPTOMS)?	Has met criteria for Anxiety Disorder Due to a GMC during past month	O ? O 1 O 3
When did you last have any (SYMPTOMS OF ANXIETY DISORDER DUE TO A GMC)?	Number of months prior to interview when last had a symptom of Anxiety Disorder Due to a GMC	
IF UNKNOWN: How old were you when you first started having (SYMPTOMS OF ANXIETY DISORDER DUE TO A GMC)?	Age at onset of Anxiety Disorder Due to a GMC (CODE 99 IF UNKNOWN)	

END OF ANXIETY DISORDER DUE TO GMC

RETURN TO APPROPRIATE ANXIETY DISORDER

SUBSTANCE-INDUCED ANXIETY DISORDER

IF SYMPTOMS NOT TEMPORALLY ASSOCIATED WITH SUBSTANCE SUE,
CHECK HERE O AND RETURN TO DISORDER BEING EVALUATED.

SUBSTANCE-INDUCED ANXIETY DISORDER CRITERIA

CODE BASED ON INFORMATION ALREADY OBTAINED	A. Prominent anxiety, panic attacks, obsessions or compulsions predominate in the clinical picture.	O ? O 1 O 2 O 3

IF UNKNOWN: When did the (ANXIETY SYMPTOMS) begin? Were you already using (SUBSTANCE) or had you just stopped or cut down your use?	B. There is evidence from the history, physical examination, or laboratory findings that either: (1) the symptoms in A developed during, or within a month of, substance intoxication or withdrawal, or (2) medication use is etiologically related to the disturbance.	O ? O 1 O 2 O 3
ASK ANY OF THE FOLLOWING QUESTIONS AS NEEDED TO RULE OUT A NON-SUBSTANCE-INDUCED ETIOLOGY:<br><br>IF UNKNOWN: Which came first, the (SUBSTANCE USE) or the (ANXIETY SYMPTOMS)?<br><br>IF UNKNOWN: Have you had a period of time when you stopped using (SUBSTANCE)?<br><br>IF YES: After you stopped using (SUBSTANCE) did the (ANXIETY SYMPTOMS) get better or did they continue?<br><br>IF UNKNOWN: How much (SUBSTANCE) were you using when you began to have (ANXIETY SYMPTOMS<br><br>IF UNKNOWN: Have you had any other episodes of (ANXIETY SYMPTOMS)?<br><br>IF YES: How many? Were you using (SUBSTANCES) at those times?	C. The disturbance is NOT better accounted for by an Anxiety Disorder that is not substance-induced.<br><br>Guidelines for Primary Anxiety: Evidence that the symptoms are better accounted for by a primary (i.e., non-substance-induced) Anxiety Disorder may include any (or all) of the following:<br><br>(1) the anxiety symptoms precede the onset of the Substance Abuse or Dependence (or medication use)<br><br>(2) the anxiety symptoms persist for a substantial period of time (e.g., about a month) after the cessation of acute withdrawal or severe intoxication<br><br>(3) the anxiety symptoms are substantially in excess of what would be expected given the character, duration, or amount of the substance used<br><br>(4) there is evidence suggesting the existence of an independent non-substance-induced Anxiety Disorder (e.g., a history of recurrent non-substance-related panic attacks)	O ? O 1 O 2 O 3
IF UNKNOWN: How much did (ANXIETY SYMPTOMS) interfere with your life?<br><br>(Has it made it hard for you to do your work or be with your friends?)	E. The symptoms cause clinically significant distress or impairment in social, occupational, or other important areas of functioning.	O ? O 1 O 2 O 3
	D. The disturbance does not occur exclusively during the course of Delirium.	O 1 O 3

SUBSTANCE INDUCED ANXIETY DISORDER
CRITERIA A, B, C, D, AND E ARE ALL CODED
"3"

false

SUBSTANCE INDUCED ANXIETY DISORDER
DIAGNOSTIC SPECIFIERS

LIFETIME SEVERITY:

(Additional questions regarding impairment may be necessary.)

O Mild: (GAF = 61-80) Minimum symptom criteria are met or few, if any, symptoms in excess of those required to make the diagnosis and/or symptoms result in only minimal interference in normal functioning. Either mild disability is present or substantial or unusual effort is needed to function normally.

O Moderate: (GAF = 51-60) Intermediate between "mild" and "severe."

O Severe: (GAF = 50) Several symptoms in excess of those required to make the diagnosis and/or symptoms result in only limited functioning in most aspects of life almost all the time.

O No Information

CURRENT STATE:

O In Partial Remission: Symptoms are present but full criteria are not met, or there is a period withoutany significant symptoms lasting less than two months following the end of the episode.

O In Full Remission: During the past two months no significant signs or symptoms of the disturbance.

O Currently Meets Full Criteria: Full Criteria for an illness must be met at the time of the interview

O Currently Symptomatic: Symptoms are present but full criteria are not met. This differs fromPartial Remission in that it does not immediately follow a period of illness (i.e., there was a symptom-free period of at least two months before the current symptoms began).

O No Information

LIFETIME COURSE:

O With Full Interepisode Recovery: This specifier is used when full remission is attained betweenmost Episodes for at least two months.

O Without Full Interepisode Recovery: This specifier is used when full remission is not attainedbetween most Episodes.

O Chronic: Continuously met criteria for a minimum of four years since onset of illness.

O N/A: Only one episode

O No Information

FEATURES/SUBTYPES:

O With Generalized Anxiety: This specifier may be used if excessive anxiety or worry about anumber of events or activities predominates in the clinical presentation.

O With Panic Attacks: This specifier may be used if Panic Attacks predominate in the clinicalpresentaion.

O With Obsessive-Compulsive Symptoms: This specifier may be used if obsessions orcompulsions predominate in the clinical presentation.

O With Phobic Symptoms: This specifier may be used if phobic symptoms predominate in theclinical presentation.

O Other Type: This specifier may be used if symptoms do not fall into any of the above categories.

O No Information

ONSET:

O With Onset During Intoxication: This specifier should be used if criteria for intoxication with thesubstance are met and the symptoms develop during the intoxication syndrome.

O With Onset During Withdrawal: This specifier should be used if criteria for withdrawal from thesubstance are met and the

symptoms develop during, or shortly after, a withdrawal syndrome.

O No Information

CHRONOLOGY

IF UNCLEAR: During the past month, have you been bothered by (SUBSTANCE INDUCED ANXIETY DISORDER SYMPTOMS)?	Has met criteria for Substance Induced Anxiety Disorder during past month	O ? O 1 O 3
When did you last have any (SYMPTOMS OF SUBSTANCE INDUCED ANXIETY DISORDER)?	Number of months prior to interview when last had a symptom of Substance Induced Anxiety Disorder	
IF UNKNOWN: How old were you when you first started having (SYMPTOMS OF SUBSTANCE INDUCED ANXIETY DISORDER)?	Age at onset of Substance Induced Anxiety Disorder(CODE 99 IF UNKNOWN)	

END OF SUBSTANCE-INDUCED ANXIETY DISORDER

RETURN TO APPROPRIATE ANXIETY DISORDER

ANXIETY DISORDER NOT OTHERWISE SPECIFIED

ANXIETY DISORDER NOT OTHERWISE SPECIFIED CRITERIA

This category includes disorders with prominent anxiety or phobic avoidance that do not meet criteria for any specific Anxiety Disorder, Adjustment Disorder With Anxiety, or Adjustment Disorder With Mixed Anxiety and Depressed Mood.

O ? O 1 O 2 O 3

Just before you began having this anxiety, were you taking any drugs, stimulants or medicines?<br><br>(How much coffee, tea, or caffeinated soda do you drink a day)?<br><br>Just before these problems began, were you physically ill? (What did the doctor say?)	Clinically significant anxiety or phobic avoidance that does not meet criteria for any specific Anxiety Disorder, Adjustment Disorder With Anxiety, or Adjustment Disorder with Mixed Anxiety and Depressed Mood. (See Module I to rule out Adjustment Disorder)<br><br>Not due to the direct physiological effects of a substance (e.g., a drug of abuse, medication) or to a general medical condition	O ? O 1 O 3

IF A GENERAL MEDICAL CONDITION OR SUBSTANCE MAY BE ETIOLOGICALLY ASSOCIATED WITH THE ANXIETY, GO TO *GMC/SUBSTANCE*, F. 36 AND RETURN HERE TO MAKE RATING OF "1" OR "3."

Etiological general medical conditions include: hyper- and hypothyroidism, hypoglycemia, hyperparathyroidism, pheochromoctyoma, congestive heart failure, arrhythmias, pulmonary embolism, chronic obstructive pulmonary disease, pneumonia, hyperventilation, B-12 deficiency, porphyria, CNS neoplasms, vestibular dysfunction, encephalitis.

Etiological substances include: intoxication with central nervous stimulants (e.g., cocaine,

amphetamines, caffeine) or cannabis, hallucinogens, PCP, or alcohol, or withdrawal from central nervous system depressants (e.g., alcohol, sedatives, hypnotics) or from cocaine.

ANXIETY DISORDER NOS DIAGNOSTIC SPECIFIERS

LIFETIME SEVERITY:

(Additional questions regarding impairment may be necessary.)

O Mild: (GAF = 61-80) Minimum symptom criteria are met or few, if any, symptoms in excess of those required to make the diagnosis and/or symptoms result in only minimal interference in normal functioning. Either mild disability is present or substantial or unusual effort is needed to function normally.

O Moderate: (GAF = 51-60) Intermediate between "mild" and "severe."

O Severe: (GAF = 50) Several symptoms in excess of those required to make the diagnosis and/or symptoms result in only limited functioning in most aspects of life almost all the time.

O No Information

CURRENT STATE:

O In Partial Remission: Symptoms are present but full criteria are not met, or there is a period withoutany significant symptoms lasting less than two months following the end of the episode.

O In Full Remission: During the past two months no significant signs or symptoms of the disturbance.

O Currently Meets Full Criteria: Full Criteria for an illness must be met at the time of the interview

O Currently Symptomatic: Symptoms are present but full criteria are not met. This differs fromPartial Remission in that it does not immediately follow a period of illness (i.e., there was a symptom-free period of at least two months before the current symptoms began).

O No Information

END OF MODULE F

GO TO MODULE H

Module H: Eating Disorders

ANOREXIA NERVOSA

ANOREXIA NERVOSA CRITERIA

Now I would like to ask you some questions about your eating habits and your weight.

Have you ever had a time when you weighed much less than other people thought you ought to weigh?

A. Refusal to maintain body weight at or above a minimally normal weight for age and height (e.g., weight loss leading to maintenance of body weight less than 85% of that expected; or failure to make expected weight gain during period of growth, leading to body weight less than 85% of that expected)

O ? O 1 O 2 O 3

IF YES: Why was that?

How much did you weigh? WEIGHT =

How old were you then? AGE =

How tall were you? HEIGHT =

Interviewer : Note body frame

SELECT ONE: O Small O Medium O Large

[See chart for comparison]

At that time, were you very afraid that you could become fat?

B. Intense fear of gaining weight or becoming fat, even though underweight.

O ? O 1 O 2 O 3

At your lowest weight, did you still feel too fat or that part of your body was too fat?

IF NO: Did you need to be very thin in order to feel good about yourself?

IF NOT AND LOW WEIGHT IS MEDICALLY SERIOUS: When you were that thin, did anybody tell you it could be dangerous to your health to be that thin? What did you think?

C. Disturbance in the way in which one's body weight or shape is experienced; undue influence of body weight or shape on self-evaluation, or denial of the seriousness of the current low body weight

O ? O 1 O 2 O 3

FOR FEMALES: Before this time, were you having menstrual periods? (Did they stop? For how long?)	D. In postmenarchal females, amenorrhea, i.e., the absence of at least three consecutive menstrual cycles. (A woman is still considered to have amenorrhea if her periods occur only following hormone, e.g., estrogen administration)	O ? O 1 O 2 O 3
	ANOREXIA NERVOSA CRITERIA A, B, C, AND D ARE CODED "3"	false

ANOREXIA NERVOSA DIAGNOSTIC SPECIFIERS

Did you have eating binges in which you would eat a lot of food in a short period of time and feel that your eating was out of control? (How often?)<br><br>IF NO: What kinds of things have you done to keep weight off? Ever made yourself vomit or take laxatives, enemas, or water pills? How often?	FEATURES/SUBTYPES:<br>During the course of Anorexia Nervosa, the person has regularly engaged in binge-eating or purging behavior (i.e., self-induced vomiting or misuse of laxatives, diuretics, or enemas)<br><br>USE IN COMPLETING FEATURES/SUBTYPES SPECIFIER ON THE DIAGNOSTIC SCORE SHEET.	O 1 O 3

LIFETIME SEVERITY:

(Additional questions regarding impairment may be necessary.)

O Mild: (GAF = 61-80) Minimum symptom criteria are met or few, if any, symptoms in excess of those required to make the diagnosis and/or symptoms result in only minimal interference in normal functioning. Either mild disability is present or substantial or unusual effort is needed to function normally.

O Moderate: (GAF = 51-60) Intermediate between "mild" and "severe."

O Severe: (GAF symptoms result in only limited functioning in most aspects of life almost all the time.

O No Information

CURRENT STATE:

O In Partial Remission: Symptoms are present but full criteria are not met, or there is a periodwithout any significant symptoms lasting less than two months following the end of the episode.

O In Full Remission: During the past two months no significant signs or symptoms of thedisturbance.

O Current: Full Criteria for an illness must be met at the time of the interview

O No Information

CHRONOLOGY

When did you last have any (SYMPTOMS OF ANOREXIA NERVOSA)?	Number of months prior to interview when last had a symptom of Anorexia Nervosa	
IF UNKNOWN: How old were you when you first started having (SYMPTOMS OF ANOREXIA NERVOSA)?	Age at onset of Anorexia Nervosa (CODE 99 IF UNKNOWN)	

IF CRITERIA CURRENTLY MET FOR ANOREXIA NERVOSA, CHECK HERE O AND SKIP TO MODULE I.

BULIMIA NERVOSA

BULIMIA NERVOSA CRITERIA

Have you often had times when your eating was out of control? (Tell me about those times.)	A. Recurrent episodes of binge eating. An episode of binge eating is characterized by BOTH of the following:	
	(2) a sense of lack of control over eating during the episode (e.g., a feeling that one cannot stop eating or control what or how much one is eating)	O ? O 1 O 2 O 3
IF UNKNOWN: During these times, do you often eat within any two hour period what most people would regard as an unusual amount of food? (Tell me about that.)	(1) eating, in a discrete period of time (e.g., within any two hour period), an amount of food that is definitely larger than most people would eat during a similar period of time and under similar circumstances.	O ? O 1 O 2 O 3
	BOTH CRITERION A ITEMS 1 AND 2 ARE CODED "3"	false
Did you do anything to counteract the effects of eating that much? (Like making yourself vomit, taking laxatives, enemas, or water pills, strict dieting or fasting, or exercising a lot?)	B. Recurrent inappropriate compensatory behavior in order to prevent weight gain, such as: self-induced vomiting; misuse of laxatives, diuretics, enemas, or other medications; fasting; or excessive exercise.<br><br>USE THIS INFORMATION TO ANSWER FEATURES/SUBTYPES IN DIAGNOSTIC SPECIFIERS.	O ? O 1 O 2 O 3
How often were you eating that much and (COMPENSATORY BEHAVIOR)? (At least twice a week for at least three months?)	C. The binge eating and inappropriate compensatory behaviors both occur, on average, at least twice a week for three months.	O ? O 1 O 2 O 3
Were your body shape and weight among the most important things that affected how you felt about yourself?	D. Self-evaluation is unduly influenced by body shape and weight.	O ? O 1 O 2 O 3

E. The disturbance does not occur exclusively during episodes of Anorexia Nervosa

O ? O 1 O 2 O 3

BULIMIA NERVOSA CRITERIA A, B, C, D AND E ARE CODED "3"

false

BULIMIA NERVOSA DIAGOSTIC SPECIFIERS

LIFETIME SEVERITY:

(Additional questions regarding impairment may be necessary.)

O Mild: (GAF = 61-80) Minimum symptom criteria are met or few, if any, symptoms in excess of those required to make the diagnosis and/or symptoms result in only minimal interference in normal functioning. Either mild disability is present or substantial or unusual effort is needed to function normally.

O Moderate: (GAF = 51-60) Intermediate between "mild" and "severe."

O Severe: (GAF symptoms result in only limited functioning in most aspects of life almost all the time.

O No Information

CURRENT STATE:

O In Partial Remission: Symptoms are present but full criteria are not met, or there is a periodwithout any significant symptoms lasting less than two months following the end of the episode.

O In Full Remission: During the past two months no significant signs or symptoms of thedisturbance.

O Current: Full Criteria for an illness must be met at the time of the interview

O No Information

FEATURES/SUBTYPES:

O Purging Type: This subtype describes presentations in which the person has regularly engaged inself-induced vomiting or the misuse of laxatives, diuretics, or enemas during the course of illness.

O Nonpurging Type: This subtype describes presentations in which the person has used otherinappropriate compensatory behaviors, such as fasting or excessive exercise, but has not regularlyengaged in self-induced vomiting or the misuse of laxatives, diuretics, or enemas during the courseof illness.

O No Information

CHRONOLOGY

When did you last have any (SYMPTOMS OF BULIMIA NERVOSA)?	Number of months prior to interview when last had a symptom of Bulimia Nervosa	
IF UNKNOWN: How old were you when you first started having (SYMPTOMS OF BULIMIA NERVOSA?)	Age at onset of Bulimia Nervosa(CODE 99 IF UNKNOWN)	

IF MET CRITERIA FOR BULIMIA NERVOSA, CHECK HERE O AND GO TO MODULE I.

EATING DISORDER NOS

EATING DISORDER NOS CRITERIA

The Eating Disorder NOS category is for disorders of eating that do not meet the criteria for any specific Eating Disorder. O 1 O 3

EATING DISORDER NOS DIAGNOSTIC SPECIFIERS

LIFETIME SEVERITY:

(Additional questions regarding impairment may be necessary.)

O Mild: (GAF = 61-80) Minimum symptom criteria are met or few, if any, symptoms in excess of those required to make the diagnosis and/or symptoms result in only minimal interference in normal functioning. Either mild disability is present or substantial or unusual effort is needed to function normally.

O Moderate: (GAF = 51-60) Intermediate between "mild" and "severe."

O Severe: (GAF symptoms result in only limited functioning in most aspects of life almost all the time.

O No Information

CURRENT STATE:

O In Partial Remission: Symptoms are present but full criteria are not met, or there is a periodwithout any significant symptoms lasting less than two months following the end of the episode.

O In Full Remission: During the past two months no significant signs or symptoms of thedisturbance.

O Current: Full Criteria for an illness must be met at the time of the interview

O No Information

FEATURES/SUBTYPES:

O Regular Menses: This subtype describes presentations in which, for females, all of the criteria forAnorexia Nervosa are met except that the individual has regular menses.

O Normal Weight: This subtype describes presentations in which all the criteria for AnorexiaNervosa are met except that despite significant weight loss, the individual’s current weight is in thenormal range.

O Less Frequent: This subtype describes presentations in which all the criteria for Bulimia Nervosaare met except that the binge eating and inappropriate compensatory mechanisms occur at afrequency of less than twice a week for a duration of less than three months.

O Small Amounts: The regular use of inappropriate compensatory behavior by an individual ofnormal body weight after eating small amounts of food (e.g., self-induced vomiting after theconsumption of two cookies).

O Chewing/Spitting: Repeatedly chewing and spitting out, but not swallowing, large amounts offood.

O Binge-Eating Disorder: Recurrent episodes of binge eating in the absence of regular use ofinappropriate compensatory behaviors characteristic of Bulimia Nervosa.

O Other(describe below)

O No Information

CHRONOLOGY

When did you last have any (SYMPTOMS OF EATING DISORDER NOS)?	Number of months prior to interview when last had a symptom of Eating Disorder NOS	
IF UNKNOWN: How old were you when you first started having (SYMPTOMS OF EATING DISORDER NOS)?	Age at onset of Eating Disorder NOS (CODE 99 IF UNKNOWN)	

GO TO MODULE I

Module I: Adjustment Disorder

Instructions

CONSIDER THIS SECTION ONLY IF THERE IS A CURRENT DISTURBANCE AND IT DOES NOT MEET THE CRITERIA FOR ANOTHER AXIS I DSM-IV DISORDER; OTHERWISE

CHECK HERE O AND GO TO MODULE K.

INFORMATION OBTAINED FROM OVERVIEW OF PRESENT ILLNESS WILL USUALLY BE SUFFICIENT TO RATE THE CRITERIA.

ADJUSTMENT DISORDER (CURRENT ONLY)

	ADJUSTMENT DISORDER CRITERIA	
IF UNKNOWN: Did anything happen to you just before (ONSET OF CURRENT DISTURBANCE)	A. The development of emotional or behavioral symptoms in response to an identifiable stressor(s) occurring within three months of the onset of the stressor(s).	O ? O 1 O 2 O 3
IF YES: Do you think that (STRESSOR) had anything to do with your getting (SYMPTOMS)?	DESCRIBE:	
What effect has (SYMPTOMS) had on you and your ability to do things?<br><br>How upset were you?<br><br>Has it made it hard for you to do your work or be with friends?	B. These symptoms or behaviors are clinically significant as evidenced by either of the following:<br>1) marked distress that is in excessof what would be expected from exposure to the stressor<br><br>2) significant impairment in social or occupational (academic) functioning	O ? O 1 O 2 O 3
Have you had this kind of reaction many times before?<br><br>Were you having these (SYMPTOMS) even before (STRESSOR) happened?	C. The stress-related disturbance does not meet the criteria for another specific Axis I disorder and is not merely an exacerbation of a pre-existing Axis I or Axis II disorder.	O ? O 1 O 2 O 3
IF UNKNOWN: Did someone close to you die just before (ONSET OF CURRENT DISTURBANCE)?	D. The symptoms do not represent Bereavement.	O ? O 1 O 2 O 3

How long has it been now since (STRESSOR AND COMPLICA-TIONS ARISING FROM THE STRESSOR) was over?	E. Once the stressor (or its consequen-ces) has terminated, the symptoms do not persist for more than an additional 6 months.	O ? O 1 O 2 O 3

ADJUSTMENT DISORDER CRITERIA A, B, C, D, AND E ARE CODED "3" — false

ADJUSTMENT DISORDER DIAGNOSTIC SPECIFIERS

LIFETIME SEVERITY:

(Additional questions regarding impairment may be necessary.)

- O Mild: (GAF = 61-80) Minimum symptom criteria are met or few, if any, symptoms in excess of those required to make the diagnosis and/or symptoms result in only minimal interference in normal functioning. Either mild disability is present or substantial or unusual effort is needed to function normally.
- O Moderate: (GAF = 51-60) Intermediate between "mild" and "severe."
- O Severe: (GAF symptoms result in only limited functioning in most aspects of life almost all the time.
- O No Information

CURRENT STATE:

- O In Remission: No significant signs or symptoms of the disturbance.
- O Currently Meets Full Criteria: Full Criteria for an illness must be met at the time of the interview.
- O No Information

LIFETIME COURSE:

- O Acute: This specifier can be used to indicate persistence of symptoms for less than six months.
- O Without Full Interepisode Recovery: Full remission is not attained between most episodes.
- O Chronic: This specifier can be used to indicate persistence of full symptoms for 6 months or longer.By definition, symptoms cannot persist for more than 6 months after the termination of the stressor orits consequences. The Chronic specifier therefore applies when the duration of the disturbance islonger than 6 months in response to a chronic stressor or to a stressor that has enduringconsequences.
- O N/A: Only one episode
- O No Information

FEATURES/SUBTYPES:

- O With Depressed Mood: This subtype should be used when the predominant manifestations aresymptoms such as depressed mood, tearfulness, or feelings of hopelessness.
- O With Anxiety: This subtype should be used when the predominant manifestations are symptomssuch as nervousness, worry, or jitteriness or, in children, fears of separation from major attachmentfigures.
- O With Mixed Anxiety and Depressed Mood: This subtype should be used when the predominantmanifestation is a combination of depression and anxiety.
- O With Disturbance of Conduct: This subtype should be used when the predominant manifestation isa disturbance in conduct in which there is a violation of the rights of others or of major age-appropriate societal norms and rules (e.g., truancy, vandalism, reckless driving, fighting, defaulting onlegal responsibilities).

O With Mixed Disturbance of Emotions and Conduct: This subtype should be used when thepredominant manifestations are both emotional symptoms (e.g., depression, anxiety) and adisturbance of conduct (see above subtype).

O Unspecified: This subtype should be used for maladaptive reactions (e.g., physical complaints,social withdrawal, or work or academic inhibition) to psychosocial stressors that are not classifiable asone of the specific subtypes of Adjustment Disorder.

O No Information

GO TO MODULE K

Module K: Childhood and Adolescence Disorders

ATTENTION DEFICIT/ HYPERACTIVITY DISORDER

ASK BOTH SCREENING QUESTIONS BELOW TO DETERMINE WHETHER OR NOT TO COMPLETE THE FORMAL CRITERIA FOR ATTENTION DEFICIT/HYPERACTIVITY DISORDER.

Screening questions:	ATTENTION DEFICIT/ HYPERACTIVITY DISORDER CRITERIA	
When you were age 13 or younger, was there ever a time when you had a lot of trouble paying attention in school or a time when little distractions made it very hard for you to keep your mind on what you were doing?	(1) General screening for attention deficit	O ? O 1 O 2 O 3
When you were age 13 or younger, was there ever a time when you had a lot of difficulty staying seated when you were supposed to or a time when you got into trouble because you didn’t think before you acted?	(2) General screening for hyperactivity	O ? O 1 O 2 O 3
	EITHER SCREENING QUESTION (1) OR (2) (OR BOTH) ARE CODED "3"	false

ADHD Criteria

When you were age 13 or younger, was there ever a six month period when you often did any of the following:	A. Either (1) or (2)	
	**Inattention**	
	(1) Six (or more) of the following symptoms of inattention have persisted for at least 6 months to a degree that is maladaptive and inconsistent with developmental level:	
Made a lot of careless mistakes at school, like not reading the instructions, leaving questions blank on accident, etc.?	(a) Often fails to give close attention to details or makes careless mistakes in schoolwork, work, or other activities	O ? O 1 O 2 O 3
Were you easily distracted when trying to complete a task or while playing a game?<br><br>Did you have trouble sticking to one activity or when you were playing or doing one thing, did you often stop what you were doing because you'd think of something else you'd rather do?	(b) Often has difficulty sustaining attention in tasks or play activities	O ? O 1 O 2 O 3
Did you "tune people out" or did your parents or teachers complain that you didn't listen to them when they talked to you?	(c) Often does not seem to listen when spoken to directly	O ? O 1 O 2 O 3
Did you often leave projects incomplete or did you have a hard time following through on things?<br><br>Did your parents or teachers complain that you didn't follow instructions?	(d) Often does not follow through on instructions and fails to finish schoolwork, chores, or duties in the workplace (not due to oppositional behavior)	O ? O 1 O 2 O 3
Did you often have trouble organizing tasks and activities or did other people tell you that you were disorganized?<br><br>Was your desk or locker at school a mess, to the	(e) Often has difficulties organizing tasks and activities	O ? O 1 O 2 O 3

point you had difficulty finding the things you needed or did your teachers complain that your assignments were messy and disorganized?

Did you dislike tasks or activities that required a lot of attention?	(f) Often avoids, dislikes, or is reluctant to engage in tasks that require sustained mental effort (such as schoolwork or homework)	O ? O 1 O 2 O 3
Did you lose things a lot like homework assignments or things around your home?	(g) Often loses things necessary for tasks or activities (e.g., toys, school assignments, pencils, books or tools)	O ? O 1 O 2 O 3
Were you easily distracted by things going on around you?	(h) Often easily distracted by extraneous stimuli	O ? O 1 O 2 O 3
Did you often leave your homework at home or leave things outside by accident?<br><br>Were you often forgetful throughout your day or did other people tell you that you were forgetful?	(i) Often forgetful in daily activities	O ? O 1 O 2 O 3
	SIX OR MORE INATTENTION SYMPTOMS ENDORSED FOR A1	false

Hyperactivity

	(2) Six (or more) of the following symptoms of hyperactivity-impulsivity have persisted for at least 6 months to a degree that is maladaptive and inconsistent with developmental level:	
Did you have a hard time keeping your arms and legs still or did people often tell you to sit still, to stop moving, or to stop squirming in your seat?	(a) Often fidgets with hands or feet or squirms in seat	O O O O<br>? 1 2 3
Did you often leave your seat when you were not supposed to in school or in other places where being seated was required?<br><br>Did you often get into trouble for this?	(b) Often leaves seat in classroom or in other situations in which remaining seated is expected	O O O O<br>? 1 2 3
Did your parents often have to remind you to walk instead of run when you were out together or did your parents or teachers complain about you climbing things you shouldn’t?	(c) Often runs about or climbs excessively in situations in which it is inappropriate (in adolescents or adults, may be limited to subjective feelings of restlessness)	O O O O<br>? 1 2 3
Did you have a hard time playing quietly or did your parents or teachers often tell you to quiet down when you were playing?	(d) Often has difficulty playing or engaging in leisure activities quietly	O O O O<br>? 1 2 3
Was it hard for you to slow down or stay in one place for very long, or did people tell you to slow down a lot?	(e) Often "on the go" or often acts as if "driven by a motor"	O O O O<br>? 1 2 3
Did people say you talked too much or did you get in trouble at school for talking when you weren’t supposed to?	(f) Often talks excessively	O O O O<br>? 1 2 3

Impulsivity

Did you talk out of turn at home or did you sometimes call out the answers before you were called on at school?	(g) Often blurts out answers before questions have been completed	O O O O<br>? 1 2 3
Was it hard for you to wait your turn in games or in line at the water fountain or in the cafeteria?	(h) Often has difficulty awaiting turn	O O O O<br>? 1 2 3
Did your parents, teachers, or kids you knew complain that you cut them off when they were talking?	(i) Often interrupts or intrudes on others (e.g., butts into conversations or games)	O O O O<br>? 1 2 3
	SIX OR MORE HYPERACTIVITY-IMPULSIVITY SYMPTOMS ENDORSED FOR A2	false
	**MET CRITERIA FOR EITHER A1 OR A2 (OR BOTH)**	false
Did you have any of these experiences to the point it caused problems for you and/or your family before you were seven years old?	B. Some hyperactive-impulsive or inattentive symptoms that caused impairment were present before age 7.	O O O O<br>? 1 2 3
Did these behaviors cause problems for you in at least two areas of your life (like at school and at home)?	C. Some impairment from the symptoms is present in two or more settings (e.g., at school [or work] and at home).	O O O O<br>? 1 2 3

[IF NOT YET KNOWN]<br>How did these behaviors impact your functioning?	D. There must be clear evidence of clinically significant impairment in social, academic, or occupational functioning.	O ? O 1 O 2 O 3
	E. The symptoms do not occur exclusively during the course of Pervasive Developmental Disorder, Schizophrenia, or other Psychotic Disorder and are not better accounted for by another mental disorder (e.g., Mood Disorder, Anxiety Disorder, Dissociative Disorder, or a Personality Disorder).	O ? O 1 O 2 O 3
	ATTENTION DEFICIT/HYPER-ACTIVITY DISORDER CRITERIA A, B, C, D, AND E ARE CODED "3"	false

ATTENTION DEFICIT/ HYPERACTIVITY DISORDER DIAGNOSTIC SPECIFIERS
LIFETIME SEVERITY:

(Additional questions regarding impairment may be necessary.)

O Mild: (GAF = 61-80) Minimum symptom criteria are met or few, if any, symptoms in excess of those required to make the diagnosis and/or symptoms result in only minimal interference in normal functioning. Either mild disability is present or substantial or unusual effort is needed to function normally.

O Moderate: (GAF = 51-60) Intermediate between "mild" and "severe."

O Severe: (GAF symptoms result in only limited functioning in most aspects of life almost all the time.

O No Information

CURRENT STATE:

O In Partial Remission: This specifier applies when there has been a period of ADHD symptoms, and some clinically significant residual symptoms remain for less than two months following the end of the episode.

O In Full Remission: This specifier applies when there has been a period of ADHD synptoms, and no clinically significant residual symptoms remain for the past two months.

O Prior History: This specifier applies when there is a history of the criteria having been met for thedisorder but the individual is considered to have recovered from it.

O Currently Meets Full Criteria: Full Criteria for an illness must be met at the time of the interview.

O Currently Symptomatic: Symptoms are present but full criteria are not met. This differs fromPartial Remission in that it does not immediately follow a period of illness (i.e., there was asymptom-free period of at least two months before the current symptoms began).

O No Information

LIFETIME COURSE:

O With Full Interepisode Recovery: Full remission is attained between most Mood Episodes for at least two months.

O Without Full Interepisode Recovery: Full remission is not attained between most Mood Episodes.

O Chronic: Continuously met criteria for a minimum of two years since onset of illness.

O N/A: Only one episode

O No Information

FEATURES/SUBTYPES:

O Combined Type: This subtype should be used when both criteria A1 and A2 were met for atleast six months. Most children and adolescents with the disorder have the Combined Type. It isnot known whether the same is true for adults with the disorder.

O Predominantly Inattentive Type: This subtype should be used if criterion A1 was met butcriterion A2 was not met for at least six months.

O Predominantly Hyperactive-Impulsive Type: This subtype should be used if criterion A2 wasmet but criterion A1 was not met for at least six months. Inattention may often still be asignificant clinical feature in such cases.

O No Information

IF UNKNOWN: How old were you when you first started having (SYMPTOMS OF ADHD)?

Age at onset of ADHD (CODE 99 IF UNKNOWN)

GO TO MODULE L

Module L: Personality Disorders

INSTRUCTIONS FOR THIS SECTION

PLEASE ASSESS THE SUBJECT FOR PRESENCE OF BORDERLINE PERSONALITY DISORDER AND/OR ANTISOCIAL PERSONALITY DISORDER WITH THIS QUESTIONNAIRE.

THE DIAGNOSTIC CRITERIA FOR THE PERSONALITY DISORDERS REFER TO BEHAVIORS OR TRAITS THAT ARE CHARACTISTERIC OF THE PERSON'S RECENT AND LONG-TERM FUNCTIONING SINCE EARLY ADULTHOOD. THE CONSTELLATION OF BEHAVOIRS OR TRAITS CAUSES EITHER SIGNIFICANT IMPAIRMENT IN SOCIAL OR OCCUPATIONAL FUNCTIONING OR SUBJECTIVE DISTRESS.

MANY FEATURES CHARACTERISTIC OF THE VARIOUS PERSONALITY DISORDERS, SUCH AS BORDERLINE PERSONALITY DISORDER, MAY BE SEEN DURING AN EPISODE OF ANOTHER MENTAL DISORDER, LIKE MAJOR DEPRESSION. THE DIAGNOSIS OF A PERSONALITY DISORDER SHOULD BE MADE ONLY WHEN CHARACTERISTIC FEATURES ARE TYPICAL OF A PERSON'S LONG TERM FUNCTIONING AND ARE NOT LIMITED TO DISCRETE EPISODES OF ILLNESS.

REMEMBER TO ALWAYS RATE THE ITEM AND NOT THE QUESTION.

Frequently the subject will say yes to a question but your clinical judgment will be that the item should be coded "1"or "2". Equally, the subject may say no to a question but there is evidence to the contrary. A rating of "3" should be given only if the subject provided a convincing elaboration or example or there is clear evidence from behavior during interview or from other sources that the item is true.

START by asking the indicated question and follow-up questions as needed. In addition to the questions noted, use probes like:

Give me an extreme example?

Does that happen in a lot of different situations?

Have you always been that way?

Do you think you are more this way than most people?

BORDERLINE PERSONALITY DISORDER

BORDERLINE PERSONALITY DISORDER CRITERIA

	A pervasive pattern of instability of interpersonal relationships, self-image, and affects, and marked impulsivity beginning by early adulthood and present in a variety of contexts, as indicated by five (or more) of the following:	
Have you often become frantic when you thought that someone you really care about was going to leave you? (What have you done? Have you threatened or pleaded with him/her?)	1. Frantic efforts to avoid real or imagined abandonment<br><br>NOTE: DO NOT INCLUDE SUICIDAL OR SELF-MUTILATING BEHAVIOR COVERED IN (5)<br><br>IF CODED "3", GIVE SEVERAL EXAMPLES	O O O O<br>? 1 2 3
Do your relationships with people you really care about have lots of ups and downs? (Tell me about them.)<br><br>Were there times when you thought they were everything you wanted and then other times when you thought they were terrible? (How many relationships were like this?)	2. A pattern of unstable and intense interpersonal relationships characterized by alternating between extremes of idealization and devaluation<br><br>CODE "3" WHEN EITHER ONE PROLONGED RELATIONSHIP OR SEVERAL BRIEFER RELATIONSHIPS IN WHICH THE ALTERNATING PATTERN OCCURS AT LEAST TWICE	O O O O<br>? 1 2 3
Have you abruptly changed your sense of who you are and where you are headed? (Give me some examples of this. Does your sense of who you are often change dramatically? Tell me more about that.)<br><br>Have there been lots of sudden changes in your goals, career plans, religious beliefs (and so on)?	3. Identity disturbance: markedly and persistently unstable self-image or sense of self<br><br>NOTE: DO NOT INCLUDE NORMAL ADOLESCENT UNCERTAINTY<br><br>CODE "3" IF ACKNOWLEDGES TRAIT	O O O O<br>? 1 2 3
Have you often done things impulsively? (What kinds of things?)<br><br>How about...<br>...buying things you couldn't afford?<br>...having sex with people you hardly knew, or "unsafe sex"?<br>...drinking too much or taking drugs?<br>...driving recklessly?<br>...uncontrollable eating?	4. Impulsivity in at least two areas that are potentially self-damaging (e.g., spending, sex, substance abuse, reckless driving, binge-eating)<br><br>NOTE: DO NOT INCLUDE SUICIDAL OR SELF-MUTILATING BEHAVIOR COVERED IN CRITERION (5)<br><br>IF CODED "3", GIVE SEVERAL EXAMPLES INDICATING A PATTERN OF IMPULSIVE BEHAVIOR (NOT LIMITED TO EXAMPLES	O O O O<br>? 1 2 3

IF YES TO ANY OF THE ABOVE: Tell me about that. How often does it happen? What kinds of problems has it caused?	GIVEN). THESE BEHAVIORS CANNOT OCCUR EXCLUSIVELY IN THE CONTEXT OF A MANIC, HYPOMANIC, OR MIXED EPISODE.	
Have you tried to hurt or kill yourself or threatened to do so?<br><br>Have you ever cut, burned, or scratched yourself on purpose? (Tell me about that.)	5. Recurrent suicidal behavior, gestures, or threats, or self-mutilating behavior<br><br>CODE "3" IF TWO OR MORE EVENTS(WHEN NOT IN A MAJOR DEPRESSIVE EPISODE)	O O O O<br>? 1 2 3
Are you a moody person? (Tell me more about that. How long does your "bad" mood last? How often do these changes happen? How suddenly do your moods change?)	6. Affective instability due to a marked reactivity of mood (e.g., intense episodic dysphoria, irritability, or anxiety usually lasting a few hours and only rarely, a few days)<br><br>CODE "3" IF ACKNOWLEDGES TRAIT	O O O O<br>? 1 2 3
Do you often feel empty inside? (Tell me more about this.)	7. Chronic feelings of emptiness<br><br>CODE "3" IF ACKNOWLEDGES TRAIT	O O O O<br>? 1 2 3
Do you often have temper outbursts or get so angry that you lose control? (Tell me about this.)<br><br>Do you hit people or throw things when you get angry? (Tell me about this. Does it happen often?)<br><br>Do even little things get you very angry? (Can you give me an example? Does this happen often?)	8. Inappropriate, intense anger or difficulty controlling anger (e.g., frequent displays of temper, constant anger, recurrent physical fights)<br><br>CODE "3" IF ACKNOWLEDGES TRAIT	O O O O<br>? 1 2 3
Do you get suspicious of other people or feel especially spaced out when you are under a lot of	9. Transient, stress-related paranoid ideation or severe dissociative symptoms	O O O O<br>? 1 2 3

stress? (Tell me about that.)

IF CODED "3", GIVE SEVERAL EXAMPLES THAT DO NOT OCCUR EXCLUSIVELY DURING A PSYCHOTIC DISORDER OR A MOOD DISORDER WITH PSYCHOTIC FEATURES

AT LEAST FIVE BORDERLINE PERSONALITY DISORDER CRITERIA ARE CODED "3" — false

BORDERLINE PERSONALITY DISORDER DIAGNOSTIC SPECIFIERS

LIFETIME SEVERITY:

(Additional questions regarding impairment may be necessary.)

O Mild: (GAF = 61-80) Minimum symptom criteria are met or few, if any, symptoms in excess of those required to make the diagnosis and/or symptoms result in only minimal interference in normal functioning. Either mild disability is present or substantial or unusual effort is needed to function normally.

O Moderate: (GAF = 51-60) Intermediate between "mild" and "severe."

O Severe: (GAF symptoms result in only limited functioning in most aspects of life almost all the time.

O No Information

CHRONOLOGY

IF UNKNOWN: How old were you when you first started having (SYMPTOMS OF BORDERLINE PERSONALITY DISORDER)?

Age at onset of Borderline Personality Disorder (CODE 99 IF UNKNOWN)

End of Borderline Personality Disorder

Go to Antisocial Personality Disorder

ANTISOCIAL PERSONALITY DISORDER

ANTISOCIAL PERSONALITY DISORDER CRITERIA

NOTE: THE CRITERIA LISTED BELOW APPEAR IN A DIFFERENT ORDER THAN IN DSM-IV, THOUGH THE LETTERING HAS BEEN PRESERVED.

B. The individual is at least 18 years old — O 1 O 3

C. There is evidence of Conduct Disorder with onset before age 15 (three (or more) of the following symptoms):

Before you were 15, did you often bully or threaten other kids? (Tell me about that.)

1. Often bullies, threatens or intimidates others — O ? O 1 O 2 O 3

Before you were 15 did you start fights? (How often?)	2. Often initiates physical fights	O ? O 1 O 2 O 3
Before you were 15, did you use a weapon in a fight, like a bat, gun, brick, knife, or broken bottle?	3. Used a weapon that can cause serious harm to others (e.g. bat, gun, brick, knife, or broken bottle)	O ? O 1 O 2 O 3
Before you were 15, did you deliberately try to cause someone physical pain or suffering? (What's the worst thing you ever did?)	4. Physically cruel to people	O ? O 1 O 2 O 3
Before you were 15, did you sometimes hurt animals on purpose? (What's the worst thing you ever did?)	5. Physically cruel to animals	O ? O 1 O 2 O 3
Before you were 15, did you ever forcibly take something from someone by threatening, robbing, or mugging him or her? (Tell me more about that.)	6. Stolen things while confronting a victim (e.g. mugging, purse snatching, extortion, armed robbery)	O ? O 1 O 2 O 3
Before you were 15, did you ever force someone to have sex with you? (Tell me about it.)	7. Forced someone into sexual activity	O ? O 1 O 2 O 3
Before you were 15, did you set fires? (Tell me about that.)	8. Deliberately engaged in fire setting with the intention of causing serious damage	O ? O 1 O 2 O 3
Before you were 15, did you deliberately damage things that weren't yours? (What did you do?)	9. Deliberately destroyed other's property (other than fire setting)	O ? O 1 O 2 O 3

Question	Criterion	Rating
Before you were 15, did you ever break into a house, other building, or car? (Tell me about that.)	10. Broken into someone else's house, building, or car	O ? O 1 O 2 O 3
Before you were 15, did you lie a lot or con other people? (What would you lie about?)	11. Often lied to obtain goods or favors, or to avoid obligations (i.e. "cons" others)	O ? O 1 O 2 O 3
Before you were 15, did you ever steal or shoplift things? (Tell me about it.)	12. Stolen items of nontrivial value without confronting a victim (e.g. shoplifting, stealing without breaking and entering, forgery)	O ? O 1 O 2 O 3
Before you were 15, did you run away from home and stay away over night? (Was that more than once? With whom were you living at the time?)	13. Run away from home over night at least twice while living in a parental, or parental surrogate, home (or once without returning for a lengthy period)	O ? O 1 O 2 O 3

NOTE: THE FOLLOWING TWO ITEMS (14-15) MUST HAVE ONSET BY AGE 13.

Question	Criterion	Rating
Before you were 13, would you often stay out very late, long after the time you were supposed to be home? (How often?)	14. Often stayed out at night despite parental prohibitions, beginning before age 13	O ? O 1 O 2 O 3
Before you were 13, did you often skip school? (How often?)	15. Often truant from school beginning before age 13	O ? O 1 O 2 O 3

AT LEAST THREE CONDUCT DISORDER SYMPTOMS ARE CODED "3"

false

NOTE: IF OVER AGE 18, GIVE CONDUCT DISORDER DIAGNOSIS ONLY IF ANTISOCIAL PERSONALITY DISORDER CRITERIA ARE NOT MET.

A. There is a pervasive pattern of disregard for, and violation of, the rights of others occurring since age 15, as indicated by three (or more) of the following:

Question	Criterion	Rating
Have you done things that are against the law, even if you weren't caught? ...like stealing, selling drugs, cheating in financial deals or prostituting?	1. Failure to conform to social norms with respect to lawful behaviors as indicated by repeatedly performing acts that are grounds for arrest	O ? O 1 O 2 O 3
IF NO: Have you ever been arrested for anything?	IF CODED "3", GIVE SEVERAL EXAMPLES	
Do you lie easily if you need to, to get what you want? (Have you ever used an alias or pretended you were someone else? Have you often "conned" others to get what you want?)	2. Deceitfulness, as indicated by repeated lying, use of aliases, or conning others for personal profit or pleasure	O ? O 1 O 2 O 3
	IF CODED "3", GIVE SEVERAL EXAMPLES	
Do you often do things on the spur of the moment without thinking about how it will effect you or other people? (Why is that?)	3. Impulsivity or failure to plan ahead	O ? O 1 O 2 O 3
Was there ever a time when you had no regular place to live? (For how long?)	IF CODED "3", GIVE SEVERAL EXAMPLES	
Since you were 15 have you been in any fights? (How often)?	4. Irritability and aggressiveness, as seen with repeated physical fights or assaults	O ? O 1 O 2 O 3
Have you ever hit or thrown things at your spouse or partner?(How often?)<br>Have you ever hit a child, yours or someone else's, so hard that he or she had bruises or had to stay in bed or see a doctor?	IF CODED "3", GIVE SEVERAL EXAMPLES	
Did you ever drive a car when you were drunk or high?	5. Reckless disregard for safety of self or others	O ? O 1 O 2 O 3
How many speeding tickets have you gotten?<br>Do you always use protection if you have sex with someone you don't know?	IF CODED "3", GIVE SEVERAL EXAMPLES	

How much of the time in the last five years were you working?<br><br>IF NOT WORKING MORE THAN SIX MONTHS: Why?<br><br>When you were working, did you miss a lot of work? (Why?)<br><br>Did you ever walk off a job without having another one to go to? (How many times did this happen?)<br><br>Have you owed people money and not paid them back? (How often?)<br><br>What about not paying child support, or not giving money to children whom depended on you?	6. Reckless irresponsibility, as indicated by repeated failure to sustain consistent work behavior or honor financial obligations<br><br>IF CODED "3", GIVE SEVERAL EXAMPLES.	O O O O<br>? 1 2 3
IF HAS ACKNOWLEDGED ANTISOCIAL ACTS AS AN ADULT: How do you feel about (LIST ANTISOCIAL ACTS)?	7. Lacks remorse as indicated by being indifferent to, or rationaliz-ing having hurt or mistreated or stolen from another<br><br>CODE "3" IF LACKS REMORSE ABOUT ANTISOCIAL ACTS	O O O O<br>? 1 2 3
	AT LEAST THREE CRITERION A ITEMS ARE CODED "3"	false
	D. The occurrence of antisocial behavior is not exclusively during the course of Schizophrenia or a Manic Episode.	O O O O<br>? 1 2 3
	ANTISOCIAL PERSONALITY DISORDER CRITERIA A, B, C AND D ARE CODED "3"	false

ANTISOCIAL PERSONALITY DISORDER DIAGNOSTIC SPECIFIERS

LIFETIME SEVERITY:

(Additional questions regarding impairment may be necessary.)

O Mild: (GAF = 61-80) Minimum symptom criteria are met or few, if any, symptoms in excess of those required to make the diagnosis and/or symptoms result in only minimal interference in normal functioning. Either mild disability is present or substantial or unusual effort is needed to function normally.

O Moderate: (GAF = 51-60) Intermediate between "mild" and "severe."

O Severe: (GAF symptoms result in only limited functioning in most aspects of life almost all the time.

O No Information

CHRONOLOGY

IF UNKNOWN: How old were you when you first started having (SYMPTOMS OF CONDUCT DISORDER)?	Age at onset of Antisocial Personality/Conduct Disorder (CODE 99 IF UNKNOWN)	
End of Antisocial Personality Disorder	GO TO MODULE M	

Module M: Impulse Control Disorders

INTERMITTENT EXPLOSIVE DISORDER

INTERMITTENT EXPLOSIVE DISORDER CRITERIA

Have you ever lost control of your aggressive impulses which resulted in serious assaultive acts or destruction of property? (What did you do? When did you do it? How often has it occurred?)	A. Several discrete episodes of failure to resist aggressive impulses that result in serious assaultive acts or destruction of property.	O ? O 1 O 2 O 3
Was the aggressive behavior triggered by anything? (Was the degree of aggressiveness out of proportion to the triggering stressor?)	B. The degree of aggressiveness expressed during the episodes is grossly out of proportion to any precipitating psychosocial stressors.	O ? O 1 O 2 O 3
	C. The aggressive episodes are not better accounted for by another mental disorder (e.g., Antisocial Personality Disorder, Borderline Personality Disorder, a Psychotic Disorder, a Manic Episode, Conduct Disorder, or Attention-Deficit/Hyperactivity Disorder) and are not due to the physiological effects of a substance (e.g., a drug of abuse, a medication) or a general medical condition (e.g., head trauma, Alzheimer's Disease).	O ? O 1 O 2 O 3

INTERMITTENT EXPLOSIVE DISORDER CRITERIA A, B, AND C ARE CODED "3"

false

INTERMITTENT EXPLOSIVE DISORDER DIAGNOSTIC SPECIFIERS

LIFETIME SEVERITY:

(Additional questions regarding impairment may be necessary.)

O Mild: (GAF = 61-80) Minimum symptom criteria are met or few, if any, symptoms in excess of those required to make the diagnosis and/or symptoms result in only minimal interference in normal functioning. Either mild disability is present or substantial or unusual effort is needed to function normally.

O Moderate: (GAF = 51-60) Intermediate between "mild" and "severe."

O Severe: (GAF symptoms result in only limited functioning in most aspects of life almost all the time.

O No Information

CURRENT STATE:

O In Remission: During the past two months no significant signs or symptoms of the disturbance.

O Current: Full criteria for an illness must be met at the time of the interview.

O No Information

CHRONOLOGY

How old were you when you first experienced aggressive outbursts?

Age at onset of Intermittent Explosive Disorder (Code 99 IF UNKNOWN)

End of Intermittent Explosive Disorders

Go to Gambling

PATHOLOGICAL GAMBLING

General

Have you ever gambled? (Was there ever a period in your life when you gambled too much? Has gambling ever caused you problems? What problems did it cause? Has anyone ever objected to your gambling? Why?)

IF NO SUGGESTION THAT EVER GAMBLED EXCESSIVELY OR HAD GAMBLING RELATED PROBLEMS, IF NO SUGGESTION THAT EVER GAMBLED EXCESSIVELY OR HAD GAMBLING RELATED PROBLEMS,

CHECK HERE O

AND GO TO PYROMANIA M6

IF HAS ACKNOWLEDGED HAVING PROBLEMS: When in your life were you having the most problems because of your gambling? (How long did that period last?)

IF HAS NOT ACKNOWLEDGED HAVING GAMBLING PROBLEMS BUT GAMBLED EXCESSIVELY: When in your life were you gambling the most? (How long did that period last?)

PATHOLOGICAL GAMBLING CRITERIA

Now I'm going to ask you several questions about that time.

A. Persistent and recurrent maladaptive gambling behavior as indicated by five (or more) of the

following:

Did you ever experience frequent preoccupation with gambling, planning to gamble, or thinking about ways to get money with which to gamble?	1. Preoccupied with gambling (e.g. reliving past gambling experiences, handicapping or planning the next venture or thinking of ways to get money with which to gamble)	O<br>?	O<br>1	O<br>2	O<br>3
Did you find that you needed increasing amounts of money in order to achieve the desired excitement?	2. Needs to gamble with increasing amounts of money in order to achieve the desired excitement	O<br>?	O<br>1	O<br>2	O<br>3
Did you make repeated unsuccessful efforts to control, cut back, or stop gambling?	3. Made repeated unsuccessful efforts to control, cut back, or stop gambling	O<br>?	O<br>1	O<br>2	O<br>3
Did you often experience restlessness or irritability when attempting to cut down or stop gambling?	4. Restless or irritable when attempting to cut down or stop gambling	O<br>?	O<br>1	O<br>2	O<br>3
Did you ever gamble as a way of escaping from your problems or to relieve feelings of helplessness, guilt, anxiety, or depression?	5. Gambles as a way of escaping from problems or relieving a dysphoric mood (e.g. feelings of helplessness, guilt, anxiety, or depression)	O<br>?	O<br>1	O<br>2	O<br>3
Did you ever, after losing money gambling, return another day to get even?	6. After losing money gambling, often returns another day to get even (chasing one's losses)	O<br>?	O<br>1	O<br>2	O<br>3

Did you lie to family, therapists, or others to hide your gambling?	7. Lies to family members, therapists or others to conceal the extent of involvement with gambling	O ? O 1 O 2 O 3
Did you do something illegal such as forgery, fraud, theft, or embezzlement to finance your gambling?	8. Committed illegal acts such as forgery, fraud, theft, or embezzlement to finance gambling	O ? O 1 O 2 O 3
Did you ever jeopardize or lose a significant relationship, job, or educational or career opportunity because of gambling?	9. Jeopardized or lost a significant relationship, job, or educational or career opportunity because of gambling	O ? O 1 O 2 O 3
Did you rely on others to provide money to relieve a gambling debt?	10. Relies on others to provide money to relieve a desperate financial situation caused by gambling	O ? O 1 O 2 O 3
	AT LEAST FIVE CRITERION A ITEMS ARE CODED "3"	false
	B. The gambling behavior is not better accounted for by a Manic episode	O ? O 1 O 2 O 3
	PATHOLOGICAL GAMBLING CRITERIA A AND B ARE CODED "3"	false

PATHOLOGICAL GAMBLING DIAGNOSTIC SPECIFIERS

LIFETIME SEVERITY:

(Additional questions regarding impairment may be necessary.)

- O Mild: (GAF = 61-80) Minimum symptom criteria are met or few, if any, symptoms in excess of those required to make the diagnosis and/or symptoms result in only minimal interference in normal functioning. Either mild disability is present or substantial or unusual effort is needed to function normally.
- O Moderate: (GAF = 51-60) Intermediate between "mild" and "severe."
- O Severe: (GAF symptoms result in only limited functioning in most aspects of life almost all the time.
- O No Information

CURRENT STATE:

O In Remission: During the past two months no significant signs or symptoms of the disturbance.

O Current: Full criteria for an illness must be met at the time of the interview.

O No Information

CHRONOLOGY

How old were you when you first experienced problems with gambling?	Age at onset of Pathological Gambling? (Code 99 IF UNKNOWN)	
End of Gambling	Go to Pyromania	

PYROMANIA

PYROMANIA CRITERIA

Have you ever deliberately and purposefully set a fire on more than one occasion? (What did you burn? When did you do it? How often has it occurred?)	A. Deliberate and purposeful fire setting on more than one occasion.	O ? O 1 O 2 O 3
Did you feel a sense of tension or arousal building up before the act?	B. Tension or affective arousal before the act.	O ? O 1 O 2 O 3
Are you fascinated with, interested in, curious about, or attracted to fire and/or things associated with fire.	C. Fascination with, interest in, curiosity about, or attraction to fire and its situational contexts (e.g., paraphernalia, uses, consequences).	O ? O 1 O 2 O 3
Do you experience pleasure, gratification, or relief when setting fires, or when witnessing or participating in their aftermath?	D. Pleasure, gratification, or relief when setting fires, or when witnessing or participating in their aftermath.	O ? O 1 O 2 O 3
Did you set fires for monetary gain, to express a political idea, to hide criminal activity, to express anger or vengeance, to improve you living circumstances?	E. The fire setting is not done for monetary gain, as an expression of sociopolitical ideology, to conceal criminal activity, to express anger or vengeance, to improve one's living circumstances, in response to a delusion or hallucination, or as a	O ? O 1 O 2 O 3

IF NO: Did you set the fire because of (REFER TO SYMPTOMS OF PSYCHOSIS IF PRESENT)?

result of impaired judgement (e.g., in dementia, Mental Retardation, Substance Intoxication).

F. The fire setting is not better accounted for by Conduct Disorder, a Manic Episode, or Antisocial Personality Disorder.

O ? O 1 O 2 O 3

PYROMANIA CRITERIA A, B, C, D, E AND F ARE CODED "3"

false

PYROMANIA DIAGNOSTIC SPECIFIERS

LIFETIME SEVERITY:

(Additional questions regarding impairment may be necessary.)

O Mild: (GAF = 61-80) Minimum symptom criteria are met or few, if any, symptoms in excess of those required to make the diagnosis and/or symptoms result in only minimal interference in normal functioning. Either mild disability is present or substantial or unusual effort is needed to function normally.

O Moderate: (GAF = 51-60) Intermediate between "mild" and "severe."

O Severe: (GAF symptoms result in only limited functioning in most aspects of life almost all the time.

O No Information

CURRENT STATE:

O In Remission: During the past two months no significant signs or symptoms of the disturbance.

O Current: Full criteria for an illness must be met at the time of the interview.

O No Information

CHRONOLOGY

How old were you when you first experienced problems with (PYROMANIA)?

Age at onset of Pyromania? (Code 99 IF UNKNOWN)

End of Pyromania

Go to Trichotillomania

TRICHOTILLOMANIA

TRICHOTILLOMANIA CRITERIA

Have you ever repeatedly pulled out your hair resulting in noticeable hair loss? (When did you do it? What part of you body did you pull hair from?

A. Recurrent pulling out of one's hair resulting in noticeable hair loss.

O ? O 1 O 2 O 3

How often has it occurred?)

Do you feel an increasing sense of tension immediately before pulling out you hair or when attempting to resist the behavior?	B. An increasing sense of tension immediately before pulling out the hair or when attempting to resist the behavior.	O ? O 1 O 2 O 3
Do you feel pleasure, gratification or relief when pulling out your hair?	C. Pleasure, gratification, or relief when pulling out the hair.	O ? O 1 O 2 O 3
Do you pull out your hair because of a pre-existing skin condition?	D. The disturbance is not better accounted for by another mental disorder and is not due to a general medical condition.	O ? O 1 O 2 O 3
Does the pulling out of your hair cause you significant distress or impairment in social, occupational, or other areas of functioning?	E. The disturbance causes clinically significant distress or impairment in social, occupational, or other areas of functioning.	O ? O 1 O 2 O 3

TRICHOTILLOMANIA CRITERIA A, B, C, D, AND E ARE CODED "3" false

TRICHOTILLOMANIA DIAGNOSTIC SPECIFIERS

LIFETIME SEVERITY:

(Additional questions regarding impairment may be necessary.)

O Mild: (GAF = 61-80) Minimum symptom criteria are met or few, if any, symptoms in excess of those required to make the diagnosis and/or symptoms result in only minimal interference in normal functioning. Either mild disability is present or substantial or unusual effort is needed to function normally.

O Moderate: (GAF = 51-60) Intermediate between "mild" and "severe."

O Severe: (GAF symptoms result in only limited functioning in most aspects of life almost all the time.

O No Information

CURRENT STATE:

O In Remission: During the past two months no significant signs or symptoms of the disturbance.

O Current: Full criteria for an illness must be met at the time of the interview.

O No Information

CHRONOLOGY

How old were you when you first experienced problems with (TRICHOTILLOMANIA)?	Age at onset of Trichotillomania? (Code 99 IF UNKNOWN)	
End of Trichotillomania	Go to Compulsive Buying	

COMPULSIVE BUYING

COMPULSIVE BUYING CRITERIA

Have you ever had trouble with frequent buying of more than you could afford, or frequent shopping for longer periods of time than intended?<br><br>Have you ever be preoccupied with buying or shopping, or experienced irresistible impulses to buy or shop?	A. Frequent preoccupation with buying or impulses to buy that are experienced as irresistible, intrusive, and/or senseless, and/or frequent buying of more than can be afforded, or shopping for longer periods of time than intended?	O ? O 1 O 2 O 3
Have (SYMPTOMS) caused distress, been time consuming, or caused trouble for you or other people?	B. The buying preoccupations, impulses, or behavior causes marked distress, is time consuming, significantly interferes with social or occupational function, or results in financial problems.	O ? O 1 O 2 O 3
	IF THE SUBJECT HAS EVER BEEN MANIC OR HYPOMANIC, ASSESS TEMPORAL RELATIONSHIP OF BUYING WITH HYPOMANIC OR MANIC SYMPTOMS.	
Have the (BUYING/SHOPPING SYMPTOMS) occurred only when you were also experiencing hypomanic or manic symptoms?	C. Buying or shopping symptoms do not occur exclusively during periods of hypomania or mania.	O ? O 1 O 2 O 3

COMPULSIVE BUYING CRITERIA A, B, AND C ARE CODED "3"	false

COMPULSIVE BUYING DIAGNOSTIC SPECIFIERS

LIFETIME SEVERITY:

(Additional questions regarding impairment may be necessary.)

O Mild: (GAF = 61-80) Minimum symptom criteria are met or few, if any, symptoms in excess of those required to make the diagnosis and/or symptoms result in only minimal interference in normal functioning. Either mild disability is present or substantial or unusual effort is needed to function normally.

O Moderate: (GAF = 51-60) Intermediate between "mild" and "severe."

O Severe: (GAF symptoms result in only limited functioning in most aspects of life almost all the time.

O No Information

CURRENT STATE:

O In Remission: During the past two months no significant signs or symptoms of the disturbance.

O Current: Full criteria for an illness must be met at the time of the interview.

O No Information

CHRONOLOGY

How old were you when you first experienced problems with (COMPULSIVE BUYING)?	Age at onset of Compulsive Buying? (Code 99 IF UNKNOWN)	
End of Compulsive Buying	Go to Kleptomania	

KLEPTOMANIA

KLEPTOMANIA CRITERIA

Have you ever impulsively stolen objects that you didn't need? (What did you steal? When did you do it? How often has it occurred?)	A. Recurrent failure to resist impulses to steal objects that are not needed for personal use or for their monetary value.	O ? O 1 O 2 O 3
Do you feel a sense of tension building up before you steal something?	B. Increasing sense of tension immediately before committing the theft.	O ? O 1 O 2 O 3
Do you feel a sense of pleasure or relief after you have stolen things?	C. Pleasure, gratification, or relief at the time of committing the theft.	O ? O 1 O 2 O 3

Did you steal things only because you were angry with someone or trying to get back at someone?	D. The stealing is not committed to express anger or vengeance and is not in response to a delusion or a hallucination.	O O O O<br>? 1 2 3
Have the (KLEPTOMANIA SYMPTOMS) occurred only when you were also experiencing hypomanic or manic symptoms?	E. The stealing is not better accounted for by Conduct Disorder, a Manic Episode, or Antisocial Personality Disorder.	O O O O<br>? 1 2 3
	KLEPTOMANIA CRITERIA A, B, C, D, AND E ARE CODED "3"	false

COMPULSIVE KLEPTOMANIA DIAGNOSTIC SPECIFIERS

LIFETIME SEVERITY:

(Additional questions regarding impairment may be necessary.)

O Mild: (GAF = 61-80) Minimum symptom criteria are met or few, if any, symptoms in excess of those required to make the diagnosis and/or symptoms result in only minimal interference in normal functioning. Either mild disability is present or substantial or unusual effort is needed to function normally.

O Moderate: (GAF = 51-60) Intermediate between "mild" and "severe."

O Severe: (GAF symptoms result in only limited functioning in most aspects of life almost all the time.

O No Information

CURRENT STATE:

O In Remission: During the past two months no significant signs or symptoms of the disturbance.

O Current: Full criteria for an illness must be met at the time of the interview.

O No Information

CHRONOLOGY

How old were you when you first experienced problems with (KLEPTOMANIA)?	Age at onset of Kleptomania? (Code 99 IF UNKNOWN)	
End of Module M	Go to Module N	

Module N: Affective Spectrum Disorders

IRRITABLE BOWEL SYNDROME

Criteria

Have you ever had a period of one month or more, during which you had frequent pain in your abdomen	A. A period of one month or more of continuous or recurrent abdominal pain, relieved with defecation,	O O O O<br>? 1 2 3

that was relieved by having a bowel movement, or pain that was associated with constipation or diarrhea? (When was it? How long did it last?)	or associated with changes in frequency or consistency of stool.	
Have you ever had a period of one month or more when you had any of the following symptoms:	B. Three (or more) of the following persistently present for at least one month:	
...a persistent change in the frequency of your bowel movements?	1. Altered stool frequency	O ? O 1 O 2 O 3
...a persistent change in the consistency of your bowel movements, so that they were unusually hard or unusually loose or watery?	2. Altered stool form (hard or loose/ watery)	O ? O 1 O 2 O 3
...persistent difficulty in having bowel movements, such as having to strain, feeling frequent urgency to have a bowel movement, or a feeling that you had not completely evacuated the bowel after a movement?	3. Altered stool passage (straining or urgency, feeling of incomplete evacuation)	O ? O 1 O 2 O 3
...persistent white or yellow mucus in you stools?	4. Passage of mucus	O ? O 1 O 2 O 3
...a persistent bloated feeling in your abdomen?	5. Bloating or feeling of abdominal distention	O ? O 1 O 2 O 3

	AT LEAST THREE CRITERION B ITEMS ARE CODED "3"	false
	EITHER CRITERION A OR B IS CODED "3"	false
	DETERMINE WHETHER ETIOLOGIC ROLE OF AN ORGANIC FACTOR IN IRRITABLE BOWEL SYNDROME	
Did you see a doctor about these symptoms? Did anyone tell you the diagnosis or explanation for your symptoms?	C. The symptoms cannot be attributed to a known organic etiology, such as inflammatory bowel disease or infection.	O 1 O 3
	IRRITABLE BOWEL SYNDROME CRITERIA A OR B AND C ARE CODED "3"	false

IRRITABLE BOWEL SYNDROME DIAGNOSTIC SPECIFIERS

Lifetime Severity

(Additional questions regarding impairment may be necessary.)

O Mild: (GAF = 61-80) Minimum symptom criteria are met or few, if any, symptoms in excess of those required to make the diagnosis and/or symptoms result in only minimal interference in normal functioning. Either mild disability is present or substantial or unusual effort is needed to function normally.

O Moderate: (GAF = 51-60) Intermediate between "mild" and "severe."

O Severe: (GAF symptoms result in only limited functioning in most aspects of life almost all the time.

O No Information

Number of Episodes

How many separate times have you experienced (IRRITABLE BOWEL SYMPTOMS)?	Total number of episodes of Irritable Bowel Syndrome (CODE 99 IF TOO NUMEROUS TO COUNT)	

Chronology

How old were you when you first experienced problems with (IRRITABLE BOWEL SYMPTOMS)?	Age at onset of Irritable Bowel Syndrome? (Code 99 IF UNKNOWN)	

End Irritable Bowel Syndrome — Continue below with Migraine

MIGRAINE

MIGRAINE CRITERIA

Have you ever had an attack of moderate or severe headache, lasting between two hours and three days? (Tell me about it. Do you have separate attacks with pain-free intervals in between?)	A. Headache attacks, lasting 2 to 72 hours (untreated or unsuccessfully treated).	O ? O 1 O 2 O 3

During the headache attack...	B. The headache has at least two of the following characteristics:	
...where in your head is the pain located? (Only on one side?)	1. Unilateral location	O O O O<br>? 1 2 3
...is the headache pain throbbing or steady? (Does the pain ever pulsate?)	2. Pulsating quality	O O O O<br>? 1 2 3
...is the pain so bad that it becomes difficult or impossible for you to continue in your daily activities? (How often is it this severe?)	3. Moderate or severe intensity (inhibits or prohibits daily activities)	O O O O<br>? 1 2 3
	AT LEAST TWO B CRITERION ITEMS ARE CODED "3"	false
During these headache attacks...	C. During the headache, at least one of the following is present:	
...do you experience nausea or vomiting? (How often?)	1. Nausea and/or vomiting	O O O O<br>? 1 2 3
...do bright lights or loud sounds make the headache worse?	2. Photophobia or phonophobia	O O O O<br>? 1 2 3

	AT LEAST ONE C CRITERION ITEM IS CODED "3"	false
Have you had more than five such attacks during a one-year period throughout which you experienced (SYMPTOMS IN B AND C ABOVE)?	D. At least five such attacks in one-year period meeting criteria B and C above.	O ? O 1 O 2 O 3
Do you ever experience episodes of neurological symptoms, lasting less than one hour, such as disturbance of your vision, weakness or numbness on one side of your body, or difficulty speaking?	E. Aura (which may or may not be followed by headache): an attack of neurological symptoms, unequivocally localizable to cerebral cortex or brain stem, usually gradually developed over 5-30 minutes and usually lasting less than 60 minutes.	O ? O 1 O 2 O 3
IF YES: Tell me about it. How long does it last? Do you develop a headache after the (AURA)? If not, have you experienced episodes of the (AURA) recurring over months or years of time?		
	DETERMINE WHETHER ETIOLOGIC ROLE OF AN ORGANIC FACTOR IN MIGRAINE	
During the time that you had these headaches or (AURAS) were you taking any drugs or medicines? Drinking a lot? Were you given a medical diagnosis by a doctor?	F. It cannot be established that an organic factor independent of migraine caused the disturbance.	O ? O 1 O 2 O 3
	MIGRAINE CRITERIA A, B, C, D, AND F ARE CODED "3" (HEADACHES) OR CRITERIA E AND F ARE CODED "3" (AURA WITHOUT HEADACHES)	false

MIGRAINE DIAGNOSTIC SPECIFIERS

Lifetime Severity

(Additional questions regarding impairment may be necessary.)

O Mild: (GAF = 61-80) Minimum symptom criteria are met or few, if any, symptoms in excess of those required to make the diagnosis and/or symptoms result in only minimal interference in normal functioning. Either mild disability is present or substantial or unusual effort is needed to function normally.

O Moderate: (GAF = 51-60) Intermediate between "mild" and "severe."

O Severe: (GAF symptoms result in only limited functioning in most aspects of life almost all the time.

O No Information

Features/Subtypes

O With Auras: Subject endorsed criterion E.

O Without Auras: Subject did not endorse criterion E.

O No Information

Chronology

How old were you when you first experienced problems with (MIGRAINE)?	Age of onset of Migraine? (Code 99 IF UNKNOWN)	
End of Migraine	END OF SCID	
